# Receptor-based pharmacophore modeling, molecular docking, synthesis and biological evaluation of novel VEGFR-2, FGFR-1, and BRAF multi-kinase inhibitors

**DOI:** 10.1186/s13065-024-01135-0

**Published:** 2024-02-23

**Authors:** Heba T. Abdel-Mohsen, Marwa A. Ibrahim, Amira M. Nageeb, Ahmed M. El Kerdawy

**Affiliations:** 1 Chemistry of Natural and Microbial Products Department, Pharmaceutical and Drug Industries Research Institute, National Research Centre, Dokki, P.O. 12622, Cairo, Egypt; 2 Department of Pharmaceutical Chemistry, Faculty of Pharmacy, Cairo University, Kasr El-Aini Street, P.O. 11562, Cairo, Egypt; 3 High Throughput Molecular and Genetic Technology Lab, Center of Excellence for Advanced Sciences, Biochemistry Department, Biotechnology Research Institute, National Research Centre, Dokki, P.O. 12622, Cairo, Egypt; 4 School of Pharmacy, College of Health and Science, University of Lincoln, Joseph Banks Laboratories, Green Lane, Lincoln, Lincolnshire UK

**Keywords:** VEGFR-2, FGFR-1, BRAF, Multi-kinase, Anti-cancer, CADD, Pharmacophore, Molecular docking

## Abstract

A receptor-based pharmacophore model describing the binding features required for the multi-kinase inhibition of the target kinases (VEGFR-2, FGFR-1, and BRAF) were constructed and validated. It showed a good overall quality in discriminating between the active and the inactive in a compiled test set compounds with F1 score of 0.502 and Mathew’s correlation coefficient of 0.513. It described the ligand binding to the hinge region Cys or Ala, the glutamate residue of the Glu-Lys αC helix conserved pair, the DFG motif Asp at the activation loop, and the allosteric back pocket next to the ATP binding site. Moreover, excluded volumes were used to define the steric extent of the binding sites. The application of the developed pharmacophore model in virtual screening of an in-house scaffold dataset resulted in the identification of a benzimidazole-based scaffold as a promising hit within the dataset. Compounds **8a-u** were designed through structural optimization of the hit benzimidazole-based scaffold through (un)substituted aryl substitution on 2 and 5 positions of the benzimidazole ring. Molecular docking simulations and ADME properties predictions confirmed the promising characteristics of the designed compounds in terms of binding affinity and pharmacokinetic properties, respectively. The designed compounds **8a-u** were synthesized, and they demonstrated moderate to potent VEGFR-2 inhibitory activity at 10 µM. Compound **8u** exhibited a potent inhibitory activity against the target kinases (VEGFR-2, FGFR-1, and BRAF) with IC_50_ values of 0.93, 3.74, 0.25 µM, respectively. The benzimidazole derivatives **8a-u** were all selected by the NCI (USA) to conduct their anti-proliferation screening. Compounds **8a** and **8d** resulted in a potent mean growth inhibition % (GI%) of 97.73% and 92.51%, respectively. Whereas compounds **8h**, **8j**, **8k**, **8o**, **8q**, **8r**, and **8u** showed a mean GI% > 100% (lethal effect). The most potent compounds on the NCI panel of 60 different cancer cell lines were progressed further to NCI five-dose testing. The benzimidazole derivatives **8a**, **8d**, **8h**, **8j**, **8k**, **8o**, **8q**, **8r** and **8u** exhibited potent anticancer activity on the tested cell lines reaching sub-micromolar range. Moreover, **8u** was found to induce cell cycle arrest of MCF-7 cell line at the G2/M phase and accumulating cells at the sub-G1 phase as a result of cell apoptosis.

**Supplementary Information:**

The online version contains supplementary material available at 10.1186/s13065-024-01135-0.

## Introduction

Protein kinases are a class of phosphotransferases that play a fundamental role in the regulation of different cellular processes such as cellular survival, growth, proliferation, migration, and apoptosis [1]. More than 30% of cellular proteins are phosphorylated by protein kinases. Protein kinases catalyse the transfer of a gamma phosphate group from ATP to an acceptor amino acid (Tyrosine, serine, or threonine) in a substrate protein [1]. Therefore, protein kinases are categorized mainly into two main categories, the tyrosine kinases such as vascular endothelial growth factor receptor (VEGFR), fibroblast growth factor receptor (FGFR) and epidermal growth factor receptor (EGFR) and the serine/threonine kinases such as rapidly accelerated fibrosarcoma (RAF) kinases [2, 3]. In cancer, several protein kinases are dysregulated resulting in the uncontrolled growth, survival, and metastasis of tumour cells [2, 4, 5]. Hence, targeting protein kinases has received a remarkable attention in recent years for the discovery of new targeted chemotherapeutic agents for cancer treatment [6–8]. Based on the fact that cancer is regulated by multiple pathways that can compensate for one another when a single pathway is blocked, targeting multiple kinases is a more efficient strategy than targeting a single kinase [9]. Moreover, multi-kinase inhibition has numerous advantages, such as increasing potency due to its synergistic effect, reducing probable polypharmacy toxicity, avoiding pharmacokinetics incompatibilities, and enhancing selectivity [9, 10].

Angiogenesis, the formation of new blood vessels, plays an essential role in the growth and metastasis of tumour cells [11]. Hence, targeting protein kinases that initiate and sustain the angiogenic process is a prominent approach in cancer treatment [12]. Vascular endothelial growth factor (VEGF) and its receptors (VEGFRs) is a tyrosine kinase system that plays a curial role in angiogenesis both in the physiological as well as pathological conditions [13–15]. In comparison to healthy tissues, VEGFR-2, in particular, is overexpressed in various types of cancer such as malignant melanoma, breast cancer, hepatocellular carcinoma, colon cancer, etc., [16, 17]. In addition, hFGFR family is a group of four isoforms; FGFR-1 to FGFR-4 that are expressed on the cell membrane and participate in various vital physiological and pathological processes, such as proliferation, differentiation, cell migration, survival, as well as angiogenesis [18]. Binding of FGFR to its growth factor (FGF) results in its dimerization and phosphorylation of its intracellular kinase domain resulting in the initiation of a series of downstream signalling pathways. FGFRs overexpression has been reported in different types of solid tumours, for instance, FGFR-1 is amplified in breast and non-small cell lung cancers [19]. Hence, it is believed that FGFRs inhibition by small molecules that competitively bind to the ATP binding pocket is an attractive tactic for the design of novel targeted anticancer agents [20].

Moreover, when the main pro-angiogenic factors VEGF and FGF bind to their target receptors, they result in an activation of the mitogen-activated protein kinase (MAPK) signalling pathway [21]. Downstream signalling of this pathway leads to activation of RAS proteins which in turn causes subsequent activation of RAF kinases [22, 23]. RAF kinases are an intracellular serine/threonine family mediating several transcriptional factors leading to cell growth, survival, and proliferation [22, 23]. Among the RAF family, BRAF is the most sensitive isoform to activation and mutation [24].

**Fig. 1:**
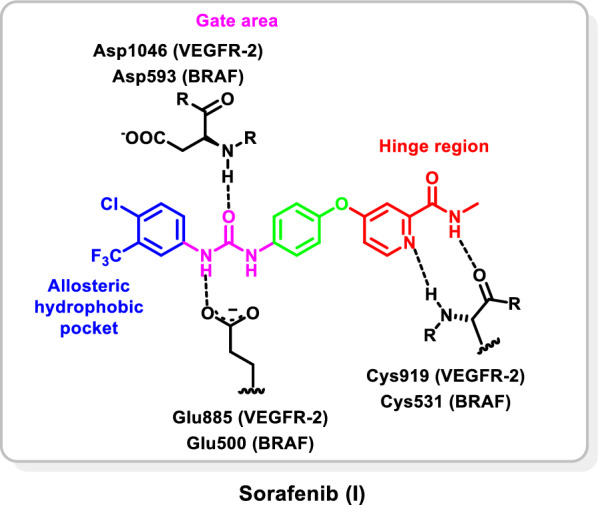
Structure of sorafenib (**I**) in the binding sites of VEGFR-2 (PDB ID: 4ASD) and BRAF (PDB ID: 1UWH)

The X-ray crystallographic structures of kinases such as VEGFR-2, FGFR-1 and BRAF demonstrated that their kinase domain comprises a smaller N-terminal lobe, larger C-terminal lobe, and an in-between ATP binding region which can be partitioned further into front pocket (front cleft or hinge region), gate area, and back cleft (allosteric back pocket). At the beginning of the C-terminal lobe there is an activation loop (A-loop) that is characterized by a highly conserved aspartate-phenylalanine-glycine (DFG) motif. Based on the 3D orientation of the DFG motif, the A-loop can exist in different conformations resulting in the existence of the protein kinase in its active (DFG-in) or inactive (DFG-out) conformations. In the catalytic cycle, the protein kinase switches between both open and closed conformations [25, 26]. Analysis of the binding modes of the co-crystallized protein kinase inhibitors (PKIs) at their target proteins demonstrated that PKIs can be classified according to their binding modes into six different types [26–30]. Among them, type II inhibitors are regarded as promising ones performing their antagonistic activity on the inactive (DFG-out) conformation accommodating into the hinge region, the gate area and extend further to the less conservative back pocket enhancing their affinity, selectivity, and residence time [31]. For example, sorafenib (**I**) (Fig. 1) is a VEGFR-2 (PDB ID: 4ASD) and BRAF (PDB ID: 1UWH) inhibitor in which the picolinamide moiety (coloured red in Fig. 1) occupies the front pocket and performs hydrogen bonding interaction with Cys919 (VEGFR-2)/Cys531 (BRAF). The ureido moiety (coloured pink in Fig. 1) extends through the gate area and forms by its NH group a hydrogen bond with the carboxylate group of αC-helix Glu885 (VEGFR-2)/Glu593 (BRAF), furthermore, the oxygen atom of the ureido moiety forms a hydrogen bond with the N–H group of DFG’s Asp1046 (VEGFR-2)/Asp500 (BRAF). In addition, sorafenib (**I**) extends into the hydrophobic back pocket by a disubstituted phenyl group (coloured blue in Fig. 1) achieving multiple hydrophobic interactions with the surrounding residues [32, 33].

**Fig. 2:**
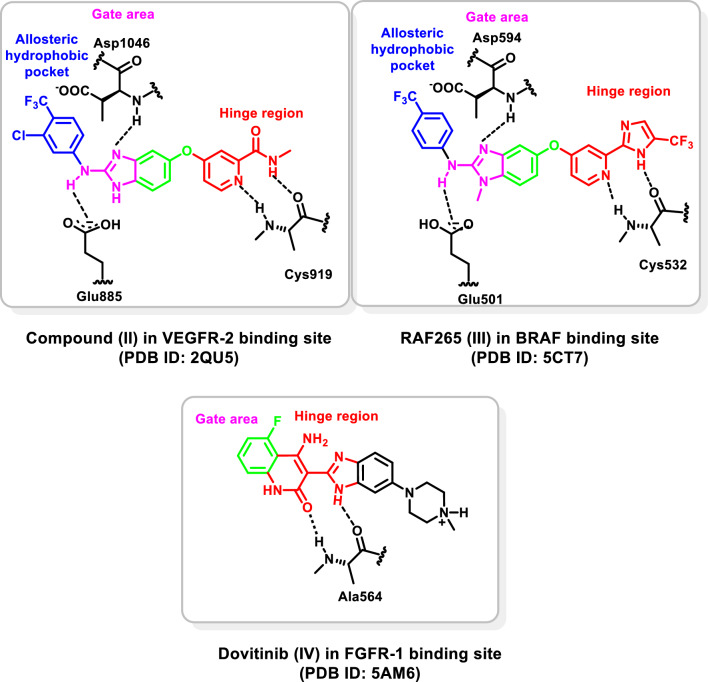
Benzimidazole-based multi-kinase inhibitors and their interactions in their targets’ kinase domain

Benzimidazole is a privileged heterobicyclic scaffold representing the core of several reported targeted chemotherapeutic agents that possess potent protein kinase inhibitory activity [34–37]. Potashman et al*.*, [38] demonstrated the potent VEGFR-2 inhibitory activity and anti-proliferative properties of a series of benzimidazole derivatives. Compound **II** (PDB ID: 2QU5) (Fig. 2) is a representative for this series showing K_i_ of 8.7 nM against VEGFR-2. In addition, RAF265 (**III**) (PDB ID: 5CT7) (Fig. 2) is a potent dual BRAF/VEGFR-2 inhibitor that showed a potent activity against melanoma and colorectal cancer [39, 40]. Dovitinib (**IV**) (PDB ID: 5AM6) (Fig. 2) is a benzimidazole-based type I multi-kinase inhibitor of VEGFR1-3 (IC_50_ = 8–13 nM), FGFR1-3 (IC_50_ = 8–9 nM), and other receptor tyrosine kinases, that showed a potent activity against a wide range of cancers [41–43].

In recent years, significant advancements have been achieved in PKIs exploration, mostly attributed to the utilization of computational techniques [44, 45]. These methods have proven instrumental in delivering valuable insights into diverse protein kinase structures and inhibitors [44, 45]. In computer-aided drug design (CADD), two primary strategies are commonly employed; structure-based drug design (SBDD) and ligand-based drug design (LBDD). These approaches allow researchers to predict and optimize the properties and activities of molecules even before they are synthesized and tested in the laboratory [46].

Due to the continuous resistance development by cancer cells on one hand and the more satisfactory effect and less drawbacks achieved by the concurrent targeting of multiple protein kinases on the other hand [47, 48], there is a continuous demand for developing small molecules that target more than one protein kinase simultaneously (multi-kinase inhibitors).

Encouraged by these facts, the ultimate goal of the current investigation is to extract the common pharmacophoric features required for achieving multi-kinase inhibition of the target kinases; VEGFR-2, FGFR-1, and BRAF. The generated pharmacophore model will be then used to virtually screen a set of in-house synthetically feasible tailored diverse scaffolds to select those satisfy the pharmacophoric features of the target multi-kinase activity. Scaffolds satisfy the constructed pharmacophore model will be structurally optimized to enhance their target kinase binding. Molecular docking will be then used to confirm the ability of the designed derivatives to perform the essential interactions with the three target kinases. Afterwards, the in silico promising derivatives will be synthesized and evaluated for their biochemical inhibitory activity against the target kinases as well as for their cytotoxic activity on several cancer cell lines. Finally, the most potent candidate will be further analyzed for its effect on cell cycle progression and apoptosis induction.

## Results and discussion

### Molecular modeling study

For the intended study, a common 3D multi-kinase pharmacophore model for type II kinase inhibitors of the target kinases (VEGFR-2, FGFR-1, and BRAF) was constructed using the receptor-based pharmacophore technique. The generated pharmacophore was then validated for its ability to discriminate between active and inactive compounds of the different kinases of interest using a pre-compiled test set of active inhibitors and inactive decoys. Next, the validated pharmacophore was used to virtually screen several in-house datasets with diverse scaffolds and the most promising scaffold was used as a starting point to develop several optimized derivatives with potential synthetic feasibility which should be, by design, multi-kinase inhibitors of the target kinases. Finally, molecular docking simulations were used to investigate the ability of the designed compounds to bind to the active sites of the target kinases accomplishing the key interactions responsible for the kinase inhibitory activity. Promising compounds were passed to the next chemical synthesis step.

#### Common 3D multi-kinase receptor-based pharmacophore model generation

##### X-ray crystallographic structures

Several X-ray crystal structures for the target kinases (VEGFR-2, FGFR-1, and BRAF) are available in the protein data bank [49]. For the current work, we are focusing on the design of type II kinase inhibitors due to their reported superiority [20]. Thus, representative structures were selected that are co-crystalized with potent structurally diverse type II kinase inhibitors which bind to the inactive DFG-out kinase conformation occupying the front cleft (hinge region), the gate area and extend beyond the gatekeeper into the hydrophobic allosteric back cleft [50]. Different kinase structures co-crystalized with the same ligand were also preferred. Hence, the X-ray crystallographic structures of VEGFR-2 (PDB ID: 3VHE, 3VNT, and 3VO3), FGFR-1 (PDB ID: 4V01 and 3RHX), and BRAF (PDB ID: 4DBN and 6B8U) were downloaded from the protein data bank [49]. The protein structures were then prepared and aligned using their alpha carbons (See Additional file 1: Section 1: computational studies for further details).

##### Manual 3D receptor-based pharmacophore models generation

Using the aligned prepared protein structures, several manual 3D pharmacophores were generated to describe the common inhibitors’ interactions. The main common ligand-target interactions involve H-bonding interaction with the hinge region Cys919, Ala564, and Cys532 in VEGFR-2, FGFR-1, and BRAF, respectively, H-bonding with DFG Asp1046, Asp641, and Asp594 in VEGFR-2, FGFR-1, and BRAF, respectively, and H-bonding with αC-helix Glu885, Glu531, and Glu501 in VEGFR-2, FGFR-1, and BRAF, respectively. These interactions were described by hydrogen bond acceptor, donor, and acceptor features, respectively, with their corresponding projected features (Site features). In addition to hydrophobic interactions with the hydrophobic allosteric back pocket in each protein structure which were described by a broader hydrophobic pharmacophoric feature. In addition, excluded volumes were employed to define the binding sites’ steric extent. The different 3D pharmacophores obtained are the result of several combinations of the different pharmacophoric features (in terms of their number and volume) giving a set of 17 combinations (See Additional file 1: Section 1: computational studies for further details).

#### Pharmacophore model selection and validation

Selection of the best 3D pharmacophore model was carried out with the aid of a compiled test set of 2387 compounds (Table 1) (See Additional file 1: Section 1: computational studies for further details).

**Table 1:** Distribution of the test set active inhibitors and inactive decoys for the target kinases VEGFR-2, FGFR-1, and BRAF

Target kinase	Total test set compounds	Active inhibitors	Inactive decoys
VEGFR-2	827	26	801
FGFR-1	620	20	600
BRAF	940	27	913
Total	2387	73	2314

The 3D pharmacophore ability to discriminate between the test set active and inactive compounds was used to evaluate its quality which was assessed based on its collective results on the whole test set. For each 3D pharmacophore, the total number of true positives **TP**_**t**_, false positives **FP**_**t**_, true negatives **TN**_**t**_, and false negatives **FN**_**t**_ were determined from its performance on each kinase test set (See Additional file 1, for further details) and were used in calculating the different assessment metrics; Sensitivity **Se**, specificity **Sp**, yield of actives **Ya**, enrichment **E**, accuracy **Acc**, discrimination ratio **DR**, F1 score **F1** and Mathew’s correlation coefficient **MCC** to evaluate the models’ performance (Table 2 (Metric’s values of the best performing pharmacophore model (**Ph4-4**) are shown in bold) and see Additional file 1: Section 1: computational studies for further details).

**Table 2:** The collective assessment metrics of the generated pharmacophore models

Ph4 no.	Se	Sp	Ya	E	Acc	DR	F1	MCC
Ph4-1	0.753	0.955	0.344	11.240	0.948	0.789	0.4721	0.4875
Ph4-2	0.753	0.958	0.362	11.832	0.952	0.786	0.4889	0.5017
Ph4-3	0.753	0.959	0.369	12.070	0.953	0.785	0.4955	0.5073
**Ph4-4**	**0.753**	**0.961**	**0.377**	**12.318**	**0.954**	**0.784**	**0.5023**	**0.5131**
Ph4-5	0.740	0.962	0.383	12.523	0.956	0.769	0.5047	0.5128
Ph4-6	0.726	0.964	0.387	12.650	0.956	0.753	0.5048	0.5106
Ph4-7	0.753	0.942	0.289	9.465	0.936	0.800	0.4183	0.4422
Ph4-8	0.753	0.935	0.267	8.730	0.929	0.806	0.3943	0.4220
Ph4-9	0.767	0.919	0.230	7.535	0.915	0.835	0.3544	0.3908
Ph4-10	0.767	0.936	0.273	8.932	0.930	0.820	0.4029	0.4318
Ph4-11	0.767	0.924	0.242	7.927	0.920	0.830	0.3684	0.4027
Ph4-12	0.808	0.905	0.211	6.915	0.902	0.893	0.3352	0.3822
Ph4-13	0.849	0.773	0.105	3.448	0.775	1.099	0.1876	0.2486
Ph4-14	0.849	0.824	0.132	4.323	0.825	1.031	0.2288	0.2918
Ph4-15	0.849	0.861	0.161	5.279	0.860	0.987	0.2713	0.3328
Ph4-16	0.479	0.952	0.240	7.839	0.938	0.504	0.3196	0.3100
Ph4-17	0.425	0.986	0.484	15.838	0.969	0.431	0.4526	0.4375

(Metric’s values of the best performing pharmacophore model (**Ph4-4**) are shown in bold)

**Fig. 3:**
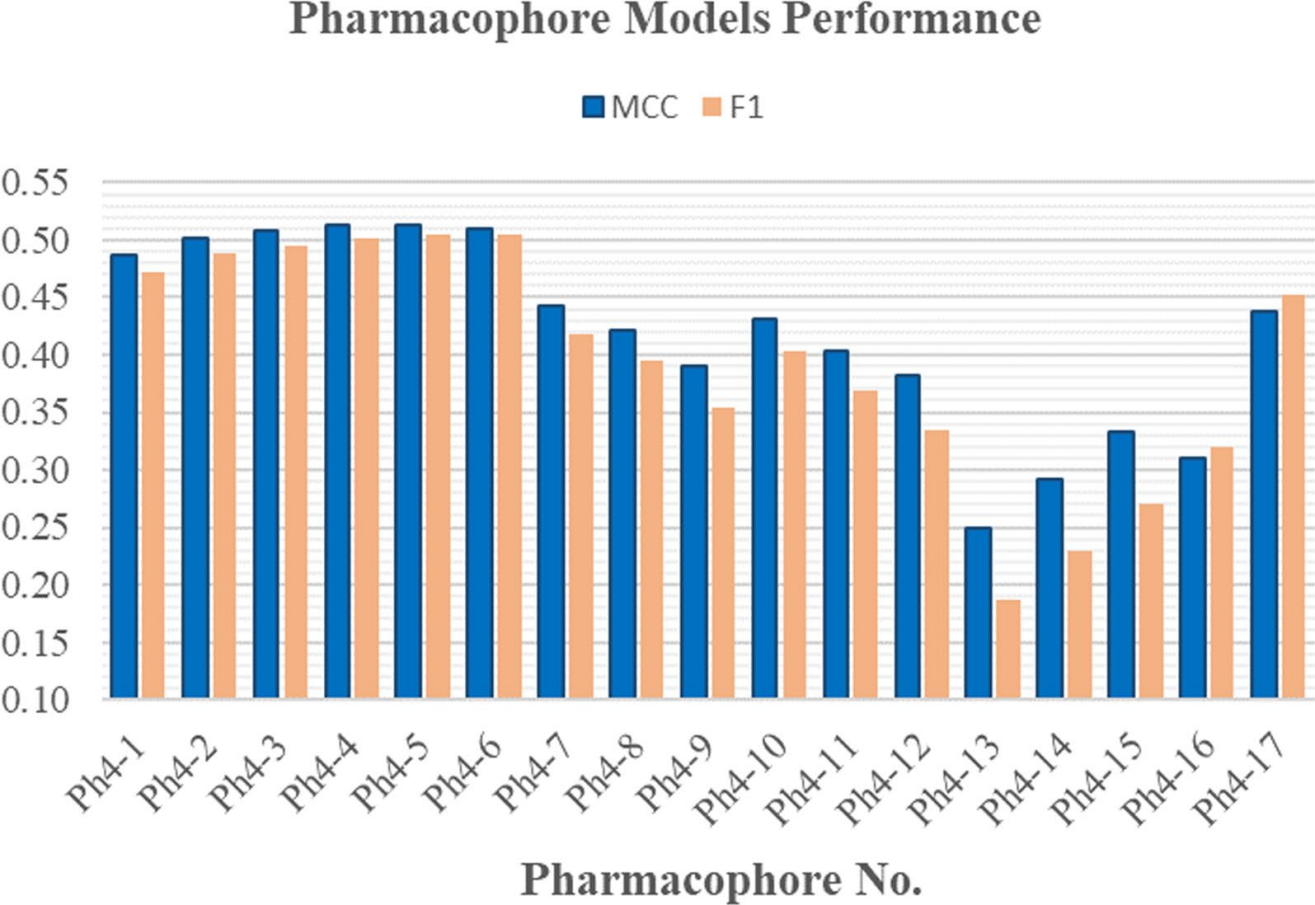
3D pharmacophore models’ performance represented by their F1 score **F1** and Mathew’s correlation coefficient **MCC** values

As can be seen in Table 2, Ph4-16 and Ph4-17 showed low sensitivity (0.479 and 0.425, respectively) meaning that they yielded a low number of true positives, however, they showed good specificity (0.952 and 0.986, respectively) and so could discard decoys and correctly consider them as inactive compounds, so these two models are biased towards inactive compounds and that is reflected in their low F1 score and MCC (0.3196 and 0.3100, respectively, for Ph4-16 and 0.4526 and 0.4375, respectively, for Ph4-17) (Table 2 and Fig. 3). On the contrary, models Ph4-12 to Ph4-15 showed good sensitivity (0.808, 0.849, 0.849 and 0.849, respectively) meaning that they yielded a high number of true positives, however, they showed low specificity (0.905, 0.773, 0.824 and 0.861, respectively) and so could not discard decoys properly and predict large number of decoys as active compounds, so these models are biased towards active compounds and this is reflected in their low MCC (0.3822, 0.2486, 0.2918 and 0.3328, respectively) (Table 2 and Fig. 3). Models Ph4-1 to Ph4-10 showed a balance between sensitivity and specificity with a sensitivity range of (0.726–0.767) and a specificity range of (0.919–0.964) indicating that the models are not biased towards either of actives or decoys (Table 2 and Fig. 3).

**Fig. 4:**
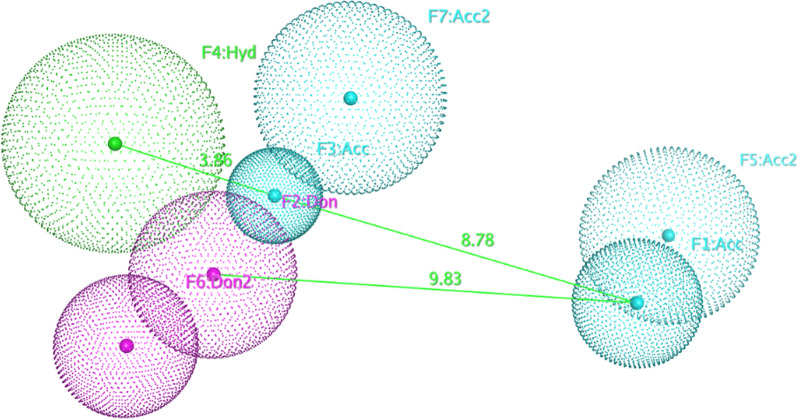
The selected pharmacophore model (**Ph4-4**) (distances in Å)

Model **Ph4-4** (Fig. 4) was selected as the best model because it showed the best overall performance on the test set. **Ph4-4** selected 146 hits out of 2387 compounds of which 55 compounds were true positives (*Se* = 0.753) and it assigned 2241 compounds as inactive compounds from which 2223 are true negatives (*Sp* = 0.961). It showed a yield of actives **Ya** of 0.377 and an enrichment value **E** of 12.318 proving the success of the pharmacophore model in improving the selection process of active compounds via the virtual screening technique versus random methods. Moreover, Ph4-4 model had an accuracy **Acc** of 0.954 emphasizing that it can accurately identify active compounds while dismissing the inactive ones. Lastly, it had a discrimination ratio **DR** of 0.784 which shows that this model has a high prediction potential for discriminating between the active and the inactive compounds. Moreover, Ph4-4 showed **F1 score** of 0.5023 and the highest **MCC** of 0.5131 indicating its good overall quality (Table 2 and Fig. 3).

Figure 4 shows the selected 3D pharmacophore model, **Ph4-4**, its pharmacophoric features, and inter-feature distances (in Å) in 3D space. **Ph4-4** consists of 4 main features [**F1**-**F4**]. Feature 1 (**F1:Acc**), a hydrogen bond acceptor, where ligands bind to the hinge region Cys or Ala, and the direction of this hydrogen bond acceptor lone pair is indicated by its projected site point feature (**F5:Acc2**). Feature 2 (**F2:Don**), a hydrogen bond donor, describing the feature required for binding to the glutamate residue of the Glu-Lys αC helix conserved pair and its projected site point feature (**F6:Don2**) indicates the direction of the hydrogen bond donor hydrogen. Feature 3 (**F3:Acc**), a hydrogen bond acceptor mapping where the ligands bind to the DFG motif Asp at the activation loop, in addition to its projected site point feature (**F7:Acc2**) indicating the direction of the hydrogen bond acceptor lone pair. Finally, the broadest feature (**F4:Hyd**) where ligands’ hydrophobic moieties occupy the allosteric back pocket next to the ATP binding site. Moreover, thirty excluded volumes (Not shown in Fig. 4 for clarity) were also added to this pharmacophore to define the steric extent of the binding sites and to restrict the highly flexible compounds (if any) to bind in the desired conformations to the binding site, simulating the actual binding scenario.

#### Virtual screening

**Fig. 5:**
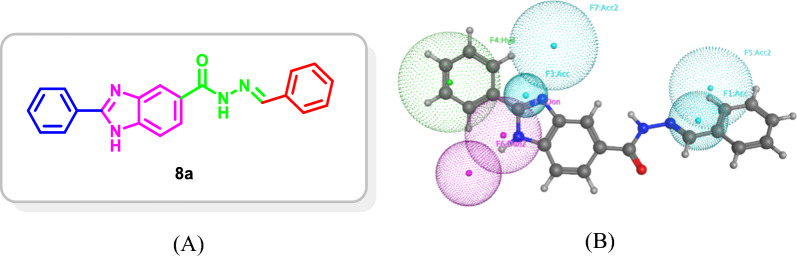
**A** The selected promising scaffold **8a** by **Ph4-4**. **B** The promising scaffold **8a** mapped onto **Ph4-4** with RMSD of 0.979Å

The pharmacophore model exhibited the best performance on the test set (Best discrimination between actives and inactive compounds), **Ph4-4**, was then used to screen an in-house dataset of diverse scaffolds. The benzimidazole scaffold **8a** was selected by **Ph4-4** as the most promising hit with the least RMSD from the assigned pharmacophore model features’ centroids (RMSD = 0.979Å) (Fig. 5).

#### Hit optimization

**Fig. 6:**
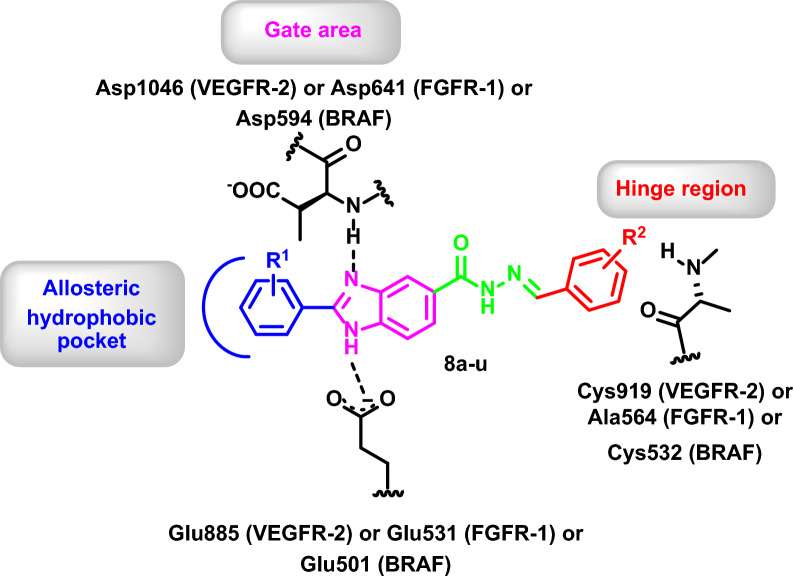
Suggested chemically feasible optimized derivatives **8a-u** from the selected promising scaffold **8a**

The promising hit scaffold was then used as a starting point to develop several optimized derivatives **8a-u** with potential chemical synthesis feasibility and probable good binding affinity which should be, by design, multi-kinase type II inhibitors for the target kinases (Fig. 6).

The design strategy took into consideration that the benzimidazole core would occupy the gate area of the target protein kinases and the imidazole moiety would be involved in hydrogen bonding with the key amino acids Glu885 and Asp1046 in VEGFR-2, Glu531 and Asp641 in FGFR-1, as well as Glu501 and Asp594 in BRAF. Thus, the 5-position of the benzimidazole moiety was substituted with different aryl groups which are decorated with hydroxy and methoxy groups at different positions to be involved in hydrogen bonding with the key amino acid Cys919 (VEGFR-2), Ala564 (FGFR-1), and Cys532 (BRAF) at the hinge region. Moreover, (un)substituted aryl groups were introduced at 2-position of the benzimidazole scaffold to occupy the allosteric hydrophobic back pocket to engage in hydrophobic interactions with the surrounding amino acid (Fig. 6).

#### Molecular docking simulation

Molecular docking is a well stablished technique for the investigation of the binding mode and binding affinity of drug-like molecules in their proposed biological targets [51–55]. In the current study, to confirm and to study the binding characteristics of the designed compounds in the binding sites of the target kinases VEGFR-2, FGFR-1 and BRAF, molecular docking studies were performed using Molecular Operating Environment (MOE, 2022.02) software. The X-ray crystallographic structures of VEGFR-2 (PDB ID: 4ASD), FGFR-1 (PDB ID: 4V01) and BRAF (PDB ID: 5CT7) in their DGF-out inactive conformation were downloaded from the Protein Data Bank (PDB) [32, 39, 49, 56]. The downloaded protein structures are co-crystalized with a type II PK inhibitor, sorafenib (**I**), ponatinib, and RAF265, respectively. Molecular docking setup was initially validated by self-docking of the co-crystalized ligands in the binding sites of their corresponding target kinases. These simulations successfully reproduced the binding pattern of the co-crystalized ligands in the target binding sites, VEGFR-2, FGFR-1 and BRAF, with energy scores of − 15.18, − 17.00 and − 15.82 kcal/mol, respectively, and with an RMSD of 0.470, 0.398 and 0.419 Å, respectively, between the docked poses and the co-crystalized ligands (For further details see Additional file 1). Additionally, the docking poses reproduced all the key interactions achieved by the co-crystallized ligands with the binding site hot spots in VEGFR-2 (Glu885, Cys919 and Asp1046), FGFR-1 (Glu531, Ala564 and Asp641), and BRAF (Glu501, Cys532 and Asp594). The validation step results indicated the suitability of the used molecular docking protocol for the molecular docking study of the target compounds **8a-u** in the binding sites of VEGFR-2, FGFR-1 and BRAF.

The docked compounds showed analogous binding patterns in the target kinases with predicted docking energy score ranges of − 14.89 to − 12.73 kcal/mol in VEGFR-2, − 14.18 to − 11.62 kcal/mol in FGFR-1, and − 13.65 to − 11.49 kcal/mol in BRAF, in comparison to the co-crystalized ligands docking score of − 15.19, − 17.00, and − 15.82 kcal/mol, respectively (See Additional file 1: Section 1: computational studies for further details).

The docked compounds showed promising binding patterns in VEGFR-2, FGFR-1 and BRAF interacting with the key amino acids in their binding pocket. The benzimidazole ring fits in the gate area stabilized via hydrogen bond interactions. By its imidazole ring, it interacts with the side chain carboxylate of Glu885, Glu531 and Glu501 of the αC helix in VEGFR-2, FGFR-1, and BRAF, respectively, and/or with Asp1046, Asp594, and Asp641 of the conserved DFG motif in VEGFR-2, FGFR-1, and BRAF, respectively.

The 2-phenyl substitution of the benzimidazole ring is directed towards the allosteric back pocket forming hydrophobic interactions with the hydrophobic side chains of the amino acids lining the pocket, Ile888, Leu889, Ile892, Val899, Leu1019, Ile1025, and Ile1044 amino acids of VEGFR-2, Ala512, Val513, Met534, Met535, Ile538, Ile545, Leu614, Leu634, Ile639, Ala640, and Phe642 of FGFR-1, and Val504, Leu505, Ile527, Leu565, Leu567, Ile572, and Ile573 of BRAF.

**Fig. 7:**
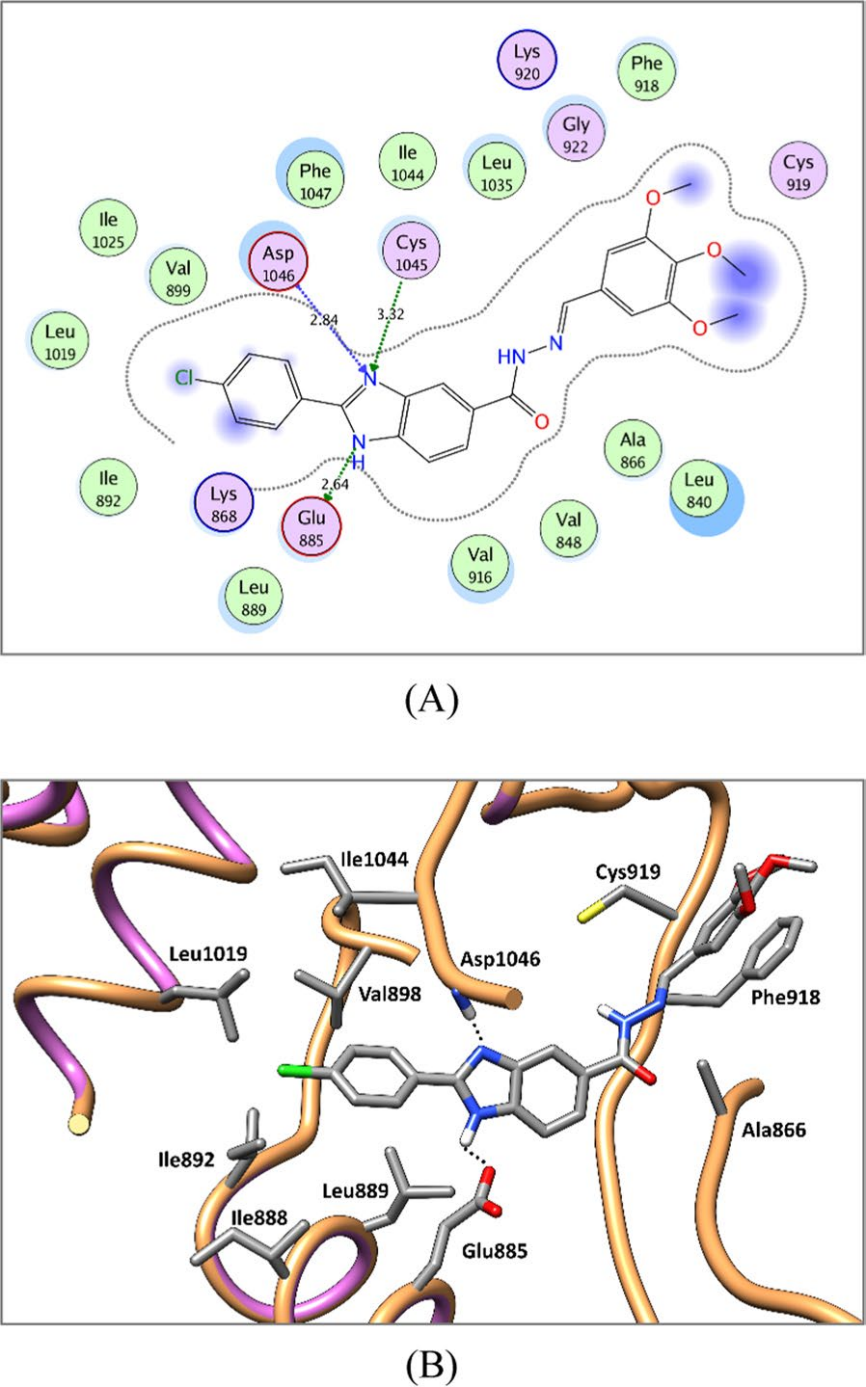
2D diagram (**A**) and 3D representation (**B**) of compound **8u** showing its interaction with the VEGFR-2 active site

**Fig. 8:**
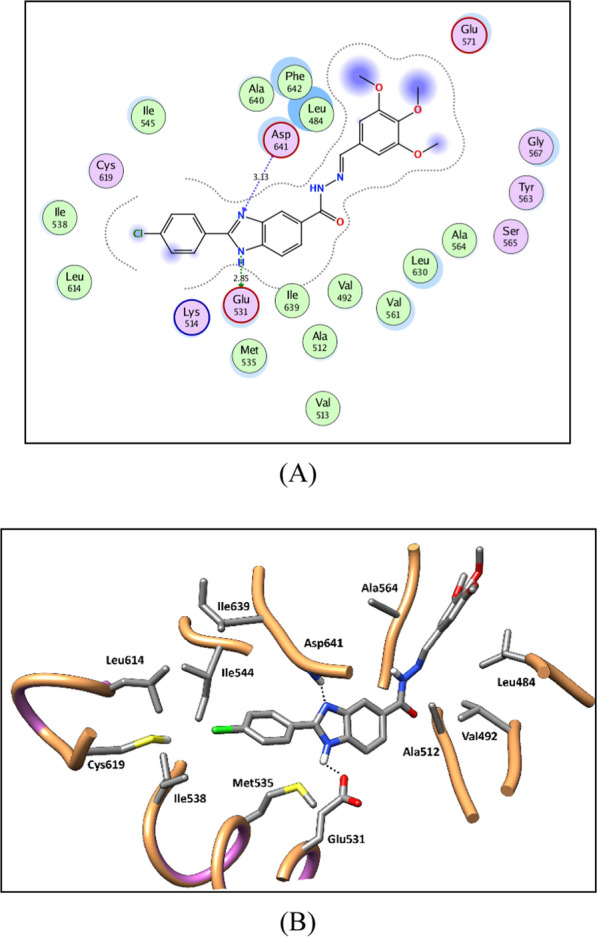
2D diagram (**A**) and 3D representation (**B**) of compound **8u** showing its interaction with the FGFR-1 active site

**Fig. 9:**
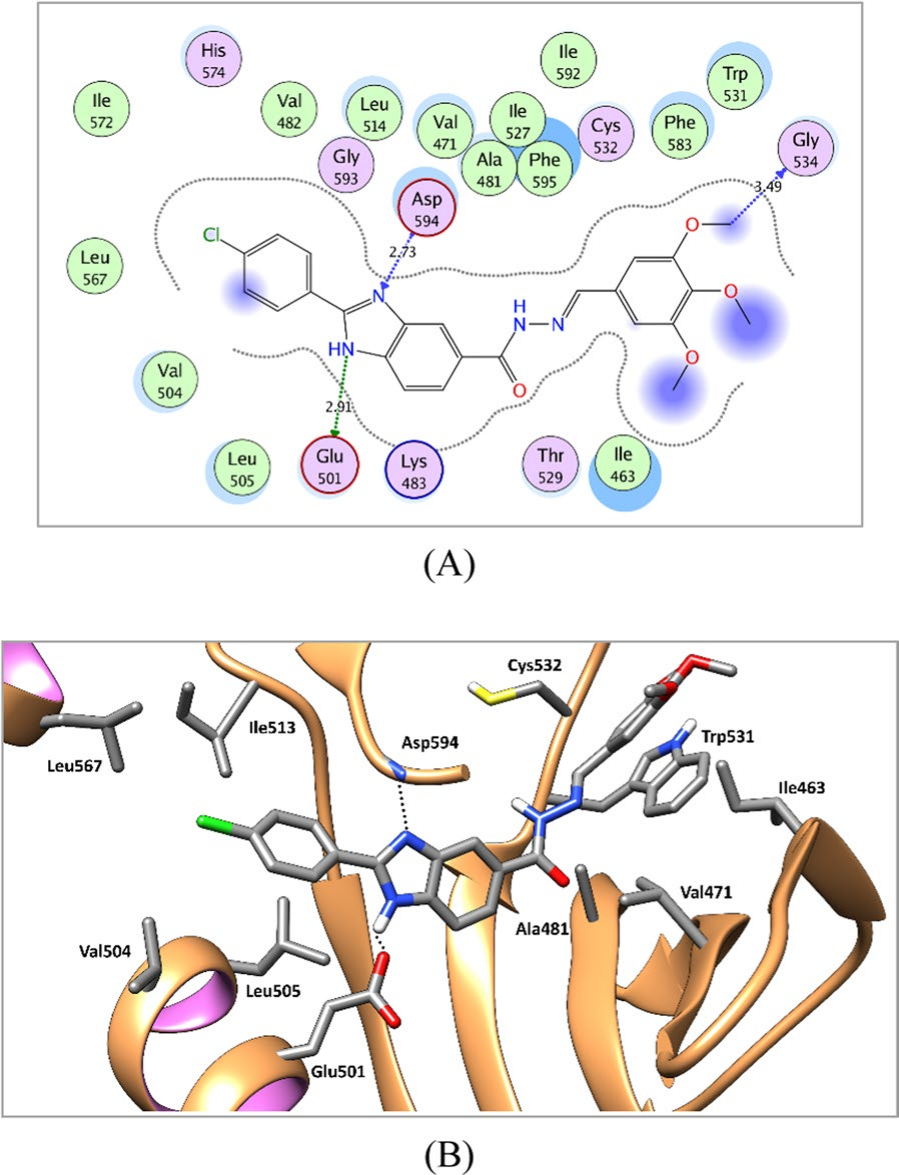
2D diagram (**A**) and 3D representation (**B**) of compound **8u** showing its interaction with the BRAF active site

The substituted benzylidene-hydrazide moiety is accommodated in the hinge region interacting in most of the target compounds through hydrogen bonding with Cys919, Ala564, and Cys532 of VEGFR-2, FGFR-1, and BRAF, respectively. Additionally, it is involved in hydrophobic interactions with the hydrophobic side chains of the amino acids in the hinge region, Leu840, Val848, Ala866, Val899, Val916, Phe918, and Leu1035 amino acids of VEGFR-2, Leu484, Val492, Ala512, Val559, Val561, Leu630, and Phe642 of FGFR-1, and Ile463, Val471, Ala481, Leu514, Trp531, Phe583, Phe595, and Leu597 of BRAF (Figs. 7, 8, 9 and see Additional file 1: Section 1: computational studies for further details).

#### ADME properties prediction

SwissADME online web tool [57–59] was used to predict the physicochemical and AMDE properties of the target compounds **8a-u**. SwissADME showed that the newly synthesized compounds possess promising predicted physiochemical and pharmacokinetic properties.

All target compounds possess promising predicted physicochemical properties and moderate predicted aqueous solubility. Moreover, they complied with Lipinski’s rule of 5 indicating that they are predicted to be orally bioavailable, and they possess a predicted SwissADME bioavailability score of 0.55 (See Additional file 1: Section 1: computational studies for further details).

**Fig. 10:**
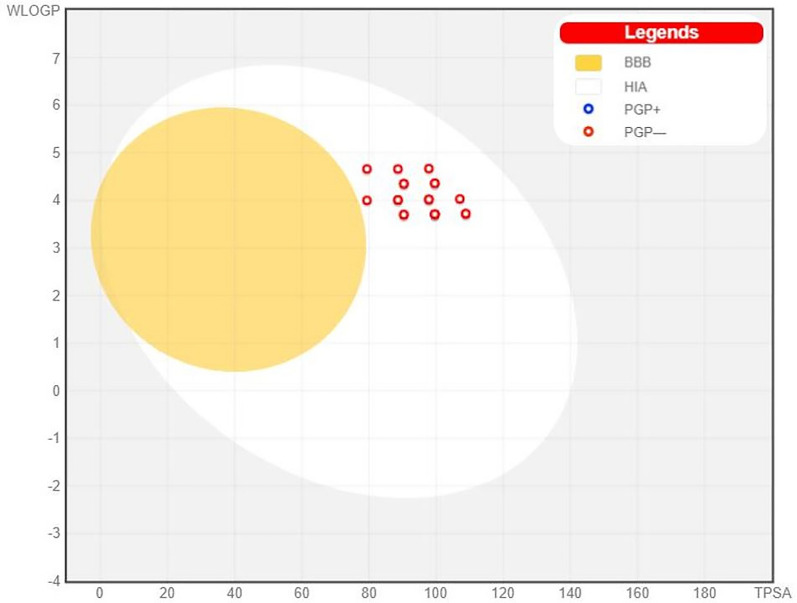
SwissADME BOILED-Egg chart for the designed compounds **8a-u**

Furthermore, as shown in SwissADME Boiled-Egg chart (Fig. 10), all target compounds showed high predicted GIT absorption with no predicted blood brain barrier (BBB) permeation and so devoid of CNS side effects. Moreover, Fig. 10 shows that all compounds are not p-glycoprotein (P-gp) substrates.

In summary, the designed benzimidazole derivatives **8a-u** are predicted to be promising type II-like multi-kinase inhibitors in terms of binding affinity and pharmacokinetic properties and can be progressed further to chemical synthesis and biological evaluation.

### Chemistry

**Fig. 11:**
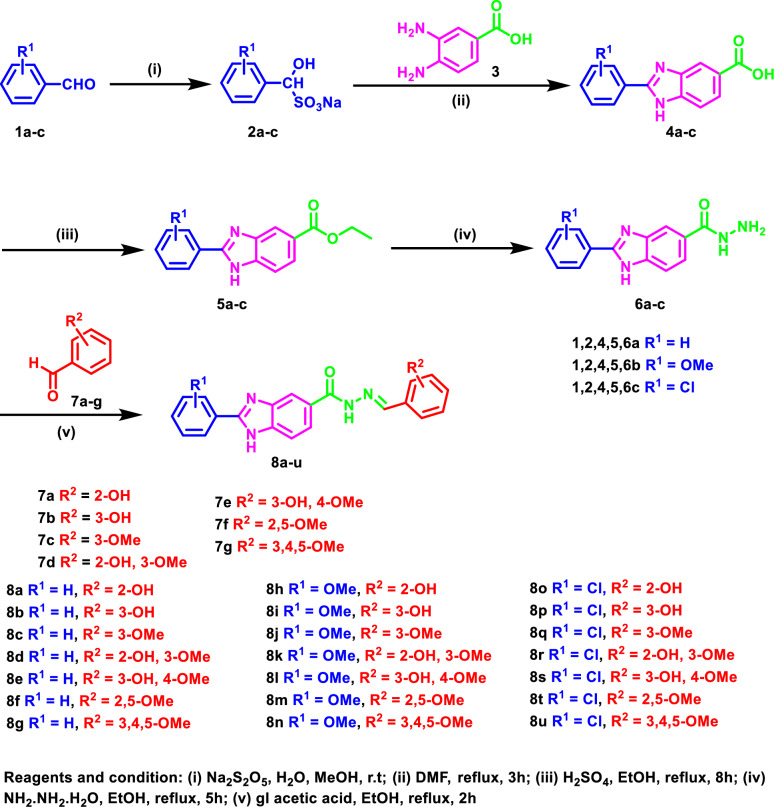
Schematic pathway for the synthesis of the target 2,5-disubstituted benzimidazoles **8a-u**

As can be seen in Fig. 11, the target 2,5-disubstituted benzimidazole derivatives** 8a-u** were synthesized by the reaction of the benzaldehyde derivatives** 1a-c** with sodium metabisulfite to give the corresponding intermediates **2a-c** which were condensed with 3,4-diaminobenzoic acid (**3**) to give the 2-aryl-benzimidzole-5-carboxylic acids **4a-c** [34]. Esterification of **4** was then carried out to afford the corresponding ethyl esters **5a-c** [60]. Hydrazinolysis of the benzimidazole esters **5a-c** was carried out to yield the benzimidazole acid hydrazides **6a-c** [61] which was followed by the reaction with different hydroxy and methoxybenzaldehyde derivatives **7a-g** to afford the target benzimidazoles **8a-u** in excellent yield.

### Biology

#### Biochemical assay

##### VEGFR-2 inhibitory activity

All the target 2,5-disubstituted benzimidazoles **8a-u** were screened for their inhibitory activity on VEGFR-2 at 10 µM concentration and the % of inhibition was depicted in Table 3 using sorafenib (**I**) as a reference standard.

**Table 3:** VEGFR-2 inhibitory activity of the synthesized 2,5-disubstituted benzimidazole derivatives **8a-u** at 10 µM in reference to sorafenib (**I**)


ID	R^1^	R^2^	% Inhibition
**8a**	H	2-OH	43.03 ± 2.32
**8b**	H	3-OH	23.82 ± 1.47
**8c**	H	3-OMe	18.52 ± 0.95
**8d**	H	2-OH, 3-OMe	53.80 ± 2.62
**8e**	H	3-OH, 4-OMe	36.36 ± 1.91
**8f**	H	2,5-OMe	58.29 ± 2.98
**8g**	H	3,4,5-OMe	47.91 ± 3.53
**8h**	OMe	2-OH	62.88 ± 3.89
**8i**	OMe	3-OH	45.16 ± 2.22
**8j**	OMe	3-OMe	15.67 ± 1.41
**8k**	OMe	2-OH, 3-OMe	69.50 ± 4.37
**8l**	OMe	3-OH, 4-OMe	50.62 ± 5.29
**8m**	OMe	2,5-OMe	6.67 ± 0.08
**8n**	OMe	3,4,5-OMe	20.81 ± 1.78
**8o**	Cl	2-OH	22.30 ± 1.95
**8p**	Cl	3-OH	22.32 ± 0.86
**8q**	Cl	3-OMe	21.49 ± 1.54
**8r**	Cl	2-OH, 3-OMe	25.71 ± 1.87
**8s**	Cl	3-OH, 4-OMe	26.72 ± 1.83
**8t**	Cl	2,5-OMe	33.99 ± 2.57
**8u**	Cl	3,4,5-OMe	80.0 ± 3.98
**Sorafenib (I)**	–	–	99 ± 0.50

Mean % inhibition (duplicate test) at a single dose (10 μM). Data are represented as mean value ± SD

The designed and synthesized 2,5-disubstituted benzimidazole **8a-u** inhibited VEGFR-2 to variable extent. In series **8a-g**, the 2,5-disubstituted benzimidazoles **8a**, **8d**, **8f** and **8g** displayed moderate inhibition of VEGFR-2 at 10 µM with inhibition % range of 43.03% to 58.29%. The substitution on the phenyl group at 5-position of the benzimidazole greatly affects the inhibition %. Compound **8a** exhibiting 2-hydroxyphenyl moiety showed inhibition % of 43.03%, whereas further introduction of a methoxy group at 3-position to yield compound **8d** resulted in increasing the potency (inhibition % = 53.80%). On the contrary, the 3-hydroxyphenyl **8b**, 3-methoxyphenyl **8c**, 3-hydroxy,4-methoxyphenyl **8e** derivatives displayed weak potency against VEGFR-2 with inhibition % of 23.82%, 18.52% and 36.36%, respectively. Compound **8f** exhibiting 2,5-dimethoxyphenyl group showed the most promising inhibition % of 58.29%, while introduction of 3,4,5-trimethoxyphenyl moiety in **8g** resulted in a slight decrease in the potency (inhibition % = 47.91%).

In series **8h-n**, compounds **8h** and **8k** displayed potent VEGFR-2 inhibition % of 62.88% and 69.50%, respectively. Meanwhile, their regioisomers **8i** and **8l**, respectively, demonstrated a decrease in the potency (inhibition % of 45.16% and 50.62%, respectively). Compounds **8j**, **8m**, and **8n** with 3-methoxyphenyl, 2,5-dimethoxyphenyl and 3,4,5-trimethoxyphenyl groups, respectively, displayed weak VEGFR-2 inhibitory activity with inhibition % of 15.67%, 6.67%, and 20.81%, respectively.

In series **8o-u**, which exhibit 4-chlorophenyl group at the 2 position, compound **8u** incorporating 3,4,5-trimethoxyphenyl group displayed a promising VEGFR-2 inhibitory activity with an inhibition % of 80%, whereas replacement of the 3,4,5-trimethoxyphenyl moiety with hydroxy or methoxyphenyl groups at different positions resulted in a weak inhibitory activity on VEGFR-2 with (inhibition % range of 21.49—33.99%) in compounds **8o-t**.

##### Multi-kinase inhibitory activity of **8u**

In reference to the potent activity of **8u** on VEGFR-2 (Table 3), it was further investigated for its inhibitory activity on VEGFR-2, FGFR-1 and BRAF at different concentrations and its IC_50_ values are presented in Table 4.

**Table 4:** IC_50_ (µM) values of compound **8u** on VEGFR-2, FGFR-1 and BRAF in reference to sorafenib (**I**)


		IC_50_ (µM)		
		VEGFR-2	FGFR-1	BRAF
**8u**		0.93 ± 0.10	3.74 ± 0.34	0.25 ± 0.03
Sorafenib		0.10 ± 0.01	0.58 ± 0.10 [62]	0.02 ± 0.002

The disubstituted benzimidazole derivative **8u** showed an interesting potent multi-kinase inhibitory activity on BRAF (IC_50_ = 0.25 µM), followed by VEGFR-2 (IC_50_ = 0.93 µM) and FGFR-1 (IC_50_ = 3.74 µM) in reference to sorafenib (**I**) which demonstrated IC_50_ = 0.02, 0.10 and 0.58 µM against BRAF, VEGFR-2 and FGFR-1, respectively.

#### Antiproliferative activity

##### Antiproliferative activity on NCI cancer cell lines at 10 µM concentration

All the target 2,5-disubstituted benzimidazoles **8a-u** were screened by NCI (USA) for their ability to supress the growth of NCI 60 cancer cell lines at 10 micromolar concentration and the results were depicted in Table 5.

**Table 5:** In vitro growth inhibition % (GI %) of target 2,5-diarylbenzimidazole **8a-u** against NCI panel of 60 tumor cell lines at 10 µM concentration

Cell name	GI%																				
	**8a**	**8b**	**8c**	**8d**	**8e**	**8f**	**8g**	**8h**	**8i**	**8j**	**8k**	**8l**	**8m**	**8n**	**8o**	**8p**	**8q**	**8r**	**8s**	**8t**	**8u**
*Leukemia*																					
CCRF-CEM	88.16	54.37	44.52	L^a^	35.93	80.48	61.96	94.27	80.82	35.00	L	89.42	45.90	38.76	L	71.92	46.97	L	L	56.12	L
HL-60(TB)	95.58	14.38	38.07	90.03	24.68	36.28	17.05	82.91	55.15	30.37	L	52.20	18.79	–	47.15	–	50.70	L	45.32	12.89	67.85
K-562	78.58	21.43	41.67	60.91	26.24	63.00	30.39	99.48	60.56	88.06	L	48.52	22.75	23.67	85.46	49.86	98.12	L	50.30	52.90	L
MOLT-4	89.54	31.49	35.43	65.79	36.14	49.49	21.20	88.52	78.97	59.41	L	78.57	35.76	22.13	60.79	68.62	71.63	L	83.69	58.90	96.04
RPMI-8226	88.59	9.59	81.63	98.47	23.38	81.35	86.93	96.71	24.77	L	L	32.41	76.82	44.49	88.15	56.71	L	L	L	62.25	L
SR	92.31	36.75	36.30	77.01	44.12	36.30	17.59	85.9	81.89	92.65	L	72.20	28.49	26.93	75.20	26.42	L	L	77.15	31.36	L
*Non-small cell lung cancer*																					
A549/ATCC	L	–^b^	20.15	79.76	–	17.21	29.27	L	11.15	L	76.72	5.94	13.69	–	86.99	7.53	L	L	7.66	8.76	70.84
EKVX	96.49	13.81	41.83	87.59	19.18	22.69	12.24	92.19	23.40	L	98.06	12.95	10.58	–	67.24	7.59	L	L	37.02	42.46	90.75
HOP-62	L	19.26	15.41	nd^c^	21.42	nd	nd	L	41.98	L	L	55.53	19.45	14.62	nd^c^	32.45	L	L	nd	18.43	L
HOP-92	L	–	–	68.37	6.30	7.10	–	L	27.01	L	70.36	17.43	–	–	L	6.79	L	L	–	36.64	L
NCI-H226	93.32	–	19.33	L	11.44	49.54	50.35	L	7.75	98.27	82.17	24.51	–	22.33	88.60	30.08	L	L	37.63	35.69	L
NCI-H23	83.12	–	9.68	L	–	46.67	49.43	94.63	6.56	L	L	23.97	–	10.75	L	56.65	L	L	29.22	33.93	L
NCI-H322M	94.14	–	6.09	88.01	–	12.31	15.67	96.71	18.26	L	95.89	24.04	–	–	70.15	–	L	L	18.15	28.86	96.14
NCI-H460	L	–	13.08	86.38	–	15.82	56.31	L	5.05	L	L	14.98	–	–	L	56.63	L	L	63.79	17.46	L
NCI-H522	L	38.06	25.96	L	29.39	37.77	50.69	L	43.41	89.70	L	38.23	21.28	96.2	L	55.77	L	L	30.91	40.86	L
*Colon cancer*																					
COLO 205	L	–	32.62	nd	35.42	nd	nd	L	32.41	L	L	76.84	–	33.71	nd	34.57	L	L	nd	18.27	L
HCC-2998	73.20	–	–	98.55	–	8.92	44.26	99.64	14.86	88.46	L	15.01	–	–	77.99	15.94	79.58	L	86.84	14.58	L
HCT-116	L	–	12.67	97.43	15.03	52.52	41.62	L	42.79	L	L	43.83	22.29	23.4	98.39	37.67	L	L	69.55	43.08	L
HCT-15	94.00	–	27.47	71.57	–	65.20	38.39	L	5.17	79.23	78.11	–	13.68	–	L	–	63.00	95.20	23.27	58.25	44.27
HT29	L	16.35	33.92	84.54	95.82	14.69	47.26	L	74.50	L	L	L	25.35	nd	L	19.88	L	L	L	20.28	L
KM12	84.00	19.80	25.98	L	21.86	30.49	36.44	L	38.72	L	L	34.46	11.66	13.78	85.44	62.74	L	L	68.41	23.33	L
SW-620	77.39	–	–	70.84	–	30.32	37.93	L	6.72	L	98.83	21.77	–	–	L	6.31	L	L	69.62	7.98	L
*CNS cancer*																					
SF-268	L	16.82	15.05	99.03	16.00	36.62	31.99	L	37.35	84.67	L	40.44	9.02	21	L	24.23	L	L	72.71	43.02	L
SF-295	L	–	18.50	83.16	10.86	31.38	54.12	L	21.96	167.36	96.39	50.95	19.91	29.8	L	11.79	L	L	84.96	51.90	L
SF-539	L	13.68	28.61	L	32.21	36.88	49.79	L	53.27	34.15	L	42.58	32.57	24.35	L	32.42	L	L	47.21	45.33	L
SNB-19	95.02	–	–	90.36	–	21.40	25.17	L	15.85	L	L	16.06	–	36.2	L	nd	L	L	39.72	45.69	L
SNB-75	L	6.56	11.00	99.21	–	–	–	95.58	17.83	L	90.95	25.75	–	–	L	7.89	L	L	6.41	27.17	L
U251	L	–	14.27	99.86	14.62	20.71	42.32	L	32.10	L	97.28	44.79	9.71	15.28	L	75.53	L	L	75.23	25.71	L
*Melanoma*																					
LOX IMVI	88.38	7.43	–	92.90	–	62.46	56.54	98.7	26.07	L	L	37.90	18.79	7.53	L	14.74	L	L	44.94	34.64	L
MALME-3 M	99.32	39.71	47.13	L	78.42	40.49	73.63	L	L	L	L	87.59	18.33	76.79	L	58.88	L	L	L	18.37	L
M14	81.80	–	8.28	L	19.74	34.40	43.27	L	39.14	70.41	90.97	63.49	–	40.28	90.88	25.37	L	L	42.14	30.86	L
MDA-MB-435	63.10	5.45	18.89	74.61	30.39	73.65	48.54	L	63.84	76.54	76.03	26.54	–	24.97	L	15.16	98.89	L	89.90	14.33	L
SK-MEL-2	60.21	–	19.50	34.31	8.51	16.71	21.79	nd	37.55	80.99	39.06	25.77	11.11	nd	L	23.97	L	L	76.06	–	L
SK-MEL-28	82.67	–	20.43	96.19	28.87	29.06	40.41	L	52.90	64.23	L	52.45	5.70	31.45	78.18	15.97	L	L	70.75	14.62	L
SK-MEL-5	85.73	10.91	39.03	L	39.52	67.75	89.35	L	40.51	L	L	55.34	22.29	42.62	L	69.47	L	L	53.32	68.78	L
UACC-257	88.80	–	14.61	L	13.76	–	54.30	85.05	47.38	57.67	L	52.86	14.32	25.57	42.37	–	L	L	24.65	–	L
UACC-62	75.88	–	14.66	99.46	18.69	35.47	70.39	L	50.84	58.87	98.01	47.57	6.39	68.48	95.30	19.57	L	L	37.51	24.28	L
*Ovarian cancer*																					
IGROV1	82.83	12.96	10.09	79.92	21.96	10.98	16.96	L	11.20	L	84.81	20.28	–	–	84.28	5.71	L	L	–	7.82	92.38
OVCAR-3	L	11.09	7.38	L	7.25	12.52	29.45	L	8.04	L	L	37.61	–	–	L	–	L	L	47.55	10.78	L
OVCAR-4	94.42	12.43	20.07	L	–	37.84	21.95	L	27.06	L	86.01	9.88	8.31	–	L	15.65	L	L	20.81	38.89	L
OVCAR-5	77.04	–	–	63.24	–	–	–	L	–	91.17	69.48	–	–	–	55.93	–	L	L	–	–	37.36
OVCAR-8	L	10.32	24.52	L	25.20	52.57	48.68	L	38.12	L	L	28.08	23.61	15.01	L	19.01	L	L	43.17	17.17	L
NCI/ADR-RES	63.15	–	6.59	47.95	–	24.51	–	63.2	–	31.99	28.06	–	–	–	56.35	–	23.04	49.30	–	11.23	24.32
SK-OV-3	L	25.63	63.95	nd	56.26	nd	nd	L	53.95	68.31	L	–	–	20.23	nd	36.21	L	L	nd	15.10	L
*Renal cancer*																					
786–0	91.76	5.43	21.32	86.04	16.58	18.59	27.80	L	23.41	L	L	18.10	29.04	–	L	22.17	L	L	52.04	26.55	L
A498	–	–	–	–	–	–	–	54.5	–	–	–	–	–	–	53.91	10.17	L	L	–	56.16	L
ACHN	L	–	–	83.27	–	36.51	33.83	L	–	L	87.70	–	8.59	9.17	94.16	–	L	L	–	53.09	69.94
CAKI-1	L	9.41	10.00	91.59	17.64	41.47	49.73	L	5.16	85.29	84.62	–	–	23.76	L	–	L	L	18.49	38.36	97.13
RXF 393	L	–	29.22	85.95	–	49.96	30.81	L	14.49	L	L	37.22	15.21	20.09	L	31.57	L	L	69.12	96.54	L
SN12C	nd	nd	nd	91.53	nd	35.21	32.21	L	nd	nd	nd	26.96	nd	9.66	59.42	nd	nd	nd	31.33	nd	nd
TK-10	65.04	–	–	50.64	–	18.99	6.66	L	–	L	40.00	–	–	–	95.02	–	L	L	–	10.93	62.64
UO-31	86.22	21.59	30.63	98.53	38.19	54.04	45.55	L	10.49	98.91	83.20	9.13	9.12	21.84	L	14.89	81.69	L	8.70	31.95	41.11
*Prostate cancer*																					
PC-3	90.74	11.75	24.64	82.18	11.47	55.54	37.87	L	34.39	L	87.92	51.22	13.61	12.94	78.51	48.79	L	L	56.49	58.53	L
DU-145	L	–	18.43	79.68	–	18.43	17.07	L	13.98	L	85.12	27.88	–	–	L	–	L	L	16.20	51.99	L
*Breast cancer*																					
MCF7	96.94	43.15	50.96	98.08	70.45	54.14	85.99	L	88.20	L	L	77.80	20.64	73.09	93.74	72.33	L	L	84.87	48.06	L
MDA-MB-231/ATCC	88.16	14.06	–	94.36	–	38.00	30.48	98.72	22.38	L	97.65	26.37	14.33	7.37	L	45.17	L	L	75.12	89.71	L
HS 578 T	L	–	19.99	92.89	6.89	49.91	51.04	L	52.61	L	89.32	46.23	–	37.73	L	27.07	L	L	86.87	48.77	L
BT-549	L	–	–	96.59	–	38.19	35.43	L	32.71	29.81	L	10.40	17.16	–	60.58	–	6.97	L	42.90	25.73	L
T-47D	93.65	50.90	69.11	nd	39.30	nd	nd	L	86.79	L	94.97	52.49	40.60	38.08	nd	79.76	L	L	nd	61.77	L
MDA-MB-468	99.81	18.93	22.71	L	7.65	24.73	39.16	L	54.60	L	L	44.35	16.21	–	L	65.77	L	L	88.69	30.39	L
Mean growth inhibition %	97.73	8.49	20.74	92.51	16.75	33.74	36	L	33.08	L	L	34.39	10.90	13.64	L	25.43	L	L	49.49	33.49	L

^a^GI% > 100; ^b^GI% < 5%; ^c^not detected

**Fig. 12:**
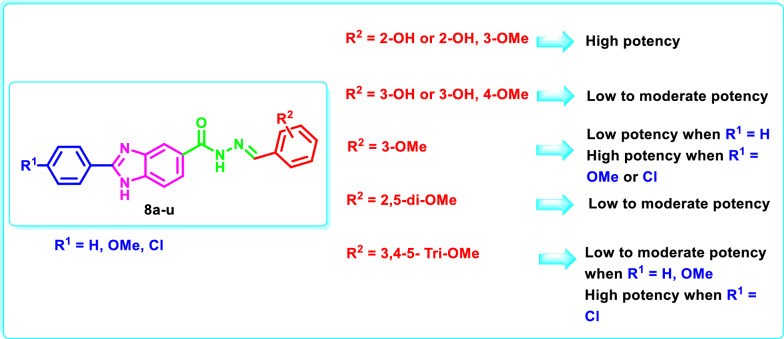
Structure activity relationship diagram showing the effect of the substitution pattern of 2- and 5-phenyl moieties of **8a-u** on the antiproliferative activity

The substitution pattern on the 2 and 5 phenyl moieties has a great influence on the growth inhibitory activity of the synthesized benzimidazoles **8a-u** on cancer cell lines (Table 5, Fig. 12). In the 2-phenylbenzimidazole series **8a-g**, incorporation of 2-hydroxyphenyl and 2-hydroxy, 3-methoxyphenyl moieties at the 5-position in **8a** and **8d**, respectively, resulted in a potent mean growth inhibition % (GI%) of 97.73% and 92.51%, respectively, with a broad spectrum antiproliferative activity against the different NCI sub-panels. Isomeric shifting of the hydroxy group to the 3-position in **8b** and **8e**, respectively, decreased the mean GI% to 8.49% and 16.75%, respectively. On the other side, the 3-methoxyphenyl **8c**, 2,5-dimethoxyphenyl **8f** and 3,4,5-trimethoxyphenyl **8g** derivatives showed moderate mean GI% of 20.74%, 33.74% and 36.00%, respectively.

In series **8h-n** which incorporates 2-(4-methoxyphenyl)benzimidazole moiety, the 2-hydroxyphenyl derivatives **8h** and **8k** showed mean GI% > 100% (lethal effect) on the tested cell lines. On the contrary, the 3-hydroxyphenyl congeners **8i** and **8l** demonstrated moderate mean GI% of 33.08 and 34.39%, respectively. Replacement of 3-hydroxyphenyl group in **8i** with 3-methoxyphenyl moiety in **8j** increased the inhibitory activity (mean GI% of 33.08% *versus* 100% (lethal effect), respectively). However, the 2,5-dimethoxyphenyl derivative **8m** and the 3,4,5-trimethoxyphenyl derivative **8n** demonstrated weak mean GI% of 10.90 and 13.64%, respectively.

Replacement of the 2-(4-methoxyphenyl)benzimidazole moiety in series **8h-n** with 2-(4-chlorophenyl)benzimidazole moiety in series **8o-u**, showed the same pattern of mean GI%. The benzimidazole derivative incorporating 2-hydroxyphenyl, 3-methoxyphenyl and 2-hydroxy, 3-methoxyphenyl **8o**, **8q** and **8r**, respectively showed a mean GI% > 100% (lethal effect). On the other hand, the derivatives **8p**, **8s** and **8t** demonstrated moderate mean GI% of 25.43, 49.49, and 33.49%, respectively. Furthermore, the 5-(3,4,5-trimethoxyphenyl)benzimidazole derivative **8u** showed a mean GI% > 100% (lethal effect) on the tested cell lines.

##### Antiproliferative activity on NCI cancer cell lines at five different concentrations

The 2,5-diaryl benzimidazole derivatives **8a**, **8d**, **8h**, **8j**, **8k**, **8o**, **8q**, **8r**, and **8u** were selected by NCI to be further assayed for their growth inhibitory activity at five dose level and their GI_50_ results were depicted in Table 6. The selected 2,5-diaryl benzimidazoles displayed potent inhibitory activity against the tested cell lines with GI_50_ up to 0.12 µM. Close examination showed that **8d** displayed the most potent GI_50_ against most of the cell lines with mean GI_50_ of 2.62 µM. Meanwhile compounds **8a**, **8h**, **8j**, **8k**, **8o**, **8q**, **8r** and **8u** demonstrated potent inhibitory activity against most of the tested cell lines with mean GI_50_ range of 2.98 to 7.98 µM.

**Table 6:** GI_50_ of compounds **8a**, **8d**, **8h**, **8j**, **8k**, **8o**, **8q**, **8r**, and **8u** on NCI cancer cell lines

Cell name	GI_50_ (µM)								
	**8a**	**8d**	**8h**	**8j**	**8k**	**8o**	**8q**	**8r**	**8u**
*Leukemia*									
CCRF-CEM	0.26	0.30	0.92	7.91	1.05	> 38.8	1.95	0.43	6.27
HL-60(TB)	3.08	2.42	2.09	36.5	7.66	0.31	19.5	1.95	3.90
K-562	3.39	2.04	1.36	6.92	3.74	0.15	3.14	0.49	1.96
MOLT-4	1.62	2.06	1.70	22.6	3.80	0.28	5.96	0.63	3.58
RPMI-8226	1.19	1.86	1.39	4.14	2.80	> 38.8	1.47	2.22	3.21
SR	1.68	1.80	1.17	10.6	2.06	0.12	4.15	0.50	3.76
*Non-small cell lung cancer*									
A549/ATCC	3.41	3.39	3.03	3.05	9.04	1.80	3.22	3.68	7.55
EKVX	1.85	1.86	3.24	2.76	2.30	9.31	3.52	4.56	14.80
HOP-62	1.47	1.65	5.09	2.70	3.11	0.84	1.92	1.04	4.68
HOP-92	nd^a^	1.58	2.83	nd	nd	> 38.8	nd	1.25	5.95
NCI-H226	4.02	0.53	6.94	2.13	3.14	4.33	2.57	1.65	1.31
NCI-H23	3.31	1.87	2.06	2.95	2.14	0.31	2.20	1.15	4.72
NCI-H322M	2.13	1.41	4.23	3.03	3.04	1.38	3.57	1.59	8.29
NCI-H460	2.37	2.63	1.87	1.85	3.27	0.25	3.34	1.35	3.19
NCI-H522	1.87	1.97	1.54	3.30	2.98	0.26	2.26	1.17	4.90
*Colon cancer*									
COLO 205	1.97	2.34	2.90	4.30	3.94	0.89	4.02	2.33	2.84
HCC-2998	3.91	1.79	4.43	3.52	2.33	1.38	4.42	1.48	11.10
HCT-116	0.88	1.84	4.93	2.99	2.90	1.08	3.06	1.19	3.26
HCT-15	1.07	2.07	1.25	3.92	3.35	0.20	5.52	2.72	73.0
HT29	2.63	3.41	nd	2.80	3.67	1.08	2.38	4.17	2.98
KM12	3.72	2.58	4.24	4.34	2.45	1.24	4.42	1.66	4.50
SW-620	3.48	3.77	1.75	3.10	4.35	0.33	2.93	1.32	4.40
*CNS cancer*									
SF-268	2.78	1.76	0.92	3.28	2.16	0.84	2.18	1.21	4.40
SF-295	2.70	2.93	1.26	2.93	2.48	1.60	2.17	2.20	4.18
SF-539	2.74	1.31	2.05	11.5	2.80	0.37	7.14	1.07	2.02
SNB-19	4.16	2.41	1.76	3.18	3.33	1.12	2.17	1.31	3.42
SNB-75	2.20	nd	0.39	1.78	3.05	0.74	5.69	nd	1.28
U251	2.11	0.76	1.64	2.18	2.17	0.83	2.03	1.50	2.59
*Melanoma*									
LOX IMVI	2.00	1.34	2.23	1.98	2.34	0.43	1.82	1.18	4.67
MALME-3 M	1.88	1.37	1.77	2.45	1.09	1.03	1.54	1.36	1.12
M14	0.92	1.57	3.65	3.42	2.81	1.81	2.15	2.07	3.77
MDA-MB-435	5.77	4.69	1.95	6.25	6.20	0.53	3.90	1.81	4.13
SK-MEL-2	3.19	1.64	5.17	10.6	6.30	4.60	3.07	nd	11.0
SK-MEL-28	4.62	1.78	2.96	3.93	3.32	1.85	2.15	1.62	1.83
SK-MEL-5	3.75	1.40	1.73	3.45	2.86	0.51	4.90	1.34	2.83
UACC-257	4.00	2.34	6.32	11.0	3.43	7.35	4.80	2.35	4.67
UACC-62	3.66	1.22	2.53	6.50	2.78	1.69	4.92	1.23	1.24
*Ovarian cancer*									
IGROV1	3.90	2.34	2.26	3.49	3.52	0.95	3.01	1.25	11.0
OVCAR-3	1.94	0.42	1.98	2.05	1.25	0.19	3.24	0.56	2.28
OVCAR-4	2.24	1.85	2.24	2.01	3.26	1.25	3.07	1.41	2.35
OVCAR-5	8.50	2.77	6.55	4.69	7.72	2.12	5.35	2.17	15.6
OVCAR-8	2.49	1.62	1.97	1.92	2.71	0.98	2.69	1.58	3.87
NCI/ADR-RES	3.51	4.65	6.74	6.35	> 100	1.15	94.8	> 100	nd
SK-OV-3	2.62	2.56	7.30	5.39	4.31	4.53	2.02	2.08	15.0
*Renal cancer*									
786–0	3.84	2.47	nd	3.84	3.69	0.83	1.85	1.49	4.88
A498	16.90	36.6	9.21	21.5	> 100	8.10	15.3	12.1	26.4
ACHN	3.28	2.28	3.02	4.25	4.19	0.81	4.21	2.92	7.93
CAKI-1	3.48	2.68	1.78	2.81	3.64	0.22	3.84	2.31	11.2
RXF 393	2.37	3.21	6.29	2.26	2.58	0.79	2.06	1.41	2.22
SN12C	4.67	2.08	4.99	2.78	3.81	3.41	2.22	1.72	3.22
TK-10	4.53	4.78	6.19	12.6	> 100	5.08	23.9	6.46	28.8
UO-31	0.60	2.34	1.70	1.78	3.34	0.31	3.46	2.07	> 100
*Prostate cancer*									
PC-3	nd	2.71	1.77	nd	nd	> 38.8	nd	1.62	5.73
DU-145	2.75	3.05	2.33	3.28	5.06	1.36	2.04	1.99	6.77
*Breast cancer*									
MCF7	2.63	1.06	1.67	2.50	1.72	0.16	2.57	0.66	0.66
MDA-MB-231/ATCC	2.03	2.41	4.48	2.43	2.80	1.41	1.89	1.82	3.39
HS 578 T	2.49	0.62	0.94	1.90	2.22	0.26	2.71	0.70	2.58
BT-549	1.80	1.52	6.99	5.47	2.85	3.57	10.1	1.36	2.69
T-47D	3.38	1.93	2.60	2.62	3.48	1.48	1.95	0.60	3.48
MDA-MB-468	3.45	0.89	2.98	3.06	1.95	1.51	2.97	1.44	3.27
Mean GI_50_	3.00	2.62	2.98	5.24	7.70	5.44	5.40	3.39	7.98

^a^Not detected

#### Cell cycle analysis

**Fig. 13:**
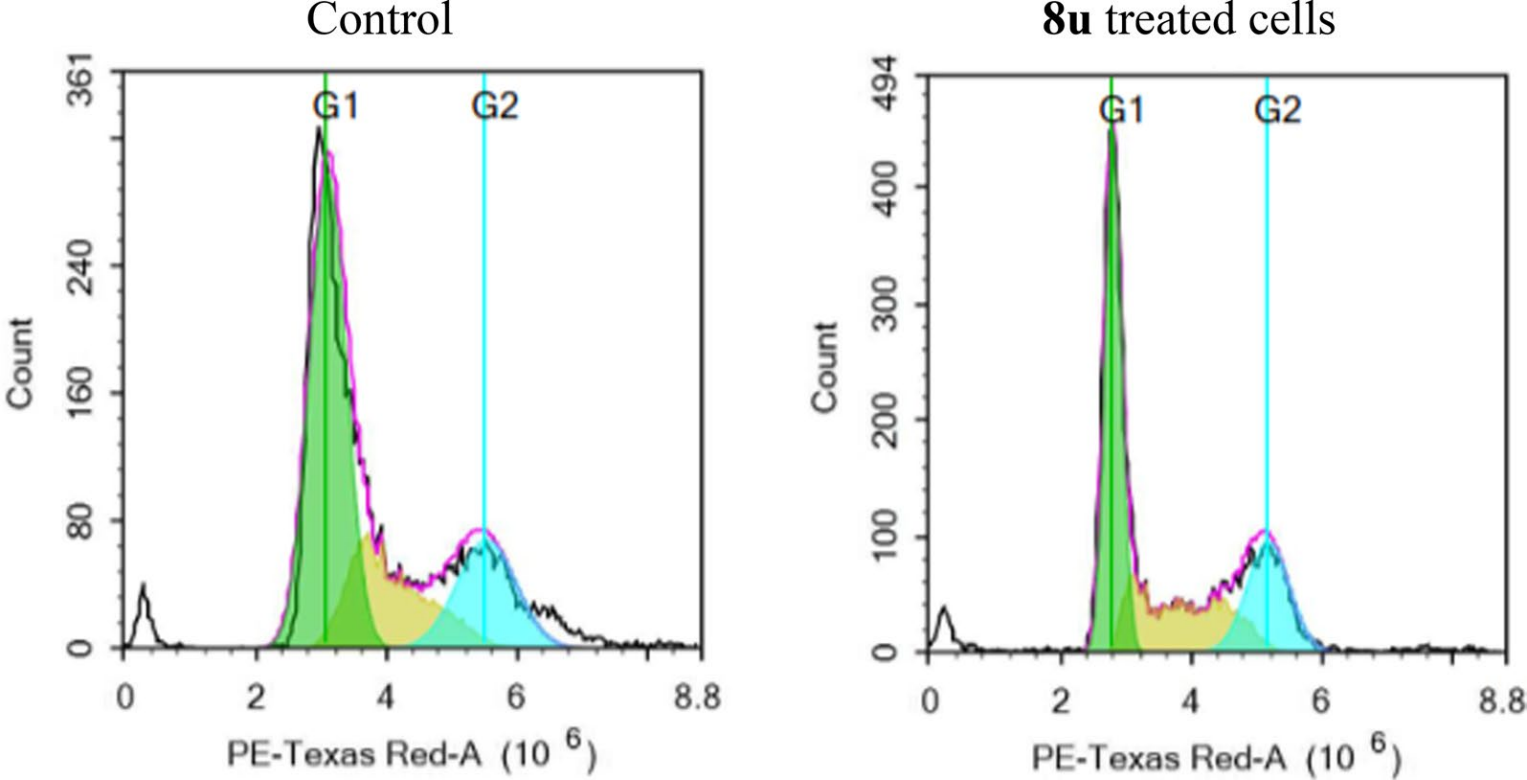
Effect of compound **8u** on the phases of cell cycle of MCF-7 cells

Encouraged by the potent multi-kinase inhibitory activity of the 2,5-diaryl benzimidazole derivative **8u** as well as its potent and broad spectrum of antiproliferative activity, it was selected as a representative to be examined for its influence on the cell cycle progression of MCF-7 cell line at its GI_50_ concentration by flow cytometry analysis using propidium iodide (PI) stain. Comparison with breast cancer MCF-7 cells treated with DMSO as control was carried out, and the results were presented in Fig. 13 and Table 7. The % of MCF-7 cells that was accumulated in the G1 phase showed a decrease from 56.30% to 45.32% after treatment with **8u**. On the other side, the accumulated % of cells in the G2/M phase increased from 20.60% to 25.57% (Table 7). These results emphasized that **8u** arrests the MCF-7 cell line at the G2/M phase. Furthermore, an increase in the percent of cells accumulated in the sub-G1 phase, from 2.64% in the control to 4.40% in the treated cells was noticed as a result of cell apoptosis.

**Table 7:** Effect of compounds **8u** on the phases of cell cycle of MCF-7 cells

Comp.	%G0/G1	%S	%G2/M	%Sub-G1
Control	56.30	23.10	20.60	2.64
**8u**	45.32	29.11	25.57	4.40

#### Apoptosis assay

**Fig. 14:**
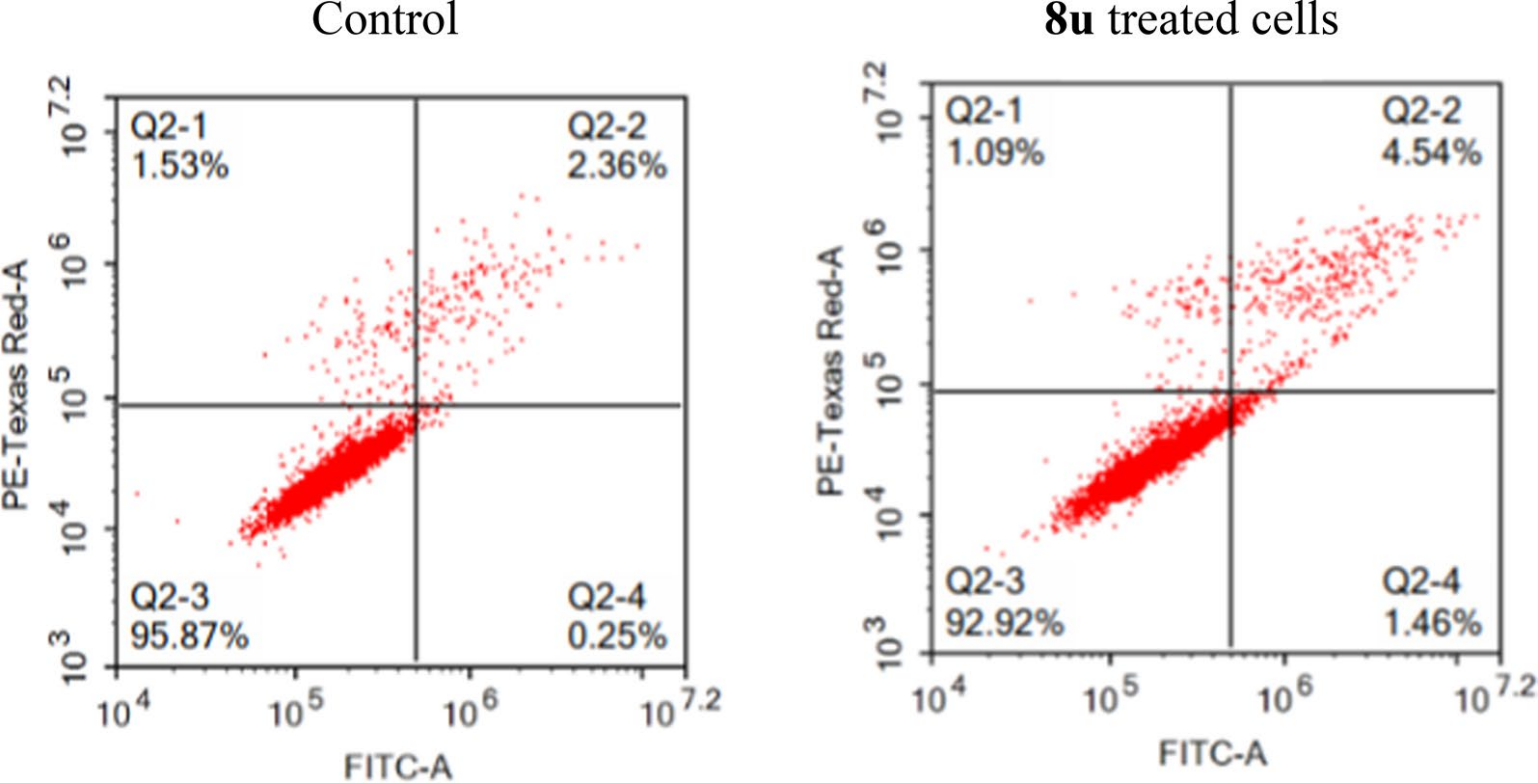
Effect of **8u** on the percentage of annexin V-FITC-positive staining in MCF-7 cells. The four quadrants identified as: **Q2-3**, viable; **Q2-4**, early apoptotic; **Q2-2**, late apoptotic; **Q2-1**, necrotic

The capability of **8u** to enhance apoptosis of MCF-7 cell line at its GI_50_ concentration was explored (Fig. 14). The presented results showed that the % of cells in the early apoptosis and late apoptosis phase increased from 0.25% and 2.36% to 1.46 and 4.54%, respectively, after treatment with **8u,** which indicates that **8u** induces cell apoptosis in MCF-7 cell line.

## Conclusion

Several receptor-based pharmacophore models describing the binding features required for the multi-kinase inhibition of the target kinases (VEGFR-2, FGFR-1, and BRAF) were constructed based on the experimental binding mode and binding interactions of several inhibitors for these target kinases. Using a compiled test set of 73 active inhibitors for the target kinases as well as 2314 inactive decoys, (**Ph4-4**) was selected as the best model showing F1 score of 0.5023 and Mathew’s correlation coefficient of 0.5131 indicating its good overall quality in discriminating between the active and the inactive compounds. Virtual screening of an in-house dataset of diverse scaffolds using the selected pharmacophore model yielded a benzimidazole-based scaffold as a promising hit among the dataset compounds with RMSD of 0.979Å. Structural optimization of the hit benzimidazole-based scaffold through (un)substituted aryl substitution on 2 and 5 positions of the benzimidazole ring produced compounds **8a-u**. Based on molecular docking simulations and ADME properties predictions, the optimization products were predicted to be promising type II-like multi-kinase inhibitors in terms of binding affinity and pharmacokinetic properties and can be progressed further to chemical synthesis and biological evaluation.

The designed compounds **8a-u** were synthesized, and they were tested for their VEGFR-2 inhibitory activity at 10 µM concentration. The benzimidazole derivatives **8h**, **8k**, and **8u** showed potent VEGFR-2 inhibition % of 62.88, 69.50, and 80.00%, respectively. The benzimidazole derivative **8u** exhibited a potent inhibitory activity against the target kinases (VEGFR-2, FGFR-1, and BRAF) with IC_50_ values of 0.93, 3.74 and 0.25 µM, respectively.

Simultaneously, compounds **8a-u** were examined at 10 µM for their antiproliferative efficacy at NCI (USA). Compounds **8a**, **8d**, **8h**, **8j**, **8k**, **8o**, **8q**, **8r** and **8u** demonstrated potent activity with GI% > 90% and were further selected to be tested at the five dose assay. It is obvious that incorporation of 2-hydroxyphenyl group in **8a**, **8h**, **8o** or 2-hydroxy, 3-methoxyphenyl group in **8d**, **8k**, **8r** is favourable (mean GI_50_ = 2.62–7.70 µM). Meanwhile, incorporation of 3-methoxyphenyl group is favourable at the five position of 2-(4-methoxyphenylbenzimidazole) **8j** and 2-(4-chlorophenylbenzimidazole) **8q** (mean GI_50_ = 5.24 and 5.40 µM, respectively). Interestingly, the introduction of 3,4,5-trimethoxyphenyl derivative is promising only in 2-(4-chlorophenylbenzimidazole) **8u** (mean GI_50_ = 7.98 µM). Encouraged by the potent activity of **8u** on both the biochemical and cellular assays, it was further assessed for its effect on cell cycle and apoptosis of MCF-7 cell line. Interestingly, **8u** was found to induce cell cycle arrest in MCF-7 cell line at the G2/M phase and accumulating cells at the sub-G1 phase as a result of cell apoptosis.

## Experimental

### Molecular modeling study

The molecular modeling study was carried out using Molecular Operating Environment software (MOE 2022.02) according to the following steps:

#### Common 3D multi-kinase receptor-based pharmacophore model generation

##### Retrieving X-ray crystallographic structures

The X-ray crystallographic structures of VEGFR-2 (PDB ID: 3VHE, 3VNT, and 3VO3), FGFR-1 (PDB ID: 4V01 and 3RHX), and BRAF (PDB ID: 4DBN and 6B8U) were downloaded from the protein data bank [49]. MOE was used to prepare the retrieved protein structures (For further details see Additional file 1: Section 1: computational studies). Finally, correctness of ligands’ structures and reported ligand interactions at the active site were further checked after the protonation step. The different prepared protein structures of VEGFR-2, FGFR-1 and BRAF were aligned and superposed using *Align* protocol in MOE using protein structures’ αCs. Consequently, the co-crystalized ligands were aligned in their bioactive conformation in the binding sites of the different protein structures.

##### Manual 3D receptor-based pharmacophore models generation

Using the *pharmacophore query editor* in MOE, the aligned co-crystalized ligands were used to generate several manual 3D pharmacophores based on their common interactions with the target kinases’ binding sites in the different protein structures. The assigned pharmacophoric features include recognition, shape, and projected site point features representing the main common features responsible for the inhibitors’ binding to the kinase domain hotspots in the different proteins. Moreover, several excluded volumes (with different volumes and number) were included to define the steric extent of the binding sites (See Additional file 1: Section 1: computational studies for further details).

#### Pharmacophore model selection and validation

For pharmacophore model selection and validation, for each kinase of the target kinases, a test set was constructed from active inhibitors and inactive decoys with type II-kinase-inhibitor-like structures (Based on their visual inspection). The test set compounds for each kinase were compiled from the Directory of Useful Decoys-Enhanced (DUD-E) [63] and/or DEKOIS 2.0 [64] databases. A large decoys/actives ratio (≈30) was maintained to mimic the natural ratio in the chemical space between the active and inactive compounds. Table 1 shows the distribution of the actives and decoys for each target kinase (See Additional file 1, Section 1: computational studies for the structure of the active inhibitors used in the test set for each target kinase).

Conformational search was then carried out for the test set compounds using *Stochastic* search in MOE which generates conformations by randomly rotating all bonds (including ring bonds) and randomly inverting tetrahedral centres followed by an all-atom energy minimization.

The generated conformers were virtually screened using the different manually generated pharmacophore models to test their ability to discriminate between the active and inactive compounds in the compiled test sets. MOE pharmacophore search-algorithm begins with prefiltration of the conformers database by calculating the conformer similarity to the pharmacophore model with respect to feature type and distance; followed by a more computationally expensive alignment of the conformer atoms to the query feature points minimizing their deviation from each other using root mean square deviation (RMSD) as the fitness criteria for the alignment quality.

The 3D pharmacophore ability in discriminating between the test set active and inactive compounds was assessed based on its collective results on the whole test set. For each 3D pharmacophore, the total number of true positives **TP**_**t**_, false positives **FP**_**t**_, true negatives **TN**_**t**_, and false negatives **FN**_**t**_ were determined (see Additional file 1, Section: computational studies). To assess the performance of the different generated pharmacophores, a set of assessment metrics were used to select and validate the best one. These metrics include sensitivity **Se**, specificity **Sp**, yield of actives **Ya**, enrichment **E**, accuracy **Acc**, discrimination ratio **DR**, **F1 score** and Mathew’s correlation coefficient **MCC** to evaluate the models’ performance (For further details see Additional file 1, Sect. 1: computational studies) [65].

#### Virtual screening

The pharmacophore model exhibited the best performance on the test set in discriminating between actives and inactive compounds was used to screen an in-house dataset of diverse scaffolds after performing conformational search on the in-house dataset using the same protocol used for the test set compounds (Vide supra).

#### Hit optimization

The promising scaffold was then used as a starting point to develop several optimized derivatives with potential synthetic feasibility which should be, by design, multi-kinase type II inhibitors for the target kinases.

#### Molecular docking study

Finally, molecular docking was used to investigate the ability of the designed compounds to bind to the binding sites of the target kinases accomplishing the key interactions responsible for the kinase inhibitory activity.

The X-ray crystallographic structure of VEGFR-2, FGFR-1, and BRAF co-crystallized with Type II kinase inhibitors (PDB ID: 4ASD, 4V01 and 5CT7, respectively) were downloaded from the protein data bank [32, 39, 49, 56] and were utilized to perform the molecular docking study. Details of the molecular docking procedures are discussed in the Additional file 1, Section 1: computational studies.

#### ADME properties prediction

SwissADME online web tool [57–59] was used to predict the physicochemical and AMDE properties of the target compounds **8a-u** (See Additional file 1, Section 1: computational studies for further details).

### Chemistry

#### General remarks

Chemicals, reagents and solvents were purchased from commercial suppliers. Chemical reactions were followed up by TLC using aluminium plates precoated with silica gel 60 F_245_ (Merck). Uncorrected melting points were measured on a Stuart SMP30 melting point apparatus. Spectral and elemental analyses of **8a-u** were recorded in the laboratory central services, National Research Centre, Cairo, Egypt and faculty of pharmacy, Cairo University. IR spectra (4000–400 cm^−1^) were detected on a Jasco FT/IR 300 E Fourier transform infrared spectrophotometer. ^1^HNMR and ^13^CNMR (DMSO-*d*_6_) were measured at 500 (125) MHz and 400 (100) MHz on Bruker instruments.

#### General procedure for the synthesis of *N*'-(substitutedbenzylidene)-2-(substituted)phenyl-1*H*-benzo[*d*]imidazole-5-carbohydrazide 8a-u

A mixture of 2-phenyl-1*H*-benzo[*d*]imidazole-5-carbohydrazides **6a-c** (0.50 mmol) and benzaldehyde derivatives **7a-g** (0.50 mmol) were reacted in EtOH (20 mL) under reflux in the presence of glacial acetic acid (1 mL) for 2h. The solution was poured into ice–water and neutralized with few drops of NH_4_OH solution. The precipitated crude products **8a-u** were filtered and were crystalized from EtOH to give the pure 2,5-diaryl benzimidazoles **8a-u**.

*N*'-(2-Hydroxybenzylidene)-2-phenyl-1*H*-benzo[*d*]imidazole-5-carbohydrazide (**8a**)

Pale brown powder; yield = 84%; mp 287–289 °C; IR (KBr) *ṽ* 3221, 3059, 2936, 2859, 1663, 1620, 1574, 1489 cm^−1^; ^1^H NMR (500 MHz; DMSO-*d*_6_) *δ*_H_ 6.91–6.98 (m, 2H), 7.30 (t, ^3^*J* = 7.5 Hz, 1H), 7.39 (dt, ^3^*J* = 8.0 Hz, ^4^*J* = 1.5 Hz, 1H), 7.54 (d, ^3^*J* = 7.0 Hz, 1H), 7.58 (t, ^3^*J* = 7.5 Hz, 2H), 7.68 (dd, ^3^*J* = 8.0 Hz, ^4^*J* = 1.0 Hz, 1H), 7.73 (d, ^3^*J* = 8.5 Hz, 1H), 7.86 (d, ^3^*J* = 8.5 Hz, 1H), 8.22 (d, ^3^*J* = 7.5 Hz, 1H), 8.27 (s, 1H), 8.67 (s, 1H), 8.99 (s, 1H), 11.46 (s, 1H), 12.20 ppm (s, 1H); ^13^C NMR (125 MHz; DMSO-*d*_6_) *δ*_C_ 116.40, 116.49, 118.14, 118.66, 119.26, 119.54, 122.13, 126.72, 128.98, 129.58, 129.70, 130.34, 130.86, 131.17, 133.15, 148.10, 153.42, 157.50, 158.62, 162.78, 163.27 ppm; Anal. Calcd for C_21_H_16_N_4_O_2_: C, 70.77; H, 4.53; N, 15.72. Found: C, 70.45; H, 4.88; N, 15.85.

*N*'-(3-Hydroxybenzylidene)-2-phenyl-1*H*-benzo[*d*]imidazole-5-carbohydrazide (**8b**)

Pale brown powder; yield = 89%; mp 308–310 °C; ^1^H NMR (400 MHz; DMSO-*d*_6_) *δ*_H_ 6.83 (d, ^3^*J* = 6.8 Hz, 1H), 7.11 (d, ^3^*J* = 7.2 Hz, 1H), 7.22 (s, 1H), 7.25 (t, ^3^*J* = 8.0 Hz, 1H), 7.51–7.60 (m, 3H), 7.70 (d, ^3^*J* = 8.4 Hz, 1H), 7.82 (d, ^3^*J* = 8.0 Hz, 1H), 8.21 (d, ^3^*J* = 7.2 Hz, 3H), 8.41 (s, 1H), 9.62 (s, 1H), 11.83 (s, 1H), 13.27 ppm (br., 1H); ^13^C NMR (100 MHz; DMSO-*d*_6_) *δ*_C_ 112.69, 117.40, 118.83, 122.25, 126.78, 127.42, 129.15, 129.67, 129.97, 130.48, 135.82, 147.49, 153.39, 157.72, 163.65 ppm; Anal. Calcd for C_21_H_16_N_4_O_2_: C, 70.77; H, 4.53; N, 15.72. Found: C, 70.90; H, 4.75; N, 15.50.

*N*'-(3-Methoxybenzylidene)-2-phenyl-1*H*-benzo[*d*]imidazole-5-carbohydrazide (**8c**)

Pale brown powder; yield = 82%; mp 163–165 °C; IR (KBr) *ṽ* 3159, 3075, 3005, 2974, 2940, 1663, 1604, 1570, 1462 cm^−1^; ^1^H NMR (500 MHz; DMSO-*d*_6_) *δ*_H_ 3.80 (s, 3H), 7.00 (d, ^3^*J* = 7.0 Hz, 1H), 7.29–7.31 (m, 1H), 7.37 (t, ^3^*J* = 7.5 Hz, 1H), 7.40–7.45 (m, 1H), 7.53 (d, ^3^*J* = 7.0 Hz, 1H), 7.58 (t, ^3^*J* = 7.0 Hz, 2H), 7.71 (br., 1H), 7.83 (d, ^3^*J* = 7.5 Hz, 1H), 8.22 (d, ^3^*J* = 7.0 Hz, 2H), 8.48 (s, 1H), 8.68 (s, 1H), 11.93 (s, 1H), 13.25 ppm (br., 1H);^13^C NMR (125 MHz; DMSO-*d*_6_) *δ*_C_ 55.11, 111.17, 112.53, 116.05, 117.44, 119.97, 121.18, 126.69, 127.29, 128.97, 129.69, 129.86, 129.94, 130.27, 135.16, 135.93, 147.18, 153.34, 159.55, 161.25, 163.70 ppm; Anal. Calcd for C_22_H_18_N_4_O_2_: C, 71.34; H, 4.90; N, 15.13. Found: C, 71.00; H, 5.21; N, 15.35.

*N*'-(2-hydroxy-3-methoxybenzylidene)-2-phenyl-1*H*-benzo[*d*]imidazole-5-carbohydrazide (**8d**)

Pale brown powder; yield = 77%; mp 188–190 °C; ^1^H NMR (400 MHz; DMSO-*d*_6_) *δ*_H_ 3.81 (s, 3H), 6.86 (t, ^3^*J* = 8.0 Hz, 1H), 7.03 (d, ^3^*J* = 8.0 Hz, 1H), 7.14 (d, ^3^*J* = 7.6 Hz, 1H), 7.51–7.60 (m, 4H), 7.72 (d, ^3^*J* = 8.4 Hz, 1H), 7.85 (d, ^3^*J* = 8.4 Hz, 1H), 8.22 (d, ^3^*J* = 7.2 Hz, 2H), 8.26 (s, 1H), 8.68 (s, 1H), 11.17 (s, 1H), 12.15 ppm (br., 1H); ^13^C NMR (100 MHz; DMSO-*d*_6_) *δ*_C_ 55.86, 113.82, 118.97, 119.08, 121.10, 122.25, 126.76, 126.81, 129.13, 129.63, 130.49, 147.28, 148.00, 148.03, 153.51, 163.35 ppm; Anal. Calcd for C_22_H_18_N_4_O_3_: C, 68.38; H, 4.70; N, 14.50. Found: C, 68.09; H, 4.97; N, 14.32.

*N*'-(3-Hydroxy-4-methoxybenzylidene)-2-phenyl-1*H*-benzo[*d*]imidazole-5-carbohydrazide (**8e**)

Pale brown powder; yield = 72%; mp 267–269 °C; ^1^H NMR (500 MHz; DMSO-*d*_6_) *δ*_H_ 3.80 (s, 3H), 6.97 (d, ^3^*J* = 8.5 Hz, 1H), 7.06 (d, ^3^*J* = 7.5 Hz, 1H), 7.29 (s, 1H), 7.53 (t like, ^3^*J* = 7.5 Hz, 1H), 7.58 (t like, ^3^*J* = 7.0 Hz, 2H), 7.68 (br., 1H), 7.80 (d, ^3^*J* = 7.0 Hz, 1H), 8.21 (d, ^3^*J* = 7.5 Hz, 2H), 8.34 (s, 1H), 8.51 (s, 1H), 9.34 (s, 1H), 11.72 (s, 1H), 13.22 ppm (br., 1H); ^13^C NMR (125 MHz; DMSO-*d*_6_) *δ*_C_ 55.58, 111.94, 112.41, 120.11, 126.68, 127.39, 128.98, 129.71, 130.27, 146.88, 147.51, 149.68, 153.26, 160.52, 163.38 ppm; Anal. Calcd for C_22_H_18_N_4_O_3_: C, 68.38; H, 4.70; N, 14.50. Found: C, 68.72; H, 4.34; N, 14.77.

*N*'-(2,5-Dimethoxybenzylidene)-2-phenyl-1*H*-benzo[*d*]imidazole-5-carbohydrazide (**8f**)

Pale brown powder; yield = 75%; mp 249–251 °C; IR (KBr) *ṽ* 3256, 3051, 2967, 2928, 1663, 1624, 1601, 1562, 1493, 1454 cm^−1^; ^1^H NMR (500 MHz; DMSO-*d*_6_) *δ*_H_ 3.75 (s, 3H), 3.82 (s, 3H), 6.99 (dd, ^3^*J* = 8.5 Hz, ^4^*J* = 2.5 Hz, 1H), 7.05 (d, ^3^*J* = 9.0 Hz, 1H), 7.41 (s, 1H), 7.53 (t like, ^3^*J* = 7.5 Hz, 1H), 7.58 (t like, ^3^*J* = 7.5 Hz, 2H), 7.70 (d, ^3^*J* = 8.0 Hz, 1H), 7.85 (d, ^3^*J* = 8.5 Hz, 1H), 8.21 (d, ^3^*J* = 7.0 Hz, 2H), 8.24 (br., 1H), 8.83 (s, 1H), 11.94 (s, 1H), 13.19 ppm (br., 1H); ^13^C NMR (125 MHz; DMSO-*d*_6_) *δ*_C_ 55.41, 56.23, 109.28, 113.40, 117.40, 122.15, 123.18, 126.68, 127.31, 128.97, 129.59, 130.30, 142.66, 152.24, 153.27, 163.41 ppm; Anal. Calcd for C_23_H_20_N_4_O_3_: C, 68.99; H, 5.03; N, 13.99. Found: C, 68.71; H, 5.24; N, 14.34.

2-Phenyl-*N*'-(3,4,5-trimethoxybenzylidene)-1*H*-benzo[*d*]imidazole-5-carbohydrazide (**8g**)

Pale brown powder; yield = 80%; mp 178–180 °C; ^1^H NMR (500 MHz; DMSO-*d*_6_) *δ*_H_ 3.70 (s, 3H), 3.84 (s, 6H), 7.04 (s, 2H), 7.53 (t like, ^3^*J* = 7.0 Hz, 1H), 7.58 (t, ^3^*J* = 7.5 Hz, 2H), 7.71 (s, 1H), 7.82 (d, ^3^*J* = 7.5 Hz, 1H), 8.21 (d like, ^3^*J* = 7.0 Hz, 3H), 8.43 (s, 1H), 11.91 (s, 1H), 13.24 ppm (s, 1H); ^13^C NMR (125 MHz; DMSO-*d*_6_) *δ*_C_ 55.92, 60.07, 104.33, 122.19, 126.66, 127.38, 127.38, 127.44, 128.97, 129.69, 129.99, 130.26, 139.23, 147.39, 153.17, 163.68 ppm; Anal. Calcd for C_24_H_22_N_4_O_4_: C, 66.97; H, 5.15; N, 13.02. Found: C, 66.72; H, 5.37; N, 13.29.

*N*'-(2-Hydroxybenzylidene)-2-(4-methoxyphenyl)-1*H*-benzo[*d*]imidazole-5-carbohydrazide (**8h**)

Pale brown powder; yield = 81%; mp 242–244 °C; ^1^H NMR (400 MHz; DMSO-*d*_6_) *δ*_H_ 3.85 (s, 3H), 6.93 (t, ^3^*J* = 8.4 Hz, 2H), 7.14 (d, ^3^*J* = 9.2 Hz, 2H), 7.30 (t, ^3^*J* = 7.6 Hz, 1H), 7.53 (d, ^3^*J* = 7.2 Hz, 1H), 7.67 (d, ^3^*J* = 8.4 Hz, 1H), 7.82 (d, ^3^*J* = 8.4 Hz, 1H), 8.15 (d, ^3^*J* = 8.8 Hz, 2H), 8.20 (br., 1H), 8.66 (s, 1H), 11.44 (s, 1H), 12.14 (s, 1H), 13.15 ppm (s, 1H); ^13^C NMR (100 MHz; DMSO-*d*_6_) *δ*_C_ 55.42, 114.52, 116.44, 118.71, 119.34, 121.89, 121.95, 126.41, 128.44, 129.70, 131.24, 147.98, 153.50, 157.50, 161.11, 163.27 ppm; Anal. Calcd for C_22_H_18_N_4_O_3_: C, 68.38; H, 4.70; N, 14.50. Found C, 68.61; H, 4.95; N, 14.32.

*N*'-(3-Hydroxybenzylidene)-2-(4-methoxyphenyl)-1*H*-benzo[*d*]imidazole-5-carbohydrazide (**8i**)

Pale brown powder; yield = 90%; mp 298–300 °C; IR (KBr) *ṽ* 3210, 3063, 2993, 2940, 2893, 1663, 1613, 1582, 1543 cm^−1^; ^1^H NMR (400 MHz; DMSO-*d*_6_) *δ*_H_ 3.85 (s, 3H), 6.83 (d, ^3^*J* = 8.0 Hz, 1H), 7.10 (d, ^3^*J* = 7.2 Hz, 1H), 7.14 (d, ^3^*J* = 9.2 Hz, 2H), 7.21 (s, 1H), 7.25 (t, ^3^*J* = 7.6 Hz, 1H), 7.65 (d, ^3^*J* = 8.4 Hz, 1H), 7.79 (d, ^3^*J* = 8.4 Hz, 1H), 8.15 (d, ^3^*J* = 8.8 Hz, 3H), 8.40 (s, 1H), 9.62 (s, 1H), 11.80 (s, 1H), 13.10 ppm (br., 1H); ^13^C NMR (100 MHz; DMSO-*d*_6_) *δ*_C_ 55.42, 112.62, 114.52, 117.34, 118.77, 121.94, 122.10, 127.08, 128.40, 129.92, 135.82, 147.36, 153.47, 157.70, 161.05, 163.63 ppm; Anal. Calcd for C_22_H_18_N_4_O_3_: C, 68.38; H, 4.70; N, 14.50. Found: C, 68.14; H, 4.91; N, 14.88.

*N*'-(3-Methoxybenzylidene)-2-(4-methoxyphenyl)-1*H*-benzo[*d*]imidazole-5-carbohydrazide (**8j**)

Pale brown powder; yield = 90%; mp 203–205 °C; IR (KBr) *ṽ* 3159, 3075, 3028, 3009, 2971, 1663, 1612, 1566, 1493 cm^−1^; ^1^H NMR (400 MHz; DMSO-*d*_6_) *δ*_H_ 3.80 (s, 3H), 3.84 (s, 3H), 7.00 (d, ^3^*J* = 7.6 Hz, 1H), 7.14 (d, ^3^*J* = 8.4 Hz, 2H), 7.30 (br., 2H), 7.37 (t, ^3^*J* = 8.0 Hz, 1H), 7.66 (d, ^3^*J* = 8.4 Hz, 1H), 7.81 (d, ^3^*J* = 8.4 Hz, 1H), 8.16 (d, ^3^*J* = 8.8 Hz, 2H), 8.19 (s, 1H), 8.48 (s, 1H), 11.90 ppm (s, 1H); ^13^C NMR (100 MHz; DMSO-*d*_6_) *δ*_C_ 55.17, 55.40, 111.15, 114.50, 116.09, 120.01, 121.99, 127.05, 128.40, 129.95, 135.97, 147.15, 153.42, 159.57, 161.07, 163.66 ppm; Anal. Calcd for C_23_H_20_N_4_O_3_: C, 68.99; H, 5.03; N, 13.99. Found: C, 68.67; H, 5.31; N, 14.23.

*N*'-(2-Hydroxy-3-methoxybenzylidene)-2-(4-methoxyphenyl)-1*H*-benzo[*d*]imidazole-5-carbohydrazide (**8k**)

Pale brown powder; yield = 82%; mp 192–194 °C; ^1^H NMR (400 MHz; DMSO-*d*_6_) *δ*_H_ 3.81 (s, 3H), 3.85 (s, 3H), 6.87 (t, ^3^*J* = 8.0 Hz, 1H), 7.03 (d, ^3^*J* = 8.0 Hz, 1H), 7.13 (d, ^3^*J* = 6.4 Hz, 1H), 7.14 (d, ^3^*J* = 8.8 Hz, 2H), 7.67 (d, ^3^*J* = 8.4 Hz, 1H), 7.82 (d, ^3^*J* = 8.4 Hz, 1H), 8.16 (d, ^3^*J* = 8.8 Hz, 2H), 8.20 (s, 1H), 8.67 (s, 1H), 11.18 (s, 1H), 12.13 (s, 1H), 13.32 ppm (br., 1H); ^13^C NMR (100 MHz; DMSO-*d*_6_) *δ*_C_ 55.47, 55.88, 113.83, 114.58, 118.98, 119.10, 121.12, 121.96, 122.02, 126.49, 128.51, 147.28, 148.01, 153.60, 161.17, 163.39 ppm; Anal. Calcd for C_23_H_20_N_4_O_4_: C, 66.34; H, 4.84; N, 13.45. Found: C, 66.67; H, 4.98; N, 13.17.

*N*'-(3-Hydroxy-4-methoxybenzylidene)-2-(4-methoxyphenyl)-1*H*-benzo[*d*]imidazole-5-carbohydrazide (**8l**)

Pale brown powder; yield = 82%; mp > 250 °C [66]; ^1^H NMR (400 MHz; DMSO-*d*_6_) *δ*_H_ 3.80 (s, 3H), 3.85 (s, 3H), 6.97 (d, ^3^*J* = 8.4 Hz, 1H), 7.06 (d, ^3^*J* = 8.0 Hz, 1H), 7.14 (d, ^3^*J* = 8.8 Hz, 2H), 7.28 (s, 1H), 7.65 (d, ^3^*J* = 8.4 Hz, 1H), 7.78 (d, ^3^*J* = 8.4 Hz, 1H), 8.15 (d, ^3^*J* = 8.4 Hz, 2H), 8.16 (s, 1H), 8.33 (s, 1H), 9.30 (s, 1H), 11.68 ppm (s, 1H); ^13^C NMR (100 MHz; DMSO-*d*_6_) *δ*_C_ 55.50, 55.66, 111.96, 112.43, 114.61, 120.32, 121.92, 122.05, 127.42, 128.52, 146.93, 147.61, 149.80, 153.42, 161.19, 163.54 ppm; Anal. Calcd for C_23_H_20_N_4_O_4_: C, 66.34; H, 4.84; N, 13.45. Found: C, 66.02; H, 4.71; N, 13.21.

*N*'-(2,5-Dimethoxybenzylidene)-2-(4-methoxyphenyl)-1*H*-benzo[*d*]imidazole-5-carbohydrazide (**8m**)

Pale brown powder; yield = 80%; mp 181–183 °C; IR (KBr) *ṽ* 3206, 3159, 3059, 3005, 2940, 1643, 1613, 1555, 1493, 1466 cm^−1^; ^1^H NMR (400 MHz; DMSO-*d*_6_) *δ*_H_ 3.76 (s, 3H), 3.82 (s, 3H), 3.85 (s, 3H), 7.00 (dd, ^3^*J* = 9.2 Hz, ^4^*J* = 2.8 Hz, 1H), 7.06 (d, ^3^*J* = 9.2 Hz, 1H), 7.14 (d, ^3^*J* = 8.8 Hz, 2H), 7.40 (s, 1H), 7.64 (d, ^3^*J* = 8.4 Hz, 1H), 7.80 (d, ^3^*J* = 8.4 Hz, 1H), 8.15 (d, ^3^*J* = 8.8 Hz, 2H), 8.18 (s, 1H), 8.82 (br., 1H), 11.88 (s, 1H), 13.14 ppm (br., 1H); ^13^C NMR (100 MHz; DMSO-*d*_6_) *δ*_C_ 55.42, 55.46, 56.26, 109.25, 113.42, 114.52, 117.48, 122.03, 123.17, 127.03, 128.42, 142.63, 152.27, 153.30, 153.43, 161.08, 163.54 ppm; Anal. Calcd for C_24_H_22_N_4_O_4_: C, 66.97; H, 5.15; N, 13.02. Found: C, 66.70; H, 5.44; N, 13.30.

2-(4-Methoxyphenyl)-*N*'-(3,4,5-trimethoxybenzylidene)-1*H*-benzo[*d*]imidazole-5-carbohydrazide (**8n**)

Pale brown powder; yield = 87%; mp 290–292 °C [66]; IR (KBr) *ṽ* 3198, 3163, 3079, 2944, 1636, 1613, 1578, 1559, 1504, 1454 cm^−1^; ^1^H NMR (400 MHz; DMSO-*d*_6_) *δ*_H_ 3.71 (s, 3H), 3.84 (s, 3H), 3.85 (s, 6H), 7.03 (s, 2H), 7.14 (d, ^3^*J* = 8.8 Hz, 2H), 7.66 (d, ^3^*J* = 8.4 Hz, 1H), 7.80 (d, ^3^*J* = 8.4 Hz, 1H), 8.15 (d, ^3^*J* = 8.8 Hz, 2H), 8.17 (s, 1H), 8.43 (s, 1H), 11.87 (s, 1H), 13.27 ppm (br., 1H); ^13^C NMR (100 MHz; DMSO-*d*_6_) *δ*_C_ 55.47, 56.00, 60.19, 104.33, 114.59, 121.84, 122.12, 127.23, 128.50, 130.09, 139.19, 147.41, 153.26, 153.42, 161.19, 163.76 ppm; Anal. Calcd for C_25_H_24_N_4_O_5_: C, 65.21; H, 5.25; N, 12.17. Found: C, 65.01; H, 5.55; N, 12.47.

2-(4-Chlorophenyl)-*N*'-(2-hydroxybenzylidene)-1*H*-benzo[*d*]imidazole-5-carbohydrazide (**8o**)

Pale brown powder; yield = 70%; mp 287–289 °C; IR (KBr) *ṽ* 3206, 3063, 1667, 1624, 1555, 1478, 1447 cm^−1^; ^1^H NMR (400 MHz; DMSO-*d*_6_) *δ*_H_ 6.91–6.95 (m, 2H), 7.30 (t like, ^3^*J* = 7.2 Hz, 1H), 7.53 (d, ^3^*J* = 6.8 Hz, 1H), 7.66 (d, ^3^*J* = 8.4 Hz, 2H), 7.73 (br., 1H), 7.85 (d, ^3^*J* = 8.0 Hz, 1H), 8.22 (d, ^3^*J* = 8.4 Hz, 2H), 8.32 (br., 1H), 8.66 (s, 1H), 11.43 (s, 1H), 12.16 (s, 1H), 13.31 ppm (br., 1H); ^13^C NMR (100 MHz; DMSO-*d*_6_) *δ*_C_ 111.83, 116.45, 118.71, 119.33, 122.57, 126.82, 128.45, 128.55, 129.19, 129.71, 131.26, 135.08, 148.06, 152.42, 157.53, 163.23 ppm; Anal. Calcd for C_21_H_15_ClN_4_O_2_: C, 64.54; H, 3.87; N, 14.34. Found: C, 64.78; H, 3.57; N, 14.51.

2-(4-Chlorophenyl)-*N*'-(3-hydroxybenzylidene)-1*H*-benzo[*d*]imidazole-5-carbohydrazide (**8p**)

Pale brown powder; yield = 75%; mp 313–315 °C; ^1^H NMR (400 MHz; DMSO-*d*_6_) *δ*_H_ 6.83 (d, ^3^*J* = 8.0 Hz, 1H), 7.10 (d, ^3^*J* = 7.2 Hz, 1H), 7.22 (s, 1H), 7.25 (t, ^3^*J* = 8.0 Hz, 1H), 7.66 (d, ^3^*J* = 8.4 Hz, 2H), 7.70 (d, ^3^*J* = 8.4 Hz, 1H), 7.82 (d, ^3^*J* = 8.4 Hz, 1H), 8.22 (d, ^3^*J* = 8.8 Hz, 2H), 8.40 (s, 1H), 9.62 (s, 1H), 11.83 (s, 1H), 11.94 (br., 1H), 13.28 ppm (s, 1H); ^13^C NMR (100 MHz; DMSO-*d*_6_) *δ*_C_ 112.62, 117.35, 118.78, 122.43, 127.56, 128.45, 128.58, 129.20, 129.90, 135.07, 135.78, 147.44, 152.27, 157.70, 163.50 ppm; Anal. Calcd for C_21_H_15_ClN_4_O_2_: C, 64.54; H, 3.87; N, 14.34. Found: C, 64.19; H, 3.95; N, 14.55.

2-(4-Chlorophenyl)-*N*'-(3-methoxybenzylidene)-1*H*-benzo[*d*]imidazole-5-carbohydrazide (**8q**)

Pale brown powder; yield = 78%; mp 171–173 °C; ^1^H NMR (400 MHz; DMSO-*d*_6_) *δ*_H_ 3.81 (s, 3H), 7.01 (d, ^3^*J* = 7.6 Hz, 1H), 7.30 (s, 2H), 7.37 (t, ^3^*J* = 7.6 Hz, 1H), 7.66 (d, ^3^*J* = 8.8 Hz, 2H), 7.71 (d, ^3^*J* = 8.4 Hz, 1H), 7.84 (d, ^3^*J* = 8.0 Hz, 1H), 8.22 (d, ^3^*J* = 8.8 Hz, 3H), 8.47 (s, 1H), 11.91 (s, 1H), 13.78 ppm (br., 1H); ^13^C NMR (100 MHz; DMSO-*d*_6_) *δ*_C_ 55.17, 111.17, 116.13, 120.04, 122.47, 127.57, 128.40, 128.48, 129.20, 129.95, 135.15, 135.94, 147.29, 152.22, 159.57, 163.57 ppm; Anal. Calcd for C_22_H_17_ClN_4_O_2_: C, 65.27; H, 4.23; N, 13.84. Found: C, 65.49; H, 4.50; N, 13.63.

2-(4-Chlorophenyl)-*N*'-(2-hydroxy-3-methoxybenzylidene)-1*H*-benzo[*d*]imidazole-5-carbohydrazide (**8r**)

Pale brown powder; yield = 87%; mp 187–190 °C; ^1^H NMR (400 MHz; DMSO-*d*_6_) *δ*_H_ 3.81 (s, 3H), 6.86 (t, ^3^*J* = 7.6 Hz, 1H), 7.03 (d, ^3^*J* = 7.6 Hz, 1H), 7.14 (d, ^3^*J* = 7.6 Hz, 1H), 7.69 (d, ^3^*J* = 8.8 Hz, 2H), 7.75 (d, ^3^*J* = 8.4 Hz, 1H), 7.90 (d, ^3^*J* = 8.4 Hz, 1H), 8.23 (d, ^3^*J* = 8.4 Hz, 2H), 8.27 (s, 1H), 8.69 (s, 1H), 11.14 (br., 1H), 12.19 ppm (s, 1H); ^13^C NMR (100 MHz; DMSO-*d*_6_) *δ*_C_ 55.82, 113.79, 114.61, 115.27, 118.92, 119.02, 121.01, 122.89, 127.45, 128.73, 129.30, 135.67, 138.17, 140.30, 147.24, 147.95, 148.10, 151.98, 162.98 ppm; Anal. Calcd for C_22_H_17_ClN_4_O_3_: C, 62.79; H, 4.07; N, 13.31. Found: C, 62.98; H, 4.32; N, 13.66.

2-(4-Chlorophenyl)-*N*'-(3-hydroxy-4-methoxybenzylidene)-1*H*-benzo[*d*]imidazole-5-carbohydrazide (**8s**)

Pale brown powder; yield = 87%; mp 298–300 °C; ^1^H NMR (400 MHz; DMSO-*d*_6_) *δ*_H_ 3.81 (s, 3H), 6.97 (d, ^3^*J* = 8.0 Hz, 1H), 7.06 (d, ^3^*J* = 7.6 Hz, 1H), 7.29 (s, 1H), 7.67 (t like, ^3^*J* = 8.8 Hz, 3H), 7.81 (d, ^3^*J* = 8.4 Hz, 1H), 8.22 (d, ^3^*J* = 8.8 Hz, 3H), 8.34 (s, 1H), 9.29 (s, 1H), 11.70 (s, 1H), 13.24 ppm (s, 1H); ^13^C NMR (100 MHz; DMSO-*d*_6_) *δ*_C_ 55.61, 111.92, 112.36, 120.25, 122.38, 127.37, 127.77, 128.47, 128.57, 129.22, 135.09, 146.91, 147.60, 149.75, 152.23, 163.36 ppm; Anal. Calcd for C_22_H_17_ClN_4_O_3_: C, 62.79; H, 4.07; N, 13.31. Found: C, 62.51; H, 4.19; N, 13.42.

2-(4-Chlorophenyl)-*N*'-(2,5-dimethoxybenzylidene)-1*H*-benzo[*d*]imidazole-5-carbohydrazide (**8t**)

Pale brown powder; yield = 91%; mp 190–192 °C; ^1^H NMR (400 MHz; DMSO-*d*_6_) *δ*_H_ 3.75 (s, 3H), 3.82 (s, 3H), 6.99 (dd, ^3^*J* = 8.8 Hz, ^4^*J* = 2.8 Hz, 1H), 7.05 (d, ^3^*J* = 9.2 Hz, 1H), 7.41 (s, 1H), 7.66 (d, ^3^*J* = 8.4 Hz, 2H), 7.69 (d, ^3^*J* = 8.8 Hz, 1H), 7.85 (d, ^3^*J* = 8.4 Hz, 1H), 8.22 (d, ^3^*J* = 8.8 Hz, 2H), 8.24 (d, ^4^*J* = 2.0 Hz, 1H), 8.83 (s, 1H), 11.92 (s, 1H), 13.39 ppm (br., 1H); ^13^C NMR (100 MHz; DMSO-*d*_6_) *δ*_C_ 56.06, 56.24, 109.24, 113.39, 117.48, 122.44, 123.13, 127.52, 128.46, 129.19, 135.10, 142.73, 152.22, 152.27, 153.28, 163.40 ppm; Anal. Calcd for C_23_H_19_ClN_4_O_3_: C, 63.52; H, 4.40; N, 12.88. Found: C, 63.70; H, 4.72; N, 12.51.

2-(4-Chlorophenyl)-*N*'-(3,4,5-trimethoxybenzylidene)-1*H*-benzo[*d*]imidazole-5-carbohydrazide (**8u**)

Pale brown powder; yield = 86%; mp 300–302 °C; ^1^H NMR (400 MHz; DMSO-*d*_6_) *δ*_H_ 3.71 (s, 3H), 3.84 (s, 6H), 7.04 (s, 2H), 7.73 (d, ^3^*J* = 8.4 Hz, 2H), 7.78 (d, ^3^*J* = 8.4 Hz, 1H), 7.90 (d, ^3^*J* = 8.4 Hz, 1H), 8.21–8.25 (m, 3H), 8.44 (s, 1H), 11.94 ppm (s, 1H); ^13^C NMR (100 MHz; DMSO-*d*_6_) *δ*_C_ 55.95, 60.12, 104.27, 114.51, 114.97, 123.31, 126.68, 128.61, 128.89, 129.41, 129.94, 136.06, 139.19, 147.64, 151.60, 153.20, 163.15 ppm; Anal. Calcd for C_24_H_21_ClN_4_O_4_: C, 62.00; H, 4.55; N, 12.05. Found C, 62.32; H, 4.40; N, 12.28.

### Biology

#### Screening of the inhibitory activity of 2,5-disubstituted benzimidazoles 8a-u on VEGFR-2

The VEGFR-2 inhibitory activity of **8a-u** was investigated at 10 µM using VEGFR-2 assay kit (BPS Biosciences—San Diego—CA—US) according to the protocol provided by the manufacturer [68] and the % of inhibition was determined (For further details see Additional file 1: section II: practical results) [67].

#### Screening of the inhibitory activity of 8u on diverse kinases

The inhibitory activity of **8u** was investigated at different concentrations using VEGFR-2, FGFR-1 and BRAF assay kits (BPS Biosciences—San Diego—CA—US) according to the protocol provided by the manufacturer (For further details see Additional file 1: section II: practical results).

#### Cell cycle analysis assay

The diaryl benzimidazole **8u** was applied at its GI_50_ concentration to MCF-7 cancer cells. After the cells were handled as previously described [69], the distribution of the cells at each stage of the cell cycle was analysed. (For further details see Additional file 1: section II: practical results).

#### Apoptosis assay

As previously reported, the Annexin V-FITC apoptosis detection kit (Abcam Inc., Cambridge, UK) in conjunction with two fluorescent channels flow cytometry was used to identify the populations of apoptosis and necrosis cells [69]. (For further details see Additional file 1: section II: practical results).

### Supplementary Information

**Additional file 1.** Section I: Computational Studies: 1. Common 3D multi-kinase receptor-based pharmacophore model generation, 2. Pharmacophore model selection and validation, 3. Molecular docking simulation, 4. ADME properties prediction. Section II: Practical Results: 1. NMR Spectra of 2,5-disubstituted benzimidazole **8a-u,** 2. Biochemical kinase assay procedure, 3. Dose response curves of 8u on VEGFR-2, FGFR-1 and BRAF, 4. Screening of cytotoxic activity against a panel of sixty human tumor cell lines, 5. Dose response curves of the 2,5-diaryl benzimidazole conjugates on NCI cancer cell lines, 6. Analysis of cell cycle distribution, 7. Apoptosis assay.

## Data Availability

Supporting materials word file is available in the online version of this article.

## Abbreviations

Å: Angstrom
Acc: Acceptor feature
Acc: Accuracy
ADME: Absorption, distribution, metabolism, and excretion
ATP: Adenosine triphosphate
BBB: Blood brain barrier
BRAF: B-rapidly accelerated fibrosarcoma
CADD: Computer-aided drug design
CNS: Central nervous system
DMSO: Dimethyl sulfoxide
DR: Discrimination ratio
DUD-E: Directory of useful decoys-enhanced
E: Enrichment
EGFR: Epidermal Growth Factor Receptor
FGF: Fibroblast Growth Factor
FGFR-1: Fibroblast growth factor receptor-1
FN_t_: Total false negatives
FP_t_: Total false positives
GIT: Gastrointestinal tract
GI: Growth inhibition
hFGFR: Human fibroblast growth factor receptor
Hyd: Hydrophobic feature
IC_50_: Half maximal inhibitory concentration
LBDD: Ligand-based drug design
MAPK: Mitogen activated protein kinases
MCC: Mathew’s correlation coefficient
MHz: Megahertz
µM: Micromolar
MOE: Molecular operating environment
NCI: National Cancer Institute
NMR: Nuclear magnetic resonance
nd: Not detected
PDB: Protein Data Bank
P-gp: Protein glycoprotein
Ph4: Pharmacophore
PKIs: Protein kinase inhibitors
ppm: Parts per million
PI: Propidium iodide
RAF: Rapidly accelerated fibrosarcoma
RAS: Reticular activating system
RMSD: Root mean square deviation
SAR: Structure activity relationship
SBDD: Structure-based drug design
SD: Standard deviation
Se: Sensitivity
Sp: Specificity
3D: Three dimensional
TLC: Thin layer chromatography
TP_t_: Total true positives
TN_t_: Total true negatives
2D: Two dimensional
USA: United States of America
VEGF: Vascular endothelial growth factor
VEGFR-2: Vascular endothelial growth factor receptor-2
Ya: Yield of actives
