# Supplementary material for: Receptor-based pharmacophore modeling, molecular docking, synthesis and biological evaluation of novel VEGFR-2, FGFR-1, and BRAF multi-kinase inhibitors
Source: BMC Chem. 2024 Feb 23;18(1):42. doi: 10.1186/s13065-024-01135-0 (PMC10893631; doi:10.1186/s13065-024-01135-0)
Supplement: Supplementary file 1 — Additional file 1. Section I: Computational Studies: 1. Common 3D multi-kinase receptor-based pharmacophore model generation, 2. Pharmacophore model selection and validation, 3. Molecular docking simulation, 4. ADME properties prediction. Section II: Practical Results: 1. NMR Spectra of 2,5-disubstituted benzimidazole 8a-u, 2. Biochemical kinase assay procedure, 3. Dose response curves of 8u on VEGFR-2, FGFR-1 and BRAF, 4. Screening of cytotoxic activity against a panel of sixty human tumor cell lines, 5. Dose response curves of the 2,5-diaryl benzimidazole conjugates on NCI cancer cell lines, 6. Analysis of cell cycle distribution, 7. Apoptosis assay. [file 13065_2024_1135_MOESM1_ESM.pdf]

Supporting Materials

Receptor-based Pharmacophore Modeling, Molecular Docking, Synthesis and Biological Evaluation of Novel VEGFR-2, FGFR1 and BRAF Multi-kinase Inhibitors

Heba T. Abdel-Mohsen[1]*, Marwa A. Ibrahim[2], Amira M. Nageeb[3], Ahmed M. El Kerdawy[2,4]

[1]Chemistry of Natural and Microbial Products Department, Pharmaceutical and Drug Industries Research Institute, National Research Centre, Dokki, P.O. 12622, Cairo, Egypt.

[2]Department of Pharmaceutical Chemistry, Faculty of Pharmacy, Cairo University, Kasr El-Aini Street, P.O. 11562, Cairo, Egypt.

[3]High Throughput Molecular & Genetic Technology Lab, Center of Excellence for Advanced Sciences, Biochemistry Department, Biotechnology Research Institute, National Research Centre, Dokki, P.O. 12622, Cairo, Egypt

[4]School of Pharmacy, College of Health and Science, University of Lincoln, Joseph Banks Laboratories, Green Lane, Lincoln, Lincolnshire, United Kingdom.

*Correspondence *****Heba T. Abdel-Mohsen; email address; hebabdelmohsen@gmail.com; ht.abdel-mohsen@nrc.sci.eg

Content **Page**

Section I: Computational Studies

1. Common 3D multi-kinase receptor-based pharmacophore model generation 3

2. Pharmacophore model selection and validation 11

3. Molecular docking simulation 19

4. ADME properties prediction 58

Section II: Practical Results

1. NMR Spectra of 2,5-disubstituted benzimidazole 8a-u 60

2. Biochemical kinase assay procedure 102

3. Dose response curves of 8u on VEGFR-2, FGFR-1 and BRAF 103

4. Screening of cytotoxic activity against a panel of sixty human tumor cell lines 104

5. Dose response curves of the 2,5-diaryl benzimidazole conjugates on NCI cancer cell lines 106

6. Analysis of cell cycle distribution 115

7. Apoptosis assay 115

Section I: Computational Studies

1. Common 3D multi-kinase receptor-based pharmacophore model generation

1.1. X-ray crystallographic structures selection and preparation

The initial protein structures of the target kinases VEGFR-2, FGFR-1, and BRAF were selected based on the following considerations:

1- In the Protein Data Bank, we searched for the holo-protein structures (protein + inhibitor) of the target kinases VEGFR-2, FGFR-1, and BRAF from 2010 till September 2021, and PDB IDs were retrieved.
2- All PDB IDs of mutated target proteins were excluded.
3- Only PDB IDs co-crystallized with type II inhibitors were retained.
4- Representative structures were selected that are co-crystalized with potent structurally diverse type II kinase inhibitors.
5- Different kinase structures co-crystalized with the same ligand were also preferred.
6- For FGFR-1, type II inhibitors of the protein were available co-crystallized only with mutated FGFR-1 structures. Thus, we made sure that the site of mutation was not in the active site or affect the functionality of the protein.
7- Finally, the X-ray crystallographic structures of VEGFR-2 (PDB ID: 3VHE, 3VNT, and 3VO3), FGFR-1 (PDB ID: 4V01 and 3RHX), and BRAF (PDB ID: 4DBN and 6B8U) were selected. The solved co-crystal structures of the target proteins FGFR-1 (PDB ID: 4V01 and 3RHX) and BRAF (PDB ID: 4DBN and 6B8U) showed a symmetric dimer, hence the most complete chain from each protein structure was used in the computational study.
8- The retained proteins’ chains were prepared according to the following table.

Table S1. Preparation of the kinase protein structures used for receptor-based pharmacophore model generation.

PDB ID	Preparation
3VHE	Water molecules were removed, then the protein was prepared using *Protonate 3D* protocol in MOE with default options.
3VNT	Water molecules and ligands that are not involved in binding were first removed, then the protein was prepared using *Protonate 3D* protocol in MOE with default options.
3VO3	Water molecules and ligands that are not involved in binding were first removed, then the protein was prepared using *Protonate 3D* protocol in MOE with default options.
4V01	Chain A, water molecules and ligands that are not involved in binding were first removed, then the protein was prepared using *Protonate 3D* protocol in MOE with default options.
3RHX	Chain A, water molecules and ligands that are not involved in binding were first removed, then the protein was prepared using *Protonate 3D* protocol in MOE with default options.
4DBN	Chain A and water molecules were first removed, then the protein was prepared using *Protonate 3D* protocol in MOE with default options.
6B8U	Chain B and water molecules were first removed, then the protein was prepared using *Protonate 3D* protocol in MOE with default options.

Figures S1-3 show the main ligand-target interactions performed by the co-crystalized ligands in the different protein structure active sites.

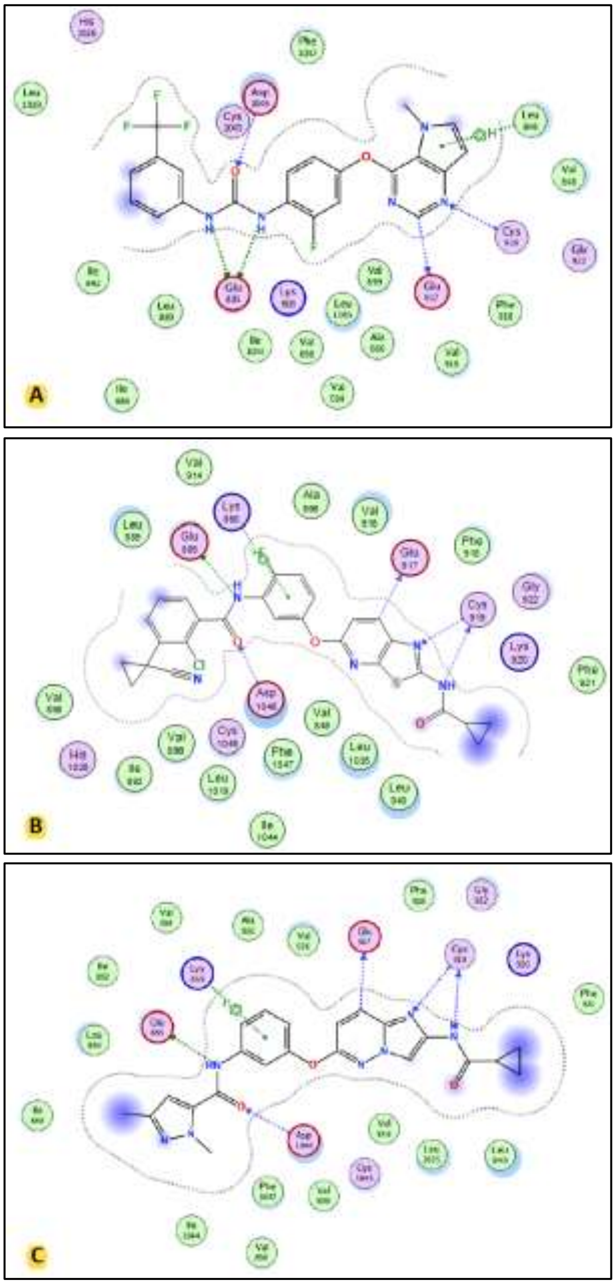

Figure S1. 2D interaction diagrams **A-C** showing ligands' interactions with the key amino acids in the VEGFR2 (DFG out conformation) active site for the selected PDB IDs: 3VHE, 3VNT and 3VO3, respectively.

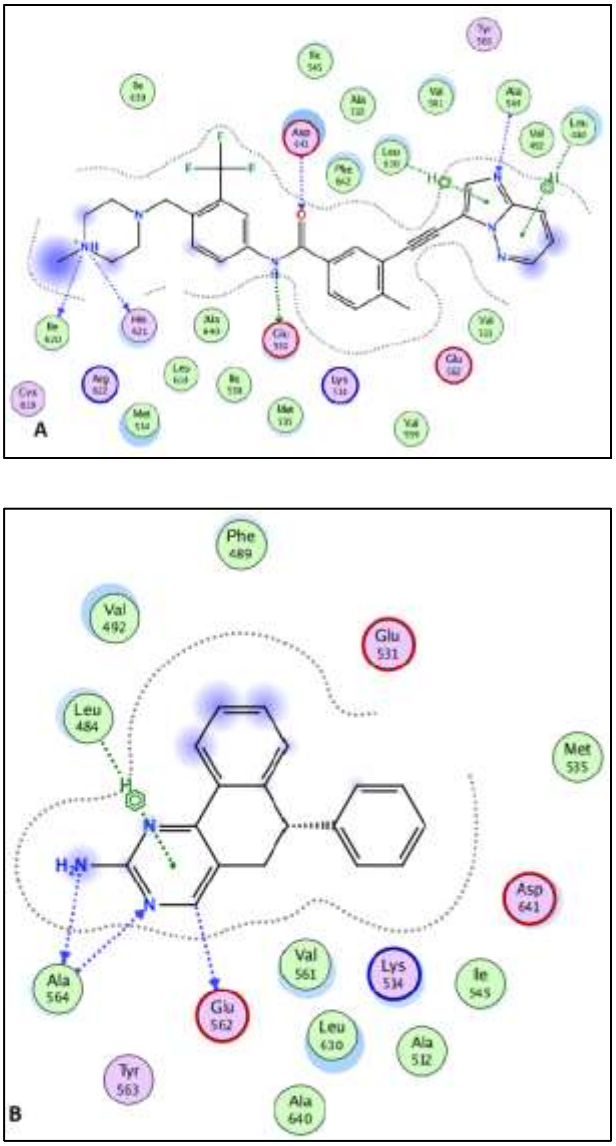

Figure S2. 2D interaction diagrams **A-B** showing ligands' interactions with the key amino acids in the FGFR-1 (DFG out conformation) active site for the selected PDB IDs: 4V01 and 3RHX, respectively.

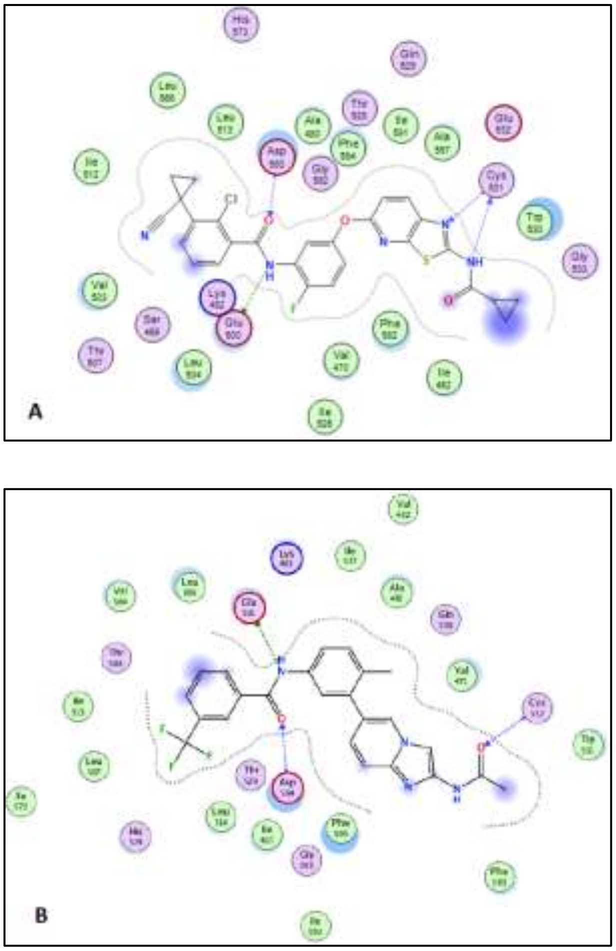

Figure S3. 2D interaction diagrams **A-B** showing ligands' interactions with the key amino acids in the BRAF (DFG out conformation) active site for the selected PDB IDs: 4DBN and 6B8U, respectively.

1.2. Manual 3D receptor-based pharmacophore models generation

Table S2. The full description of the generated Pharmacophore models' features

Pharmacophore Model no.	No. Of features	Feature types (Radius in Å)							No. Of excluded volumes
		F1	F2	F3	F4	F5	F6	F7	
1-3	7	Acc (1.5)	Don (1.9)	Acc (1.1)	Hyd (2.5)	Acc2 (2.2)	Don2 (1.6)	Acc2 (2.2)	30
4	7	Acc (1.5)	Don (1.9)	Acc (1.1)	Hyd (2.5)	Acc2 (2.2)	Don2 (1.6)	Acc2 (2.2)	30
5-6	7	Acc (1.5)	Don (1.9)	Acc (1.1)	Hyd (2.5)	Acc2 (2.2)	Don2 (1.6)	Acc2 (2.2)	30
7-10	7	Acc (1.8)	Don (1.9)	Acc (1.1)	Hyd (2.5)	Acc2 (2.2)	Don2 (1.6)	Acc2 (2.2)	30
11	7	Acc (2)	Don (1.9)	Acc (1.1)	Hyd (2.5)	Acc2 (2.2)	Don2 (1.6)	Acc2 (2.2)	30
12	7	Acc (2.4)	Don (1.9)	Acc (1.1)	Hyd (2.5)	Acc2 (2.5)	Don2 (1.6)	Acc2 (2.2)	30
13	7	Acc (2.4)	Don (1.9)	Acc (1.9)	Hyd (2.5)	Acc2 (2.5)	Don2 (2)	Acc2 (2.2)	30
14-15	7	Acc (2.4)	Don (1.9)	Acc (1.6)	Hyd (2.5)	Acc2 (2.4)	Don2 (2)	Acc2 (2.2)	30
16-17	7	Acc (1.6)	Don (1.9)	Acc (1.4)	Hyd (1.3)	Acc2 (2.4)	Don2 (1.6)	Acc2 (2.2)	32

Table S3. Radii of the excluded volumes used in the generated manual pharmacophores.

Excluded volume	Radius (Å)																
	Ph-1	Ph-2	Ph-3	Ph-4	Ph-5	Ph-6	Ph-7	Ph-8	Ph-9	Ph-10	Ph-11	Ph-12	Ph-13	Ph-14	Ph-15	Ph-16	Ph-17
**V1**	1.3	1.3	1.3	1.3	1.3	1.3	1.3	1.3	1.3	1.3	1.3	1.3	1.3	1.3	1.8	1.2	1.2
**V2**	1.2	1.2	1.2	1.2	1.2	1.2	1.2	0.6	1.2	1.2	1.2	1.2	1.2	1.4	1.4	1.2	1.2
**V3**	1.3	1.3	1.3	1.3	1.3	1.3	1.3	1.3	1.3	1.3	1.3	1.3	1.3	1.3	1.6	1.4	1.4
**V4**	1	1	1	1	1	1	1	1	1	1	1	1	1	1	2.3	1.6	1.6
**V5**	1.3	1.3	1.3	1.3	1.3	1.3	1.3	1.3	1.3	1.3	1.3	1.3	1.3	1.5	1.5	1.2	1.2
**V6**	0.9	0.9	0.9	0.9	0.9	0.9	0.9	0.9	0.7	0.9	0.9	1	1	1.1	1.1	1	1
**V7**	1.2	1.2	1.2	1.2	1.2	1.2	1.2	1.2	0.8	1	1	1.1	0.6	0.6	0.6	1.1	1.1
**V8**	1	1	1	1	1	1	1	1	1	1	1	1	1	1	2.2	1.3	1.3
**V9**	0.8	0.8	0.8	0.8	0.8	0.8	0.8	0.8	0.8	0.8	0.8	0.8	0.8	0.8	2.2	1.1	1.1
**V10**	1.3	1.3	1.3	1.3	1.3	1.3	1.3	1.3	1.3	1.3	1.3	1.3	1.3	1.3	2.5	1.3	1.3
**V11**	0.9	0.9	0.9	0.9	0.9	0.9	0.9	0.9	0.9	0.9	0.9	0.9	0.9	0.9	2	1	1
**V12**	1.1	1.1	1.1	1.1	1.1	1.1	1.1	1.1	1.1	1.1	1.1	1.1	0.8	0.8	0.8	1.1	1.1
**V13**	1	1	1	1	1	1	1	1	0.7	0.9	0.9	2.5	2.5	2.5	2.5	1.1	1.1
**V14**	0.7	0.7	0.7	1	1.2	1.2	1	1	1	1	1	1	1	1	1	1.3	1.3
**V15**	1.1	1.5	1.5	1.5	1.5	1.5	1.5	1.5	1.5	1.5	1.5	1.5	1.5	1.5	1.8	1.1	1.1
**V16**	1.1	1.1	1.1	1.1	1.1	1.1	1.1	1.1	1.1	1.1	1.1	1.1	1.1	1.1	1.1	1.3	1.3
**V17**	1.5	1.5	1.5	1.5	1.5	1.5	1.5	1.5	1.5	1.5	1.5	1.5	1.5	1.5	1.5	1	1
**V18**	1	1	1	1	1	1	1	1	1	1	1	1	1	1	2	1.5	1.5
**V19**	1.3	1.3	1.3	1.3	1.3	1.3	1.1	1.1	1.1	1.3	1.3	1.3	1	1.1	1.1	1.1	1.1
**V20**	1	1	1	1	1	1	1	1	1	1	1	1	1	1	1.7	1.1	1.1
**V21**	1.1	1.1	1.1	1.1	1.1	1.1	1.1	1.1	1.1	1.1	1.1	1.1	1.1	1.1	2	1.3	1.3
**V22**	1.1	1.1	1.1	1.1	1.1	1.1	1.1	1.1	1.1	1.1	1.1	1.1	1.1	1.1	2.5	0.9	0.9
**V23**	0.9	0.9	0.9	0.9	0.9	0.9	0.9	0.9	0.9	0.9	0.9	0.9	0.9	0.9	2	1.3	1.3
**V24**	1.2	1.2	1.2	1.2	1.2	1.2	0.8	0.8	0.8	1	1	1	1	1	1	1.2	1.2
**V25**	0.8	0.8	0.8	0.8	0.8	0.8	0.6	0.8	0.6	1.2	0.8	1.5	1.5	2.3	2.3	2	2
**V26**	0.9	0.9	0.9	0.9	0.9	0.9	0.9	0.9	0.9	0.9	0.9	0.9	0.9	0.9	0.9	1.1	1.1
**V27**	0.9	1.2	1.3	1.3	1.3	1.5	1.3	1.3	1.3	1.3	1.3	1.3	1.3	1.3	1.4	0.9	0.9
**V28**	0.8	0.8	0.8	0.8	0.8	0.8	0.8	0.8	0.8	0.8	0.8	0.8	0.8	0.8	1	1.3	1.3

**V29**	1	1	1	1	1	1	1	1	1	1	1	1	1	1	2	1.1	1.1
**V30**	1.1	1.1	1.1	1.1	1.1	1.1	1.1	1.1	1.1	1.1	1.1	1.1	1.1	1.1	2	1.6	1.6
**V31**	-	-	-	-	-	-	-	-	-	-	-	-	-	-	-	1.1	1.1
**V32**	-	-	-	-	-	-	-	-	-	-	-	-	-	-	-	1.2	1.2

2. Pharmacophore model selection and validation

Table S4. The compiled VEGFR-2 active compounds used for pharmacophore selection and validation

#	Structure	#	Structure
**1**	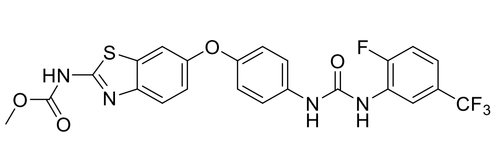	**2**	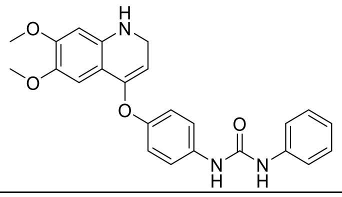
**3**	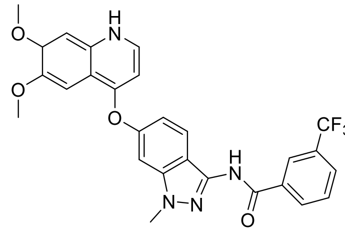	**4**	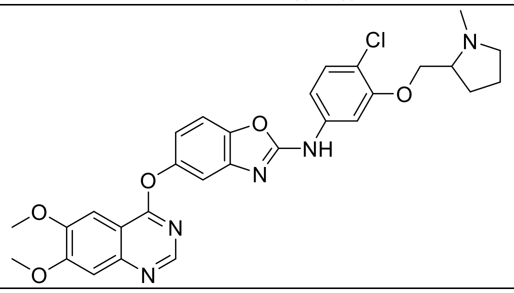
**5**	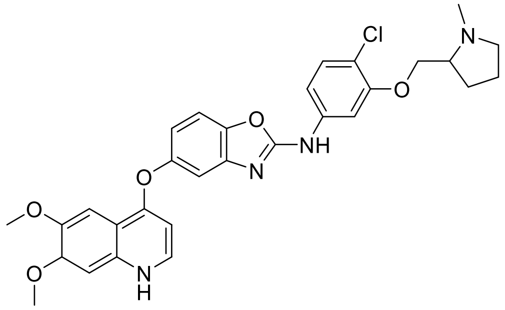	**6**	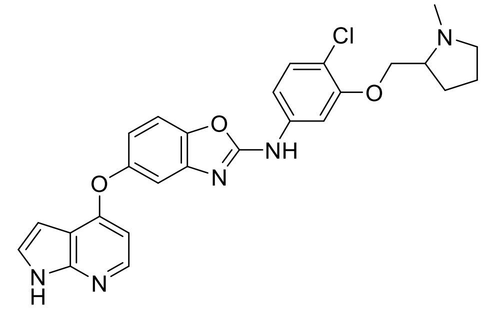
**7**	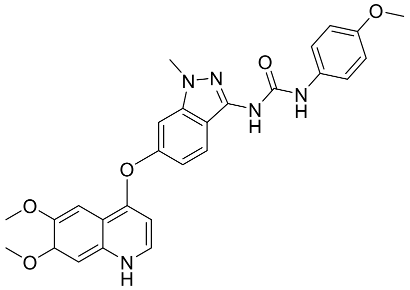	**8**	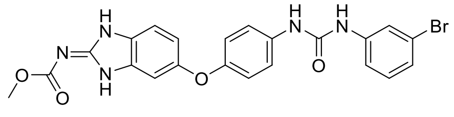
**9**	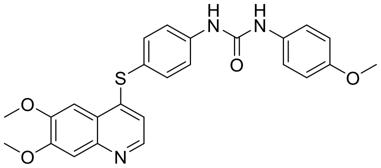	**10**	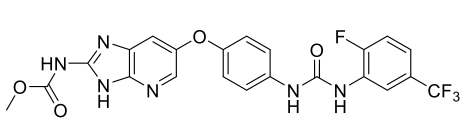

**11**	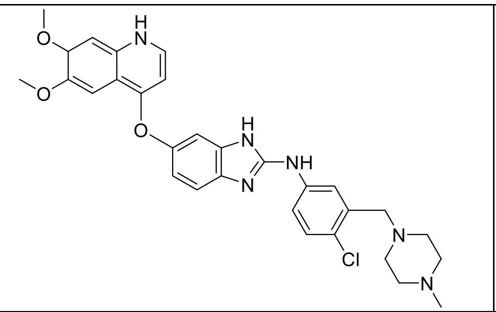	**12**	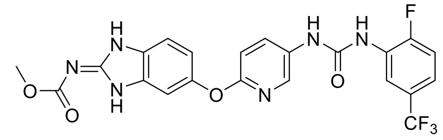
**13**	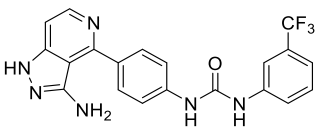	**14**	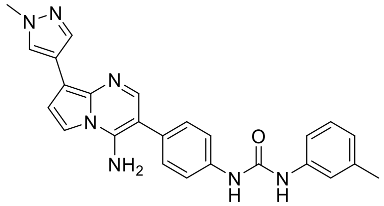
**15**	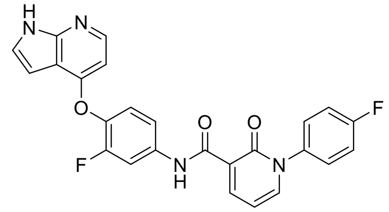	**16**	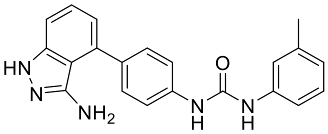
**17**	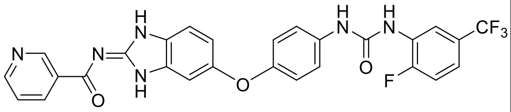	**18**	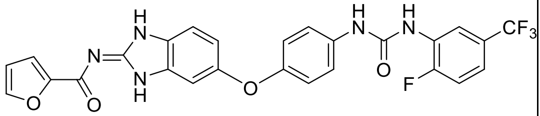
**19**	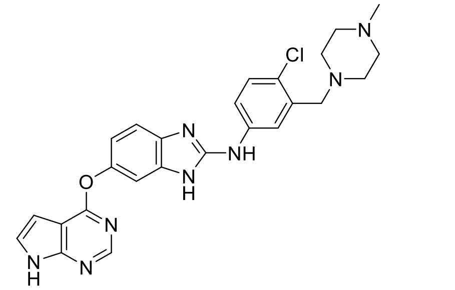	**20**	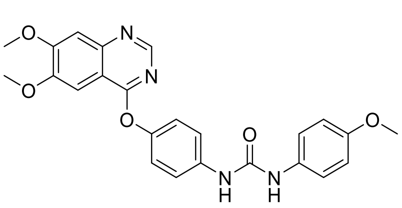
**21**	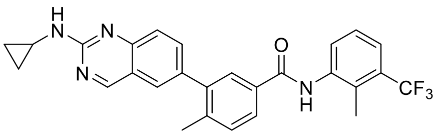	**22**	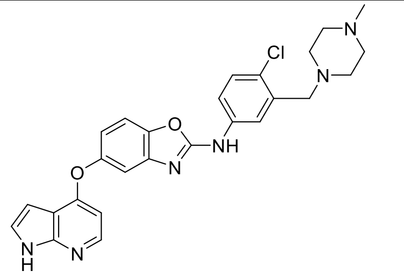
**23**	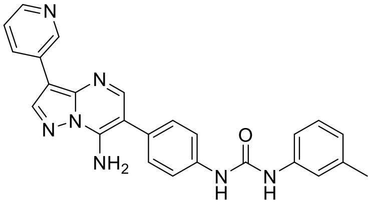	**24**	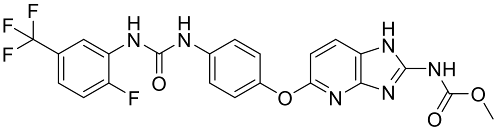

**25**	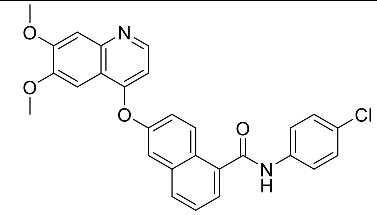	**26**	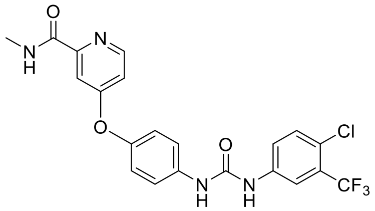

Table S5. The compiled FGFR-1 active compounds used for pharmacophore selection and validation

#	Structure	#	Structure
**1**	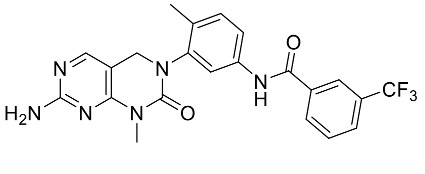	**2**	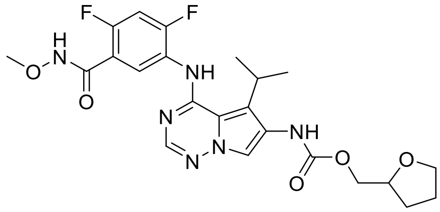
**3**	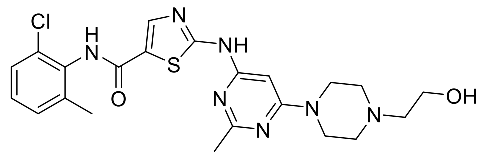	**4**	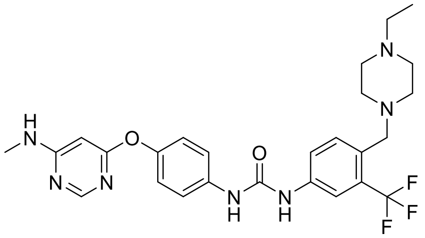
**5**	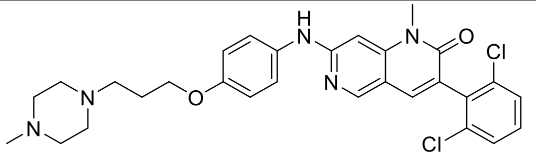	**6**	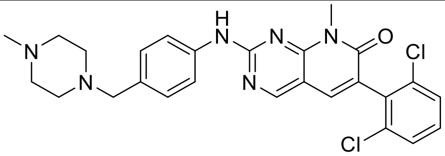
**7**	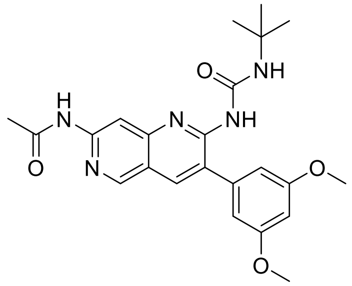	**8**	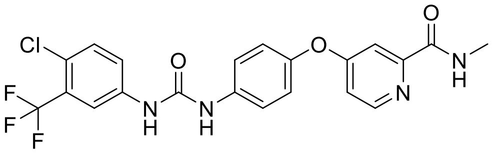
**9**	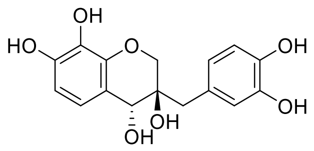	**10**	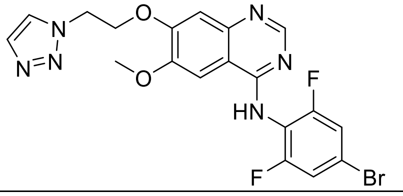

**11**	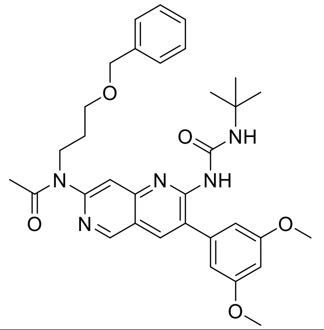	**12**	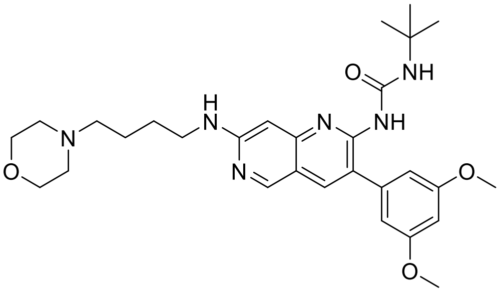
**13**	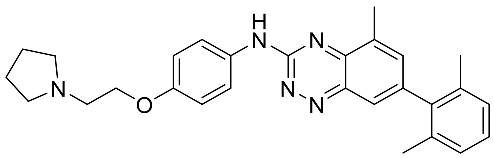	**14**	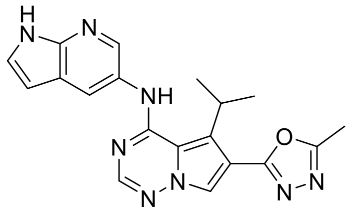
**15**	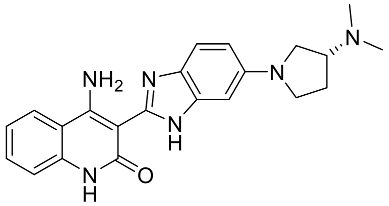	**16**	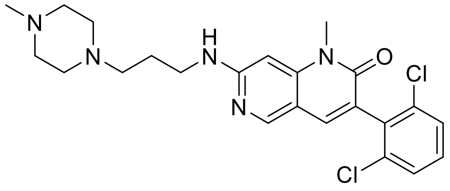
**17**	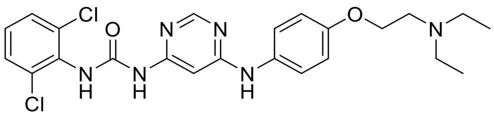	**18**	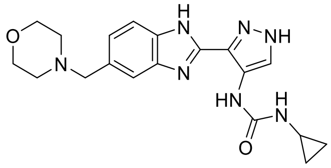
**19**	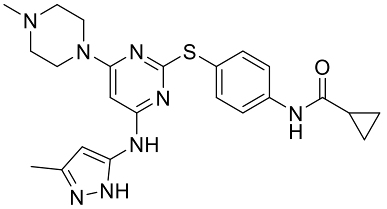	**20**	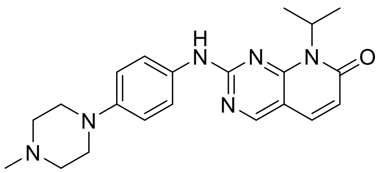

Table S6. The compiled BRAF active compounds used for pharmacophore selection and validation

#	Structure	#	Structure
**1**	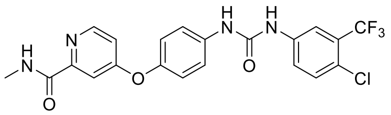	**2**	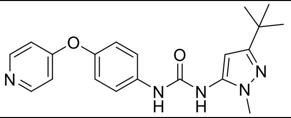
**3**	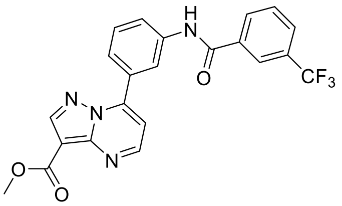	**4**	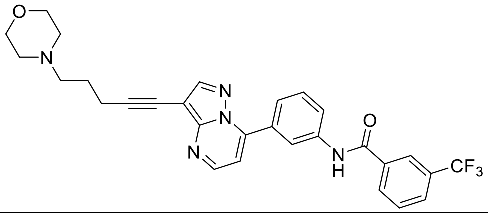

**5**	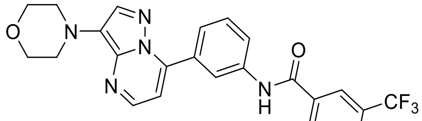	**6**	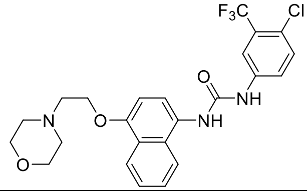
**7**	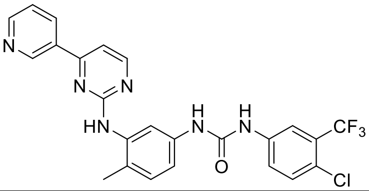	**8**	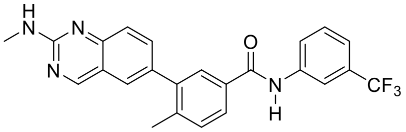
**9**	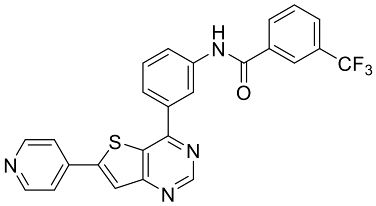	**10**	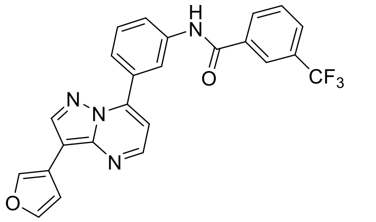
**11**	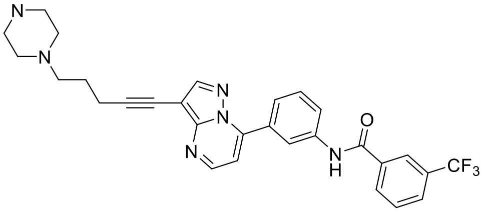	**12**	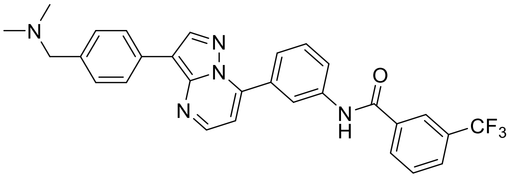
**13**	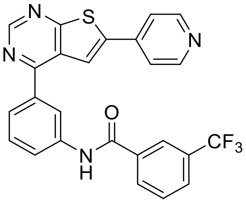	**14**	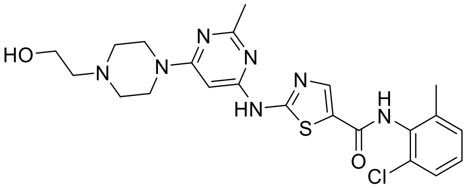
**15**	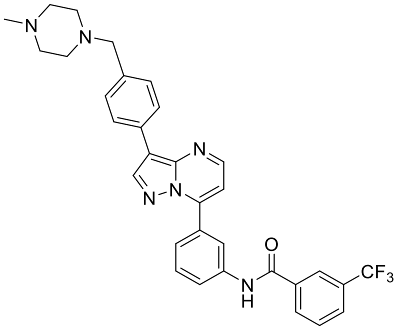	**16**	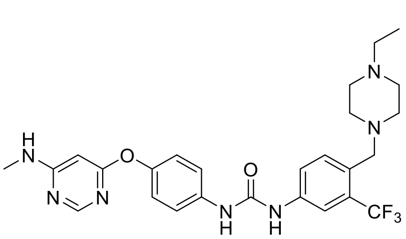
**17**	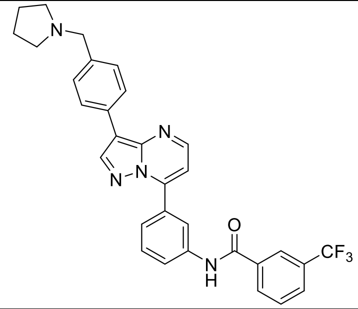	**18**	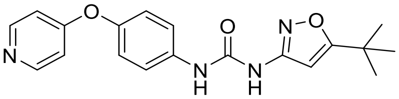

**19**	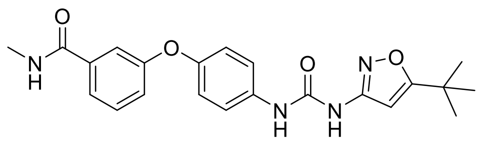	**20**	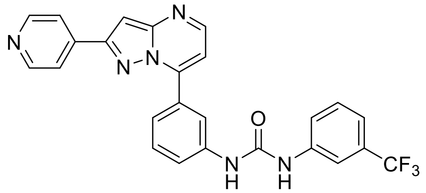
**21**	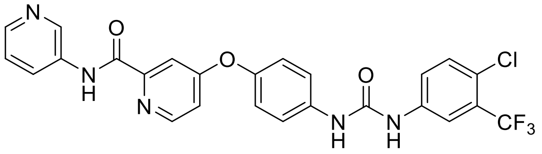	**22**	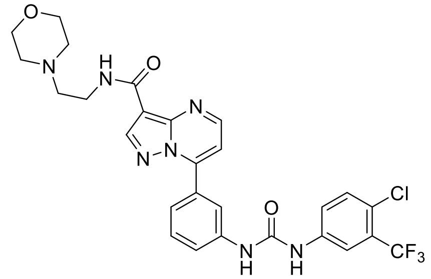
**23**	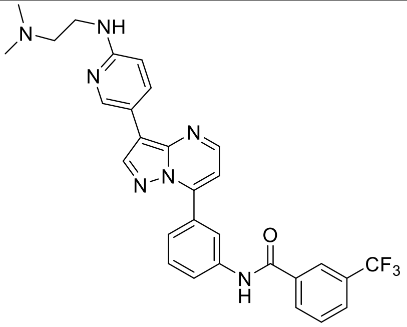	**24**	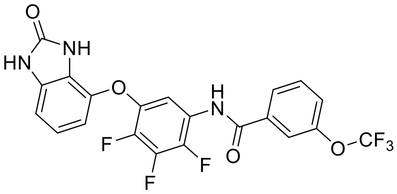
**25**	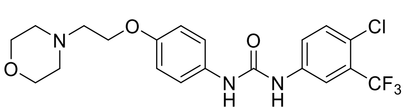	**26**	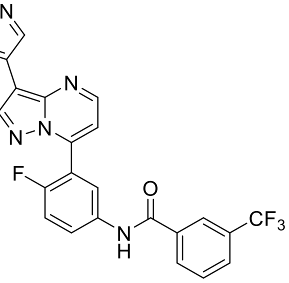
**27**	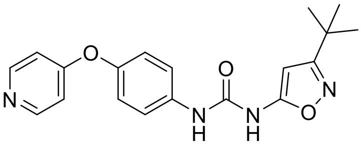		

Table S7. The discrimination parameters of the different generated pharmacophore models on the compiled test set for each target kinase VEGFR2, FGFR-1, and BRAF.

Ph4 no.	VEGFR2 test set					BRAF test set					FGFR1 test set					Total				
	n	TP	FN	TN	FP	n	TP	FN	TN	FP	n	TP	FN	TN	FP	n_t	TP_t	FP_t	FN_t	TN_t
**Ph4-1**	48	20	6	773	28	49	21	6	885	28	63	14	6	551	49	160	55	105	18	2209
**Ph4-2**	43	20	6	778	23	49	21	6	885	28	60	14	6	554	46	152	55	97	18	2217
**Ph4-3**	43	20	6	778	23	47	21	6	887	26	59	14	6	555	45	149	55	94	18	2220
**Ph4-4**	42	20	6	779	22	47	21	6	887	26	57	14	6	557	43	146	55	91	18	2223
**Ph4-5**	41	20	6	780	21	45	21	6	889	24	55	13	7	558	42	141	54	87	19	2227
**Ph4-6**	39	20	6	782	19	44	21	6	890	23	54	12	8	558	42	137	53	84	20	2230
**Ph4-7**	57	21	5	765	36	68	21	6	866	47	65	13	7	548	52	190	55	135	18	2179
**Ph4-8**	62	21	5	760	41	72	21	6	862	51	72	13	7	541	59	206	55	151	18	2163
**Ph4-9**	76	21	5	746	55	85	22	5	850	63	82	13	7	531	69	243	56	187	17	2127
**Ph4-10**	63	21	5	759	42	72	22	5	863	50	70	13	7	543	57	205	56	149	17	2165
**Ph4-11**	69	21	5	753	48	88	22	5	847	66	74	13	7	539	61	231	56	175	17	2139
**Ph4-12**	104	21	5	718	83	93	25	2	845	68	82	13	7	531	69	279	59	220	14	2094
**Ph4-13**	189	22	4	634	167	217	25	2	721	192	182	15	5	433	167	588	62	526	11	1788
**Ph4-14**	163	22	4	660	141	164	25	2	774	139	142	15	5	473	127	469	62	407	11	1907
**Ph4-15**	145	22	4	678	123	134	25	2	804	109	105	15	5	510	90	384	62	322	11	1992
**Ph4-16**	58	9	17	752	49	44	19	8	888	25	44	7	13	563	37	146	35	111	38	2203
**Ph4-17**	17	10	16	794	7	28	18	9	903	10	19	3	17	584	16	64	31	33	42	2281

Table S8. Assessment metrics of pharmacophore models performance.

Assessment metric	Equation	Insight
**Sensitivity (*Se*)**	$Se = \frac{TP}{A}$	Model ability to select correct active molecules
**Specificity (*Sp*)**	$Sp = \frac{TN}{N - A}$	The goodness of the model in discarding inactive compounds
**Yield of actives (*Ya*)**	$Ya = \frac{TP}{n}$	The hit rate
**Enrichment (*E*)**	$E = \frac{TP/n}{A/N}$	how many times the virtual screening workflow performs better than a random selection in retrieving active compounds
**Accuracy (*acc*)**	$acc = \frac{TP + TN}{N}$	the accuracy of the virtual screening workflow in discriminating between active and inactive compounds
**Discrimination ratio (*DR*)**	$DR = \frac{Se}{Sp}$	the ratio in the prediction accuracy of the active and inactive compounds of the virtual screening workflow
**F1 Score (*F1*)**	$F1 = \frac{TP}{TP + \frac{1}{2}(FP + FN)}$	Measures the overall quality of the pharmacophore model in discriminating between active and inactive compounds (0 - 1)
**Matthew's correlation coefficient (MCC)**	$MCC = \frac{(TP \times TN) - (FP \times FN)}{\sqrt{(TP + FP)(TP + FN)(TN + FP)(TN + FN)}}$	Measures of the overall quality of the pharmacophore model in discriminating between active and inactive compounds (−1 - 1)

TP is the number of true positive, **FP** is the number of false positive, **FN** is the number of false negative, **TN** is the number of true negative, **A** is the number of actives, **n** is the number of selected compounds as hits and **N** is the total number of compounds in the dataset.

3. Molecular docking simulation

3.1. Molecular docking protocol validation

For the VEGFR-2 crystal structure (PDB ID: 4ASD); water molecules were first removed, then the protein was prepared for the docking study using *Protonate 3D* protocol in MOE with default options. The co-crystalized ligand (sorafenib) was used to define the active site for docking. Triangle Matcher placement method and London dG scoring function were used for docking. Docking protocol was first validated by self-docking of the co-crystallized ligand (sorafenib) in the vicinity of the active site of the receptor giving a docking pose with an energy score (S) = −15.18 kcal/mol and an RMSD of 0.470Å from the co-crystalized ligand pose (figure 4).

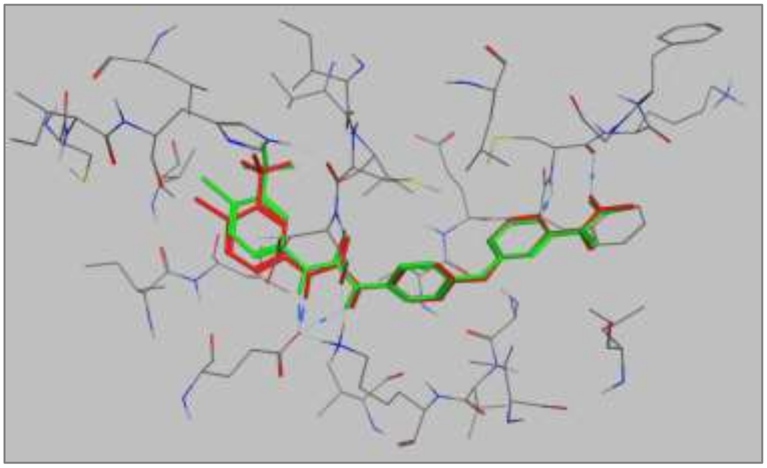

(A)

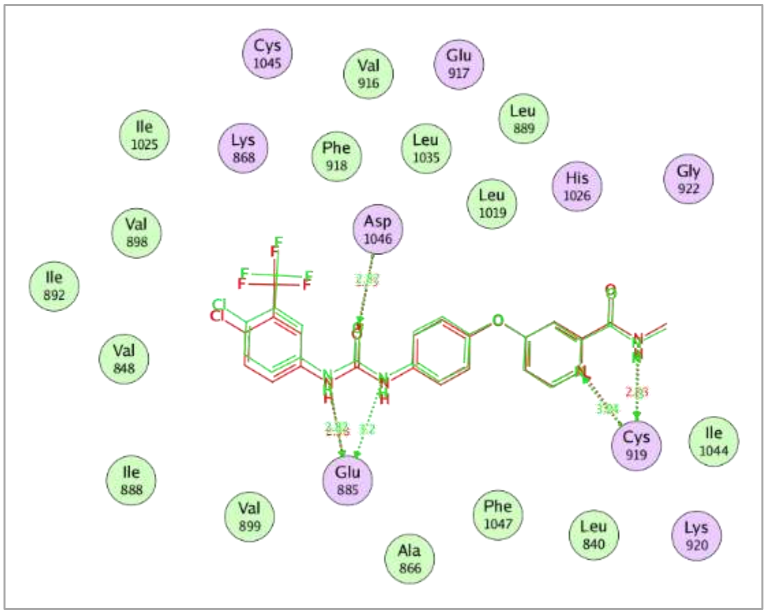

(B)

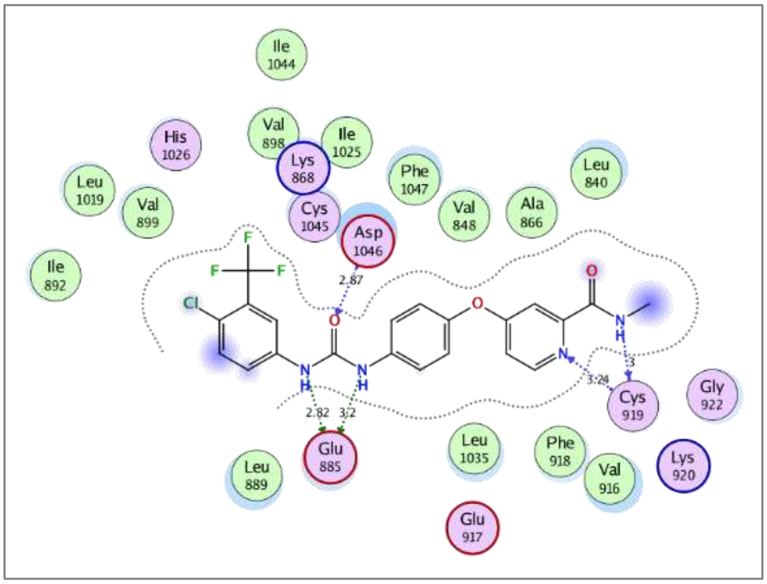

(C)

Figure S4. 2D diagram (A) and 3D representation (B) of the superimposition of docking pose (green) and the co-crystallized (red) of sorafenib in the VEGFR-2 active site with RMSD of 0.470Å. (C) 2D interaction diagram showing sorafenib docking pose interactions with the key amino acids (hot spots) in the VEGFR-2 active site. (Distances in Å)

For the FGFR-1 crystal structure (PDB ID: 4V01); chain A, water molecules and ligands which are not involved in binding were first removed, then the protein was prepared for the docking study using *Protonate 3D* protocol in MOE with default options. The co-crystalized ligand (ponatinib) was used to define the active site for docking. Triangle Matcher placement method and London dG scoring function were used for docking. Docking protocol was first validated by self-docking of the co-crystallized ligand (ponatinib) in the vicinity of the active site of the receptor giving a docking pose with an energy score (S) = −17.00 kcal/mol and an RMSD of 0.398Å from the co-crystalized ligand pose (figure 5).

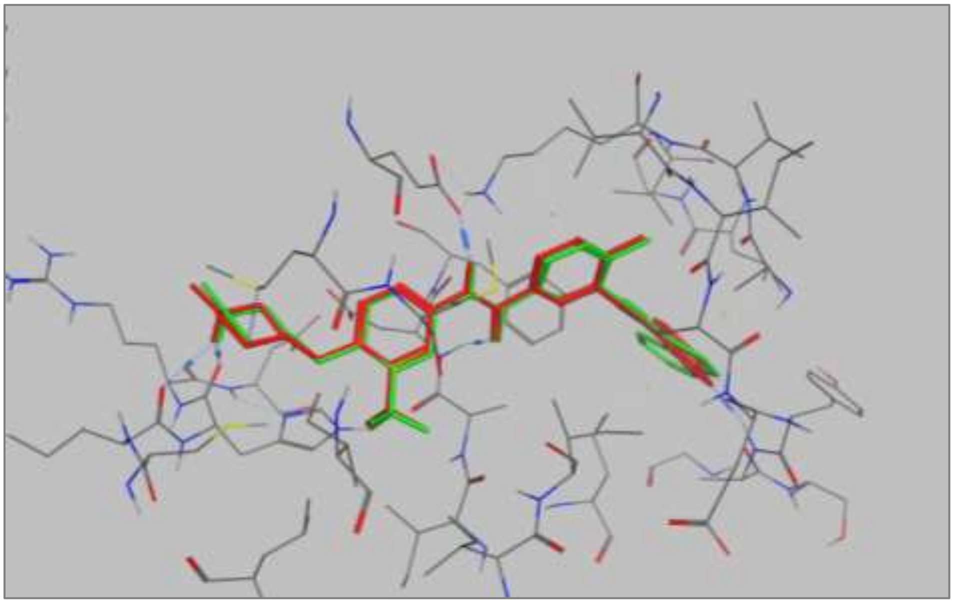

(A)

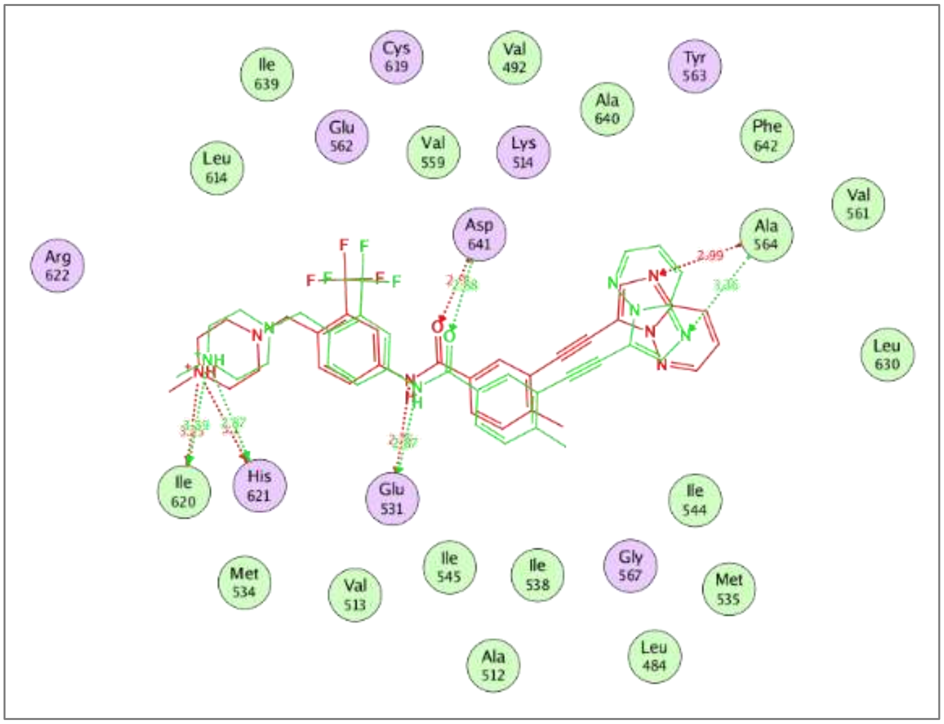

Ile 639
Cys 619
Val 492
Tyr 563
Ala 640
Phe 642
Leu 614
Glu 562
Val 559
Lys 514
Val 561
Asp 641
Ala 564
Arg 622
2.99
Leu 630
Ile 620
His 621
Glu 531
Ile 544
Met 534
Val 513
Ile 545
Ile 538
Gly 567
Met 535
Ala 512
Leu 484

(B)

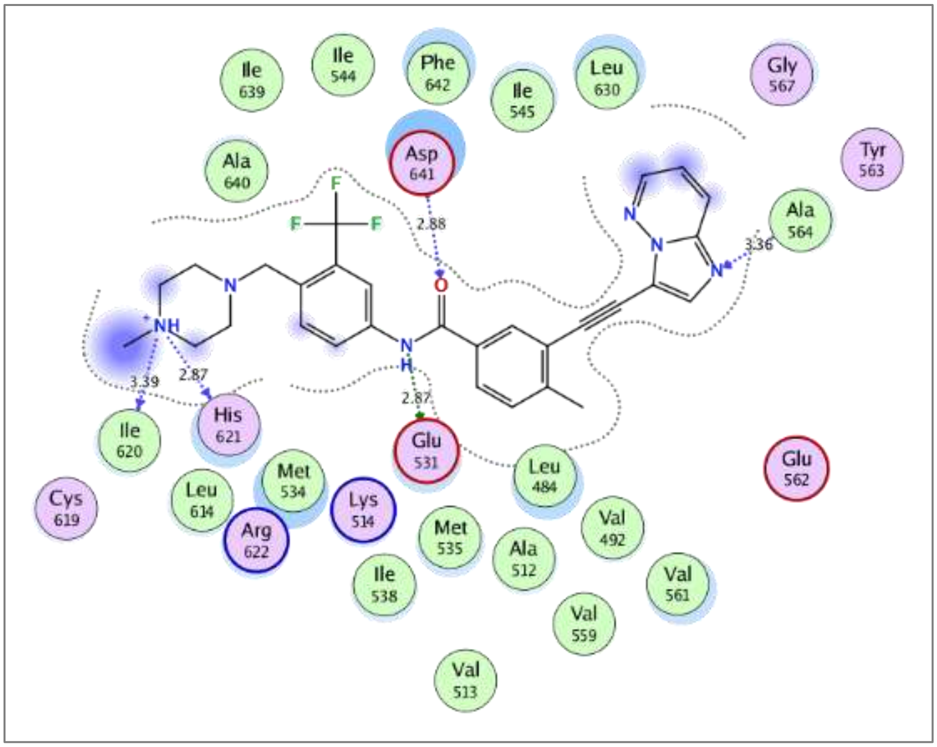

(C)

Figure S5. 2D diagram (A) and 3D representation (B) of the superimposition of docking pose (green) and the co-crystallized (red) of ponatinib in the FGFR-1 active site with RMSD of 0.398Å. (C) 2D interaction diagram showing ponatinib docking pose interactions with the key amino acids (hot spots) in the FGFR-1 active site. (Distances in Å)

For BRAF crystal structure (PDB ID: 5CT7), Chain B was first removed. Then, protein structure was prepared for the docking study using *Protonate 3D* protocol in MOE with default options. The co-crystalized ligand, RAF265, was used to define the active site in the protein structure for docking. Triangle Matcher placement method and London dG scoring function were used for docking. Docking protocol was first validated by re-docking of the co-crystallized ligand in the vicinity of the active site giving a docking pose with an energy score (S) = −15.82 kcal/mol and an RMSD of 0.419Å from the co-crystalized ligand pose (figure 6).

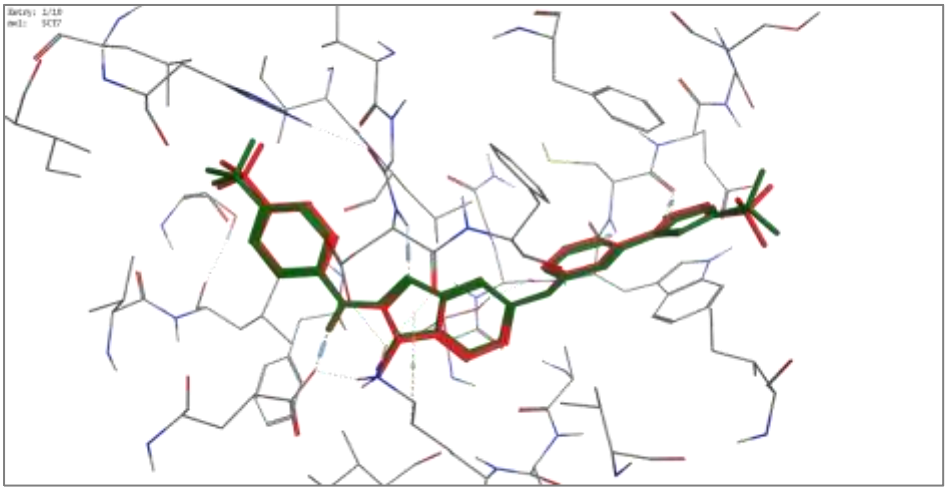

(A)

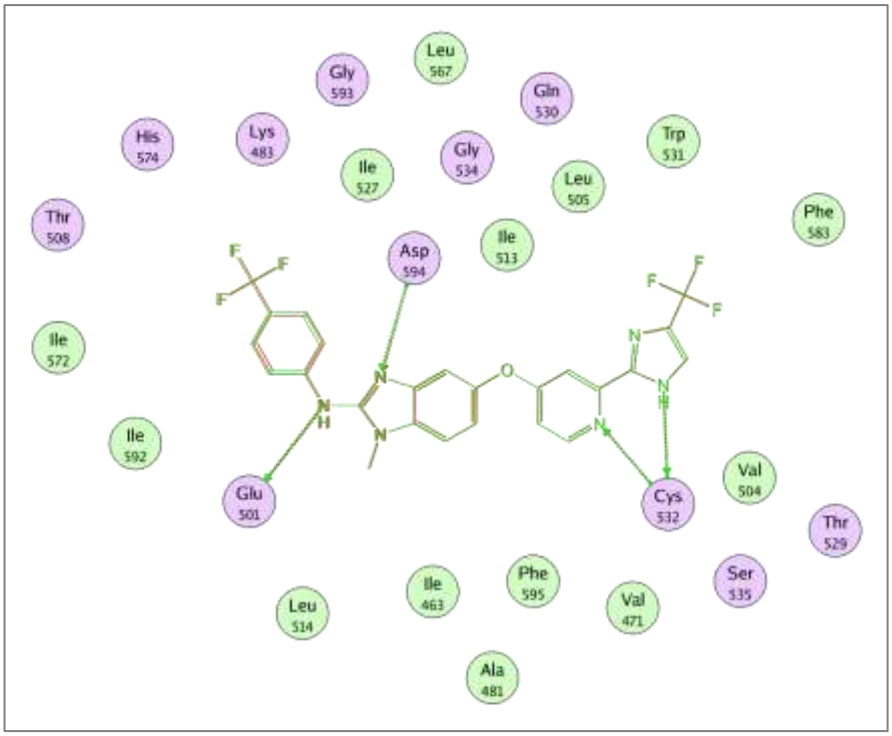
Leu 567
Gly 593
Gln 530
His 574
Lys 483
Trp 531
Ile 527
Gly 534
Leu 505
Thr 508
Ile 513
Phe 583
Asp 594
Ile 572
Ile 592
Val 504
Glu 501
Cys 532
Thr 529
Ser 535
Leu 514
Ile 463
Phe 595
Val 471
Ala 481
F
F
F
N
H
N
N
O
N
N
N
F
F
F

(B)

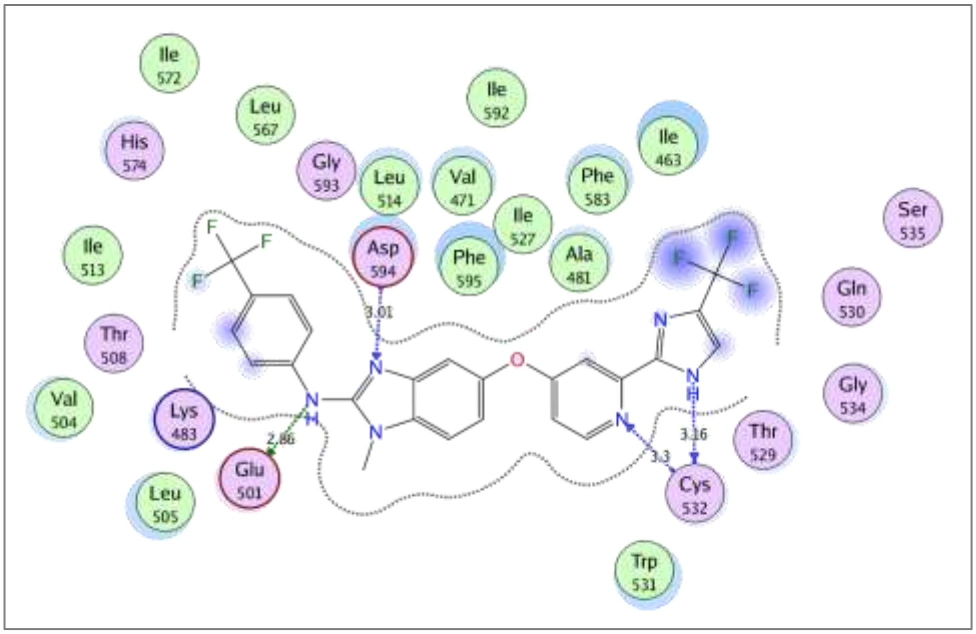

(C)

Figure S6. 2D diagram (A) and 3D representation (B) of the superimposition of docking pose (green) and the co-crystallized (red) of RAF265 in the BRAF active site with RMSD of 0.419Å. (C) 2D interaction diagram showing RAF265 docking pose interactions with the key amino acids (hot spots) in the BRAF active site. (Distances in Å)

3.2. Molecular docking of the target compounds 8a-u

Table S9. Docking energy scores (*S*) in kcal/mol for the newly synthesized compounds and the reference compounds in VEGFR-2, BRAF and FGFR-1 active sites

Compound	Energy score (S) kcal/mol VEGFR-2	Energy score (S) kcal/mol FGFR-1	Energy score (S) kcal/mol BRAF
**8a**	−13.55	−12.11	−11.60
**8b**	−13.98	−11.62	−11.49
**8c**	−12.73	−12.20	−11.52
**8d**	−14.13	−13.04	−11.69
**8e**	−13.25	−13.45	−12.62
**8f**	−14.35	−12.65	−13.13
**8g**	−13.81	−13.87	−11.85
**8h**	−14.12	−12.88	−13.26
**8i**	−14.52	−13.51	−12.23
**8j**	−13.52	−12.85	−13.19
**8k**	−14.62	−12.98	−13.65
**8l**	−13.63	−12.77	−13.38
**8m**	−14.89	−13.39	−12.90
**8n**	−14.49	−13.31	−13.07
**8o**	−13.88	−12.53	−13.21
**8p**	−14.30	−12.33	−12.78
**8q**	−13.08	−12.18	−11.86
**8r**	−14.21	−13.67	−13.40
**8s**	−14.77	−12.68	−13.08
**8t**	−14.64	−14.18	−12.74
**8u**	−14.16	−13.07	−12.66
**Co-crystalized ligand**	−15.19	−17.00	−15.82

Docking poses of the designed compounds, in VEGFR-2 active site (Figure S7-27)

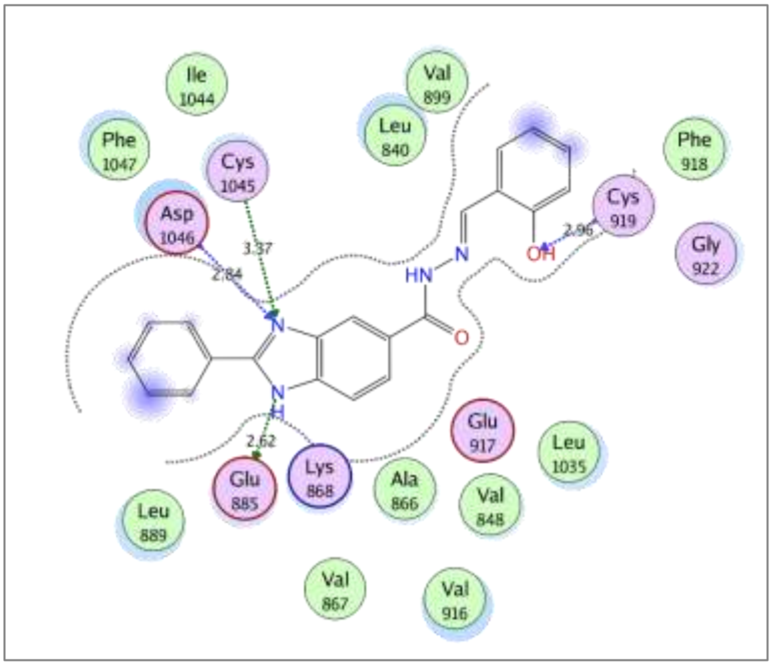

Figure S7. 2D diagram of compound **8a** showing its interaction with the VEGFR-2 active site

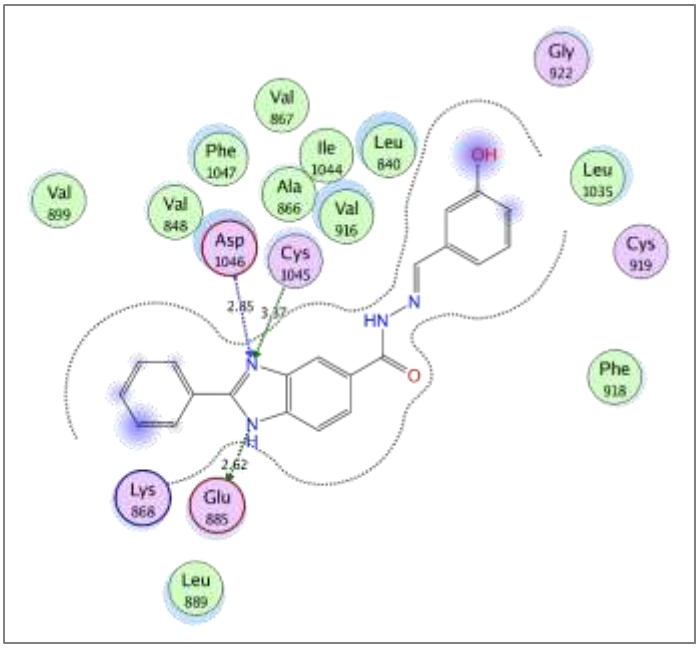

Figure S8. 2D diagram of compound **8b** showing its interaction with the VEGFR-2 active site

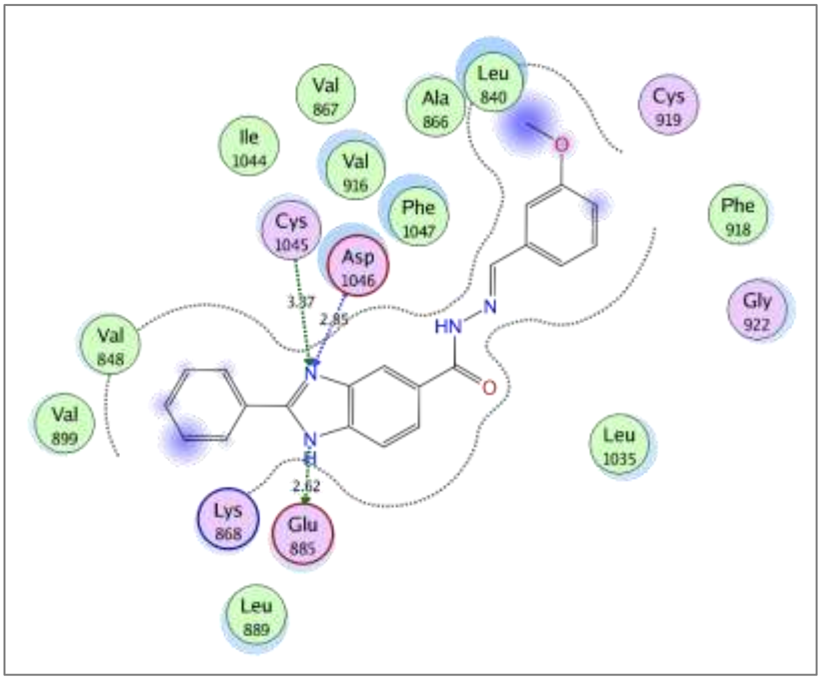

Figure S9. 2D diagram of compound **8c** showing its interaction with the VEGFR-2 active site

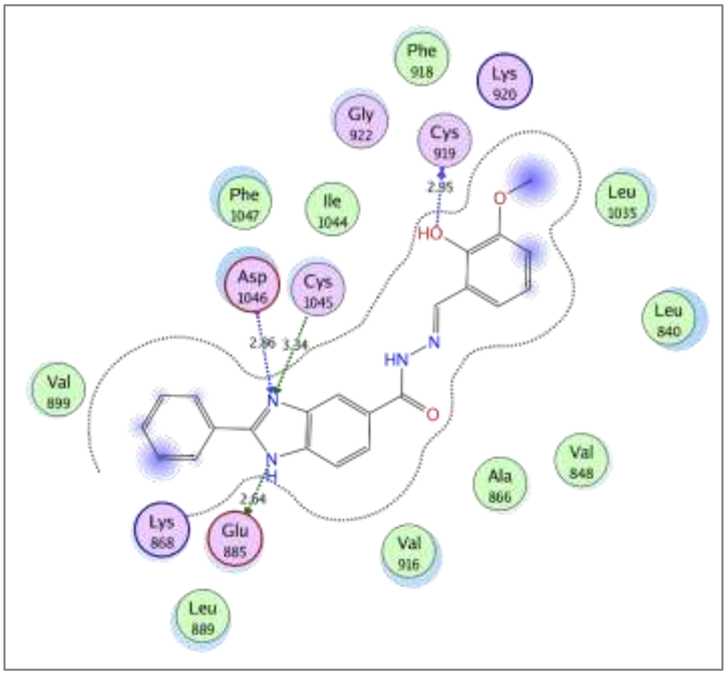

Figure S10. 2D diagram of compound **8d** showing its interaction with the VEGFR-2 active site

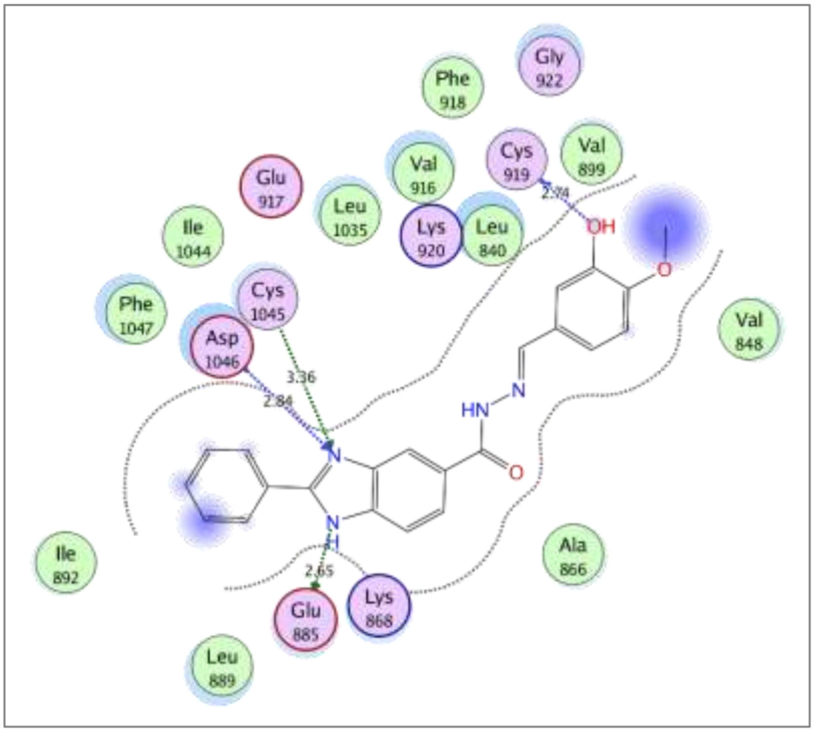

Figure S11. 2D diagram of compound **8e** showing its interaction with the VEGFR-2 active site

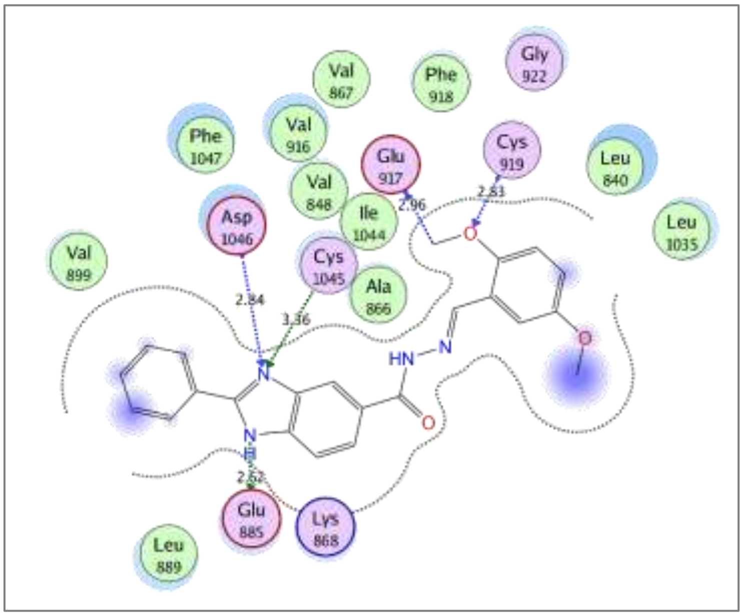

Figure S12. 2D diagram of compound **8f** showing its interaction with the VEGFR-2 active site

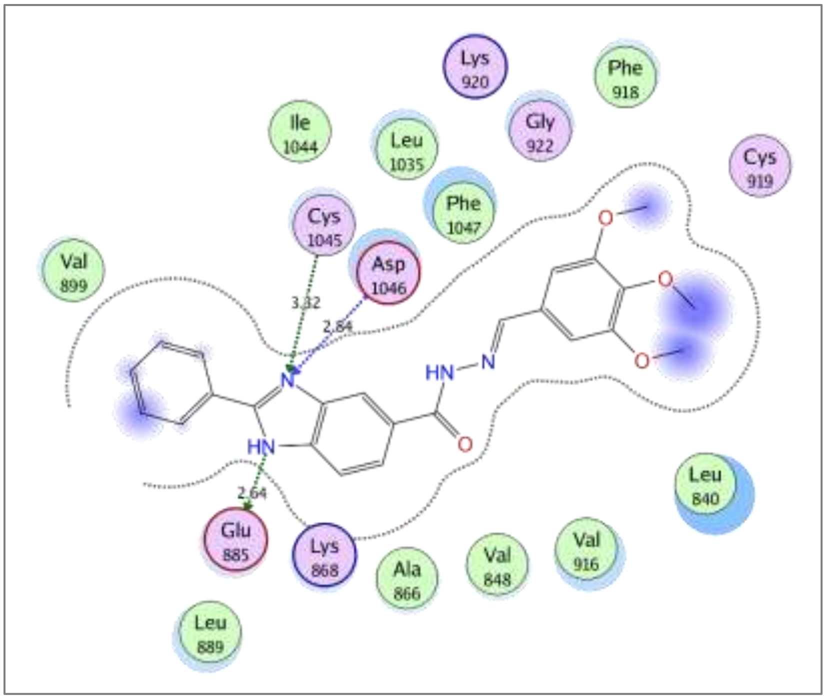

Figure S13. 2D diagram of compound **8g** showing its interaction with the VEGFR-2 active site

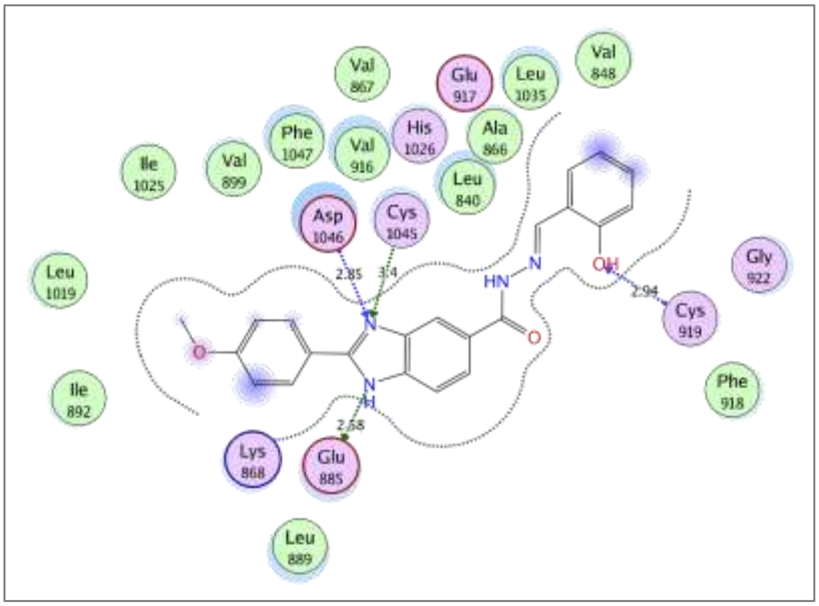

Figure S14. 2D diagram of compound **8h** showing its interaction with the VEGFR-2 active site

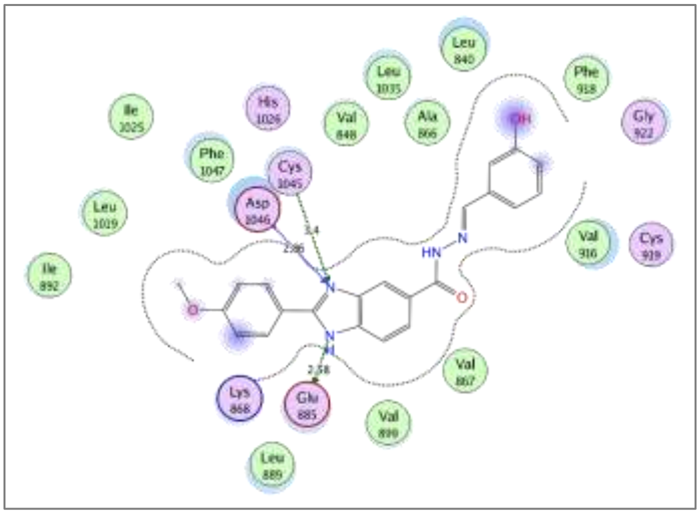

Figure S15. 2D diagram of compound **8i** showing its interaction with the VEGFR-2 active site

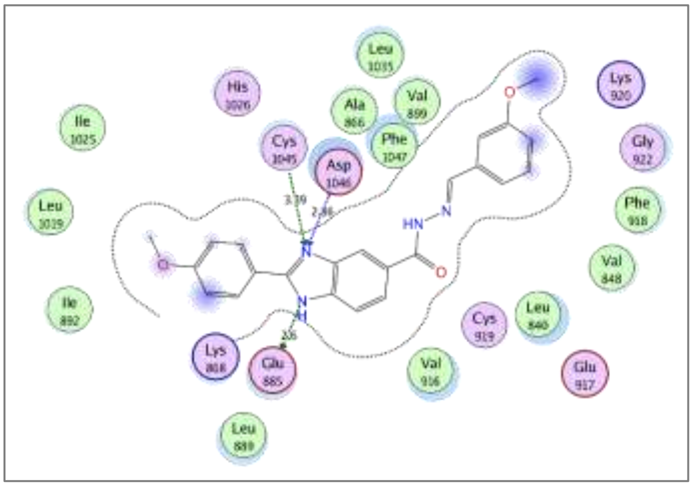

Figure S16. 2D diagram of compound **8j** showing its interaction with the VEGFR-2 active site

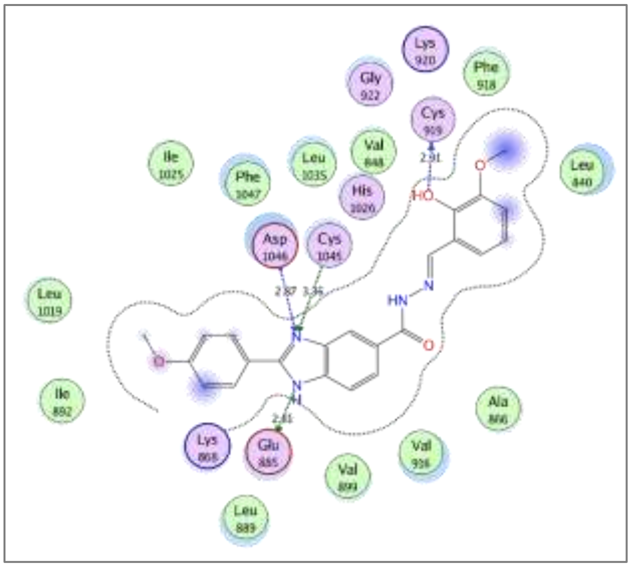

Figure S17. 2D diagram of compound **8k** showing its interaction with the VEGFR-2 active site

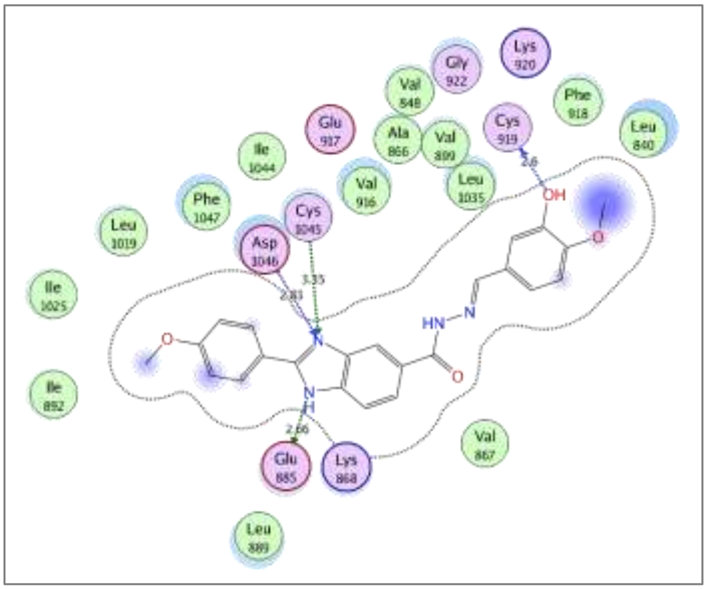

Figure S18. 2D diagram of compound **8l** showing its interaction with the VEGFR-2 active site.

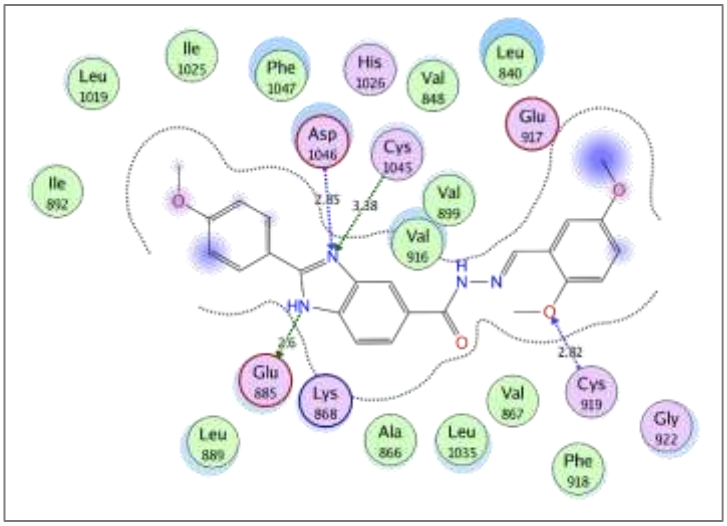

Figure S19. 2D diagram of compound **8m** showing its interaction with the VEGFR-2 active site

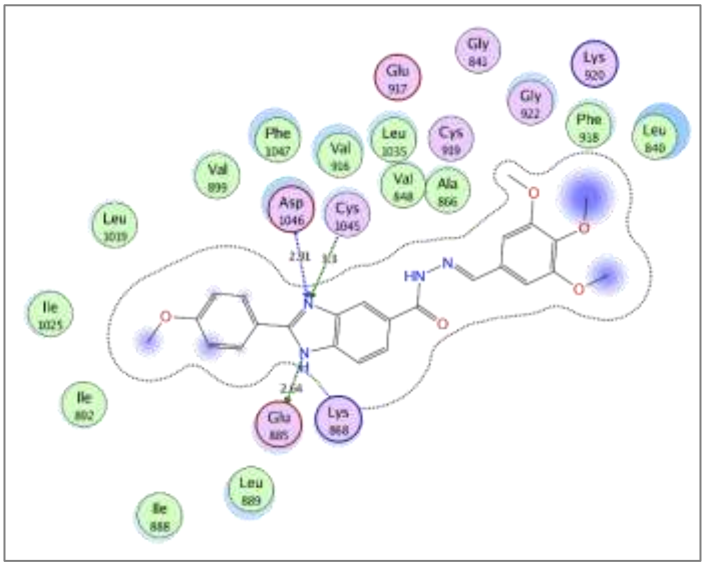

Figure S20. 2D diagram of compound **8n** showing its interaction with the VEGFR-2 active site

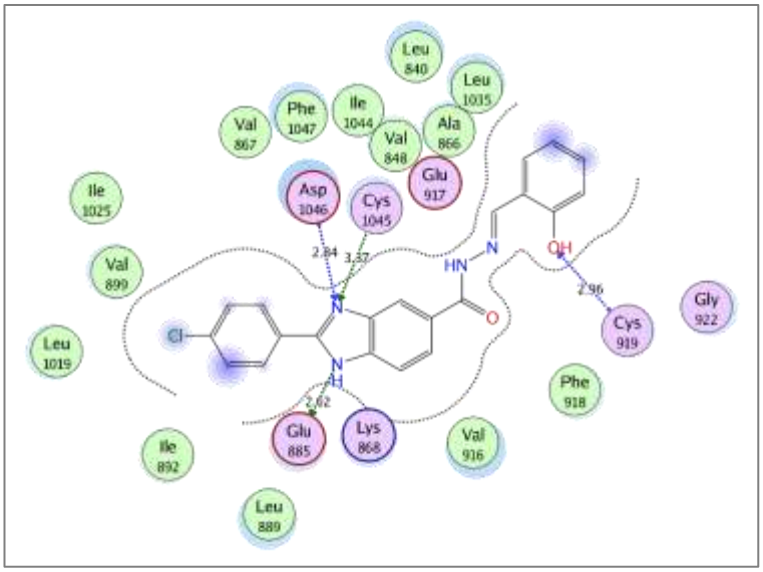

Figure S21. 2D diagram of compound **8o** showing its interaction with the VEGFR-2 active site

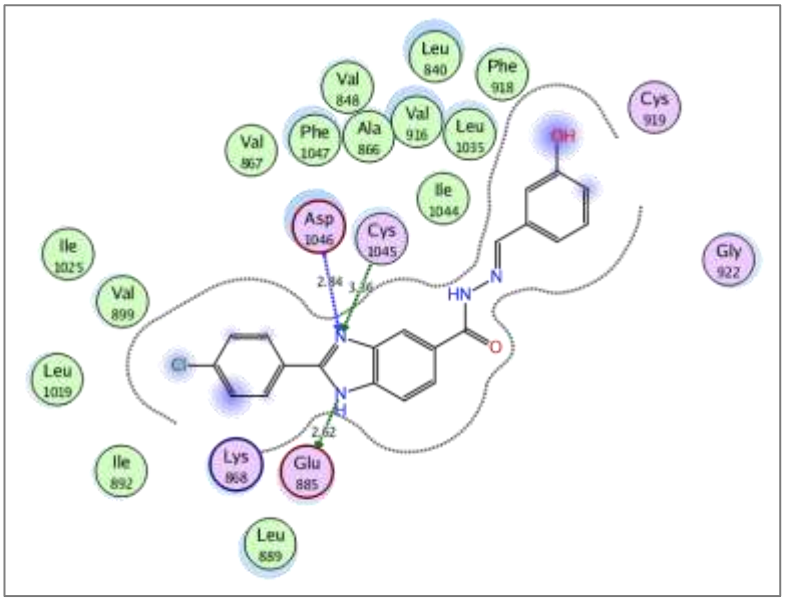

Figure S22. 2D diagram of compound **8p** showing its interaction with the VEGFR-2 active site

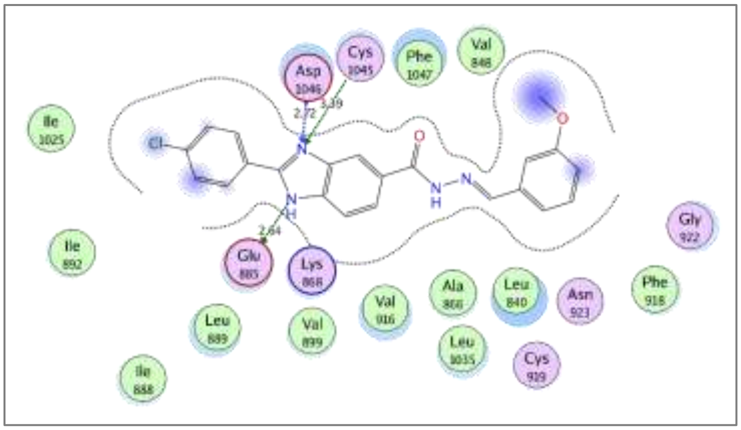

Figure S23. 2D diagram of compound **8q** showing its interaction with the VEGFR-2 active site

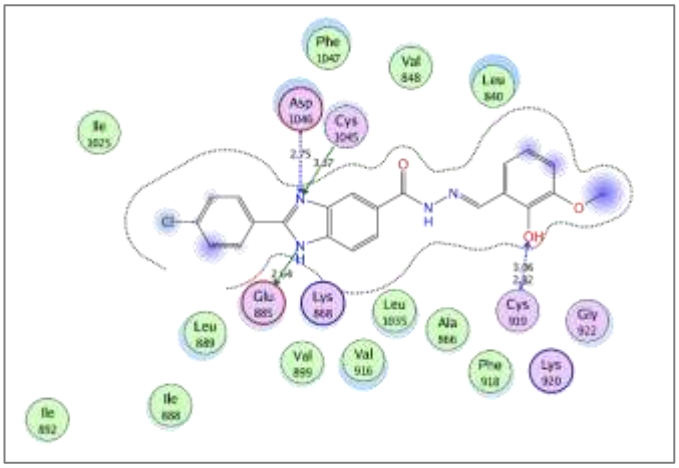

Figure S24. 2D diagram of compound **8r** showing its interaction with the VEGFR-2 active site

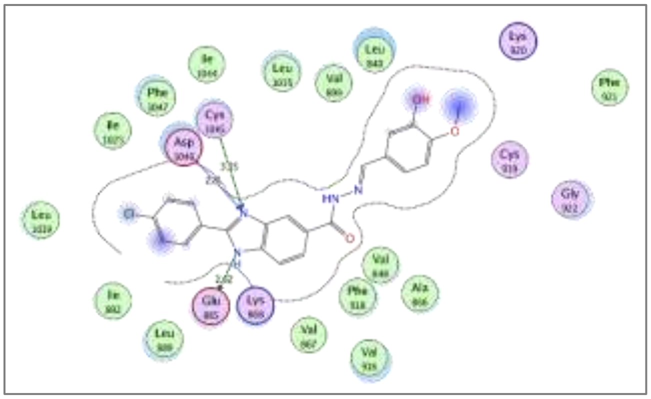

Figure S25. 2D diagram of compound **8s** showing its interaction with the VEGFR-2 active site

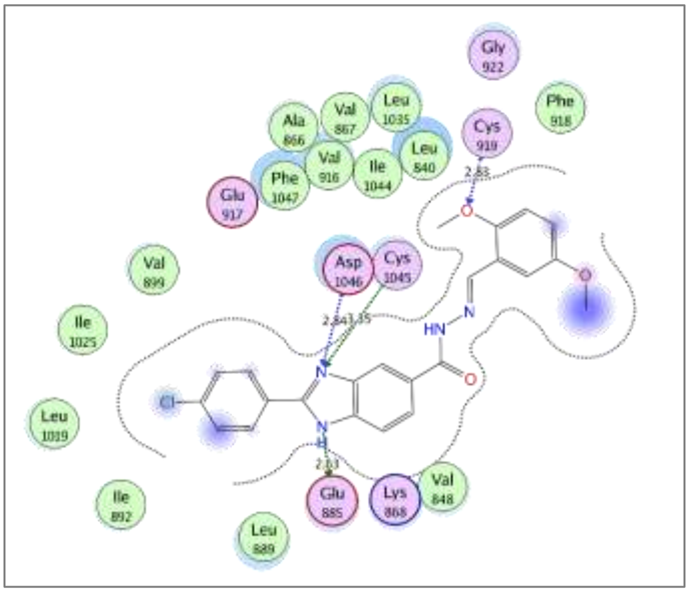

Figure S26. 2D diagram of compound **8t** showing its interaction with the VEGFR-2 active site

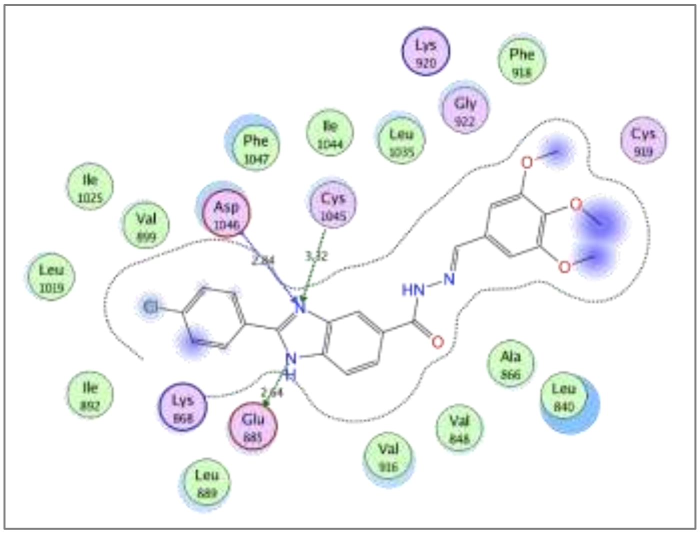

Figure S27. 2D diagram of compound **8u** showing its interaction with the VEGFR-2 active site

Docking poses of the designed compounds in FGFR-1 active site (Figure S28-48)

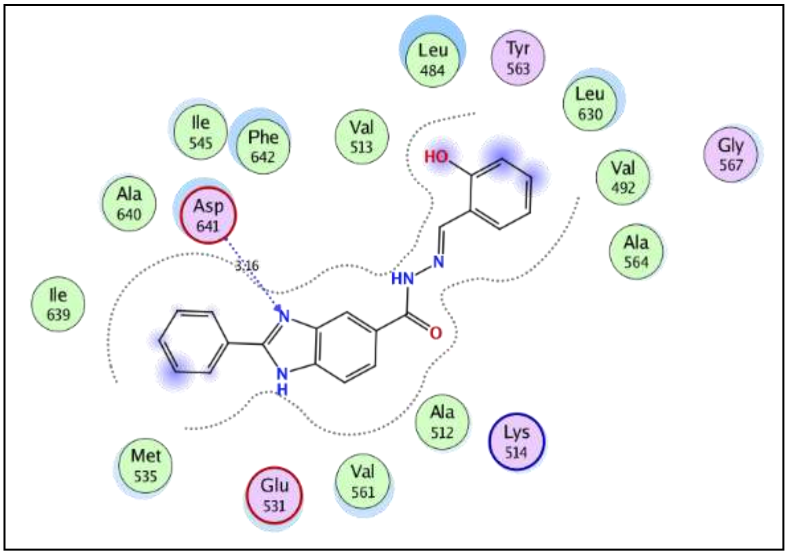

Figure S28. 2D diagram of compound **8a** showing its interaction with the FGFR-1 active site

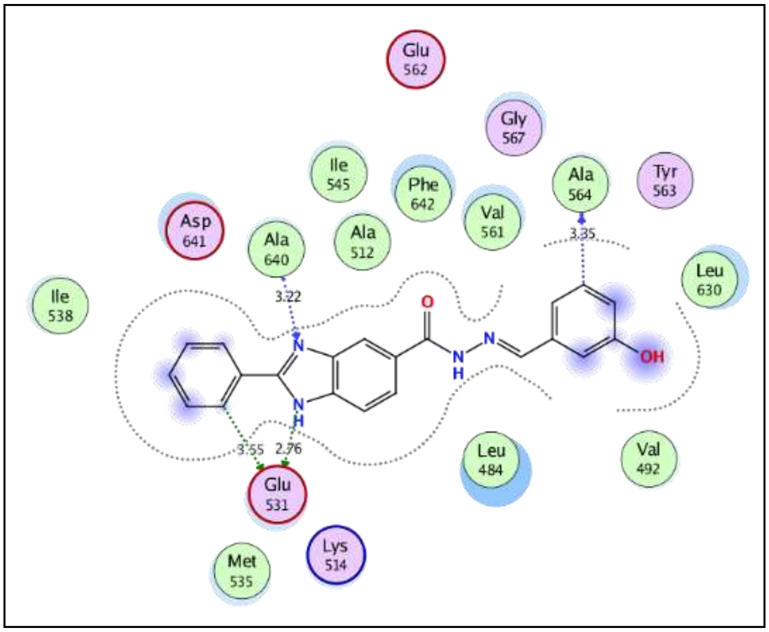

Figure S29. 2D diagram of compound **8b** showing its interaction with the FGFR-1 active site

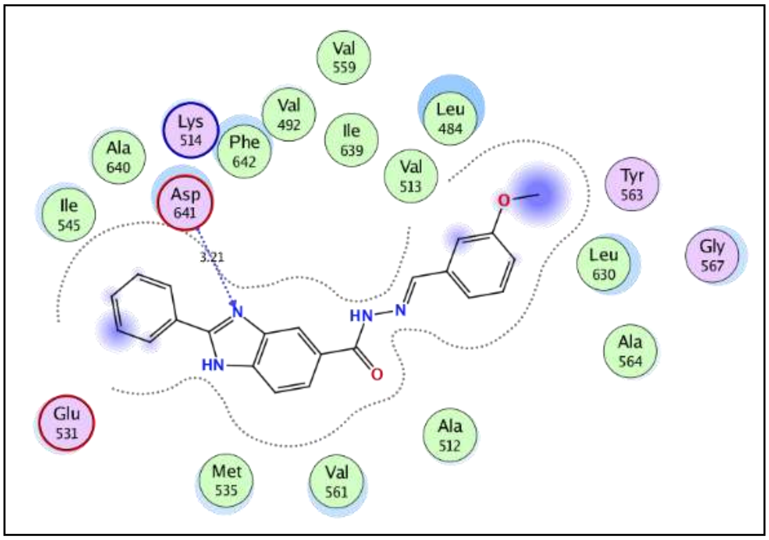

Figure S30. 2D diagram of compound **8c** showing its interaction with the FGFR-1 active site

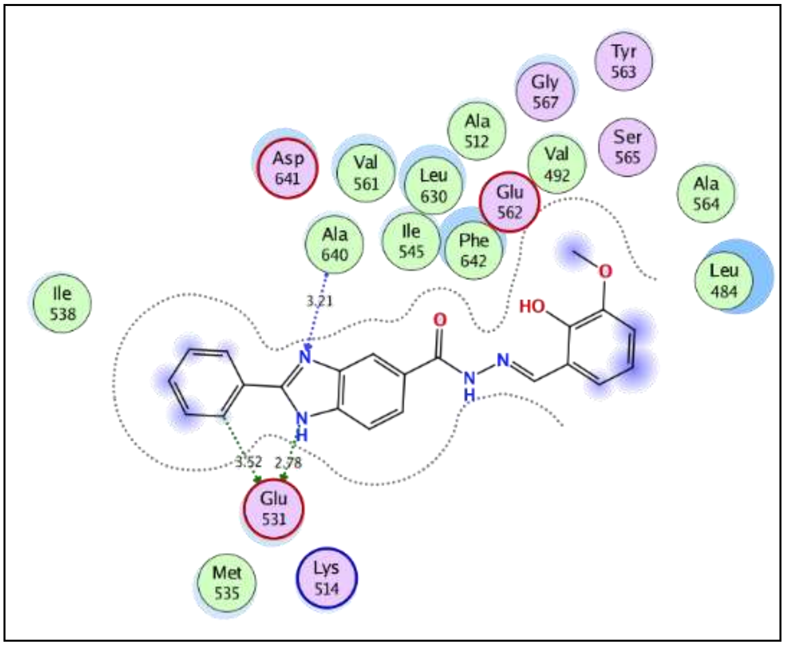

Figure S31. 2D diagram of compound **8d** showing its interaction with the FGFR-1 active site

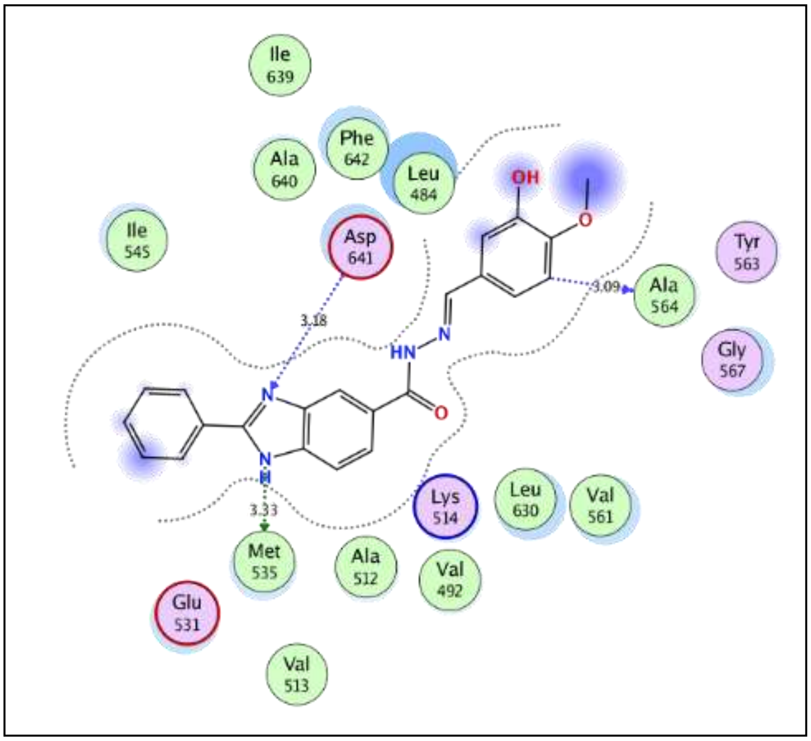

Figure S32. 2D diagram of compound **8e** showing its interaction with the FGFR-1 active site

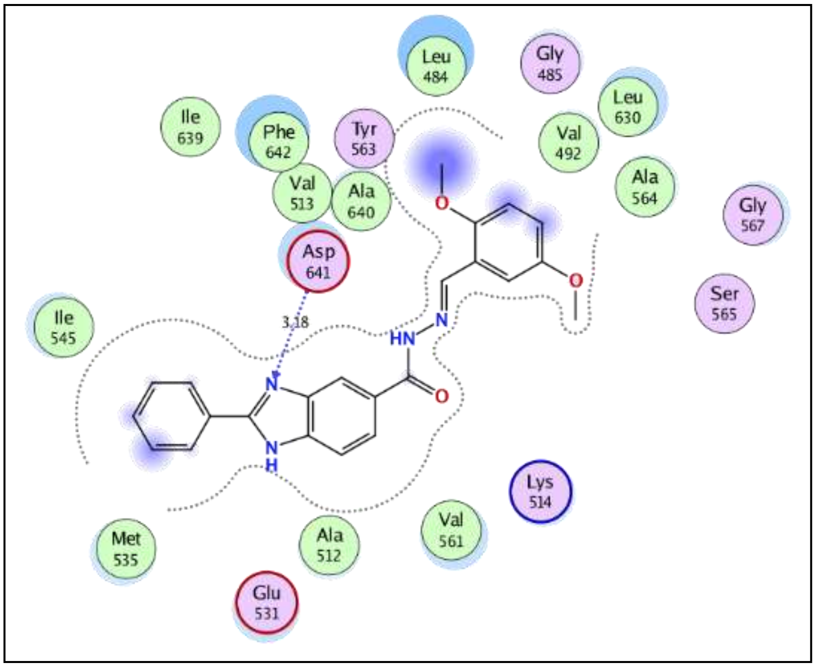

Figure S33. 2D diagram of compound **8f** showing its interaction with the FGFR-1 active site

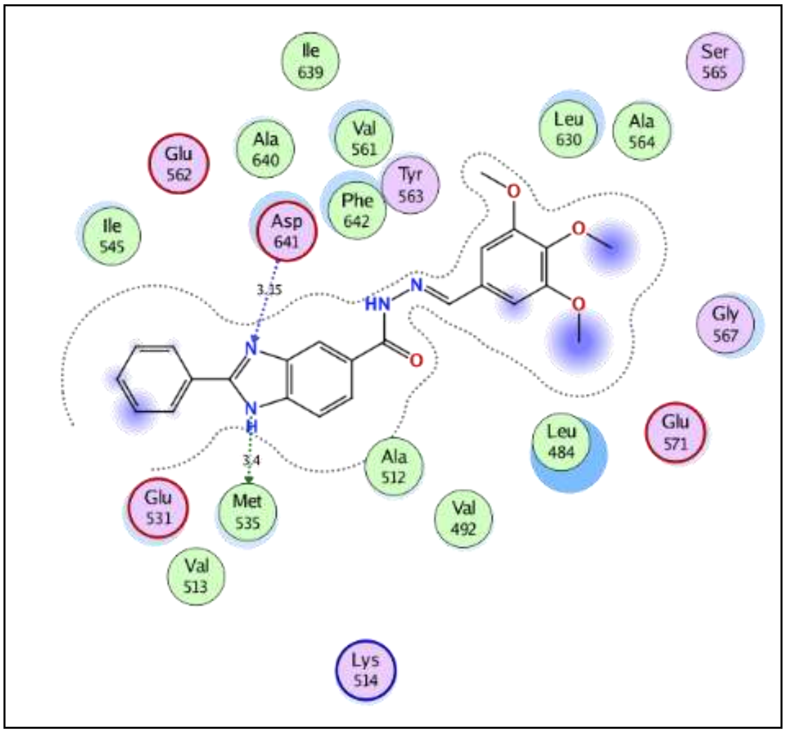

Figure S34. 2D diagram of compound **8g** showing its interaction with the FGFR-1 active site

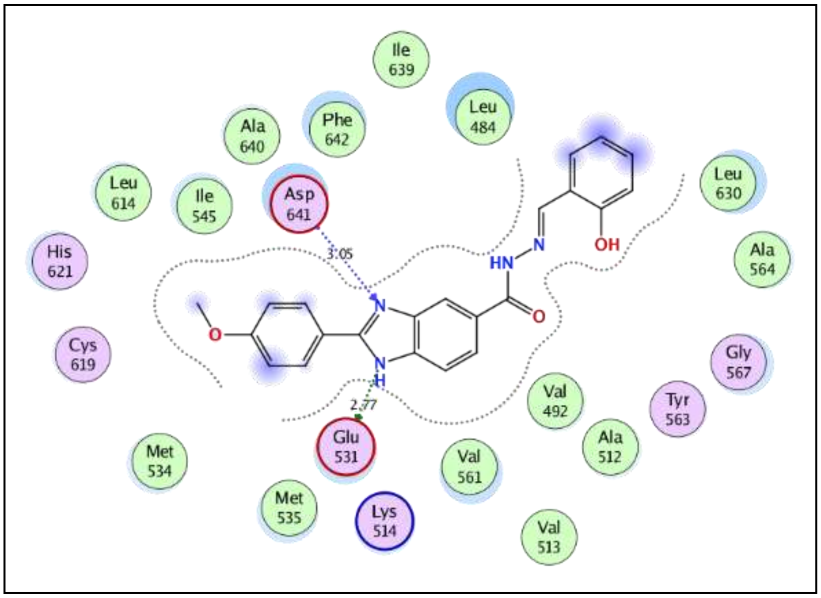

Figure S35. 2D diagram of compound **8h** showing its interaction with the FGFR-1 active site

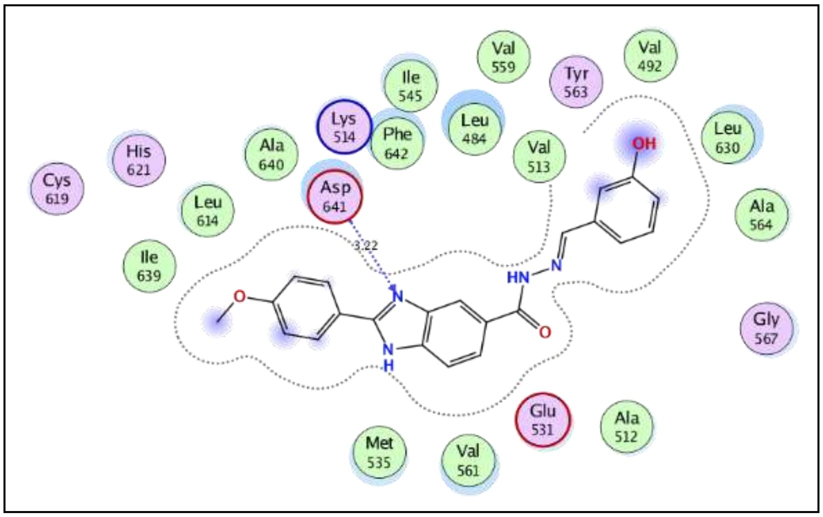

Figure S36. 2D diagram of compound **8i** showing its interaction with the FGFR-1 active site

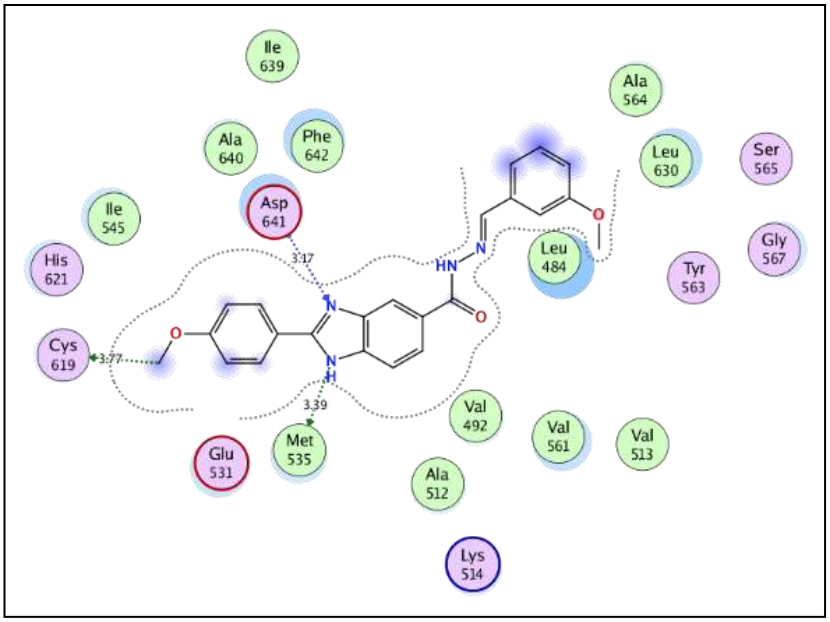

Figure S37. 2D diagram of compound **8j** showing its interaction with the FGFR-1 active site

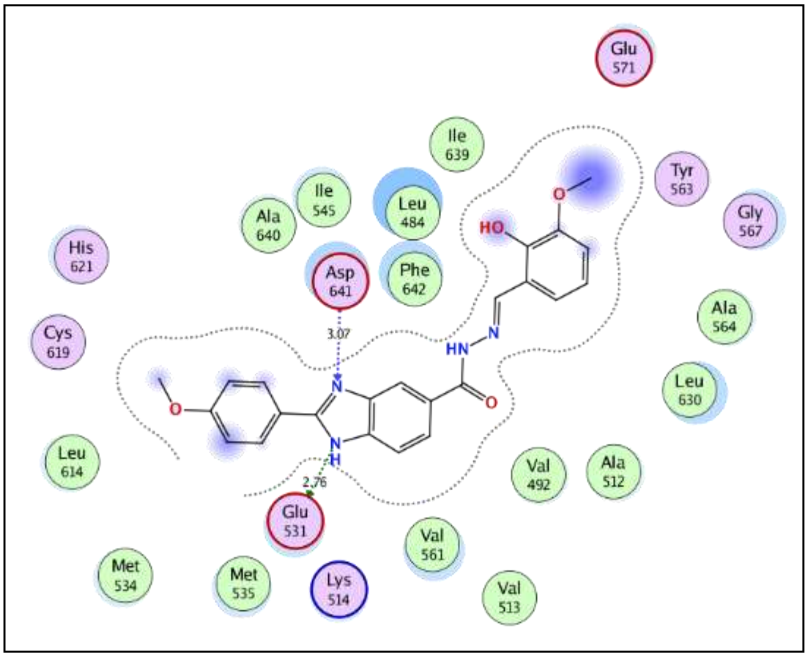

Figure S38. 2D diagram of compound **8k** showing its interaction with the FGFR-1 active site

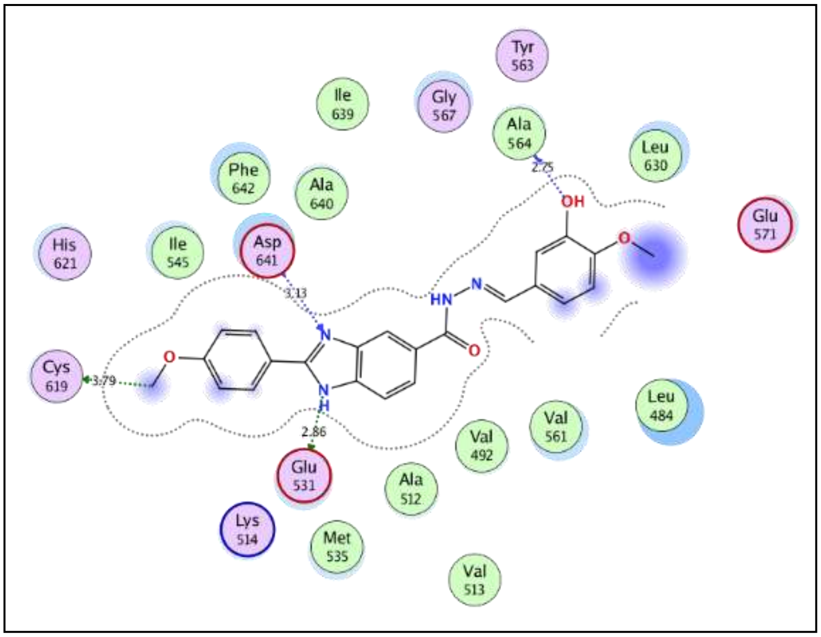

Figure S39. 2D diagram of compound **8l** showing its interaction with the FGFR-1 active site

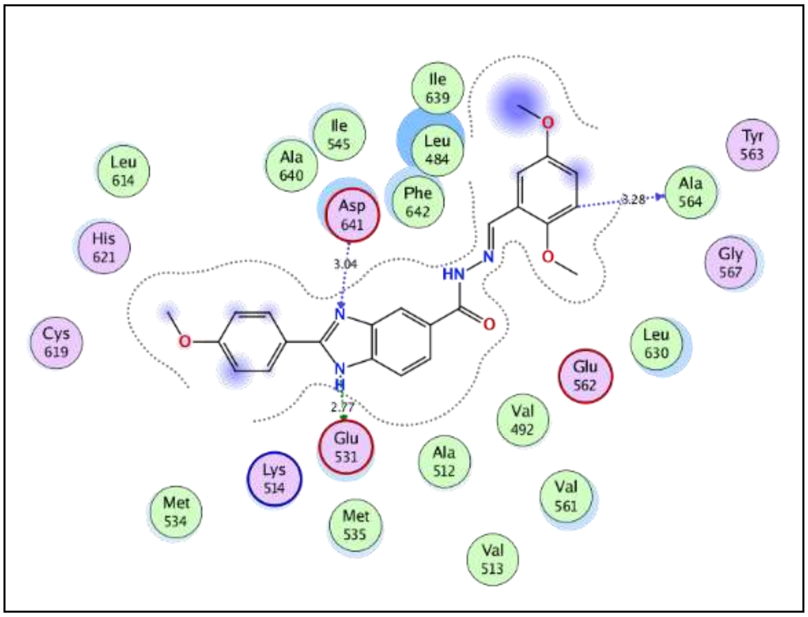

Figure S40. 2D diagram of compound **8m** showing its interaction with the FGFR-1 active site

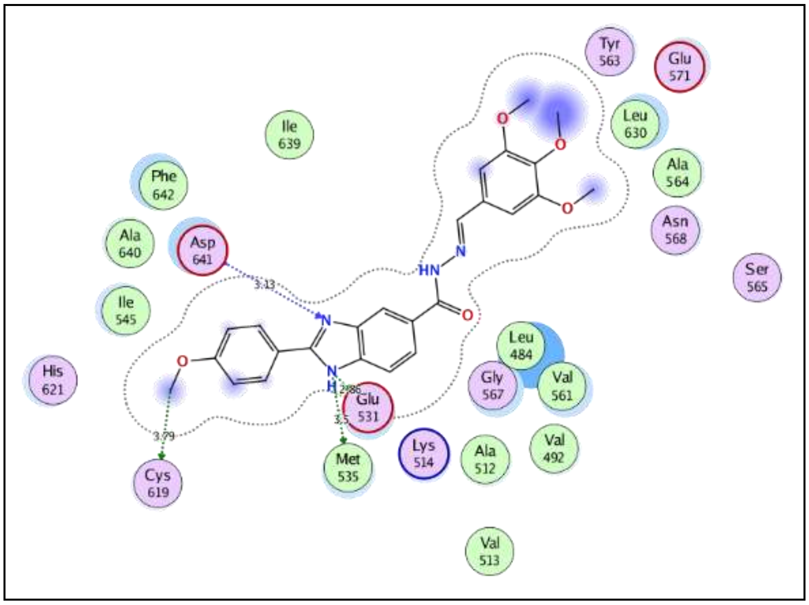

Figure S41. 2D diagram of compound **8n** showing its interaction with the FGFR-1 active site

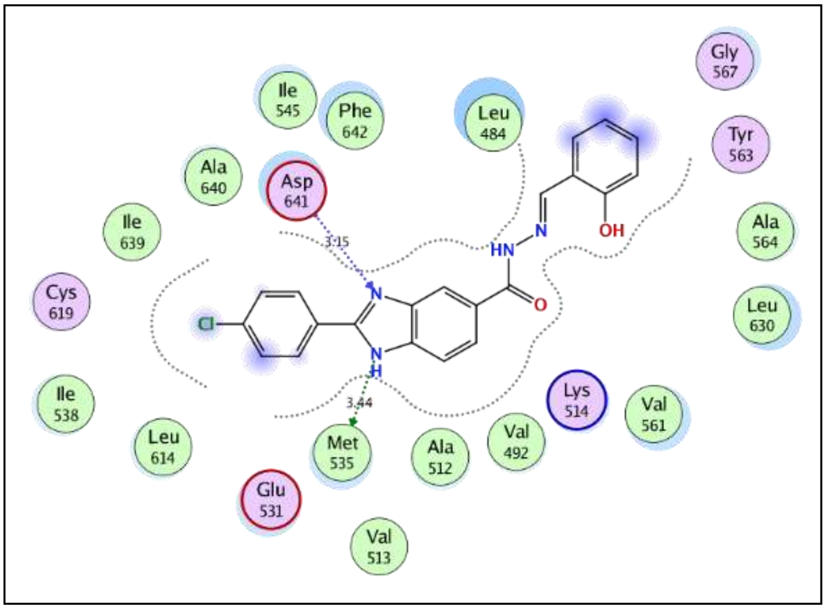

Figure S42. 2D diagram of compound **8o** showing its interaction with the FGFR-1 active site

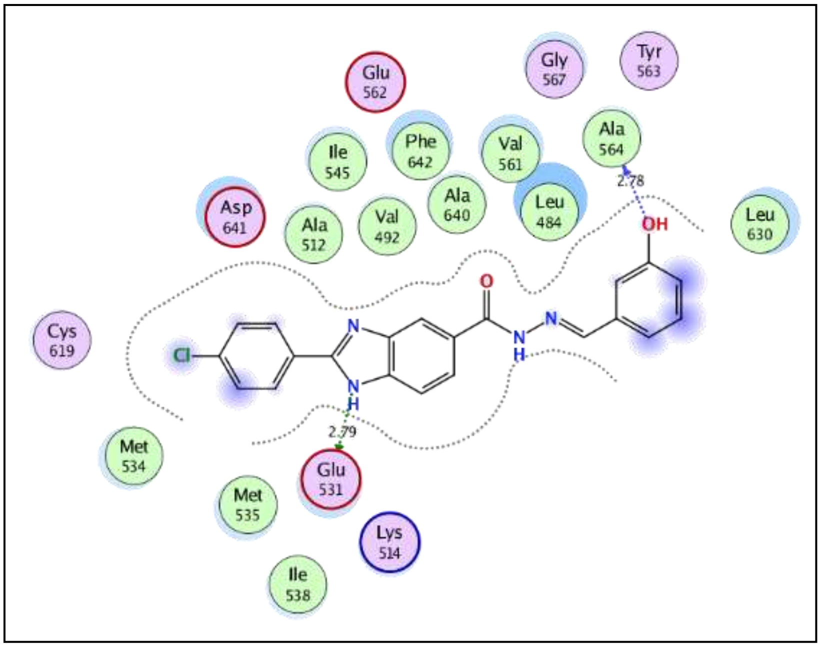

Figure S43. 2D diagram of compound **8p** showing its interaction with the FGFR-1 active site

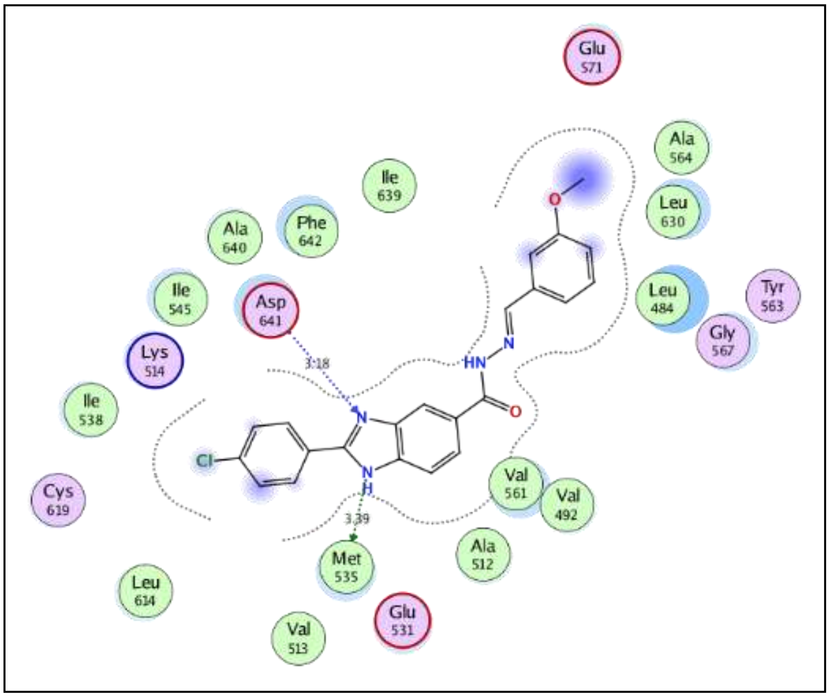

Figure S44. 2D diagram of compound **8q** showing its interaction with the FGFR-1 active site

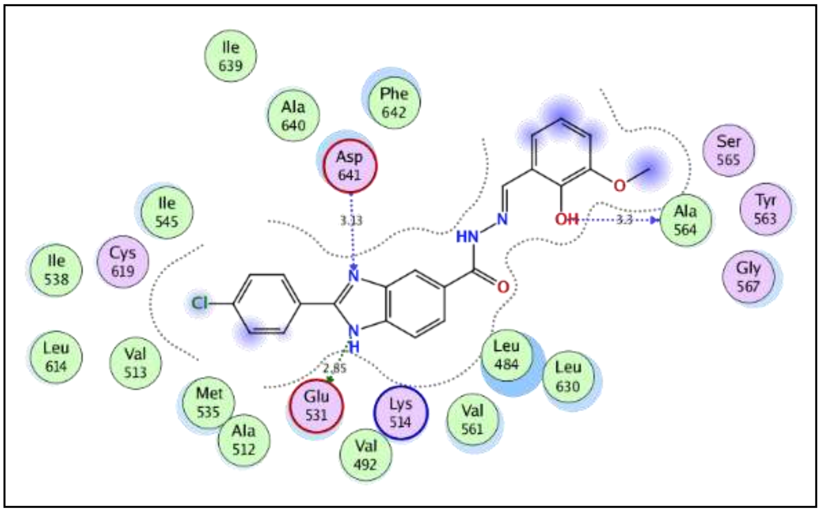

Figure S45. 2D diagram of compound **8r** showing its interaction with the FGFR-1 active site

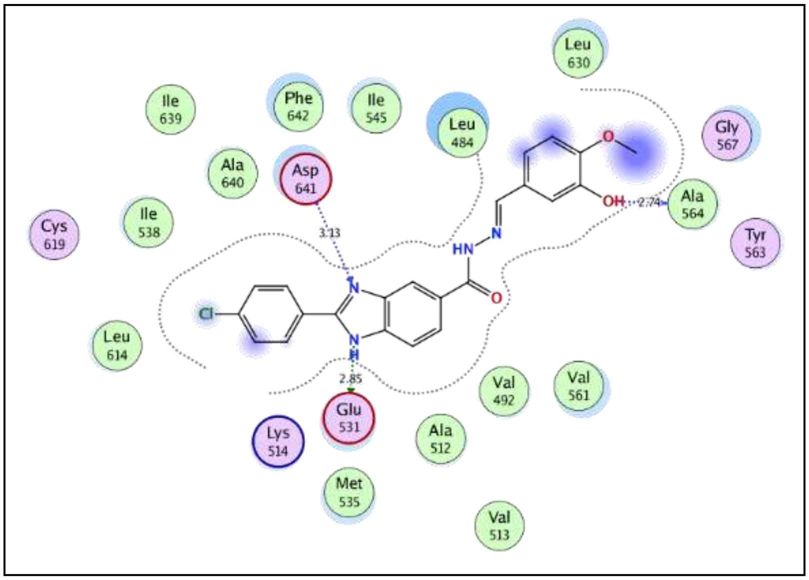

Figure S46. 2D diagram of compound **8s** showing its interaction with the FGFR-1 active site

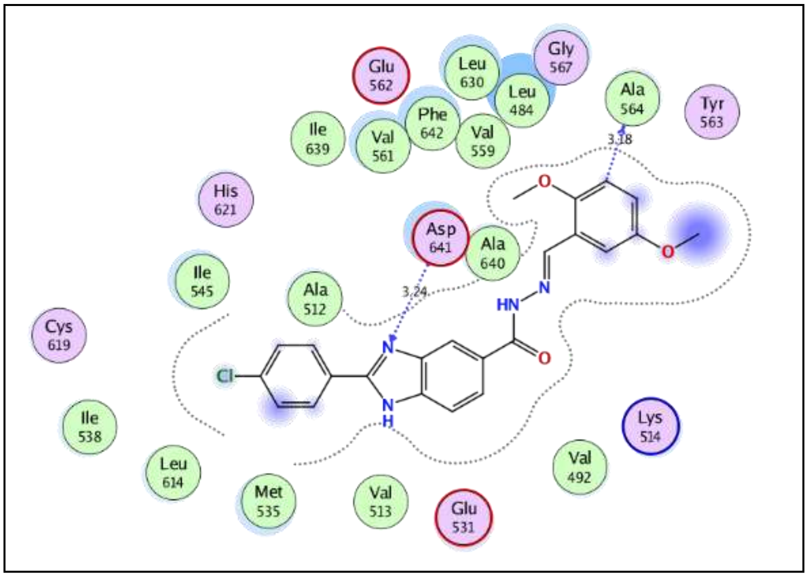

Figure S47. 2D diagram of compound **8t** showing its interaction with the FGFR-1 active site

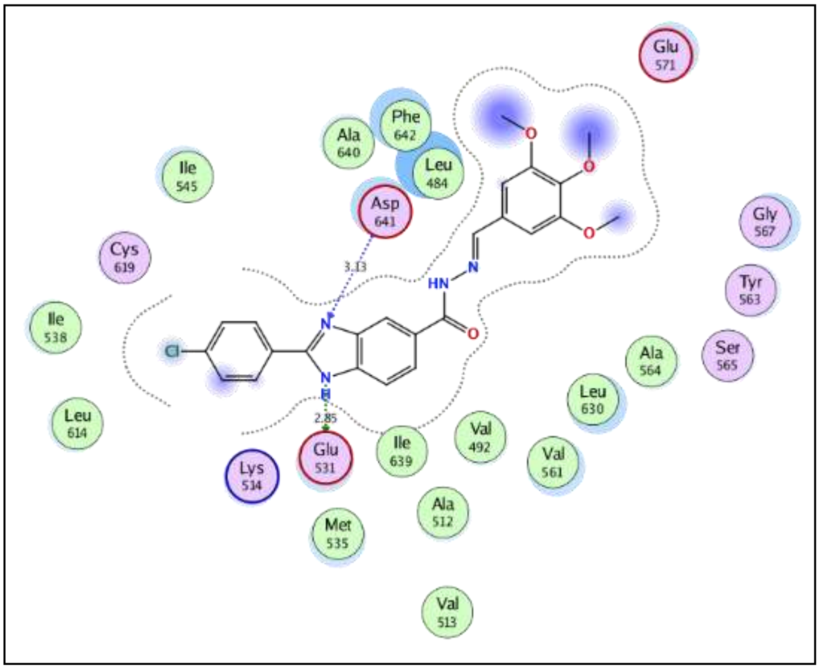

Figure S48. 2D diagram of compound **8u** showing its interaction with the FGFR-1 active site

Docking poses of the designed compounds, in BRAF active site (Figure S49-69)

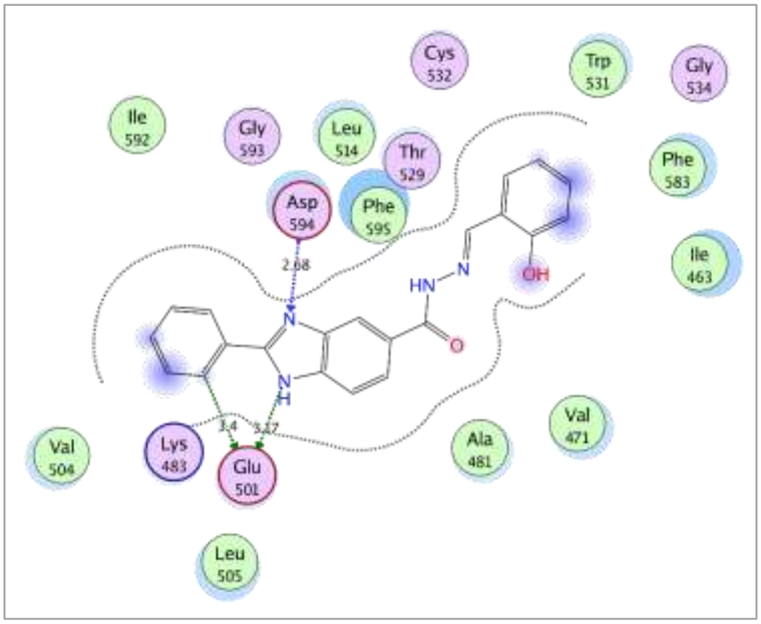

Figure S49. 2D diagram of compound **8a** showing its interaction with the BRAF active site

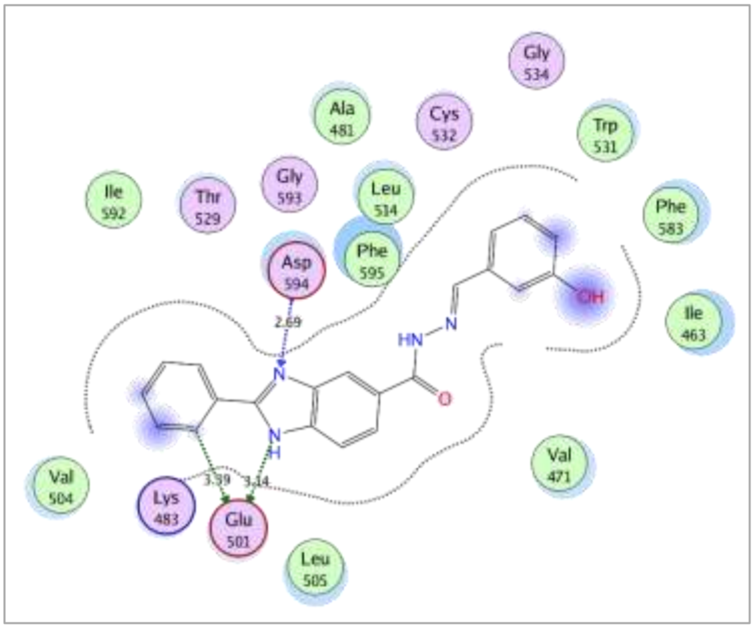

Figure S50. 2D diagram of compound **8b** showing its interaction with the BRAF active site

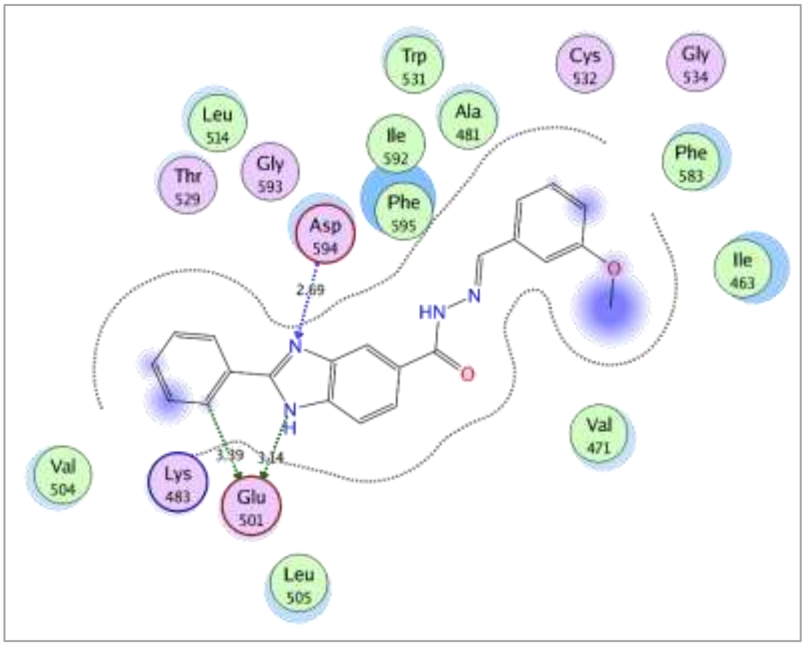

Figure S51. 2D diagram of compound **8c** showing its interaction with the BRAF active site

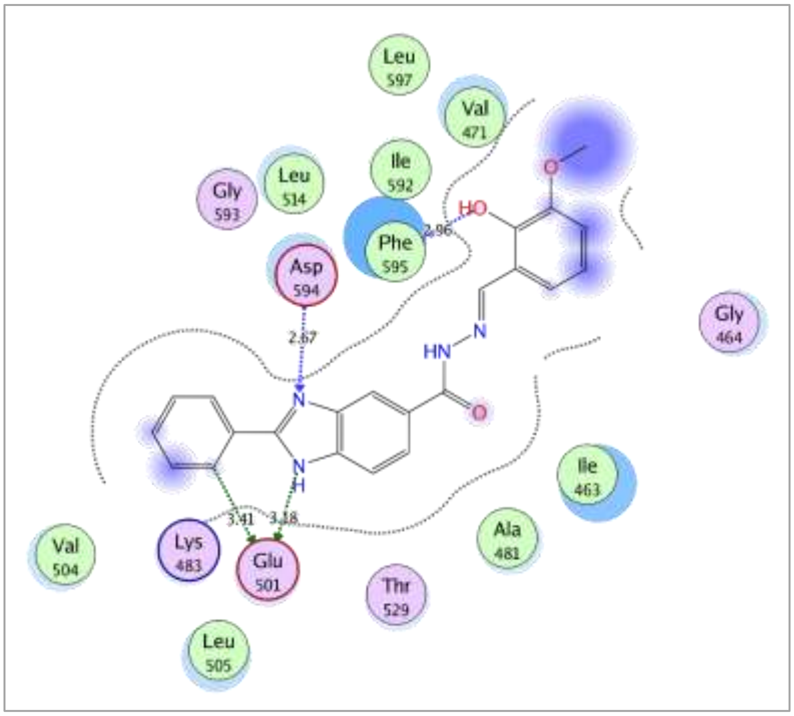

Figure S52. 2D diagram of compound **8d** showing its interaction with the BRAF active site

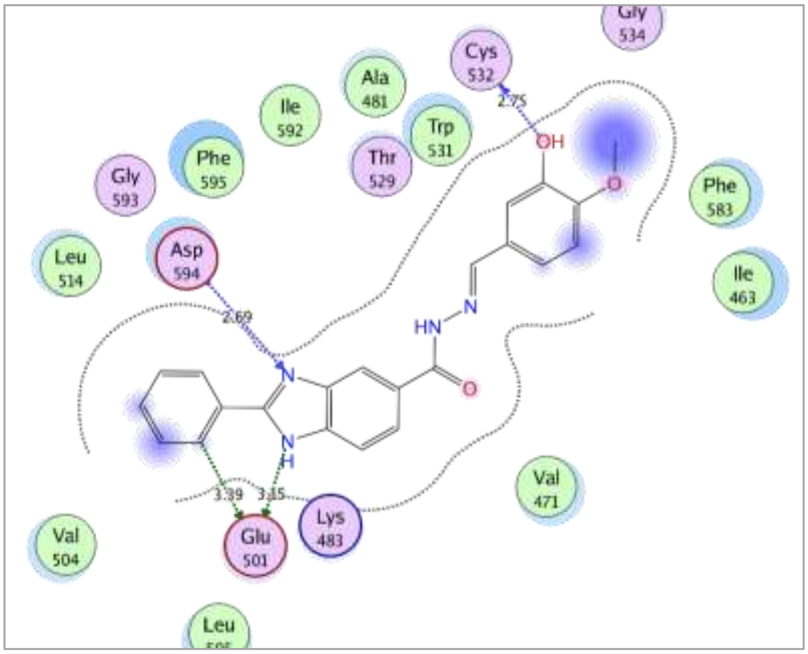

Figure S53. 2D diagram of compound **8e** showing its interaction with the BRAF active site

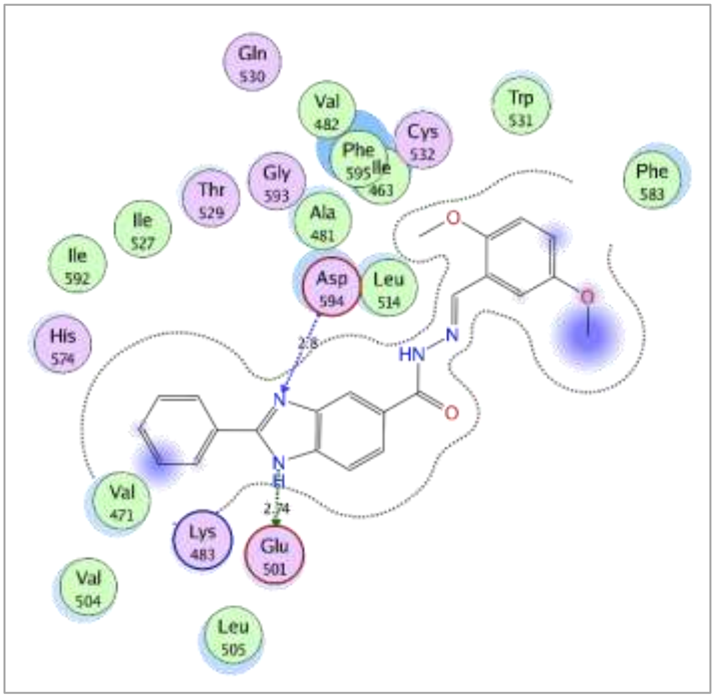

Figure S54. 2D diagram of compound **8f** showing its interaction with the BRAF active site

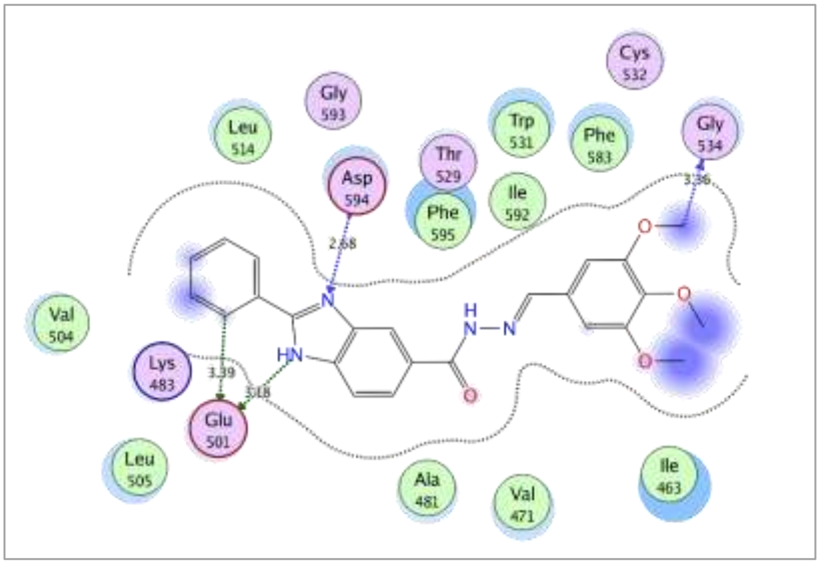

Figure S55. 2D diagram of compound **8g** showing its interaction with the BRAF active site

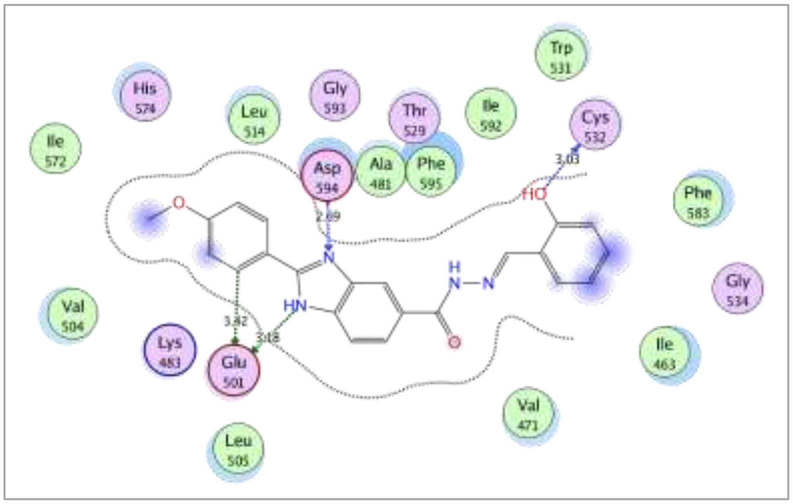

Figure S56. 2D diagram of compound **8h** showing its interaction with the BRAF active site

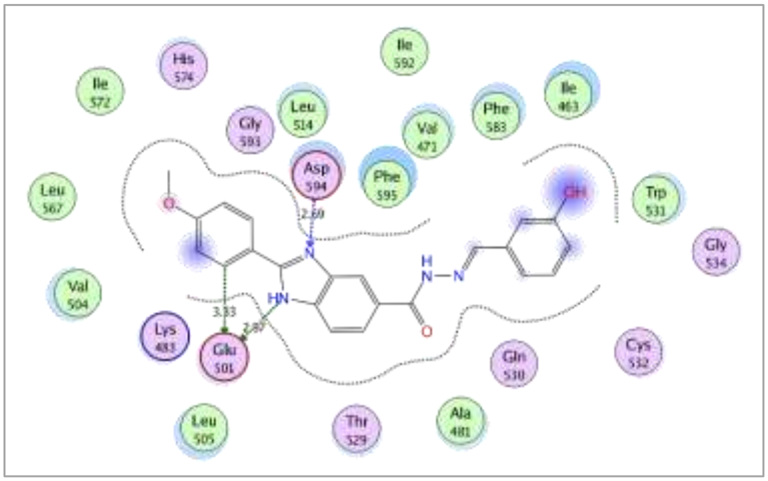

Figure S57. 2D diagram of compound **8i** showing its interaction with the BRAF active site

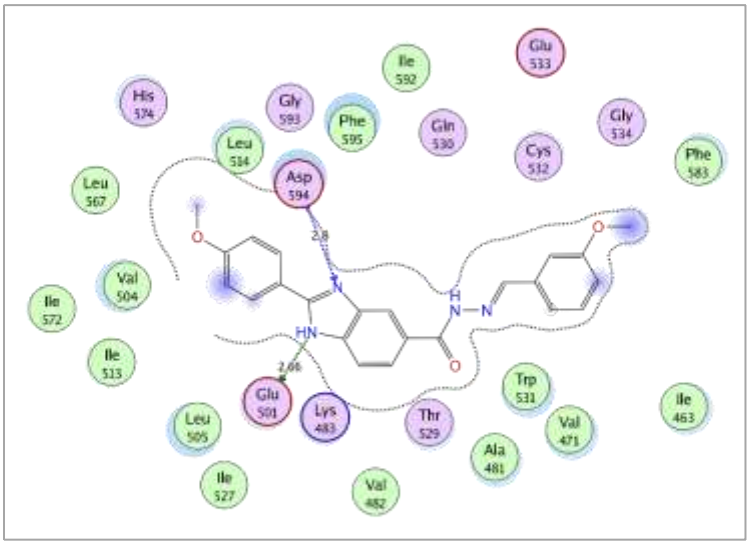

Figure S58. 2D diagram of compound **8j** showing its interaction with the BRAF active site

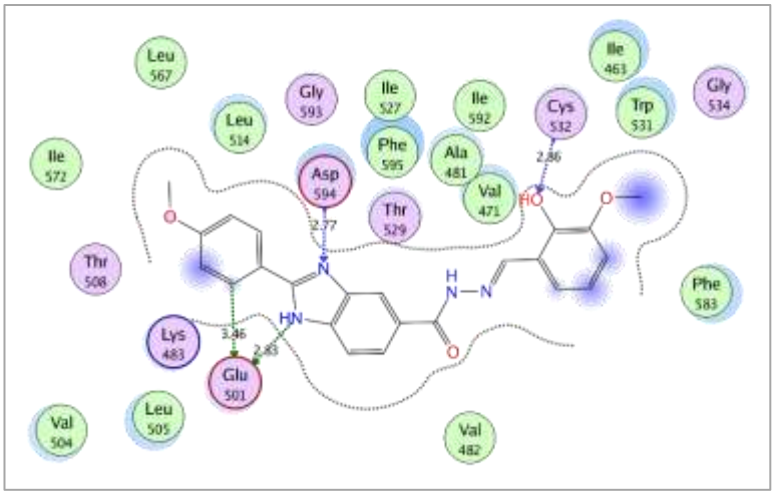

Figure S59. 2D diagram of compound **8k** showing its interaction with the BRAF active site

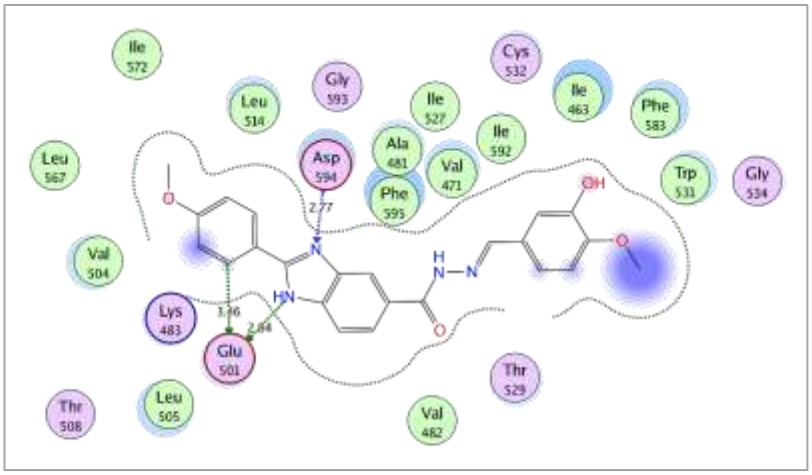

Figure S60. 2D diagram of compound **8l** showing its interaction with the BRAF active site

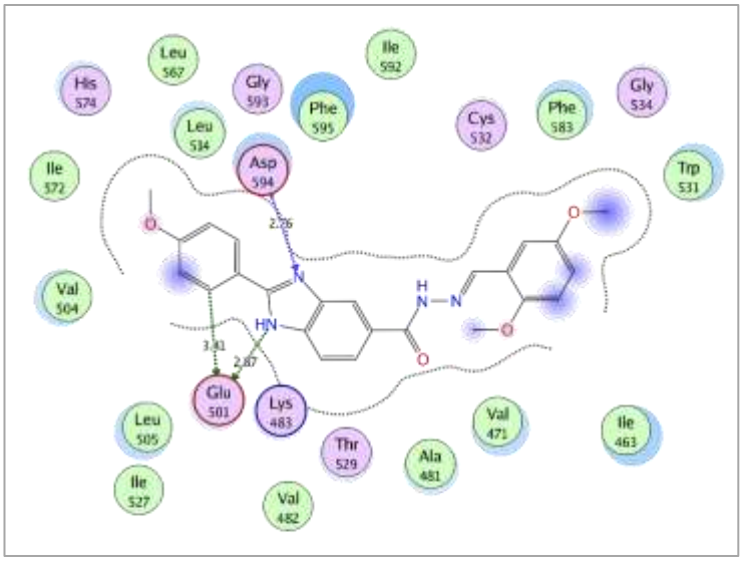

Figure S61. 2D diagram of compound **8m** showing its interaction with the BRAF active site

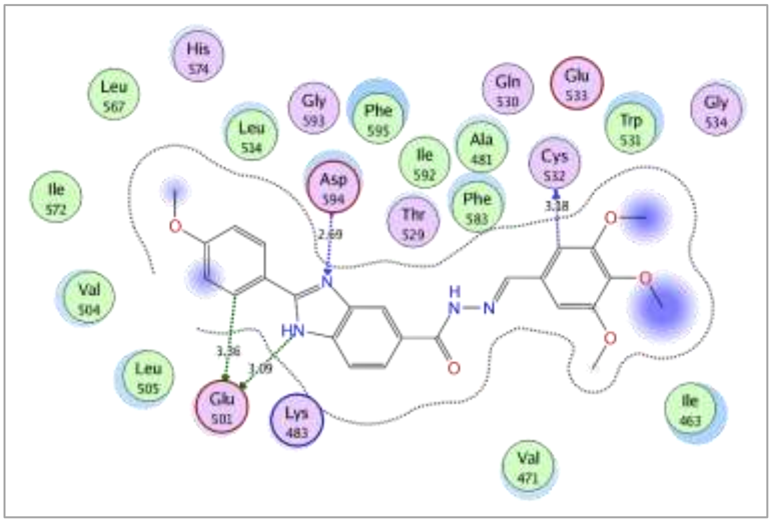

Figure S62. 2D diagram of compound **8n** showing its interaction with the BRAF active site

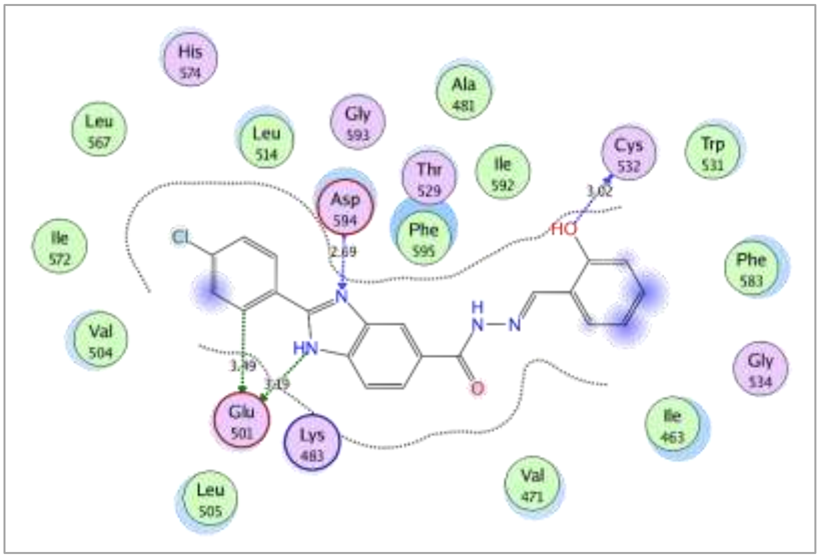

Figure S63. 2D diagram of compound **8o** showing its interaction with the BRAF active site

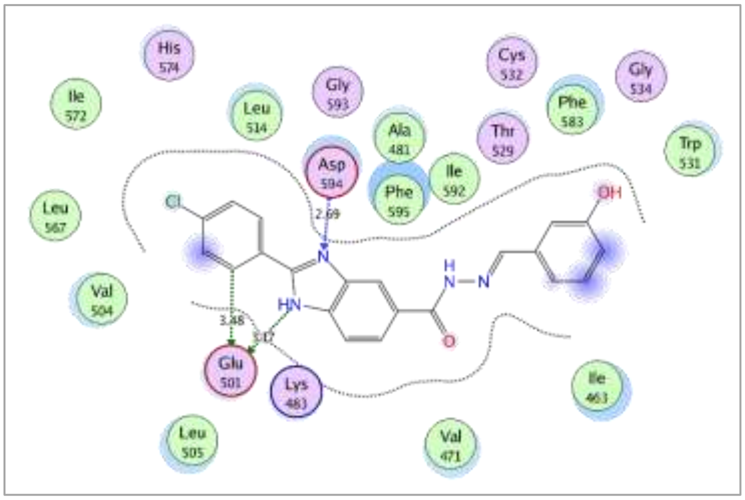

Figure S64. 2D diagram of compound **8p** showing its interaction with the BRAF active site

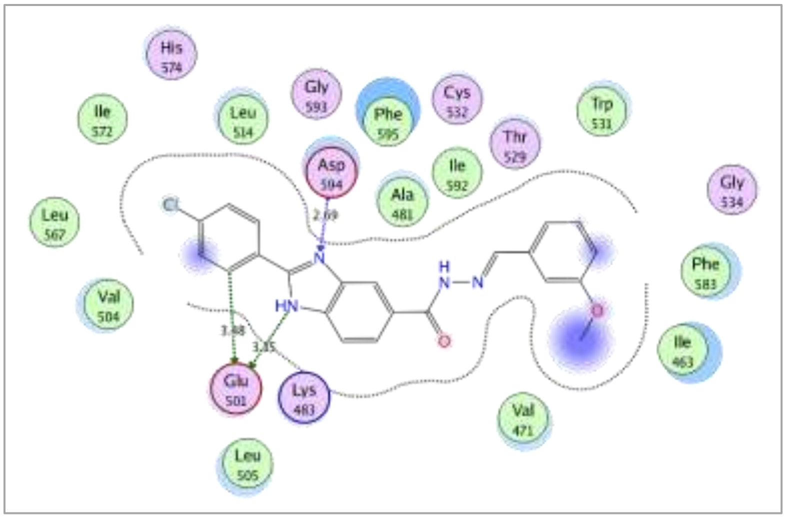

Figure S65. 2D diagram of compound **8q** showing its interaction with the BRAF active site

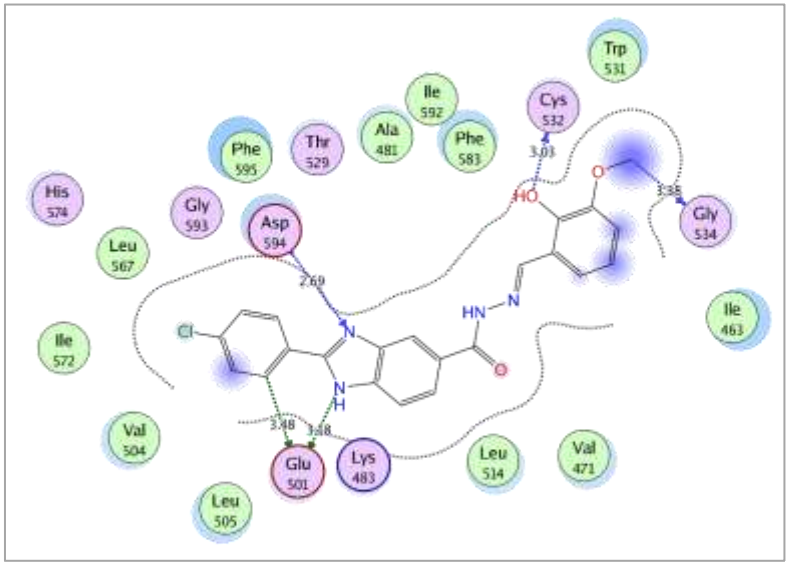

Figure S66. 2D diagram of compound **8r** showing its interaction with the BRAF active site

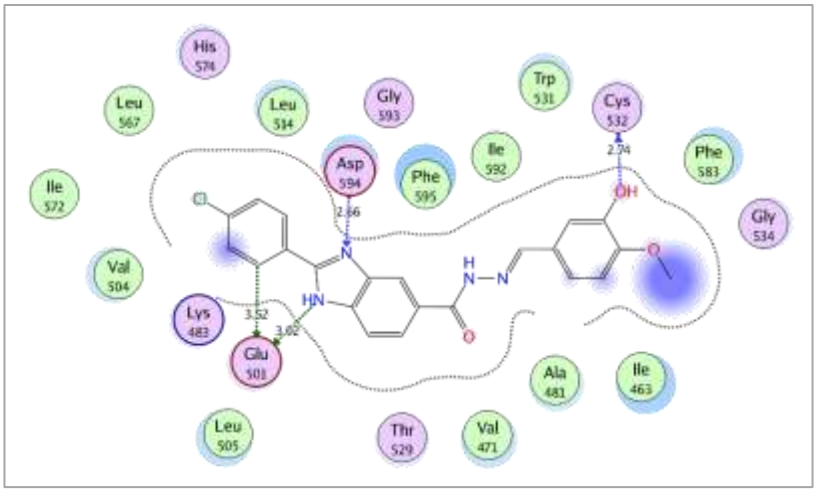

Figure S67. 2D diagram of compound **8s** showing its interaction with the BRAF active site

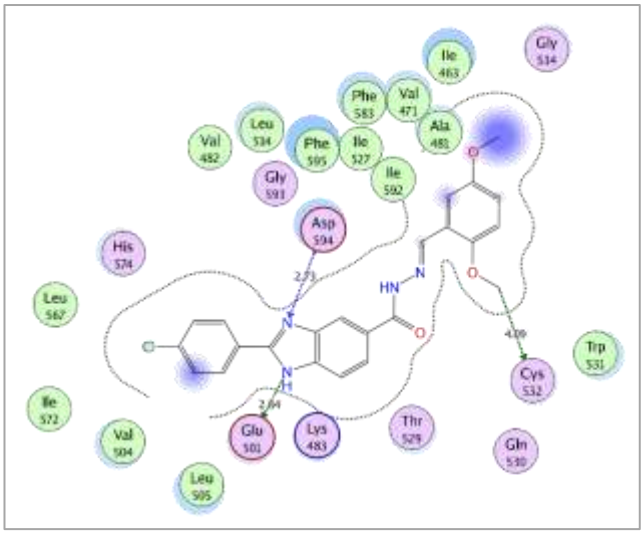

Figure S68. 2D diagram of compound **8t** showing its interaction with the BRAF active site

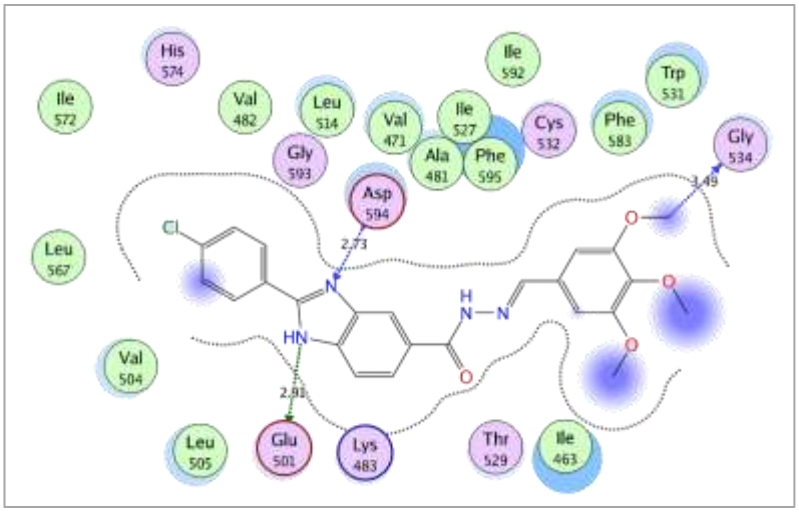

Figure S69. 2D diagram of compound **8u** showing its interaction with the BRAF active site

4. ADME properties prediction

SwissADME online web tool [http://www.swissadme.ch/index.php] was used to predict the physicochemical and AMDE properties of the target compounds **8a-u**. The target compounds' SMILES were generated using Molecular Operating Environment (MOE, 2022.02) software then they were submitted to the online web tool.

Table S10. SwissADME predicted physicochemical, AMDE properties as well as medicinal chemistry friendliness of the target compounds

ID	MW	#Rot. bond	#H-bond acc.	#H-bond don.	TPSA	Cons. Log P	Esol Solubility Class
**8a**	356.38	5	4	3	90.37	3.22	Moderately soluble
**8b**	356.38	5	4	3	90.37	3.23	Moderately soluble
**8c**	370.40	6	4	2	79.37	3.60	Moderately soluble
**8d**	386.40	6	5	3	99.60	3.29	Moderately soluble
**8e**	386.40	6	5	3	99.60	3.23	Moderately soluble
**8f**	400.43	7	5	2	88.60	3.63	Moderately soluble
**8g**	430.46	8	6	2	97.83	3.58	Moderately soluble
**8h**	386.40	6	5	3	99.60	3.17	Moderately soluble
**8i**	386.40	6	5	3	99.60	3.16	Moderately soluble
**8j**	400.43	7	5	2	88.60	3.54	Moderately soluble
**8k**	416.43	7	6	3	108.83	3.22	Moderately soluble
**8l**	416.43	7	6	3	108.83	3.16	Moderately soluble
**8m**	430.46	8	6	2	97.83	3.57	Moderately soluble
**8n**	460.48	9	7	2	107.06	3.55	Moderately soluble
**8o**	390.82	5	4	3	90.37	3.70	Moderately soluble
**8p**	390.82	5	4	3	90.37	3.66	Moderately soluble
**8q**	404.85	6	4	2	79.37	4.13	Moderately soluble
**8r**	420.85	6	5	3	99.60	3.74	Moderately soluble
**8s**	420.85	6	5	3	99.60	3.79	Moderately soluble
**8t**	434.87	7	5	2	88.60	4.21	Moderately soluble
**8u**	464.90	8	6	2	97.83	4.11	Moderately soluble

Section II: Practical Results

1. NMR Spectra of 2,5-disubstituted benzimidazole 8a-u

N'-(2-Hydroxybenzylidene)-2-phenyl-1*H*-benzo[*d*]imidazole-5-carbohydrazide (**8a**)

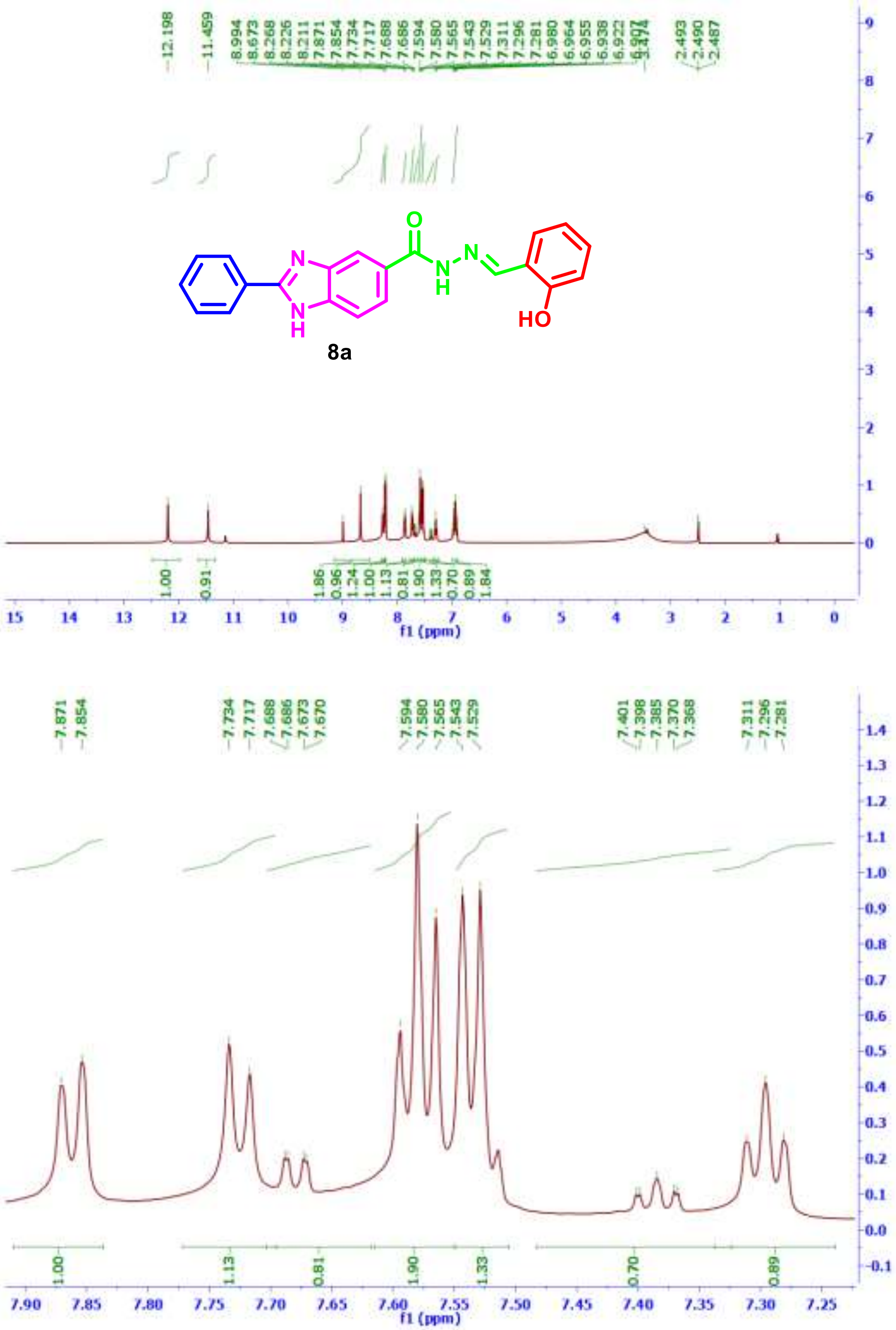

Figure S1. ^{1}H (400 MHz) NMR spectrum of **8a** in DMSO-d_6

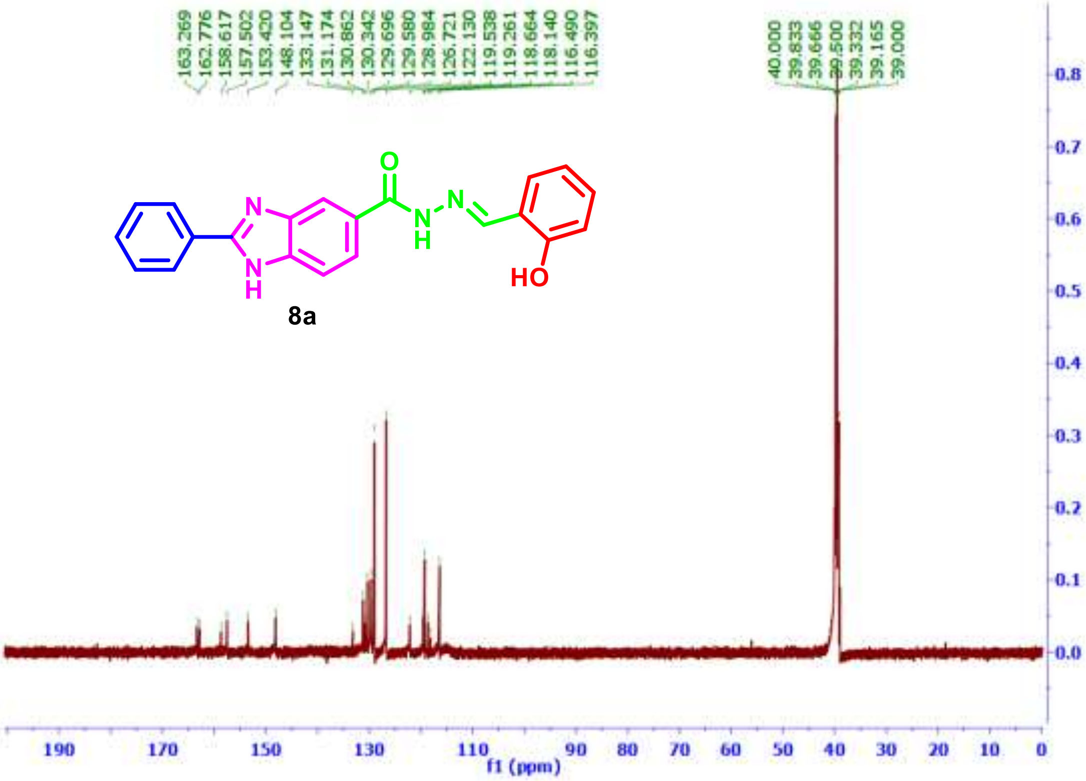

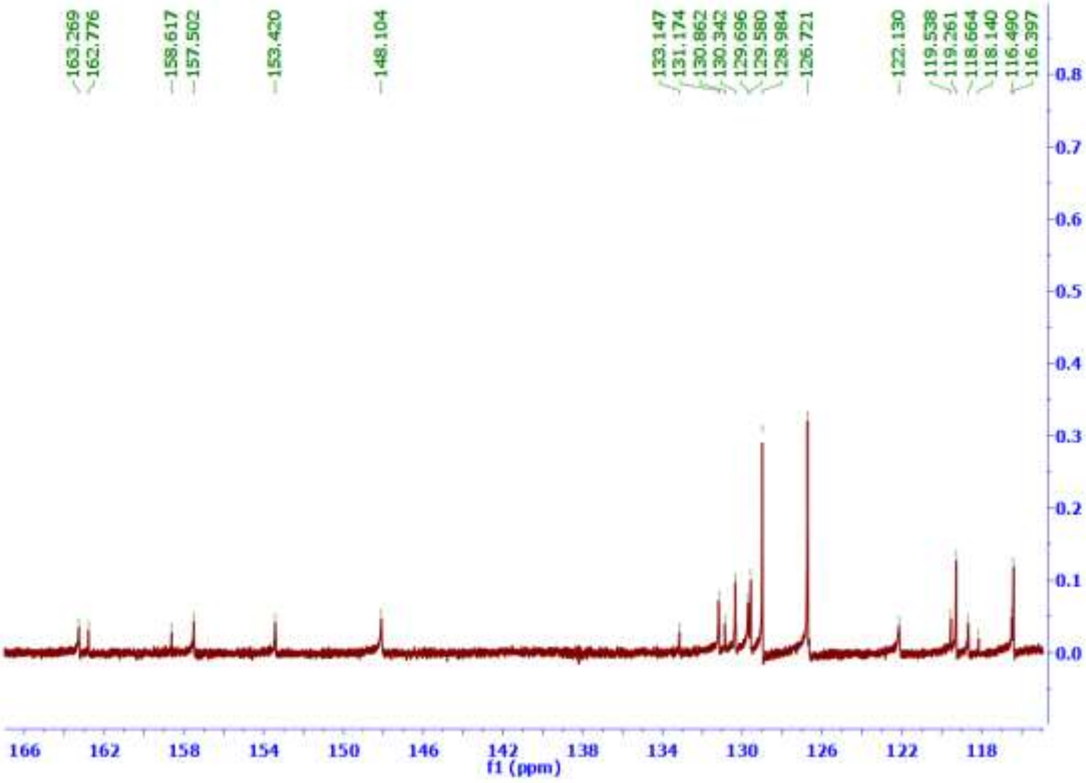

Figure S2. ^{13}C (100 MHz) NMR spectrum of **8a** in DMSO-d_6

N'-(3-Hydroxybenzylidene)-2-phenyl-1*H*-benzo[*d*]imidazole-5-carbohydrazide (**8b**)

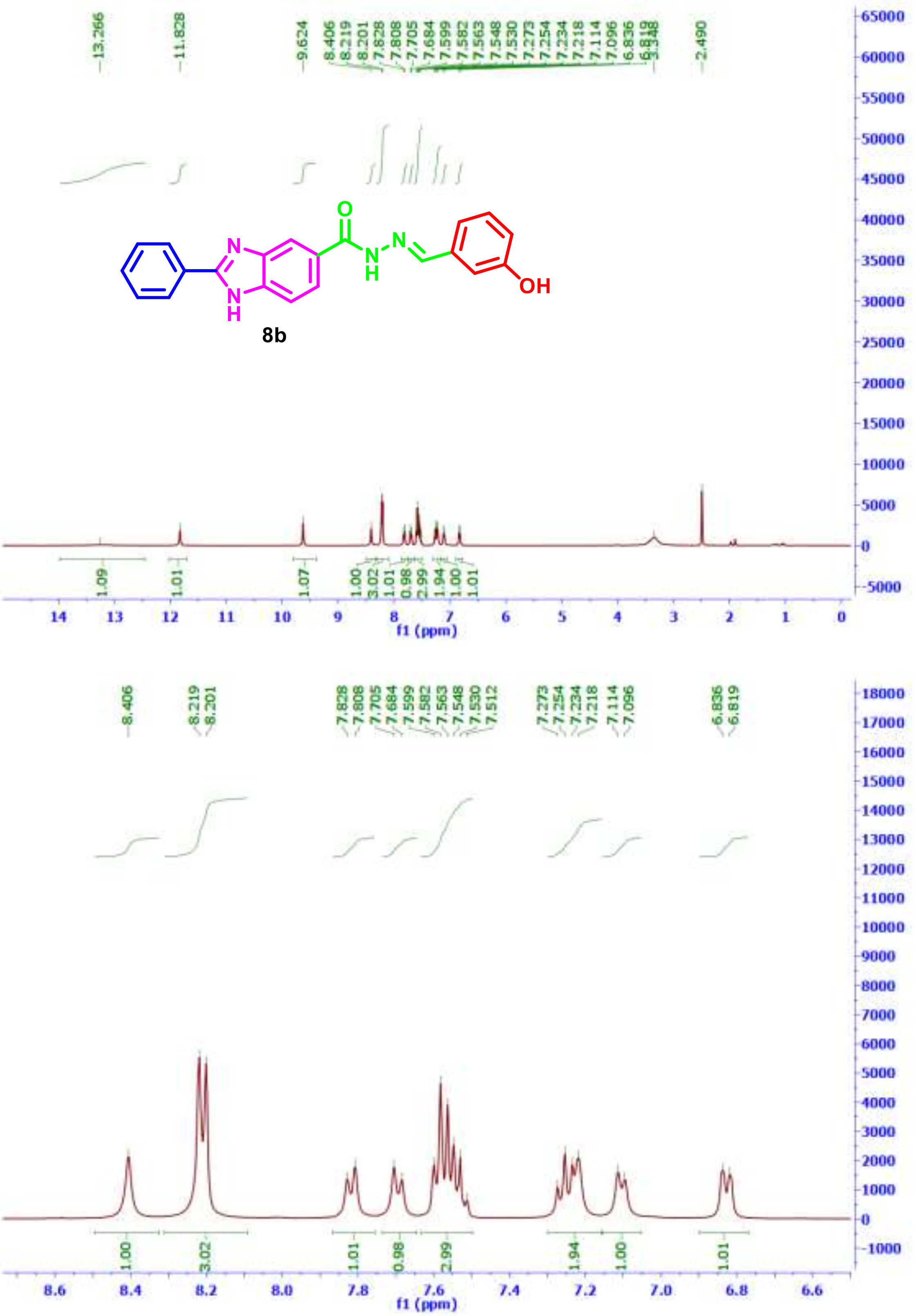

Figure S3. ^{1}H (400 MHz) NMR spectrum of **8b** in DMSO-d_6

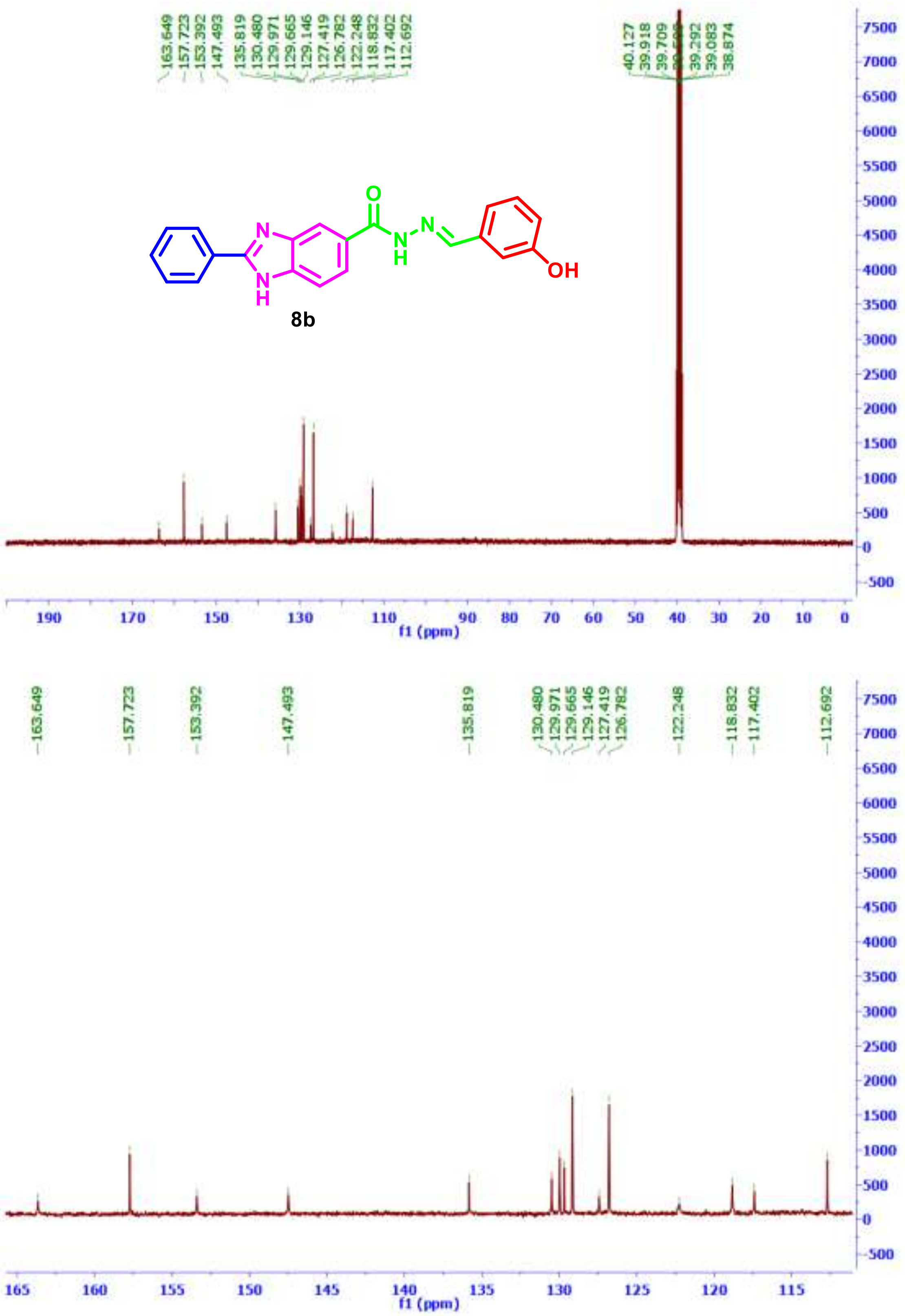

Figure S4. ^{13}C (100 MHz) NMR spectrum of **8b** in DMSO-d_6

N'-(3-Methoxybenzylidene)-2-phenyl-1*H*-benzo[*d*]imidazole-5-carbohydrazide (**8c**)

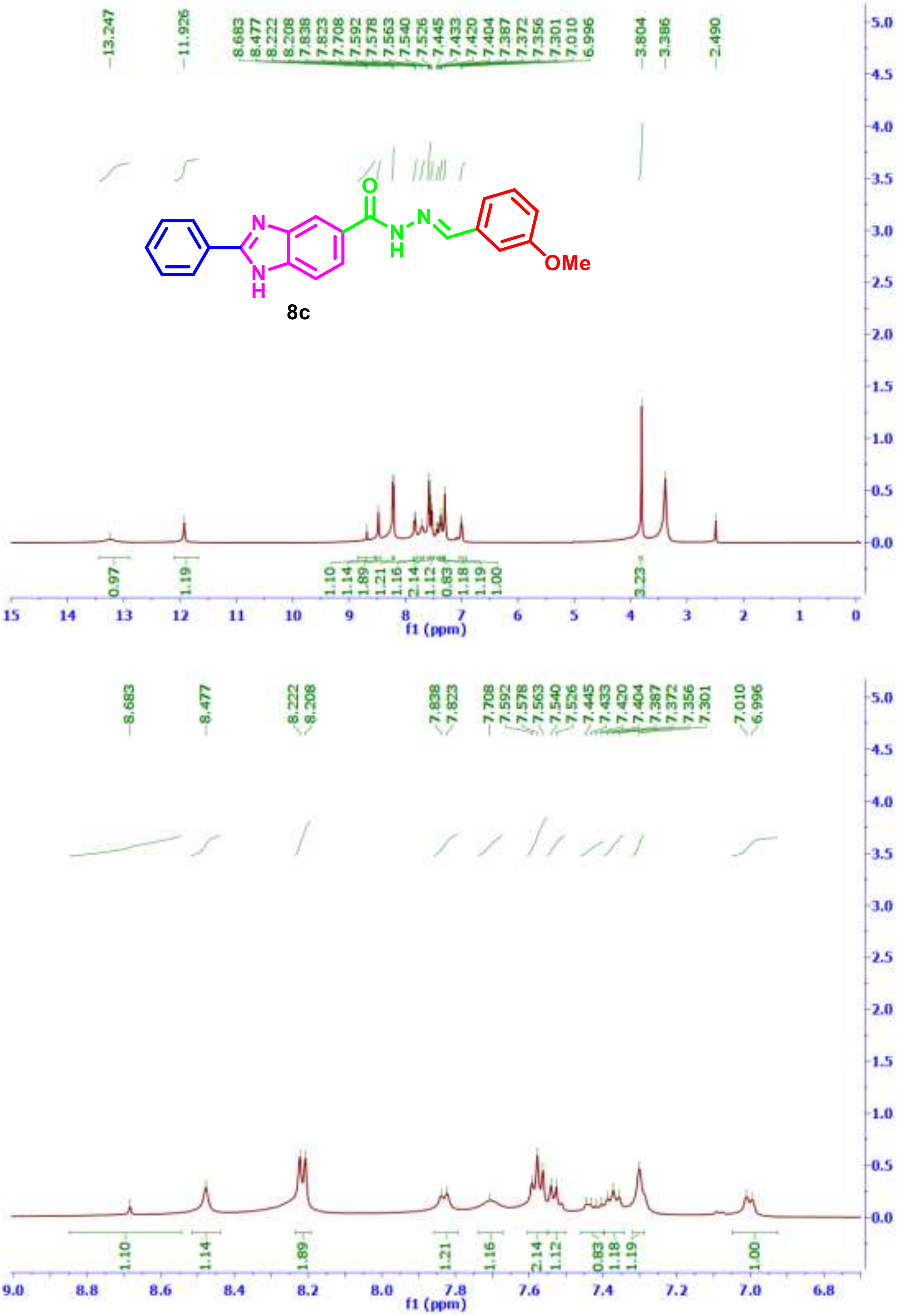

Figure S5. ^{1}H (500 MHz) NMR spectrum of **8c** in DMSO-d_6

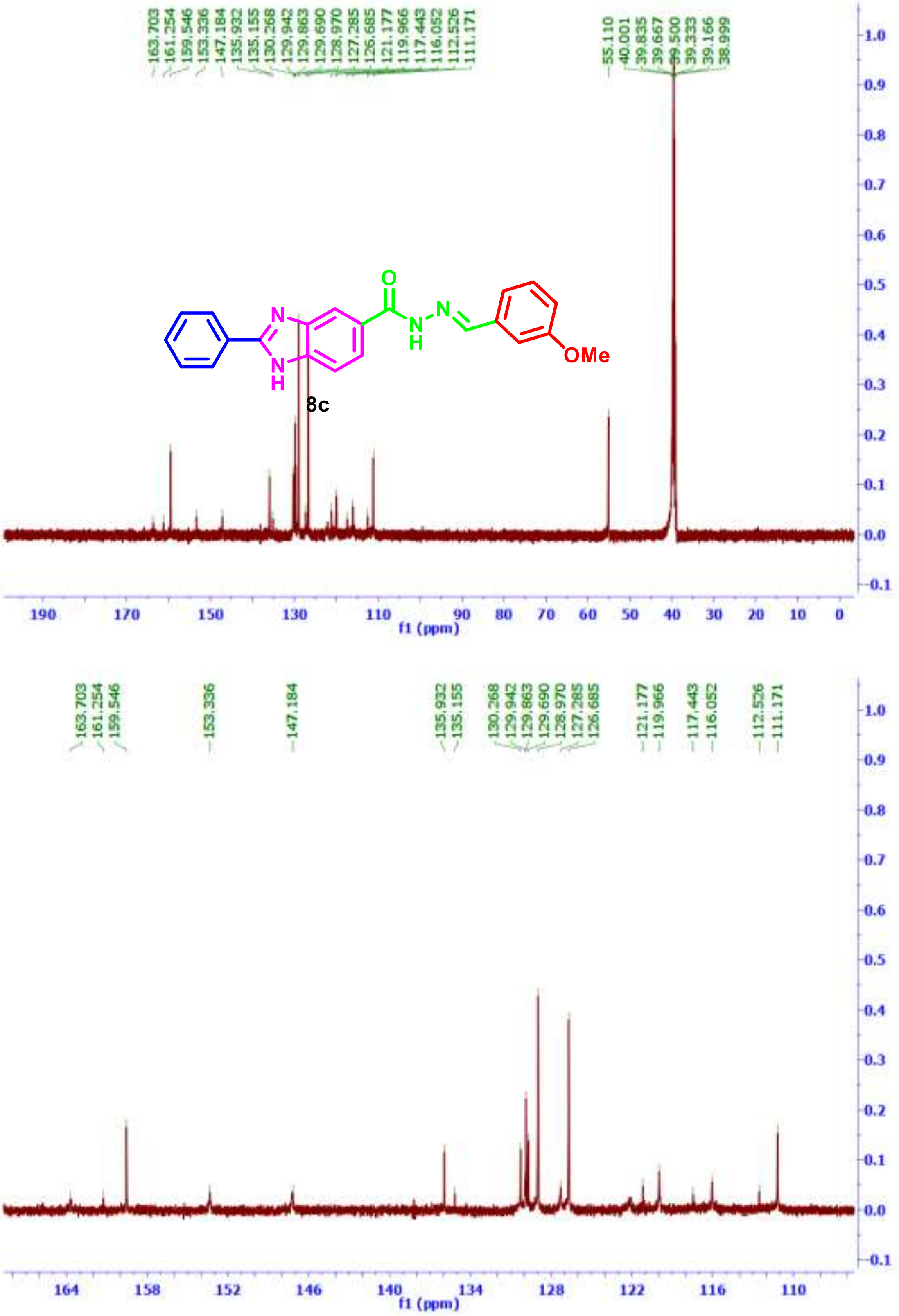

Figure S6. ^{13}C (125 MHz) NMR spectrum of **8c** in DMSO-d_6

N'-(2-Hydroxy-3-methoxybenzylidene)-2-phenyl-1*H*-benzo[*d*]imidazole-5-carbohydrazide (**8d**)

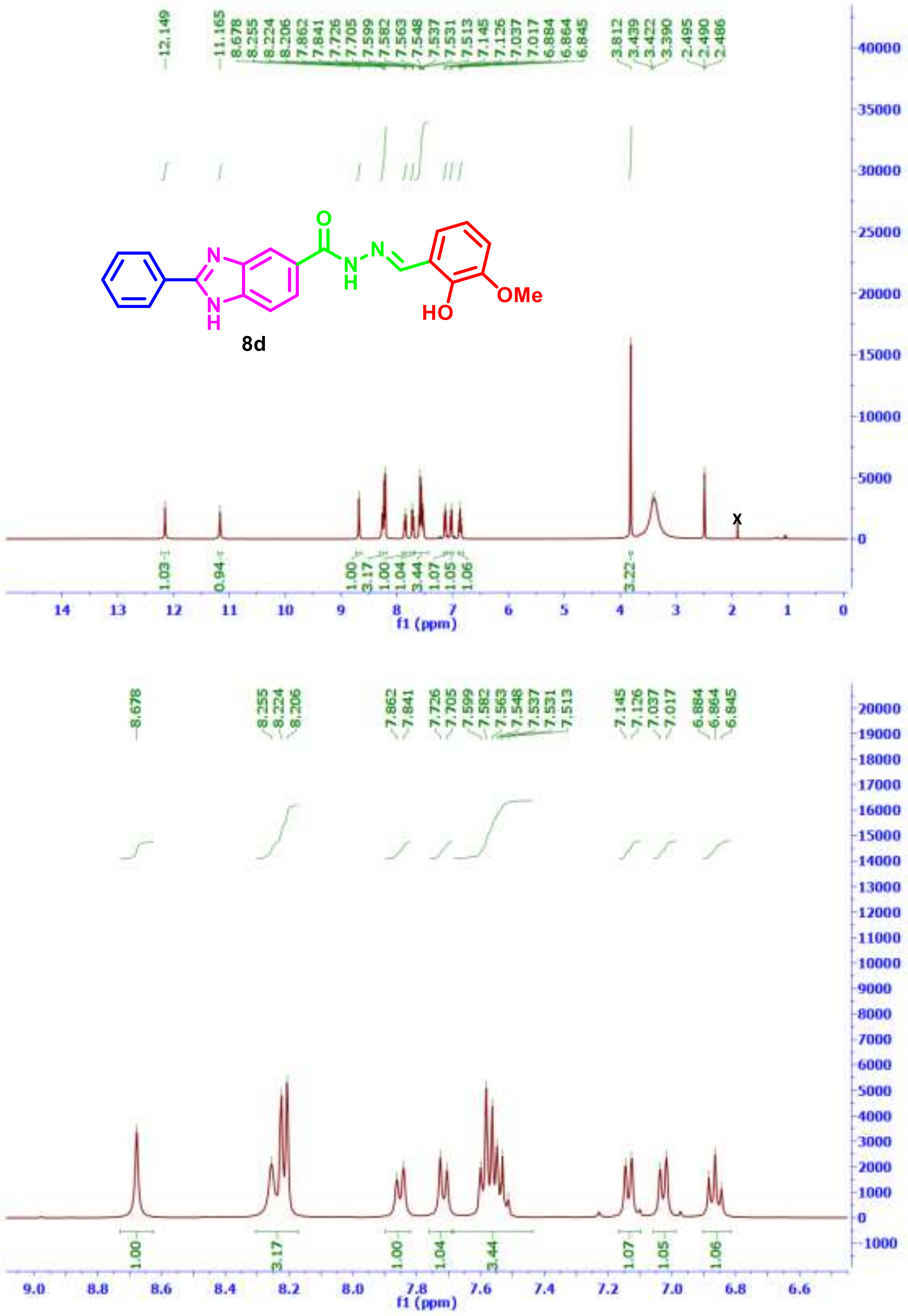

Figure S7. ^{1}H (400 MHz) NMR spectrum of **8d** in DMSO-d_6

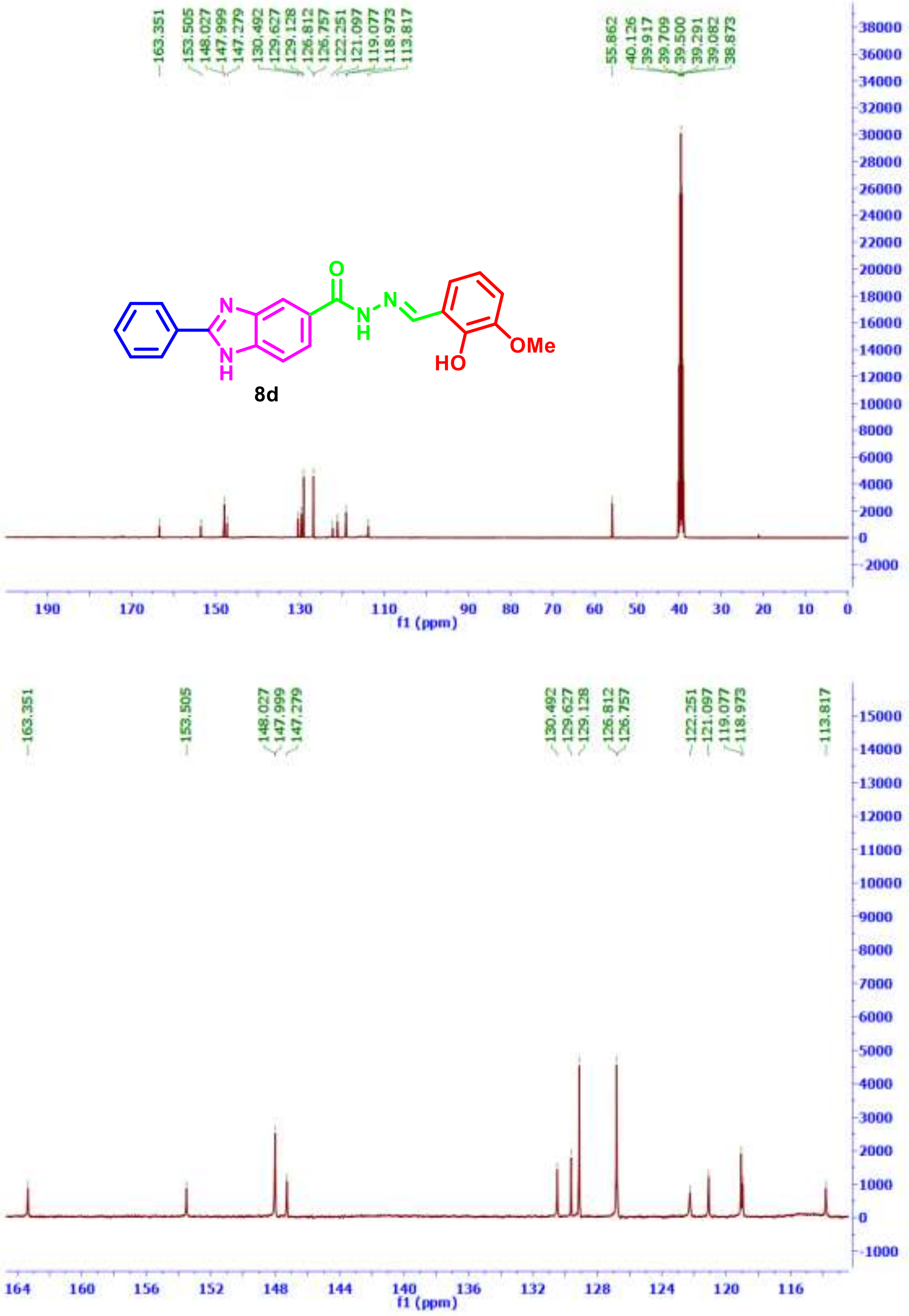

Figure S8. ^{13}C (100 MHz) NMR spectrum of **8d** in DMSO-d_6

N'-(3-Hydroxy-4-methoxybenzylidene)-2-phenyl-1*H*-benzo[*d*]imidazole-5-carbohydrazide (**8e**)

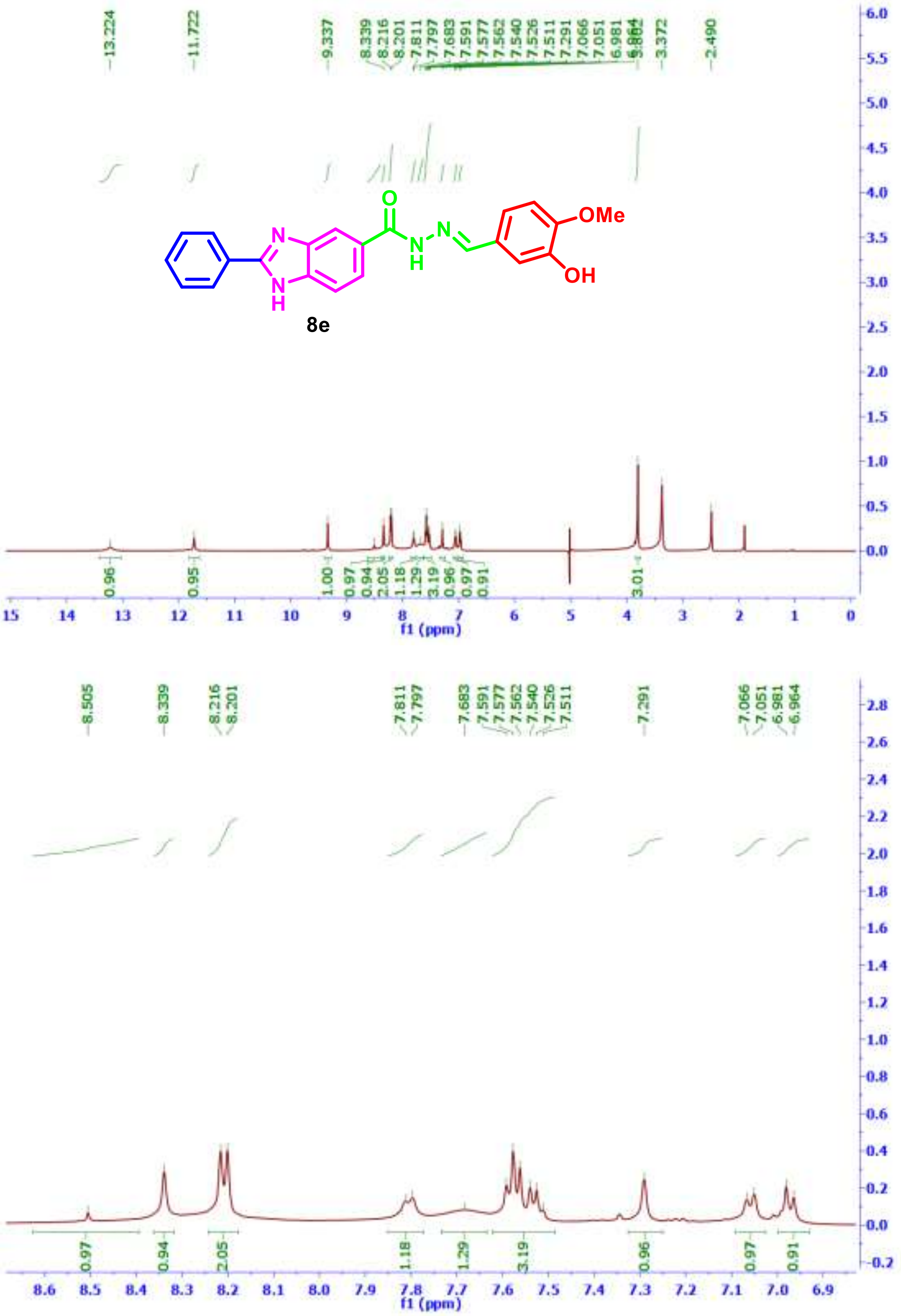

Figure S9. ^{1}H (500 MHz) NMR spectrum of **8e** in DMSO-d_6

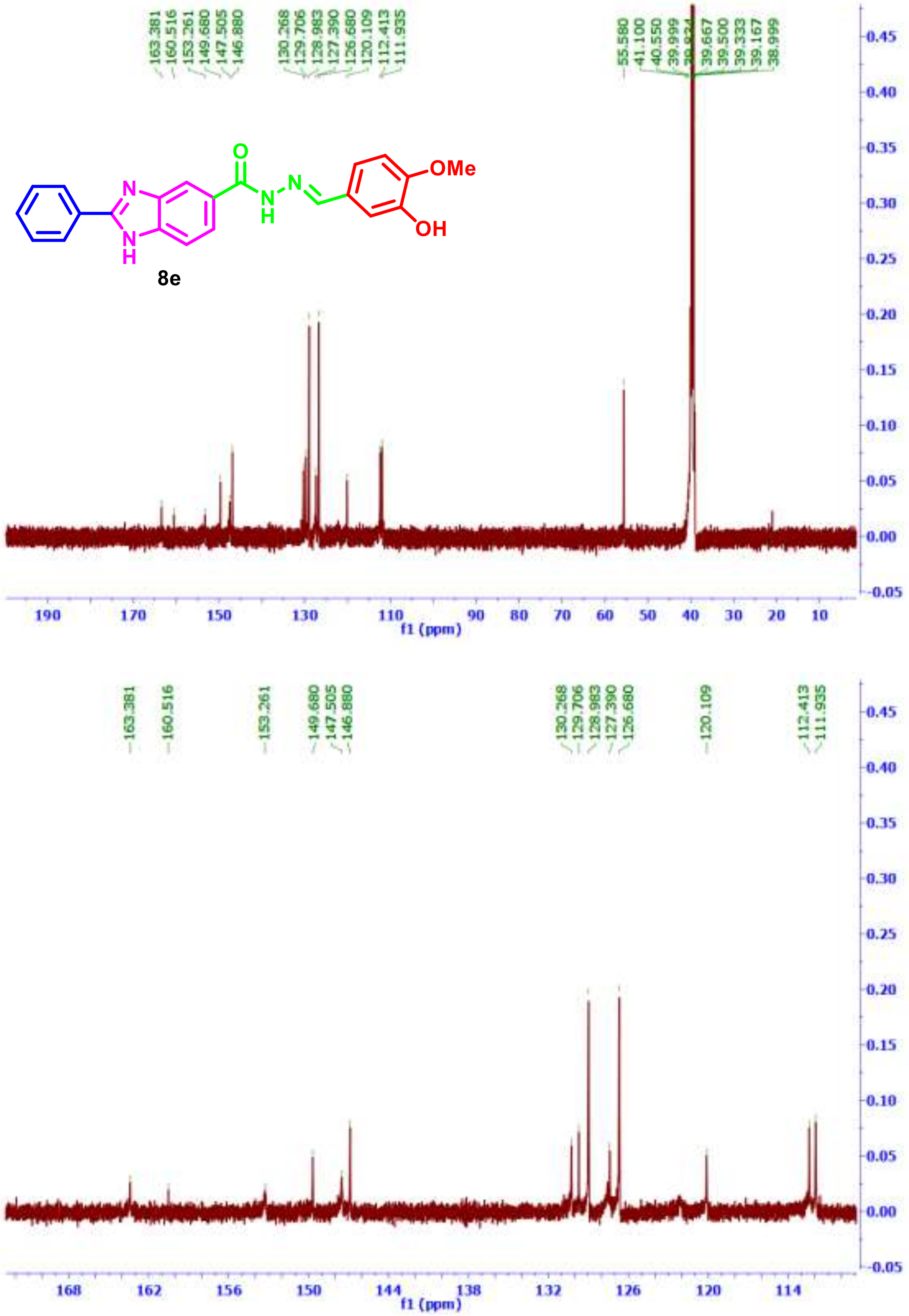

Figure S10. ^{13}C (125 MHz) NMR spectrum of **8e** in DMSO-d_6

N'-(2,5-Dimethoxybenzylidene)-2-phenyl-1*H*-benzo[*d*]imidazole-5-carbohydrazide (**8f**)

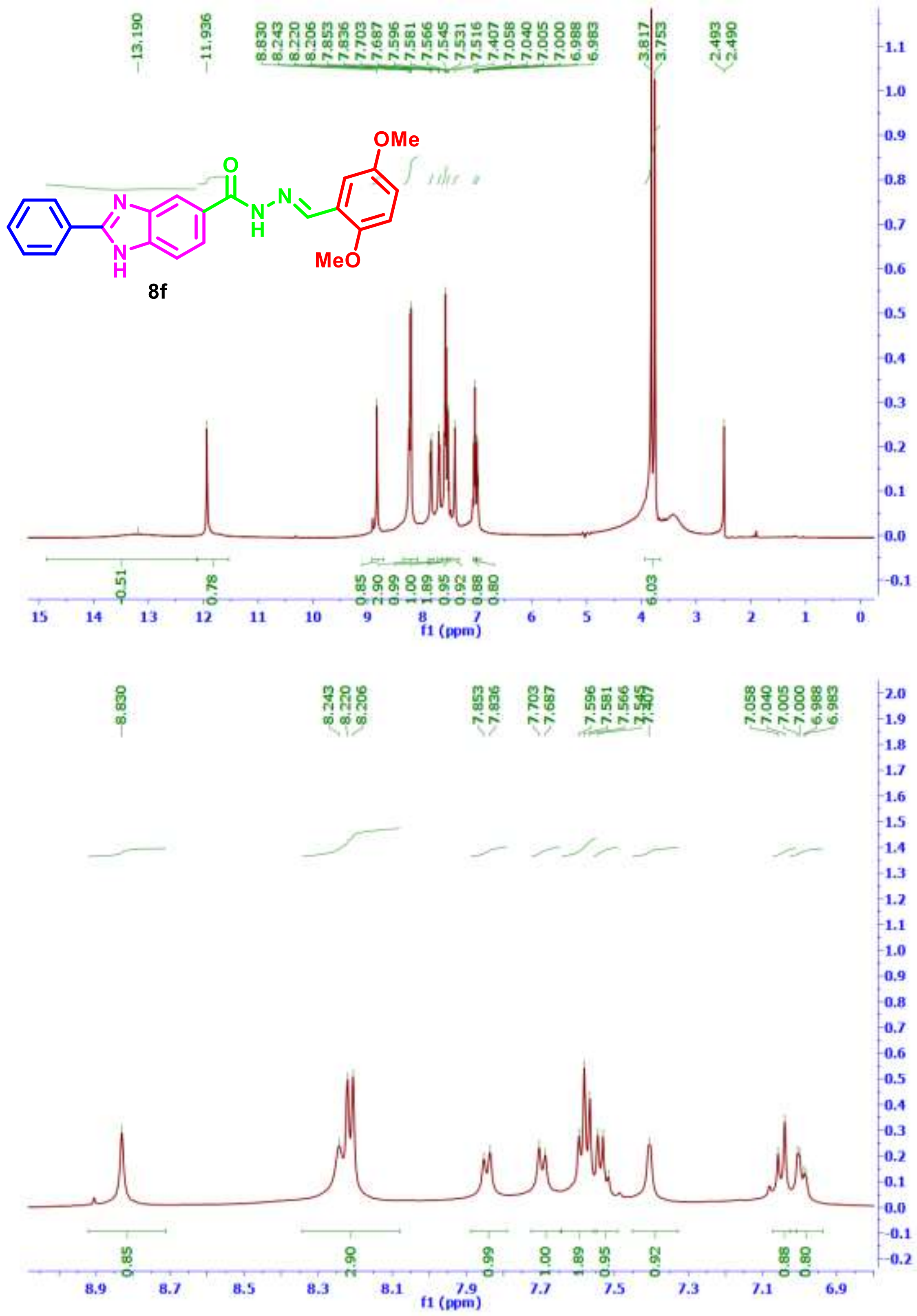

Figure S11. ^{1}H (500 MHz) NMR spectrum of **8f** in DMSO-d_6

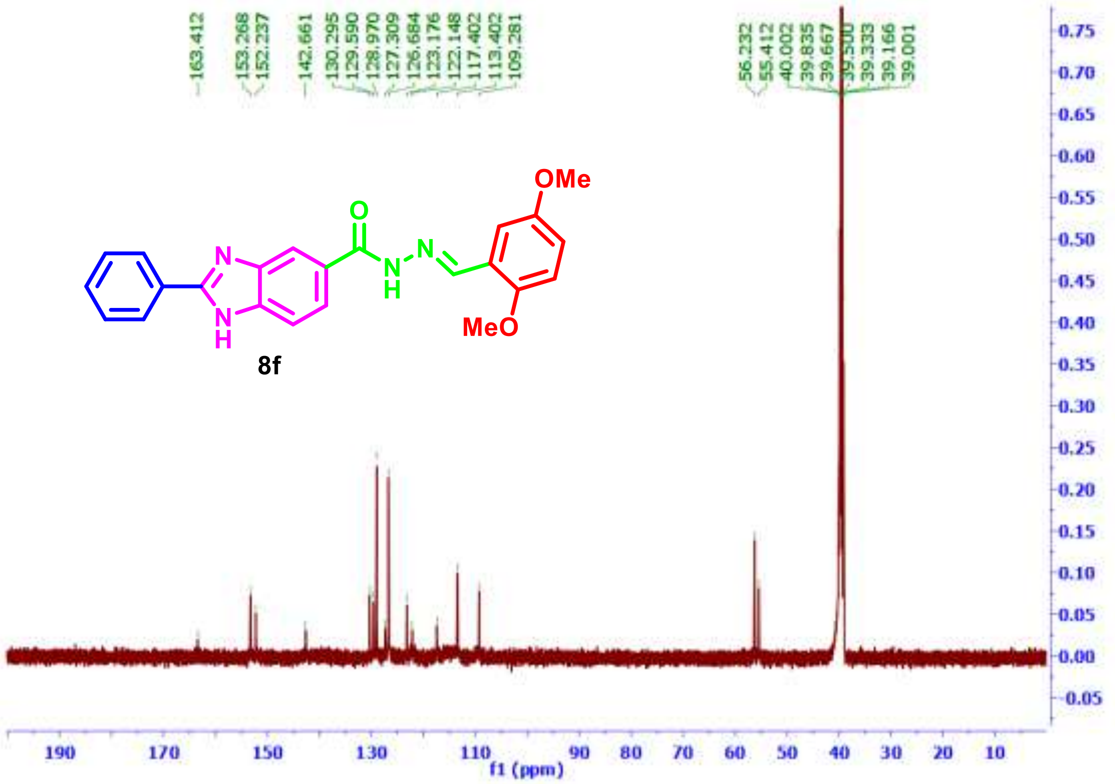

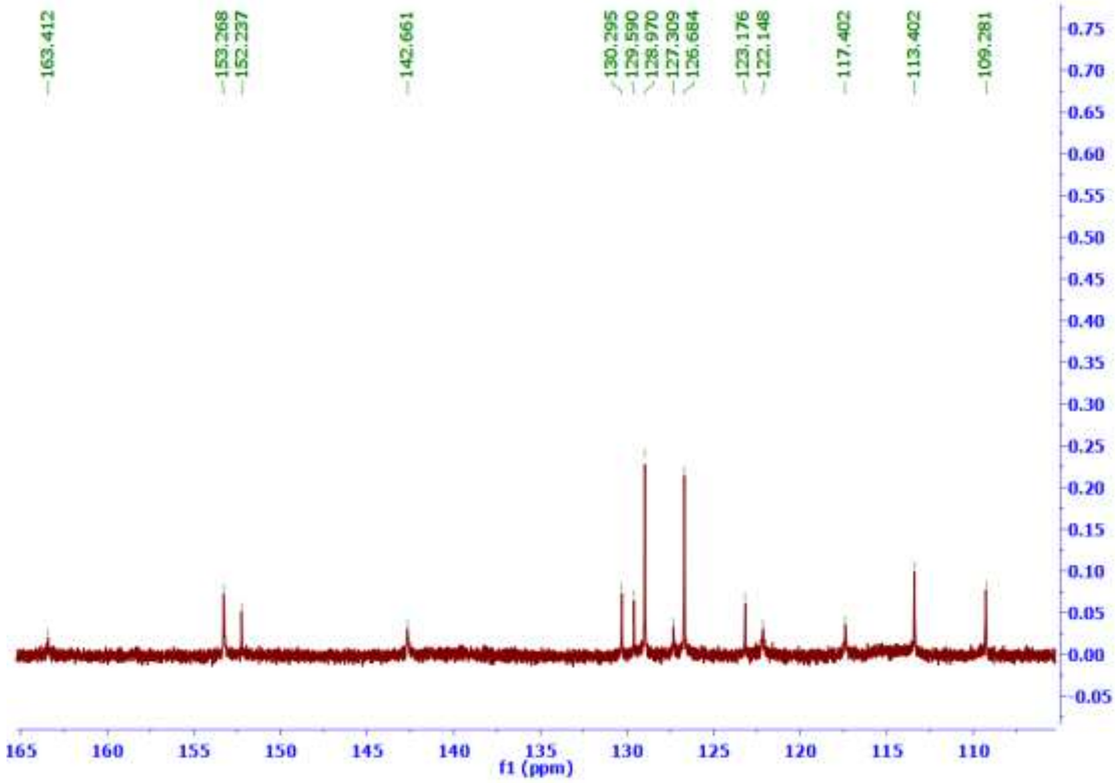

Figure S12. ^{13}C (125 MHz) NMR spectrum of **8f** in DMSO-d_6

2-Phenyl-*N*'-(3,4,5-trimethoxybenzylidene)-1*H*-benzo[*d*]imidazole-5-carbohydrazide (**8g**)

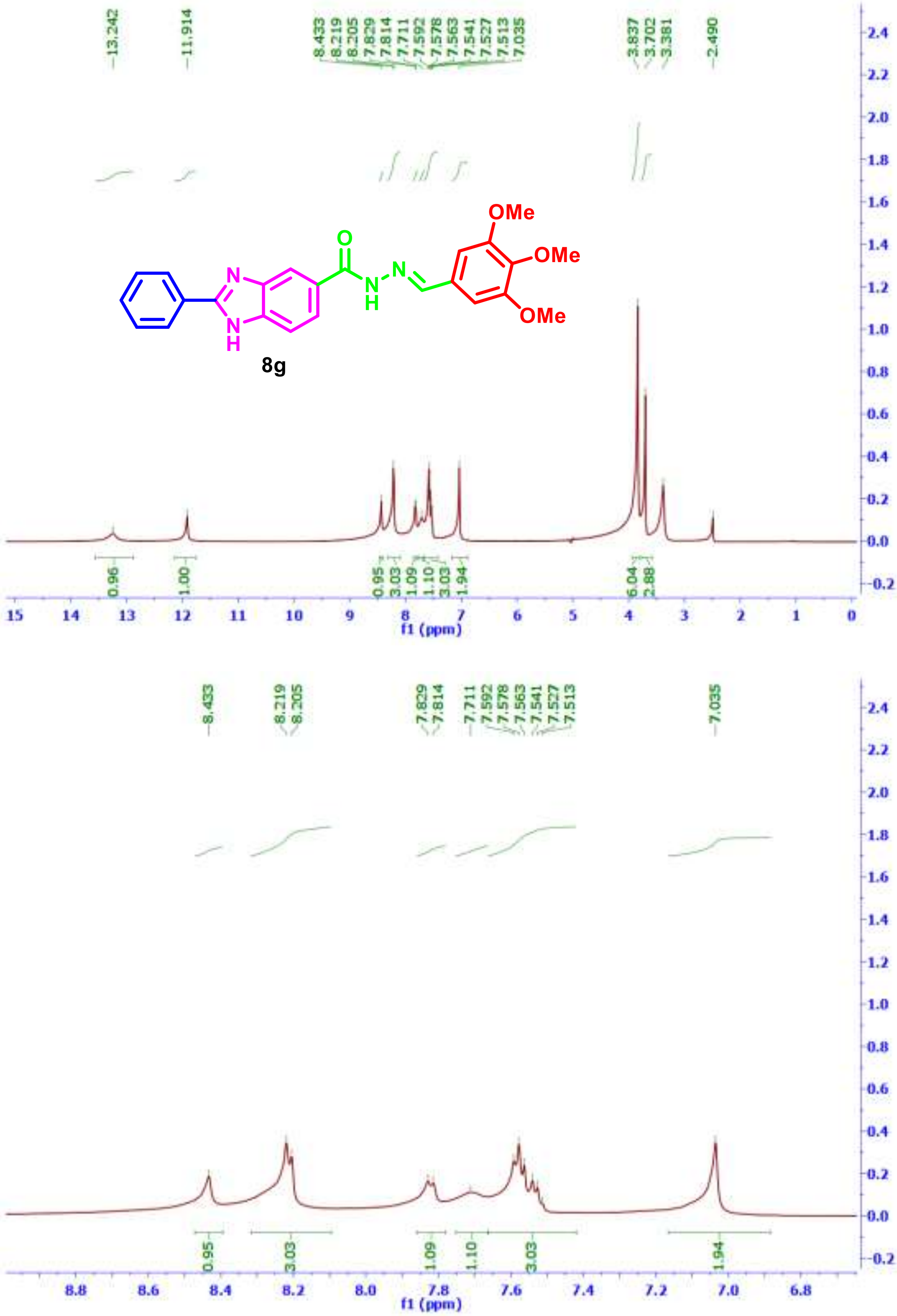

Figure S13. ^{1}H (500 MHz) NMR spectrum of **8g** in DMSO-d_6

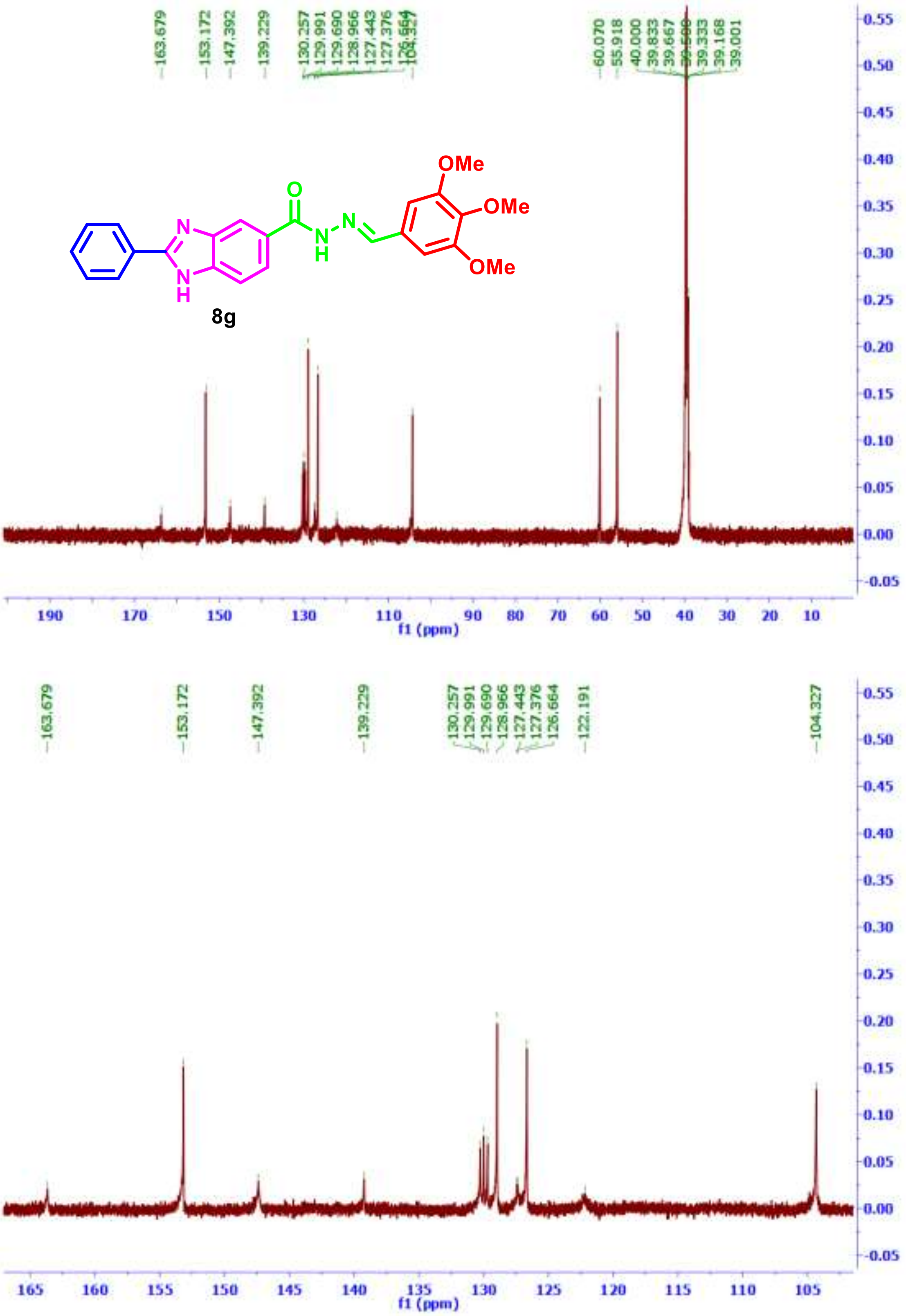

Figure S14. ^{13}C (125 MHz) NMR spectrum of **8g** in DMSO-d_6

N'-(2-Hydroxybenzylidene)-2-(4-methoxyphenyl)-1*H*-benzo[*d*]imidazole-5-carbohydrazide (**8h**)

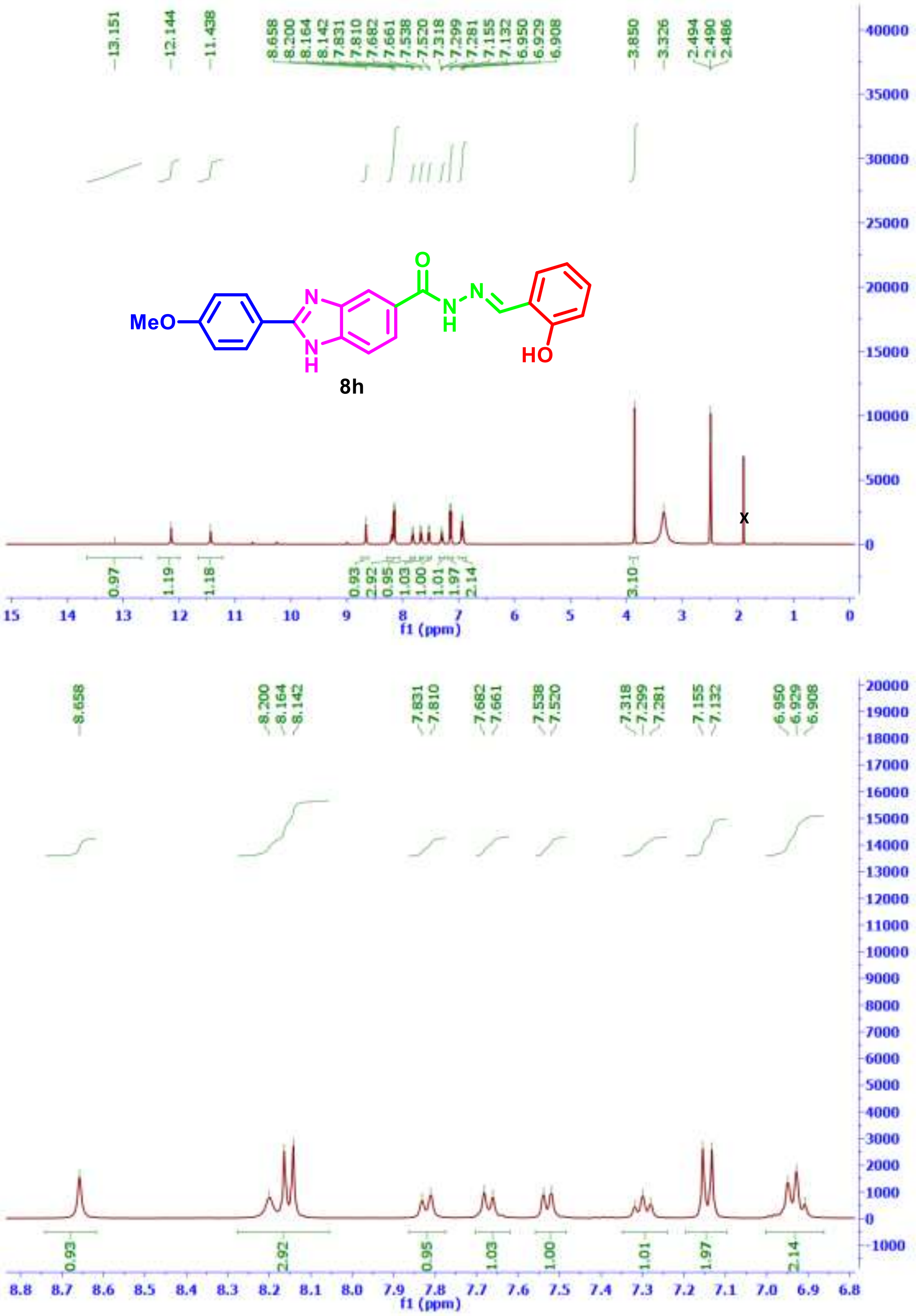

Figure S15. ^{1}H (400 MHz) NMR spectrum of **8h** in DMSO-d_6

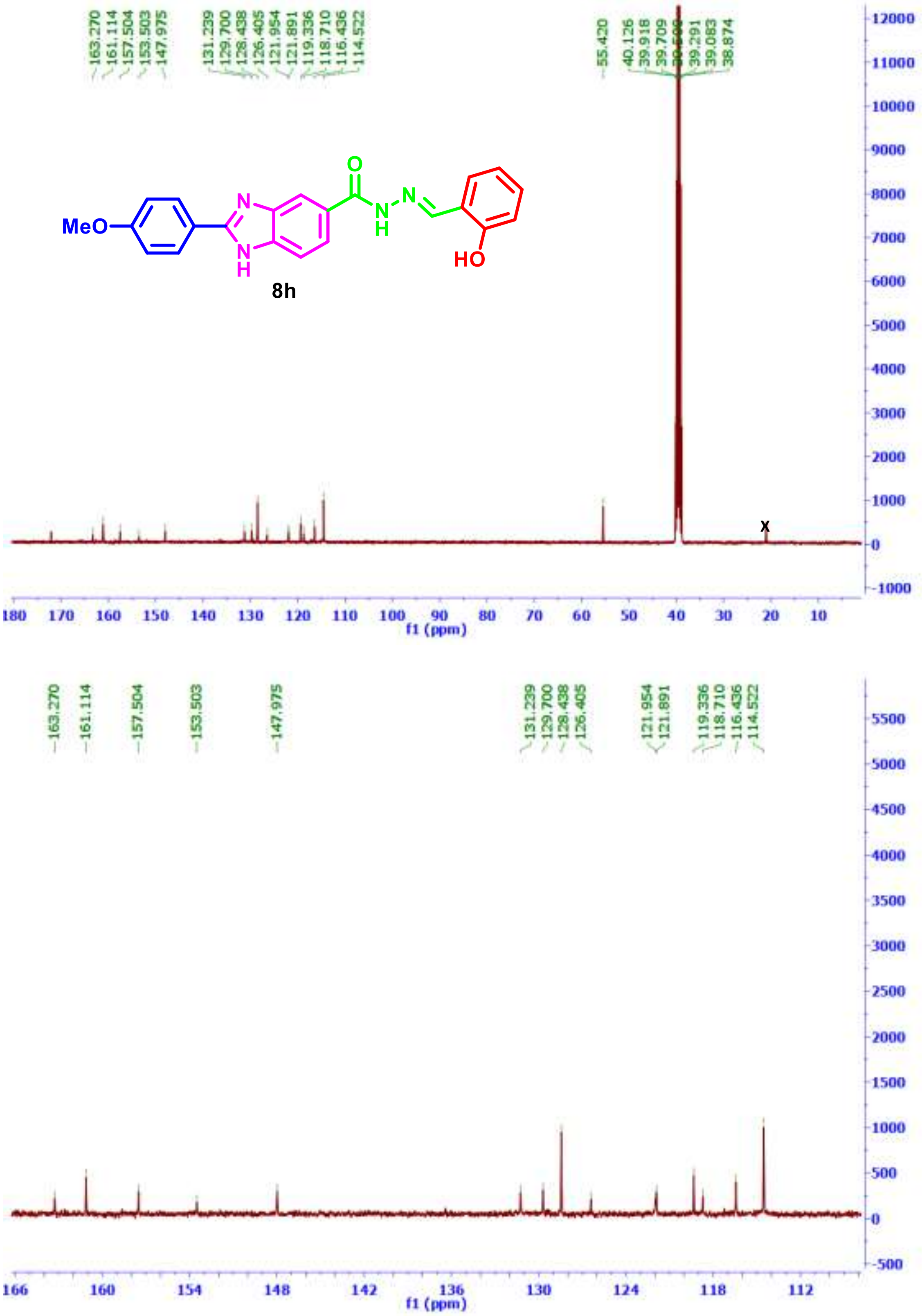

Figure S16. ^{13}C (100 MHz) NMR spectra of **8h** in DMSO-d_6

N'-(3-Hydroxybenzylidene)-2-(4-methoxyphenyl)-1*H*-benzo[*d*]imidazole-5-carbohydrazide (**8i**)

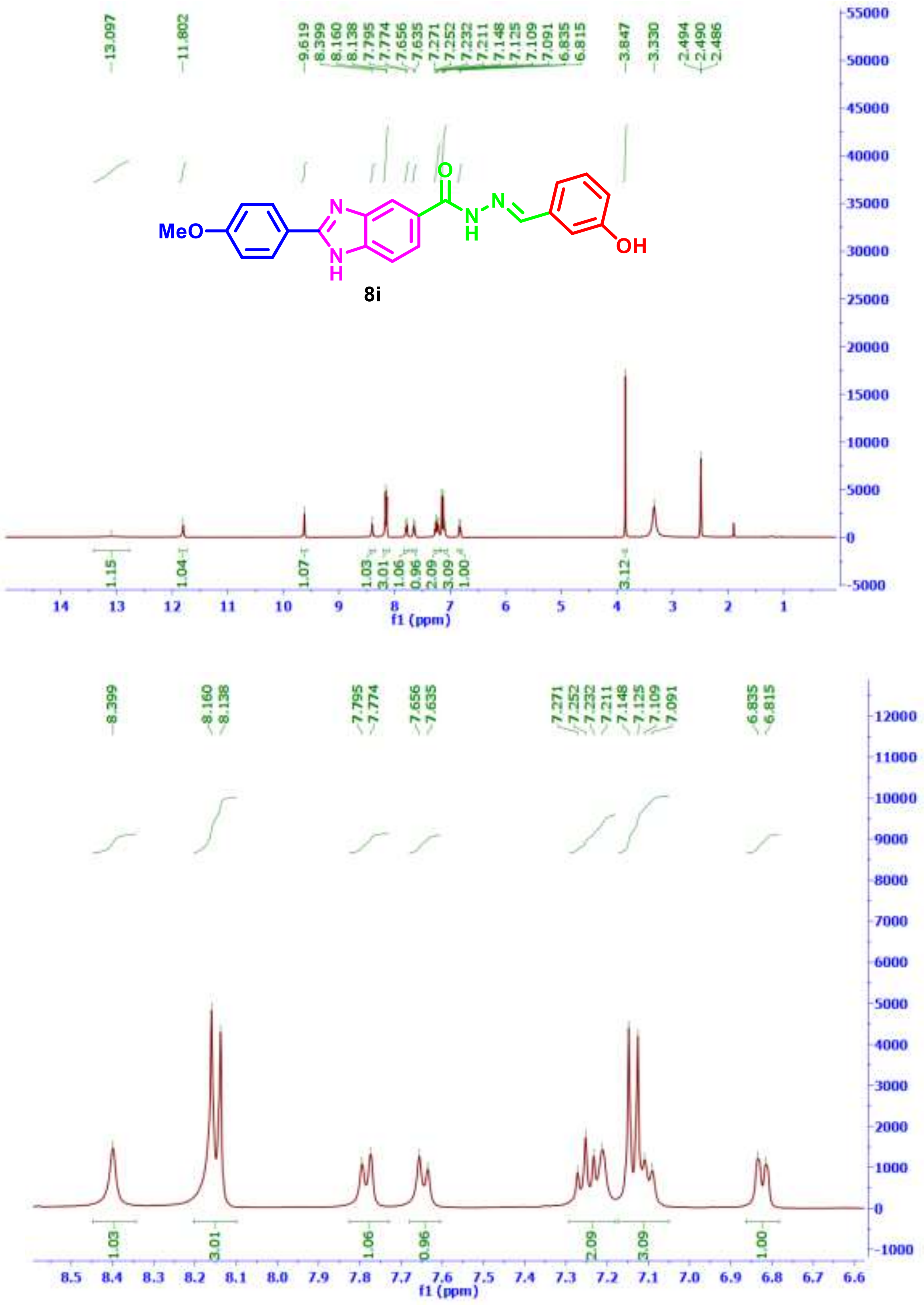

Figure S17. ^{1}H (400 MHz) NMR spectrum of **8i** in DMSO-d_6

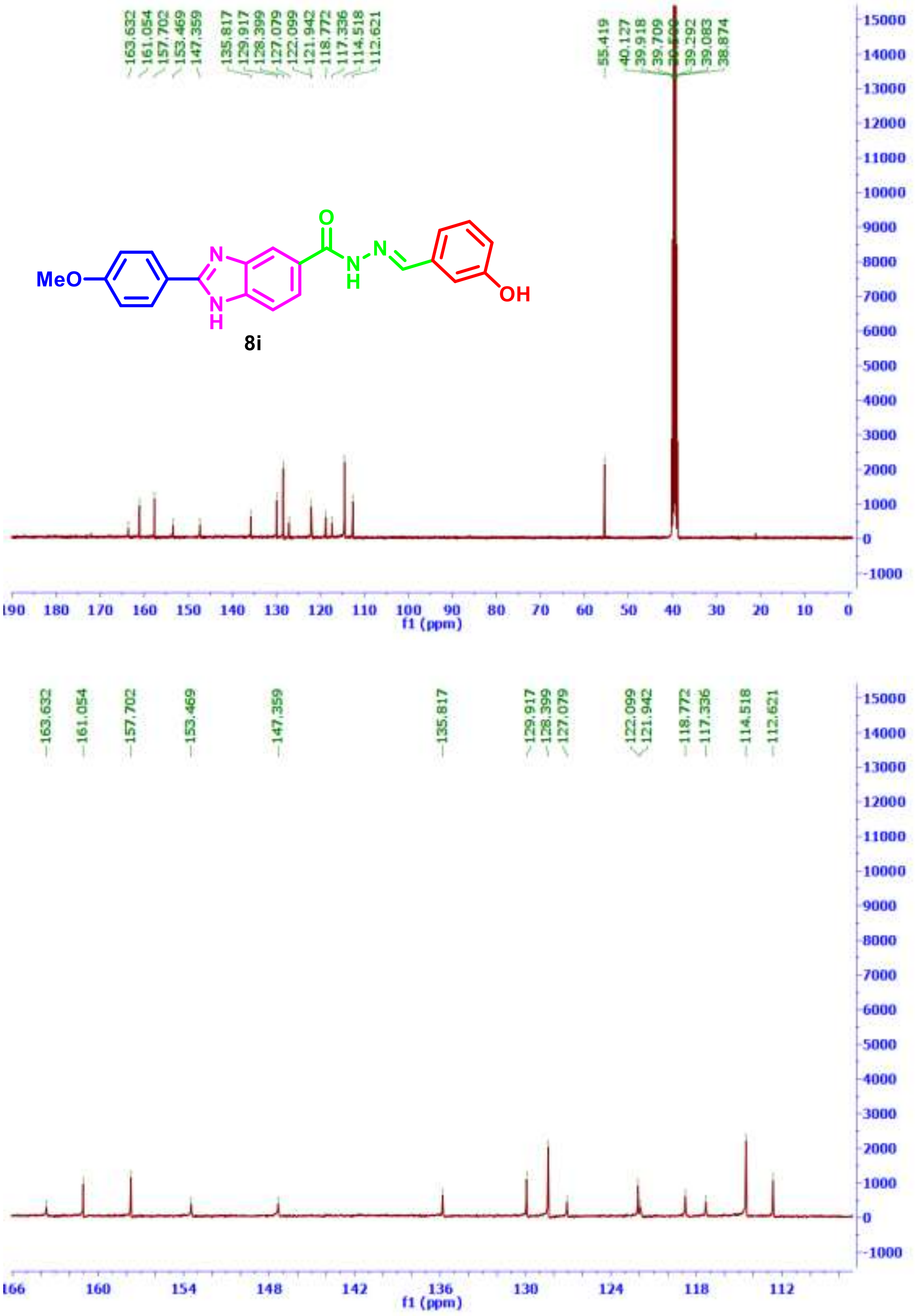

Figure S18. ^{13}C (100 MHz) NMR spectrum of **8i** in DMSO-d_6

N'-(3-Methoxybenzylidene)-2-(4-methoxyphenyl)-1*H*-benzo[*d*]imidazole-5-carbohydrazide (**8j**)

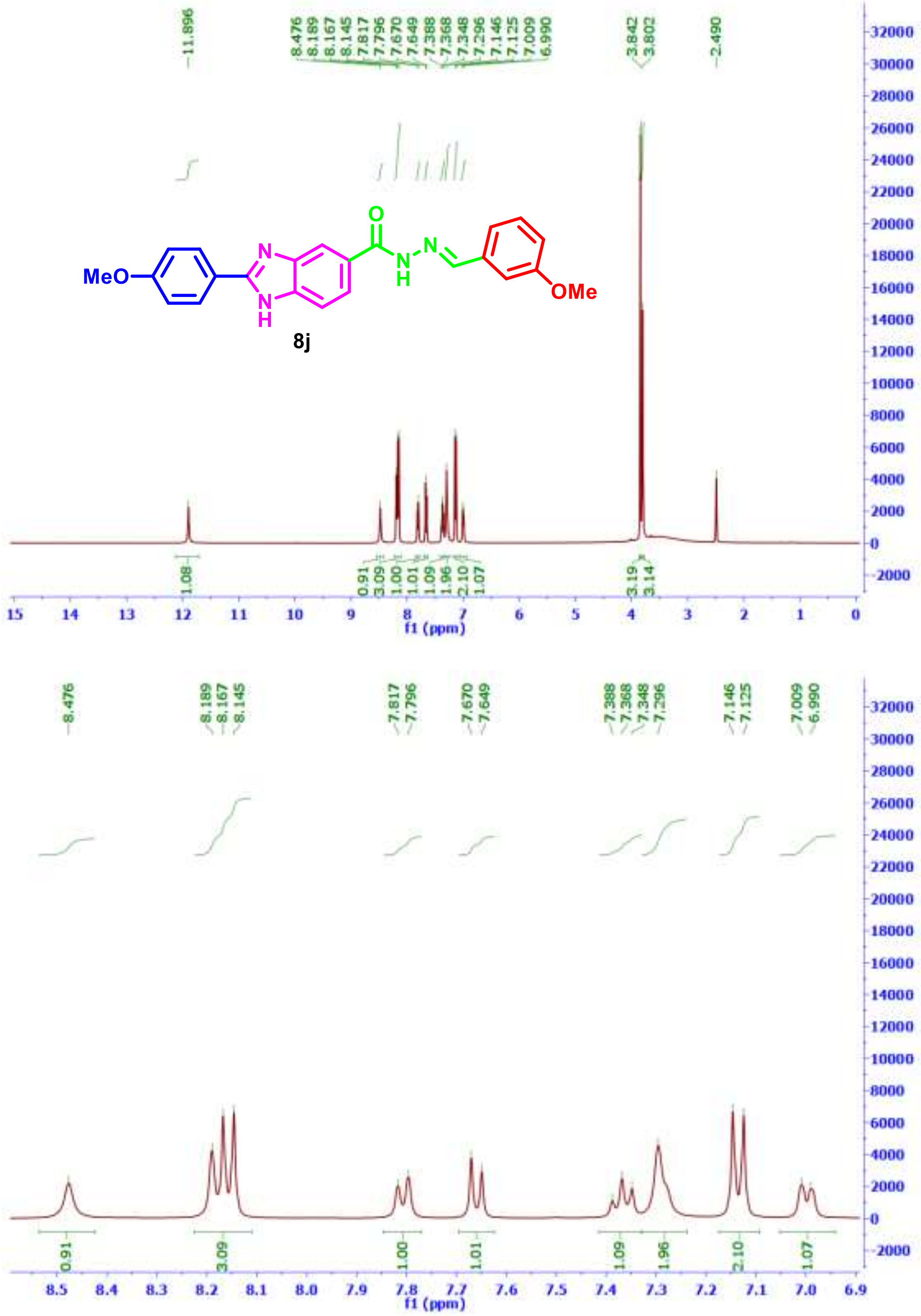

Figure S19. ^{1}H (400 MHz) NMR spectrum of **8j** in DMSO-d_6

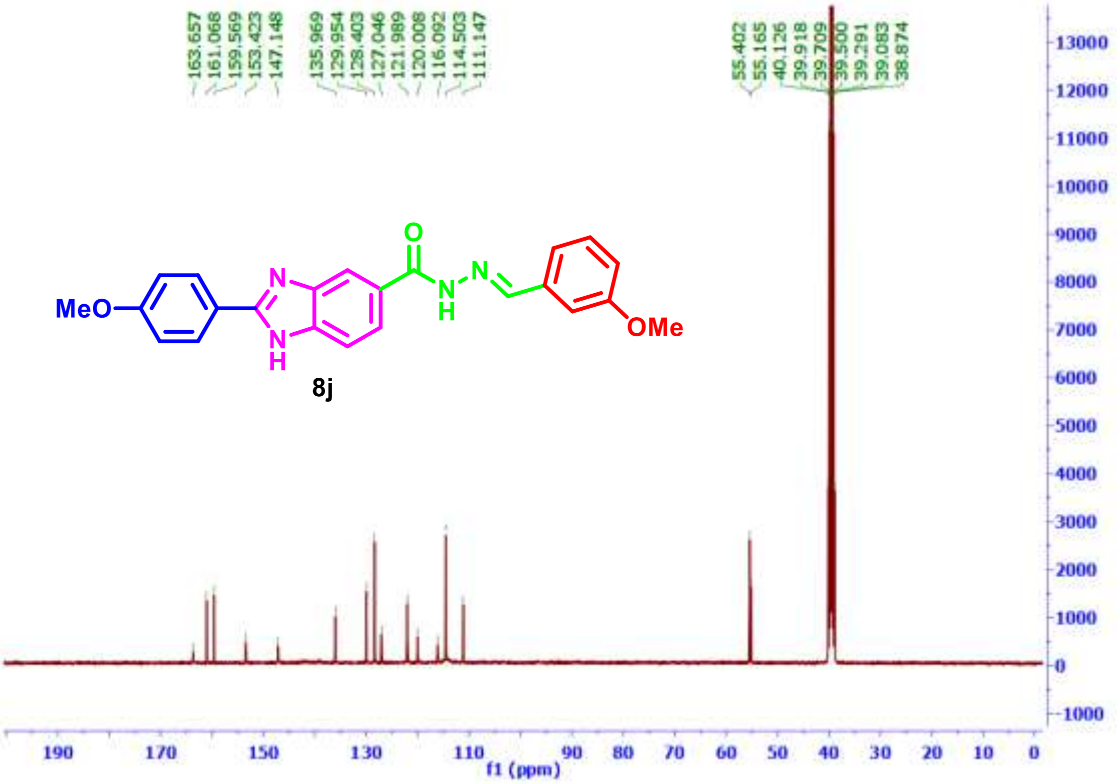

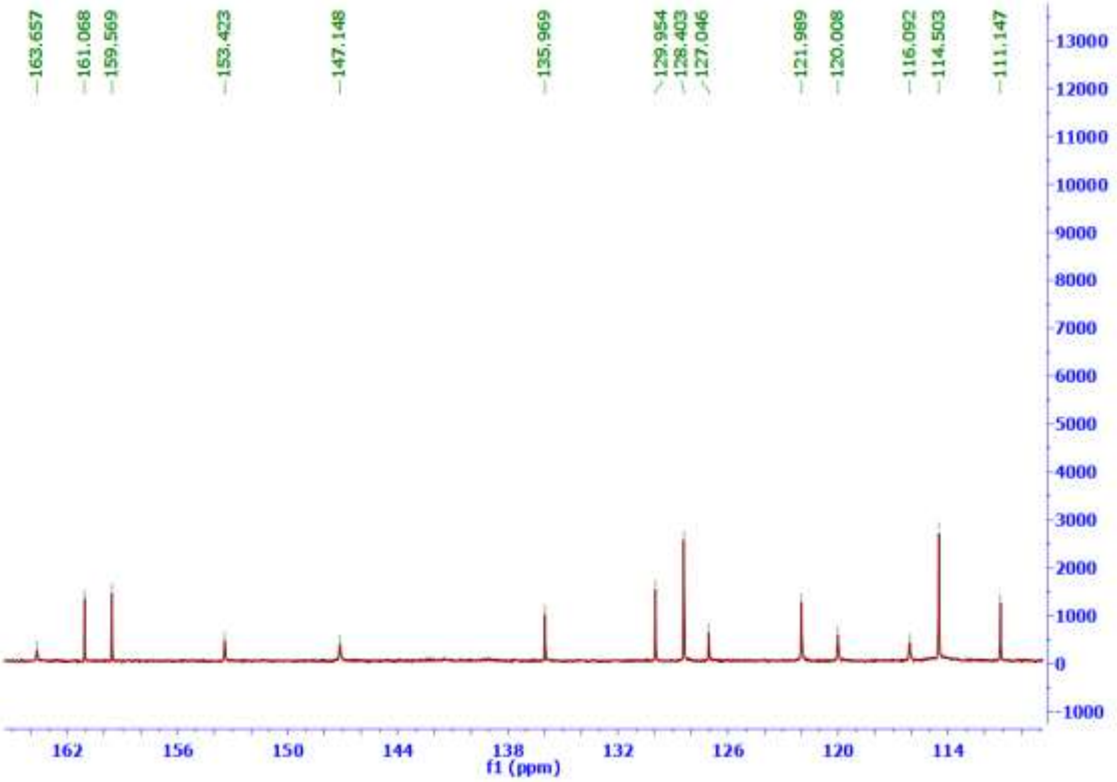

Figure S20. ^{13}C (100 MHz) NMR spectrum of **8j** in DMSO-d_6

N'-(2-Hydroxy-3-methoxybenzylidene)-2-(4-methoxyphenyl)-1*H*-benzo[*d*]imidazole-5-carbohydrazide (**8k**)

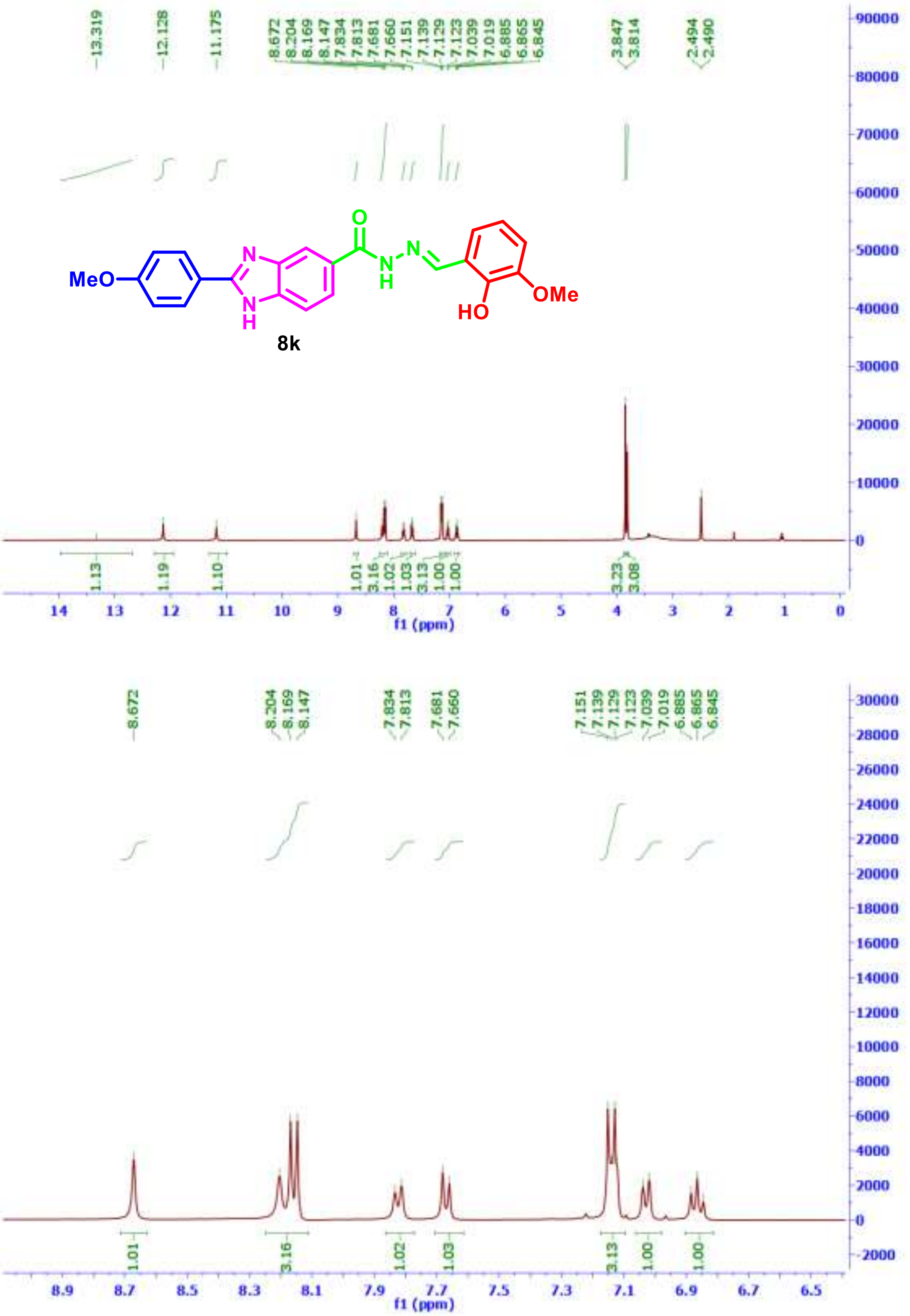

Figure S21. ^{1}H (400 MHz) NMR spectrum of **8k** in DMSO-d_6

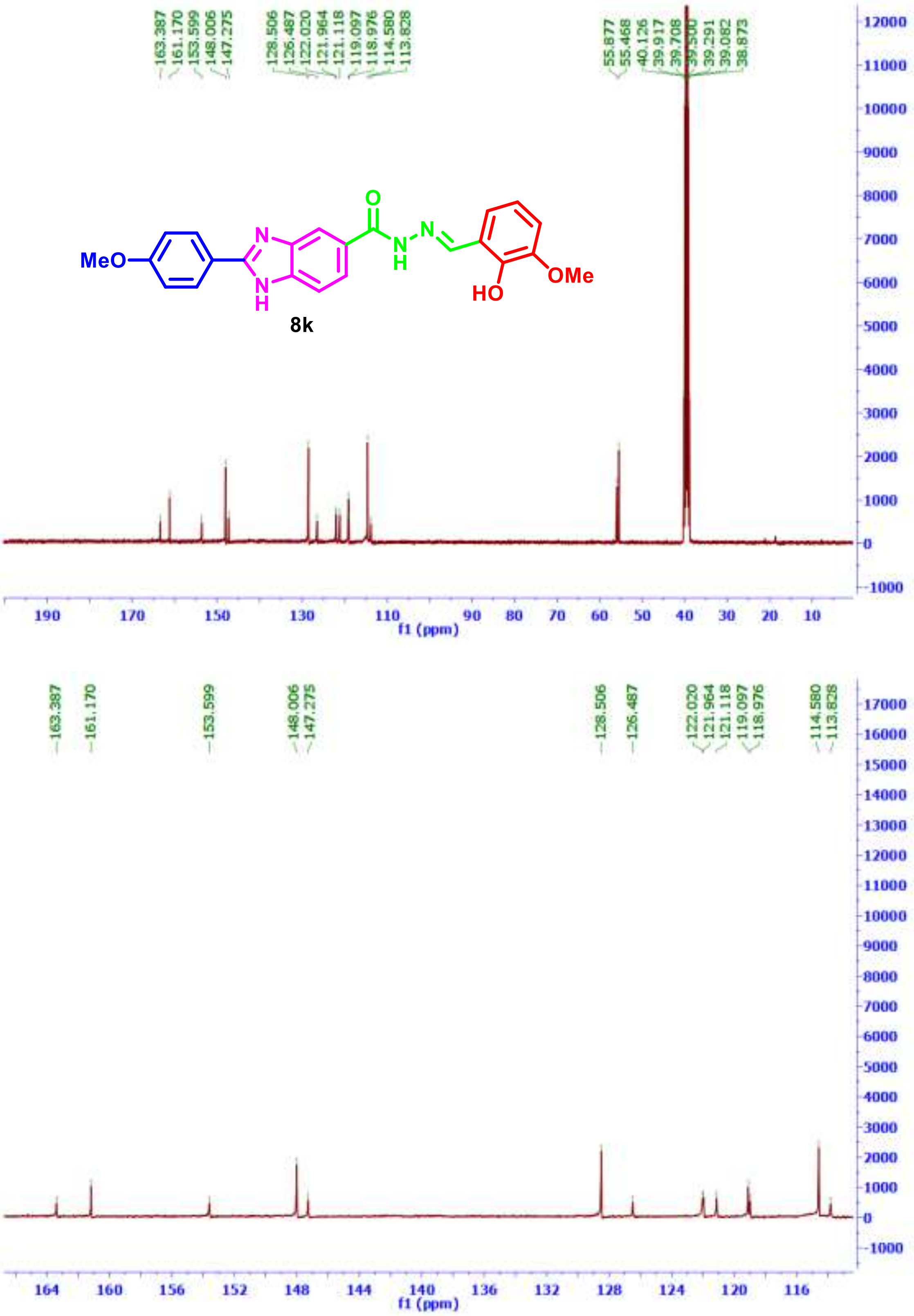

Figure S22. ^{13}C (100 MHz) NMR spectrum of **8k** in DMSO-d_6

N'-(3-Hydroxy-4-methoxybenzylidene)-2-(4-methoxyphenyl)-1*H*-benzo[*d*]imidazole-5-carbohydrazide (**8l**)

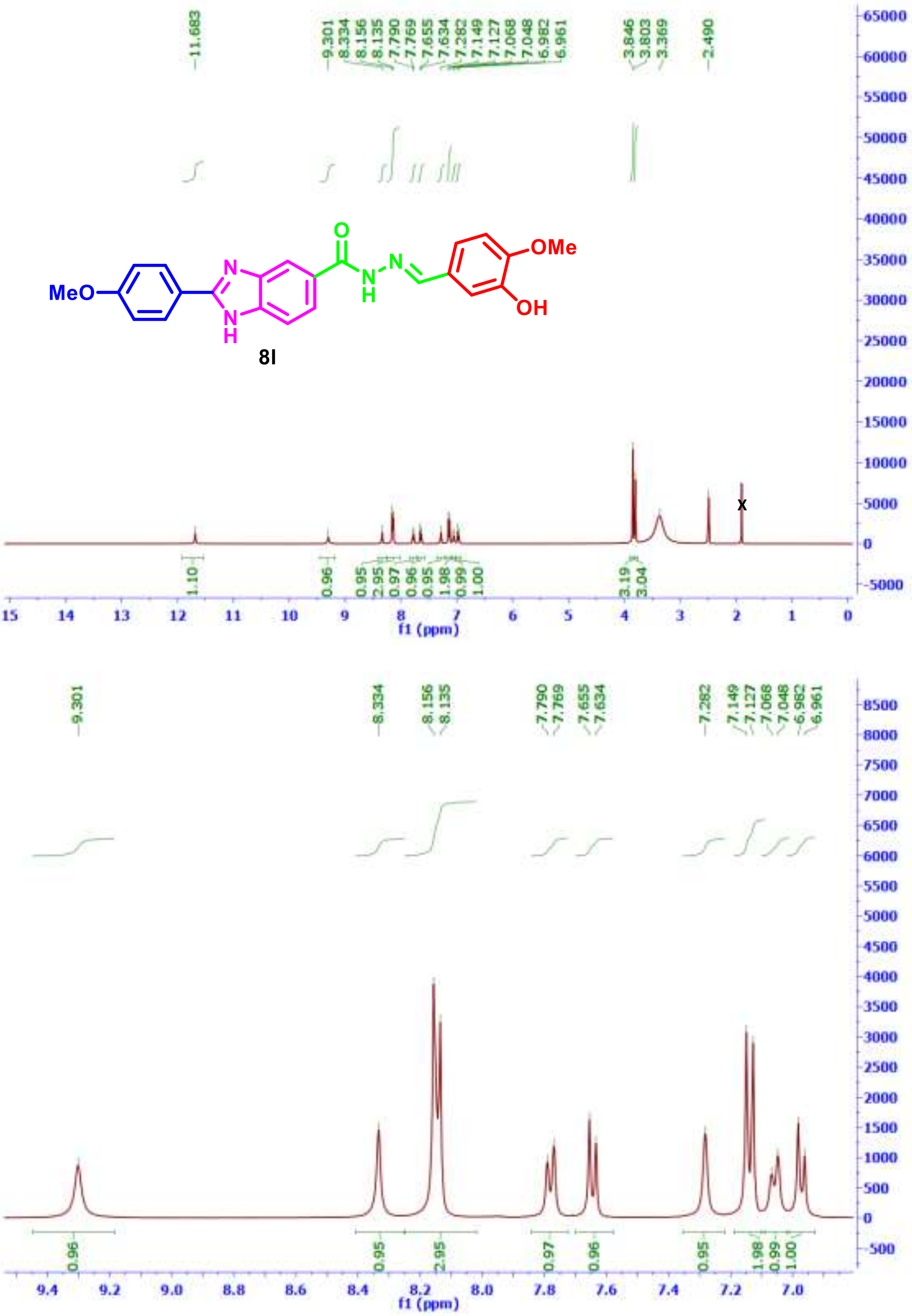

Figure S23. ^{1}H (400 MHz) NMR spectrum of **8l** in DMSO-d_6

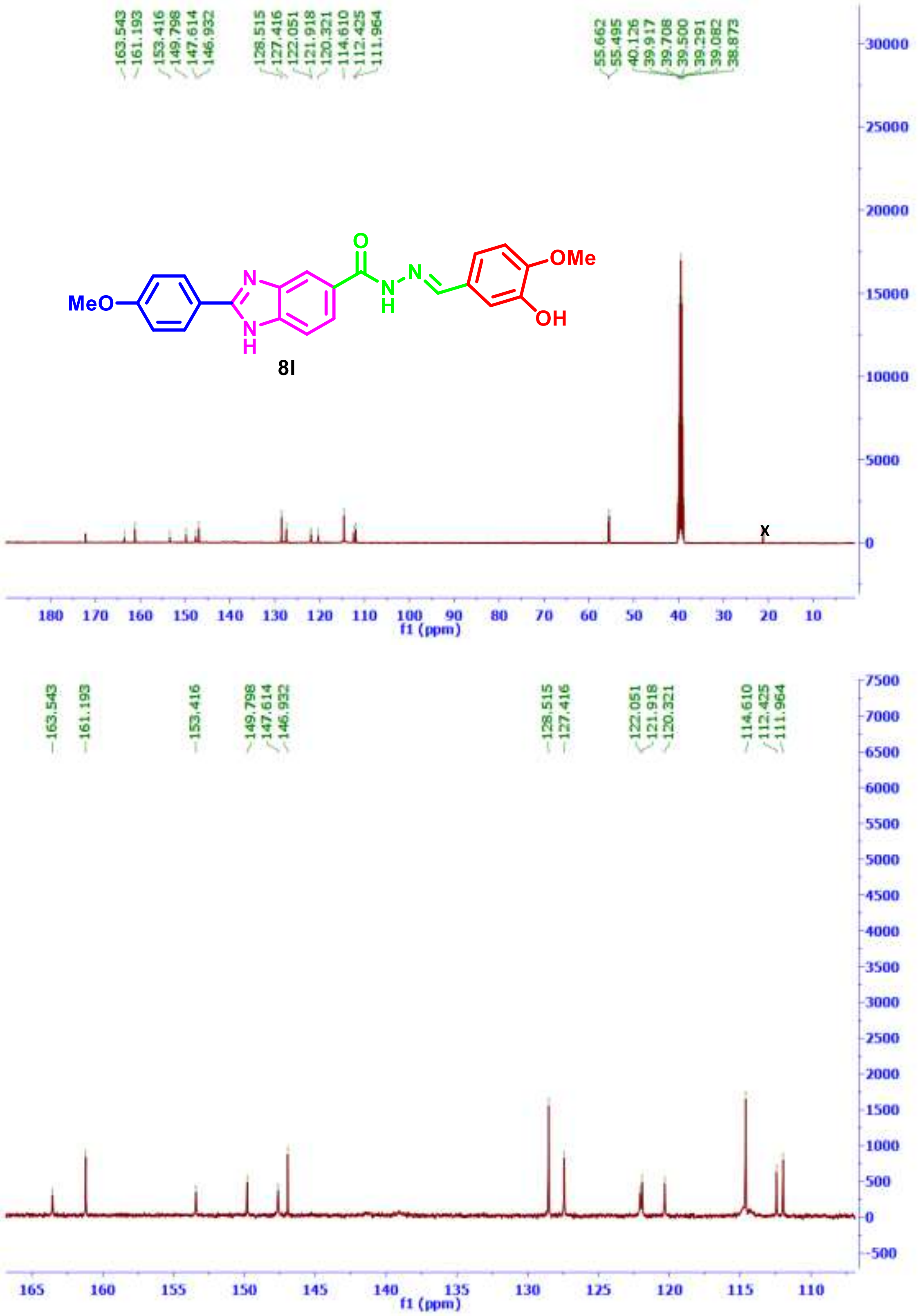

Figure S24. ^{13}C (100 MHz) NMR spectrum of **8l** in DMSO-d_6

N'-(2,5-Dimethoxybenzylidene)-2-(4-methoxyphenyl)-1*H*-benzo[*d*]imidazole-5-carbohydrazide (**8m**)

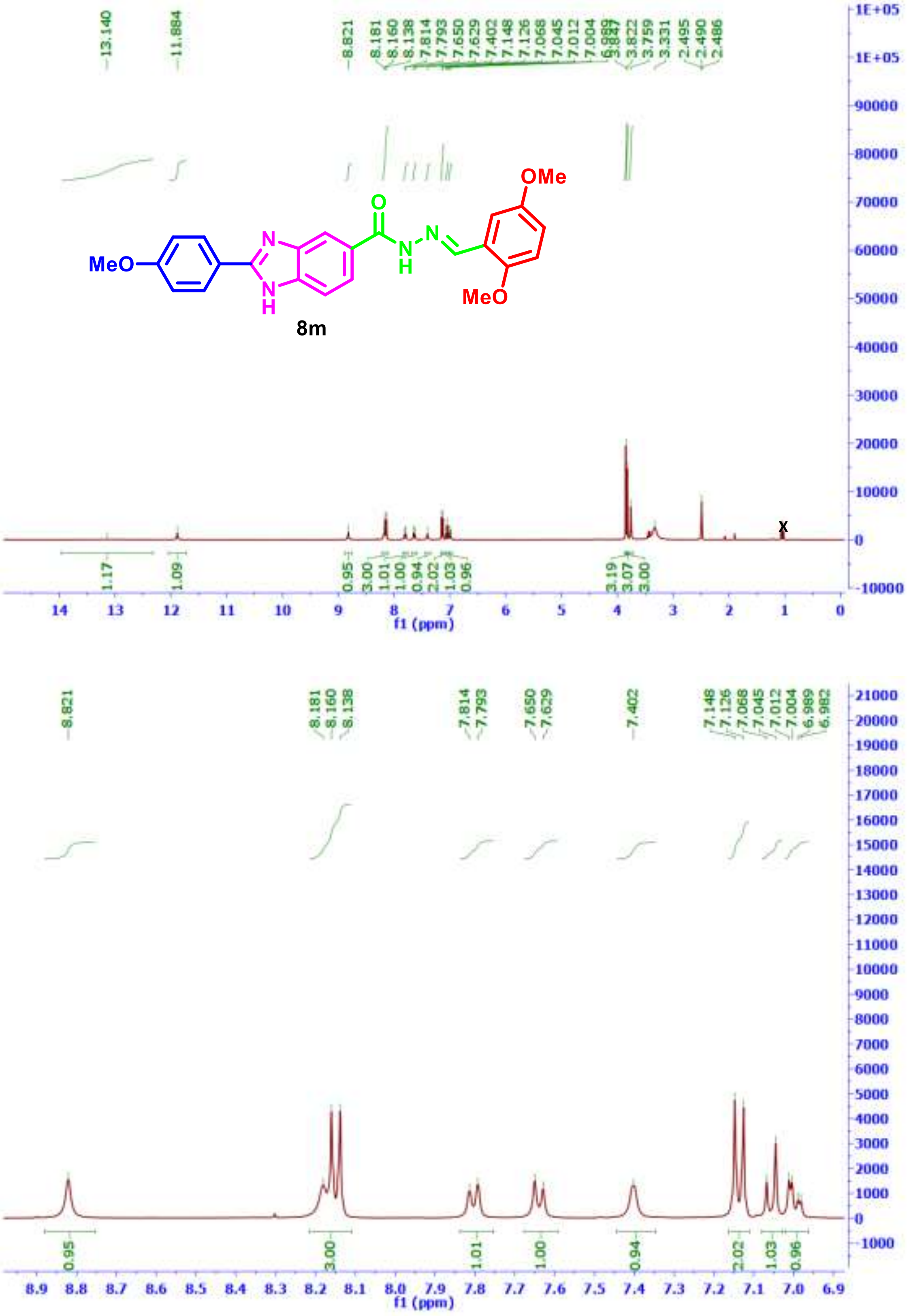

Figure S25. ^{1}H (400 MHz) NMR spectrum of **8m** in DMSO-d_6

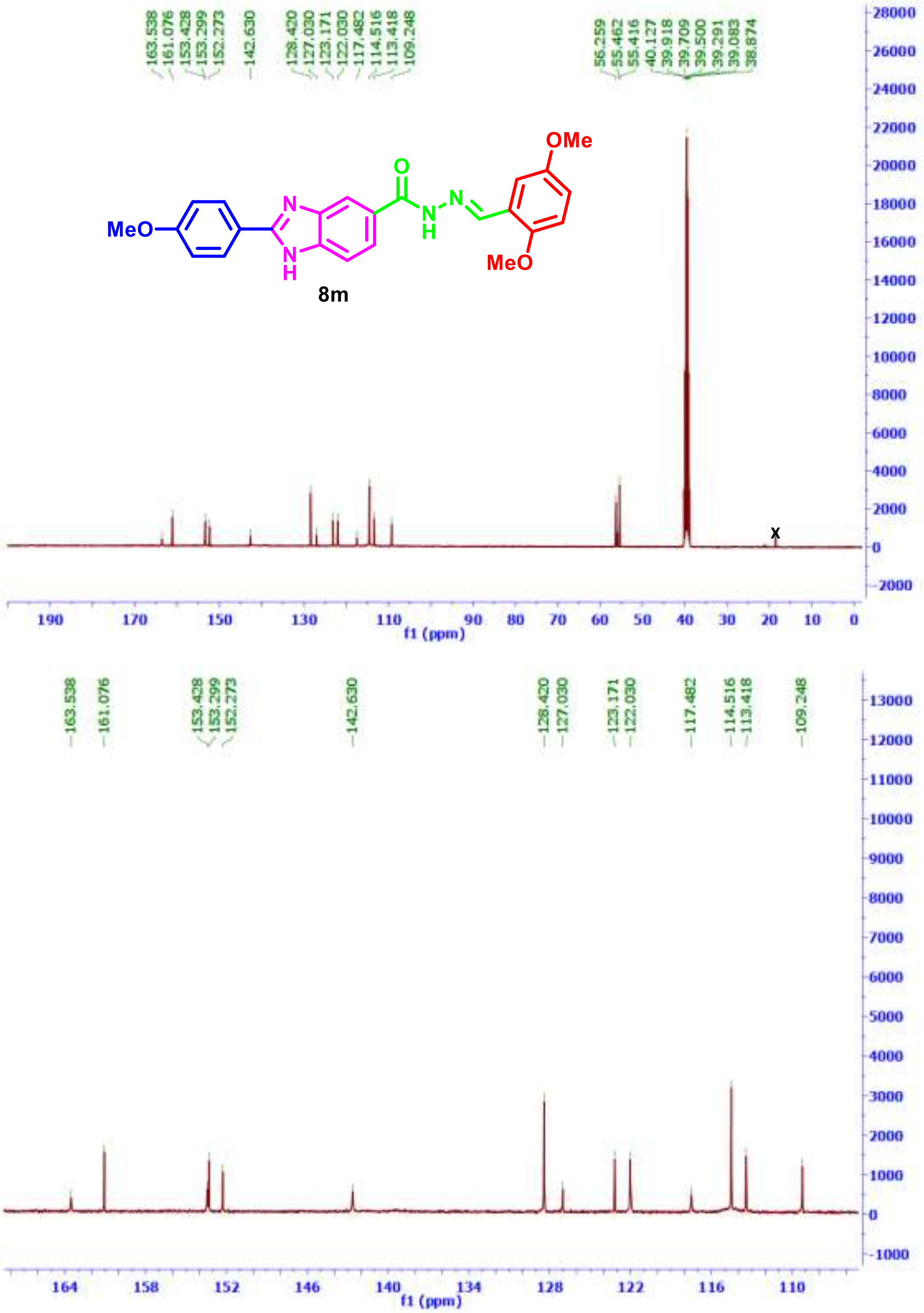

Figure S26. ^{13}C (100 MHz) NMR spectrum of **8m** in DMSO-d_6

2-(4-Methoxyphenyl)-*N*'-(3,4,5-trimethoxybenzylidene)-1*H*-benzo[*d*]imidazole-5-carbohydrazide (**8n**)

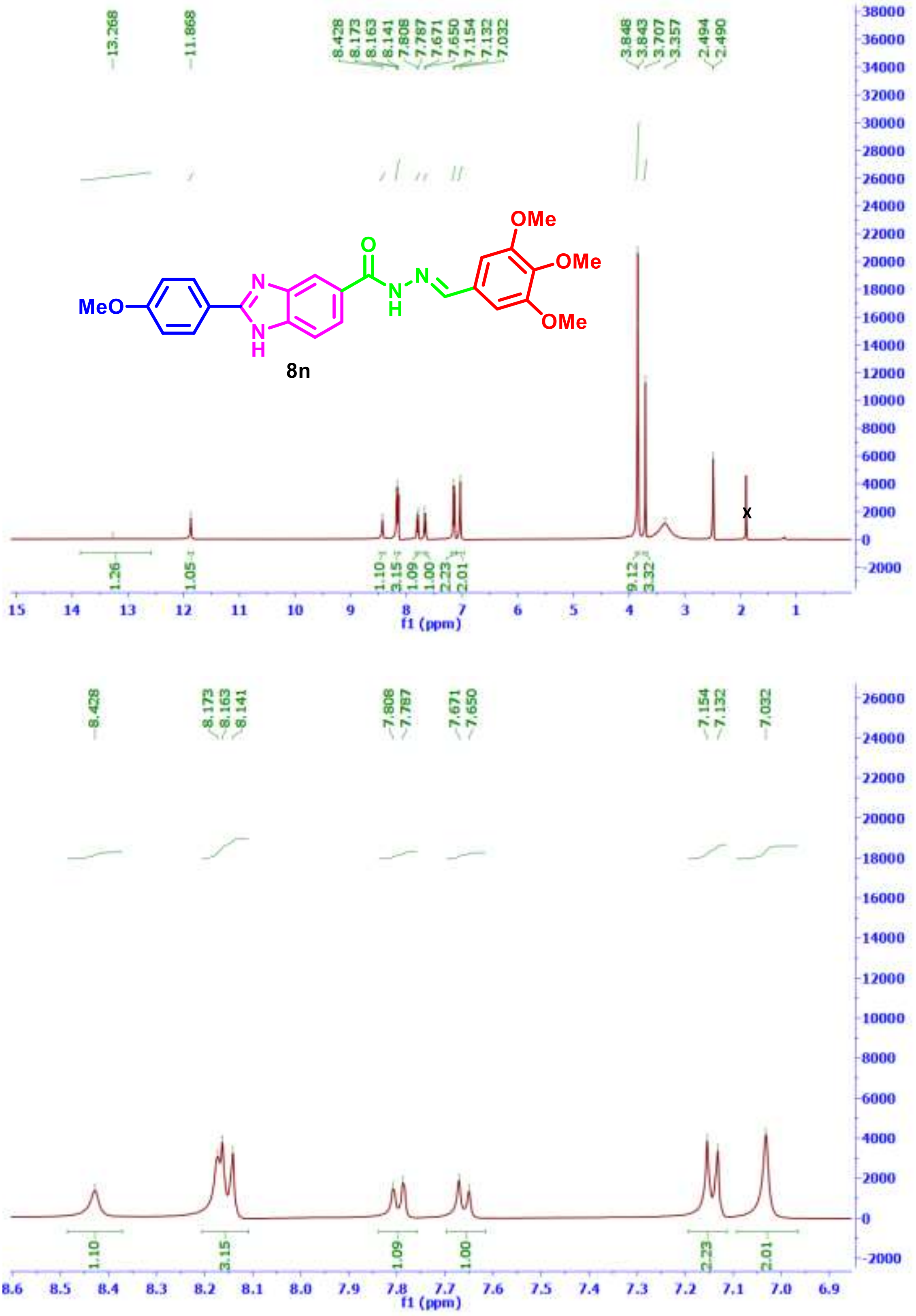

Figure S27. ^{1}H (400 MHz) NMR spectrum of **8n** in DMSO-d_6

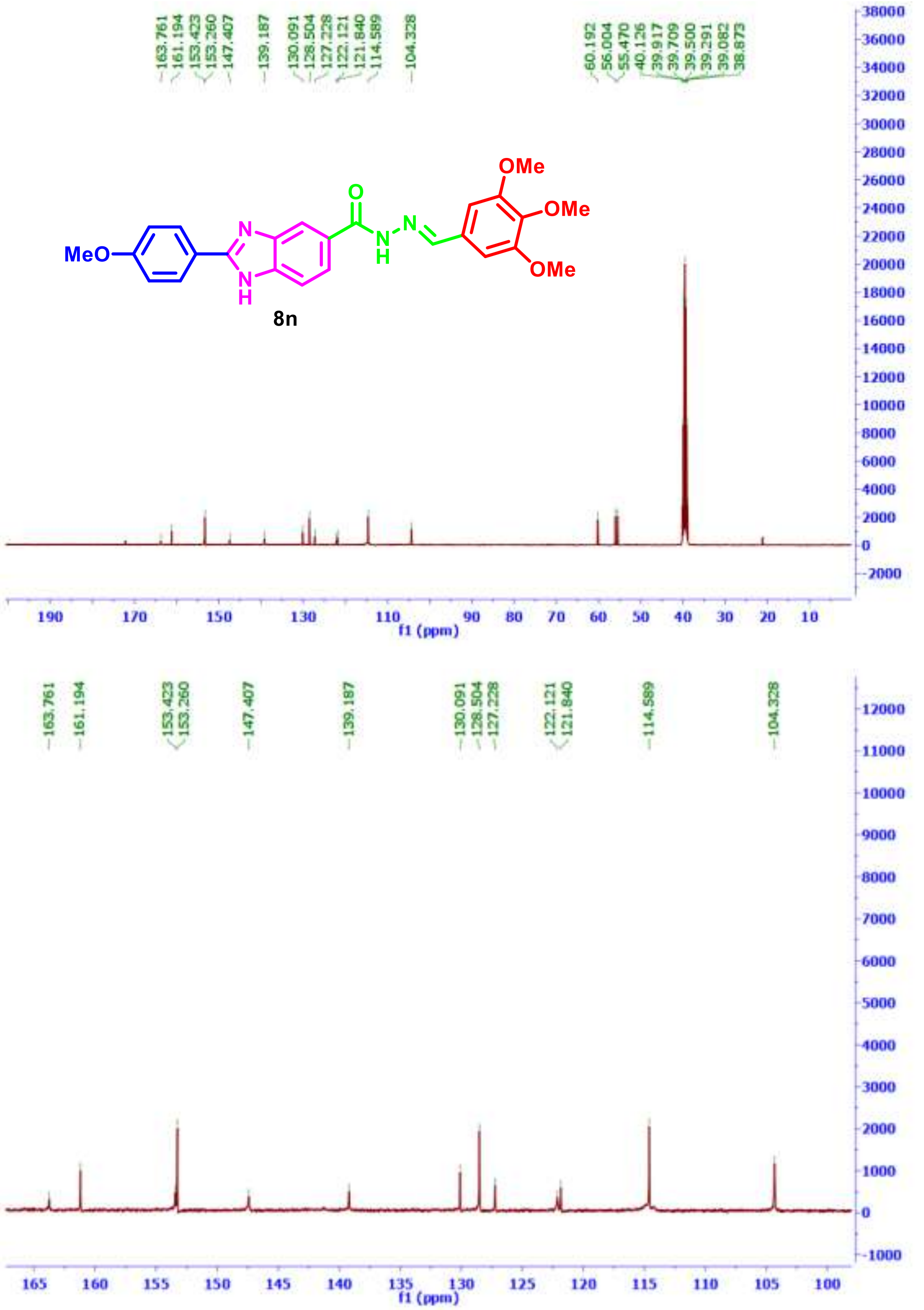

Figure S28. ^{13}C (100 MHz) NMR spectrum of **8n** in DMSO-d_6

2-(4-Chlorophenyl)-*N'*-(2-hydroxybenzylidene)-1*H*-benzo[*d*]imidazole-5-carbohydrazide (**8o**)

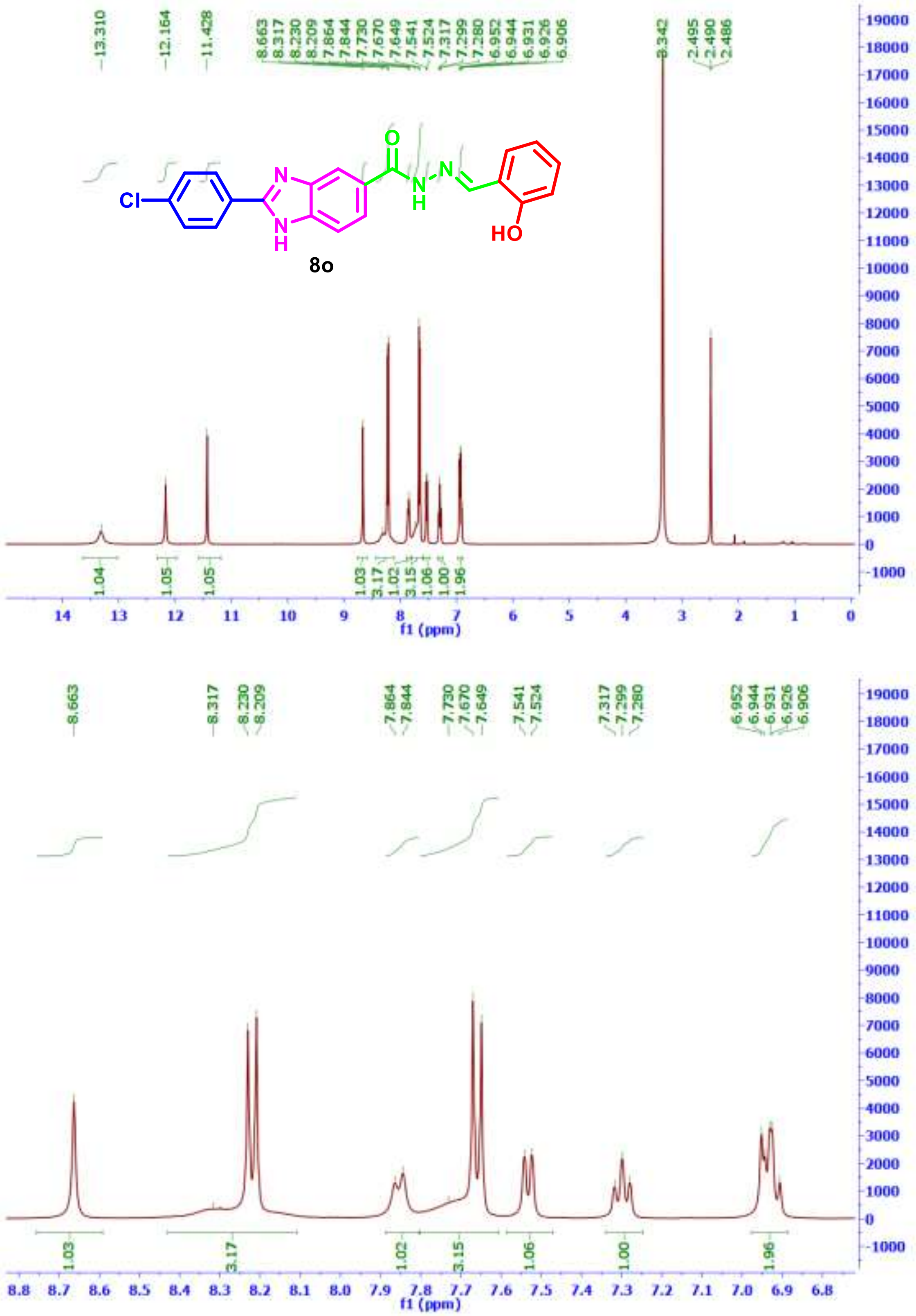

Figure S29. ^{1}H (400 MHz) NMR spectrum of **8o** in DMSO-d_6

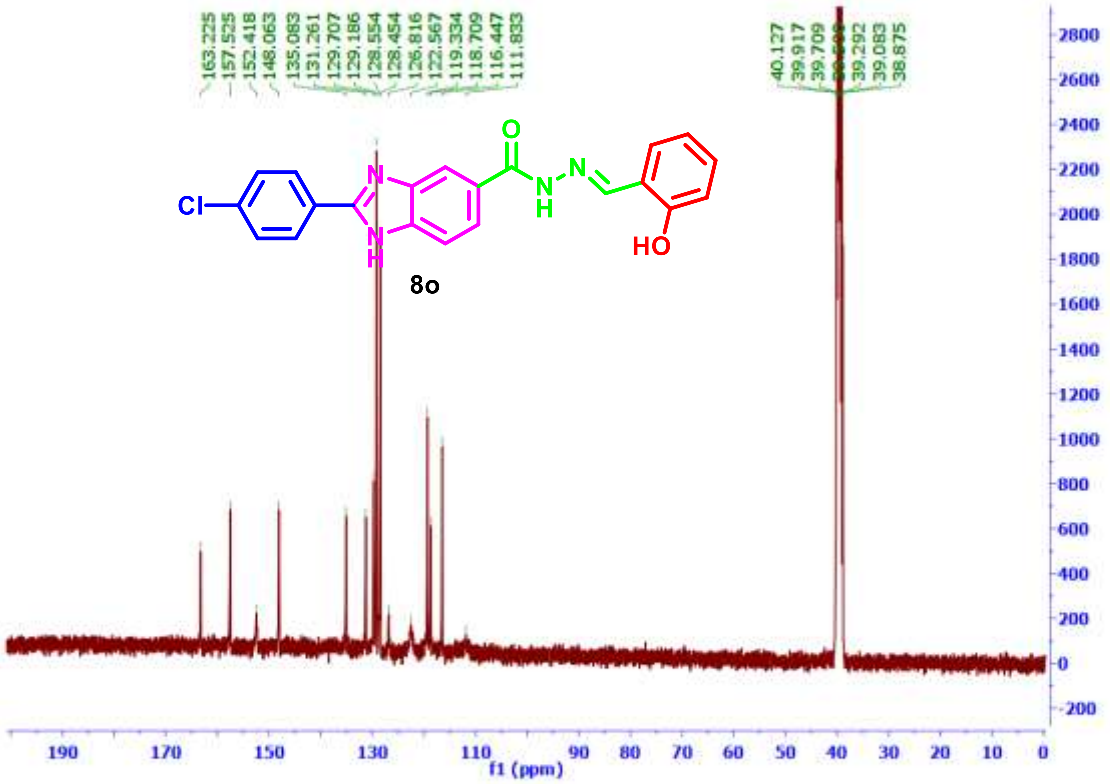

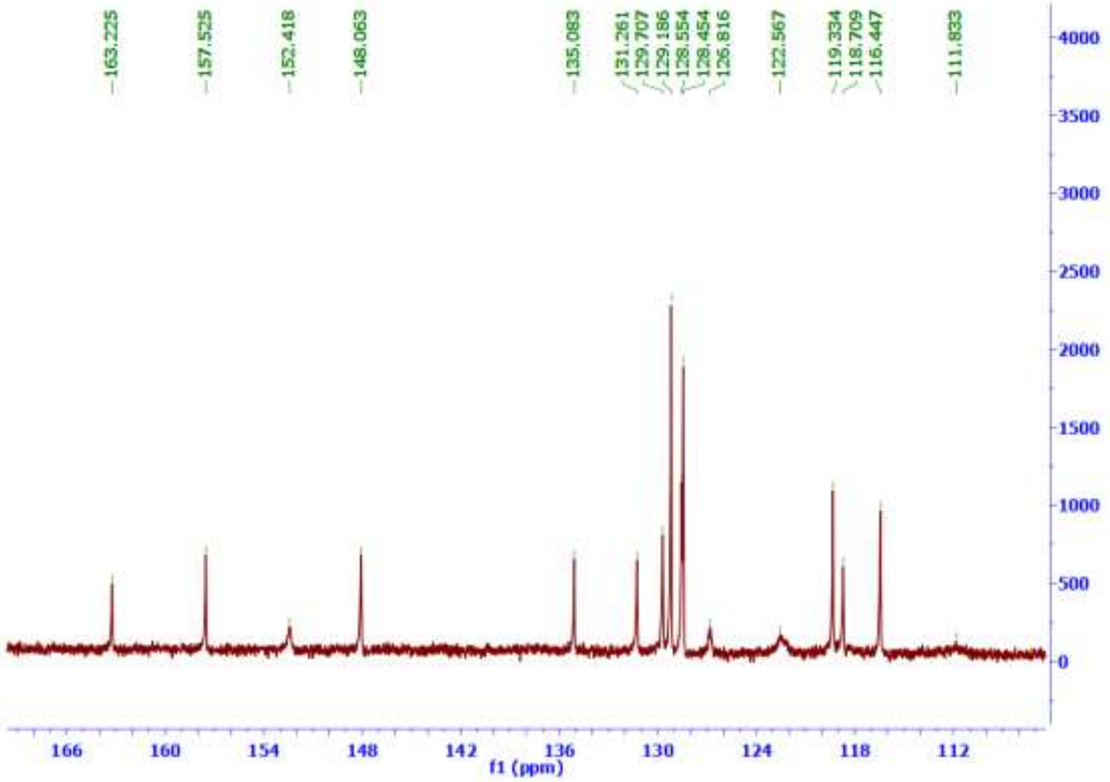

Figure S30. ^{13}C (100 MHz) NMR spectrum of **8o** in DMSO-d_6

2-(4-Chlorophenyl)-*N*'-(3-hydroxybenzylidene)-1*H*-benzo[*d*]imidazole-5-carbohydrazide (**8p**)

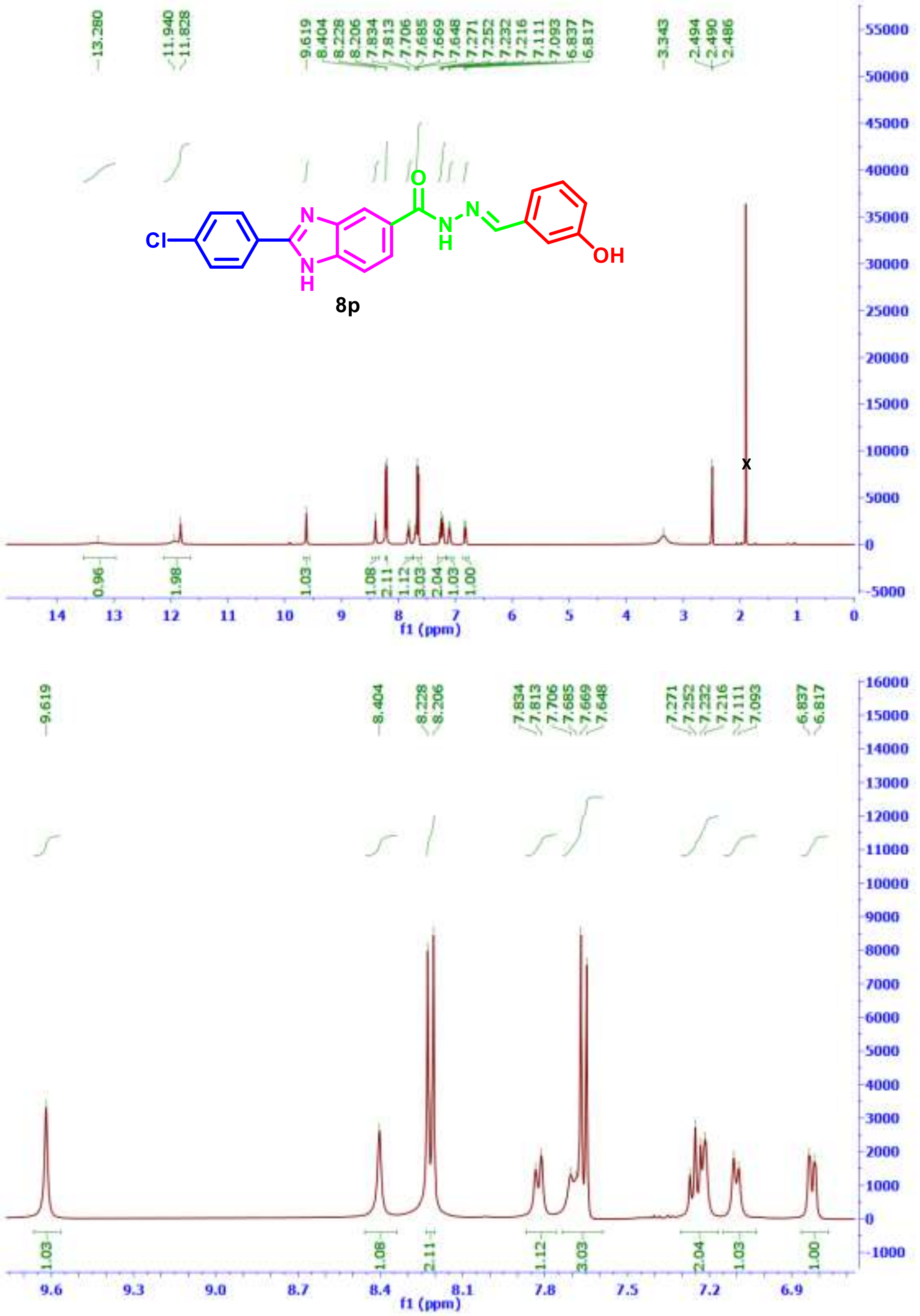

Figure S31. ^{1}H (400 MHz) NMR spectrum of **8p** in DMSO-d_6

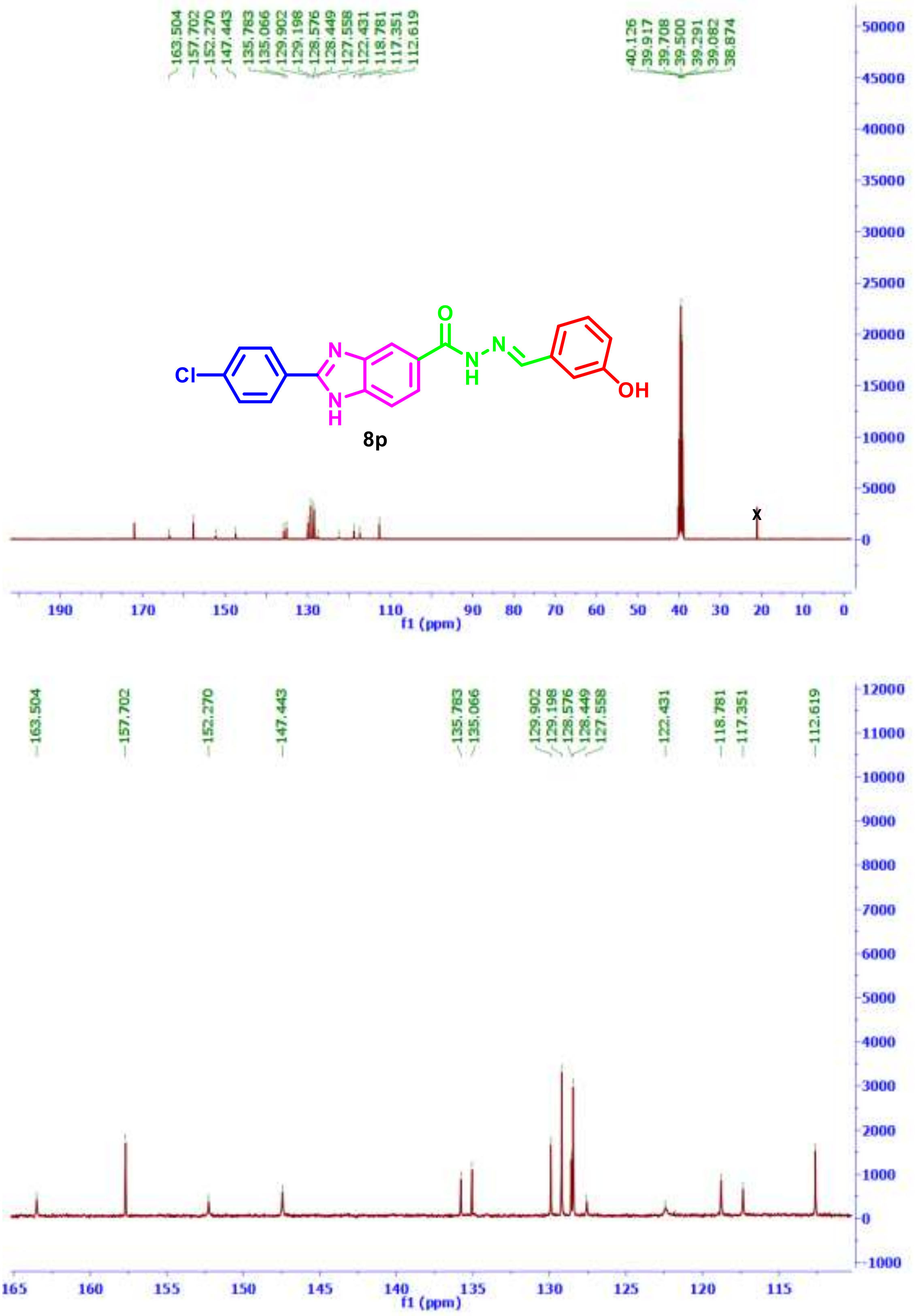

Figure S32. ^{13}C (100 MHz) NMR spectrum of **8p** in DMSO-d_6

2-(4-Chlorophenyl)-*N*'-(3-methoxybenzylidene)-1*H*-benzo[*d*]imidazole-5-carbohydrazide (**8q**)

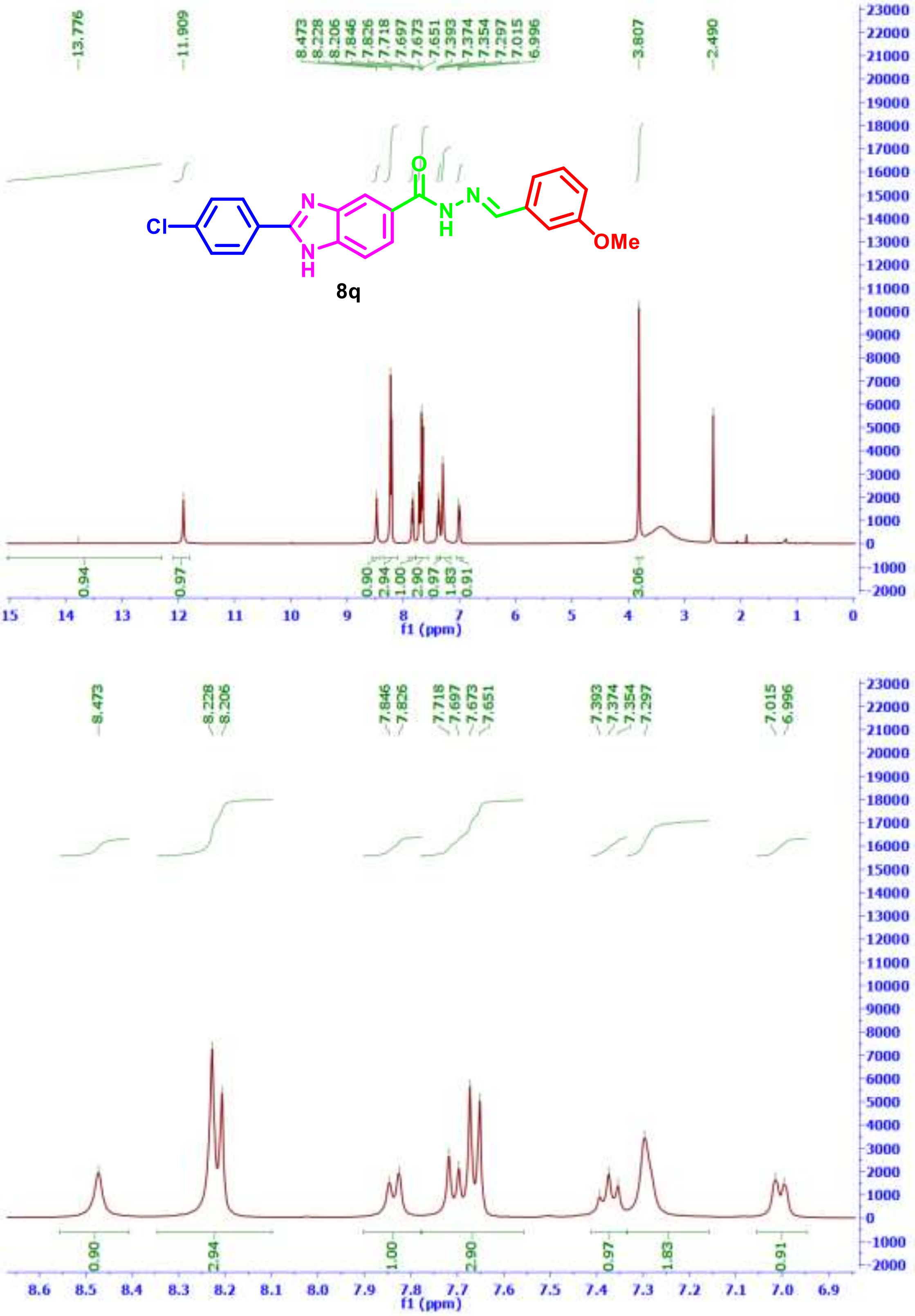

Figure S33. ^{1}H (400 MHz) NMR spectrum of **8q** in DMSO-d_6

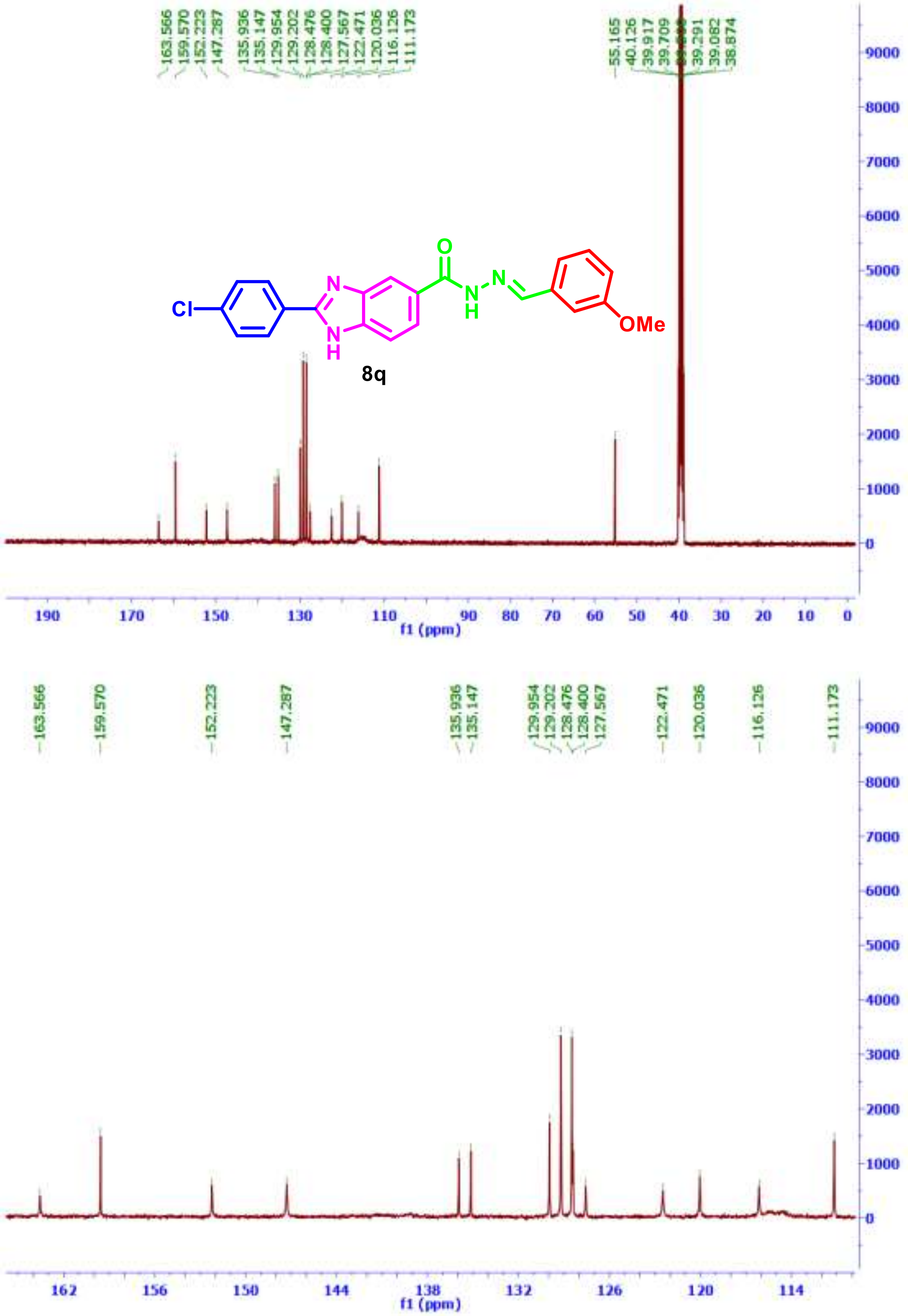

Figure S34. ^{13}C (100 MHz) NMR spectrum of **8q** in DMSO-d_6

2-(4-Chlorophenyl)-*N*'-(2-hydroxy-3-methoxybenzylidene)-1*H*-benzo[*d*]imidazole-5-carbohydrazide (**8r**)

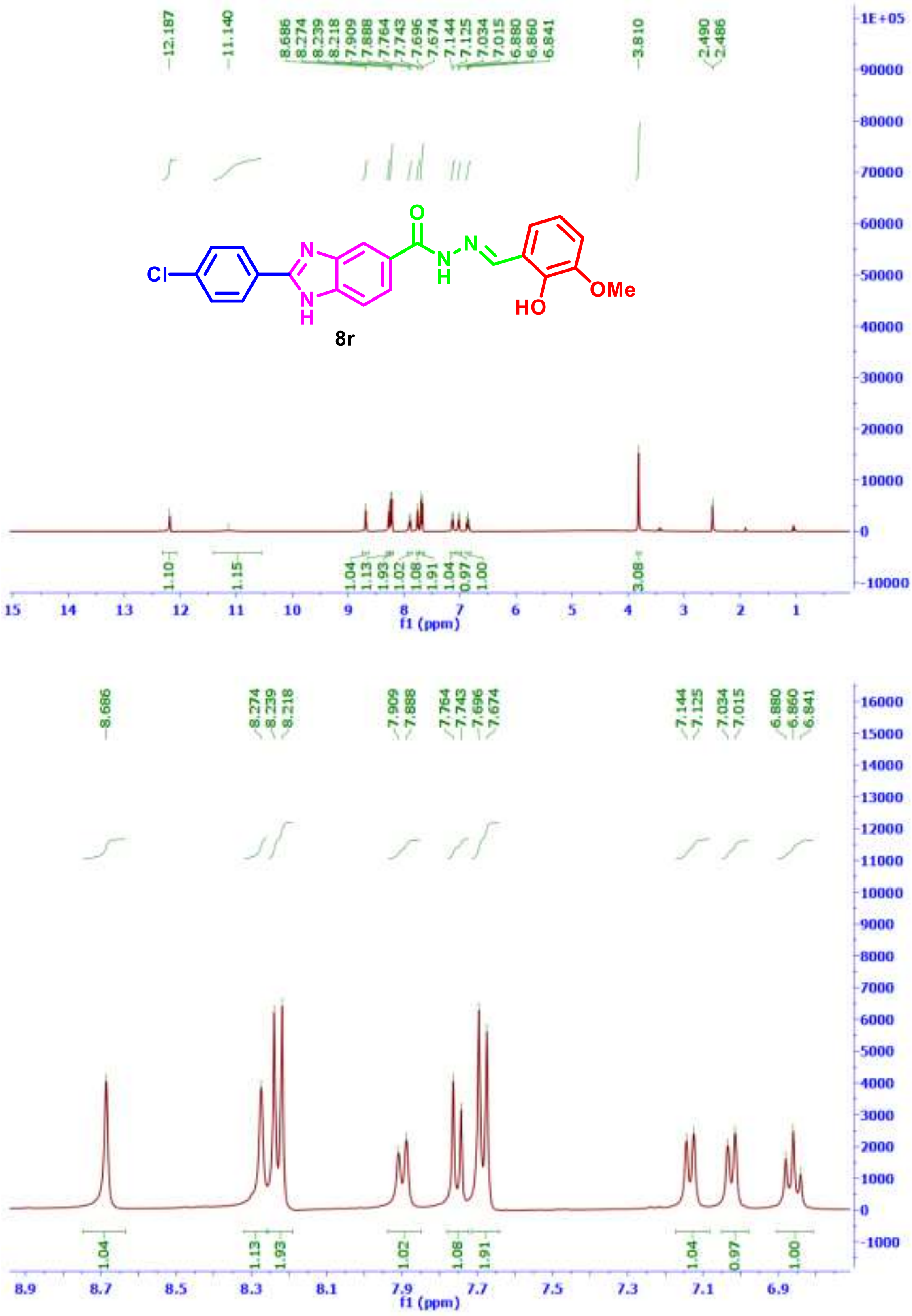

Figure S35. ^{1}H (400 MHz) NMR spectrum of **8r** in DMSO-d_6

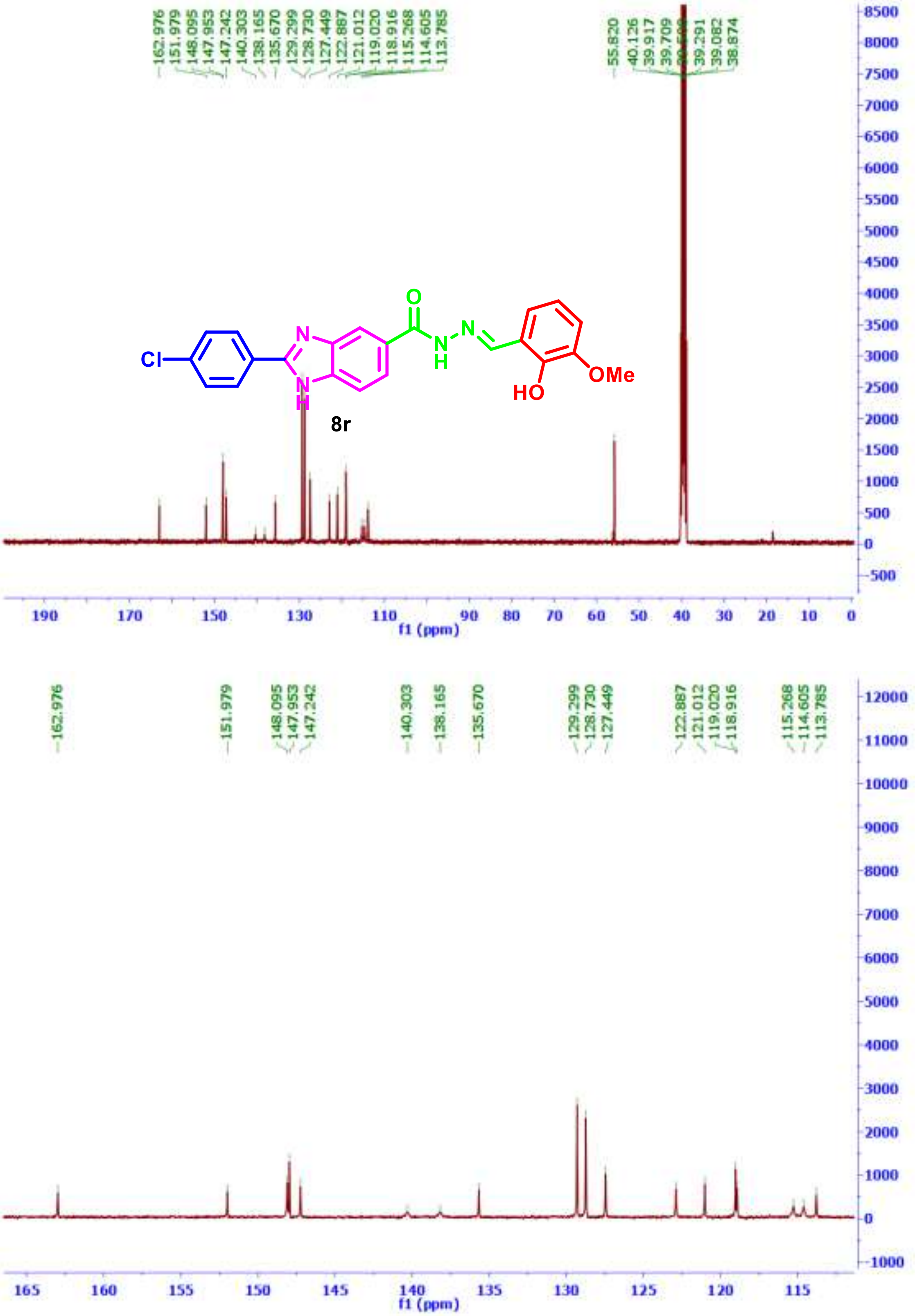

Figure S36. ^{13}C (100 MHz) NMR spectrum of **8r** in DMSO-d_6

2-(4-Chlorophenyl)-*N*'-(3-hydroxy-4-methoxybenzylidene)-1*H*-benzo[*d*]imidazole-5-carbohydrazide (**8s**)

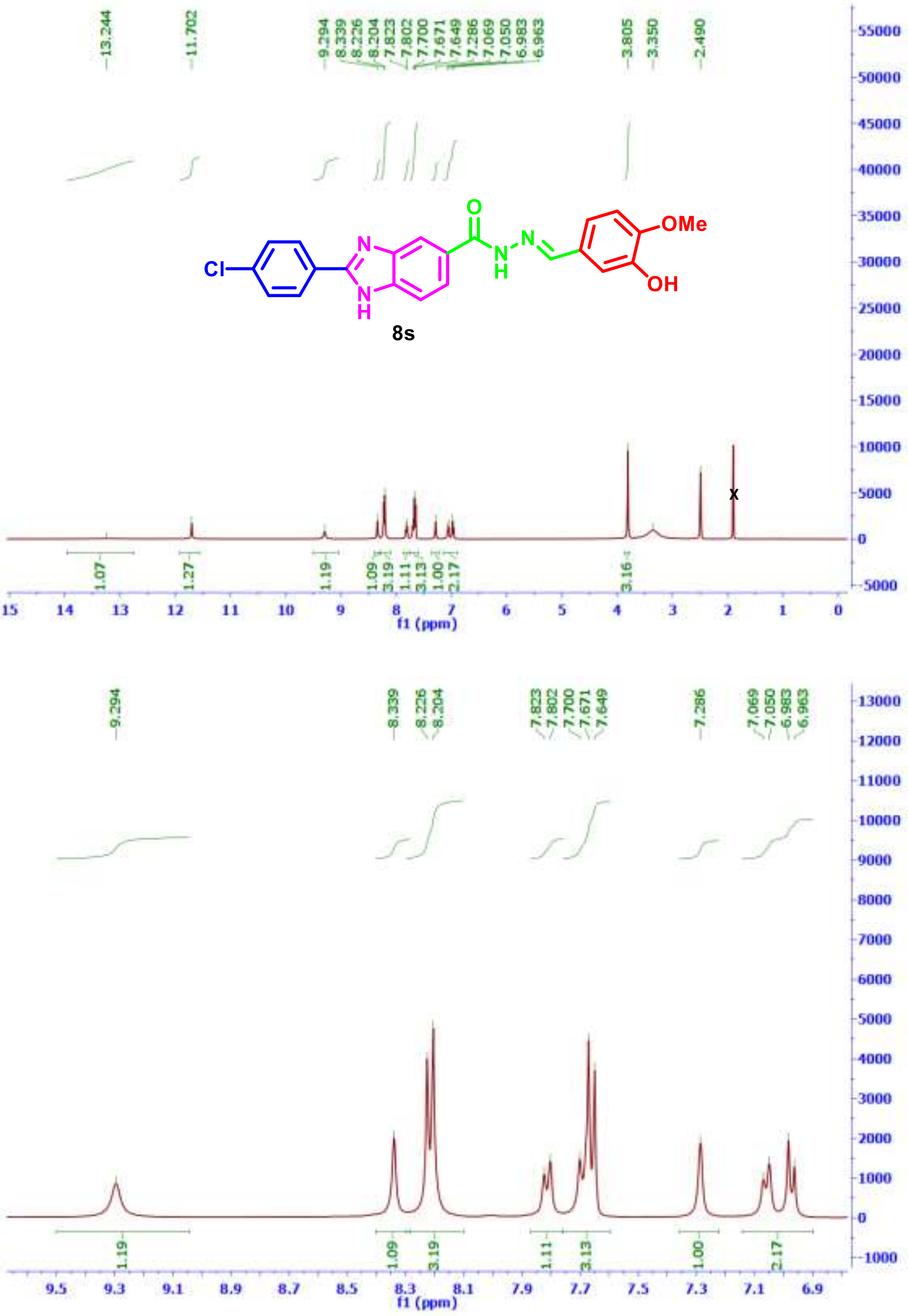

Figure S37. ^{1}H (400 MHz) NMR spectrum of **8s** in DMSO-d_6

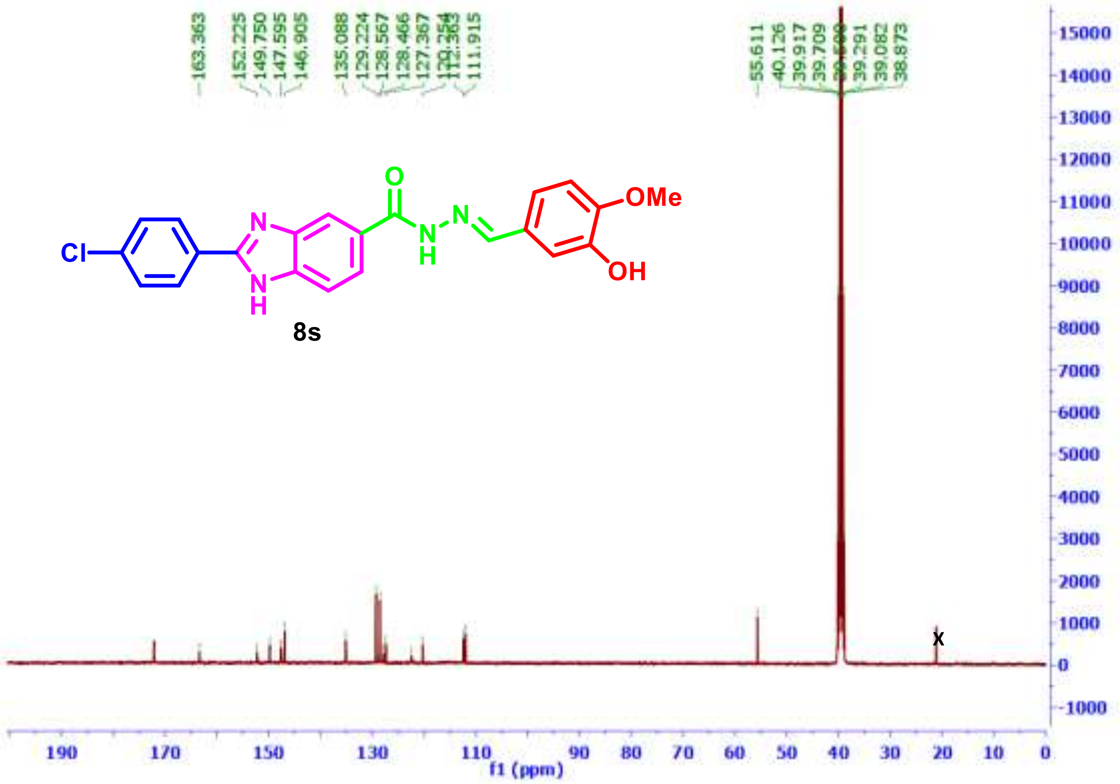

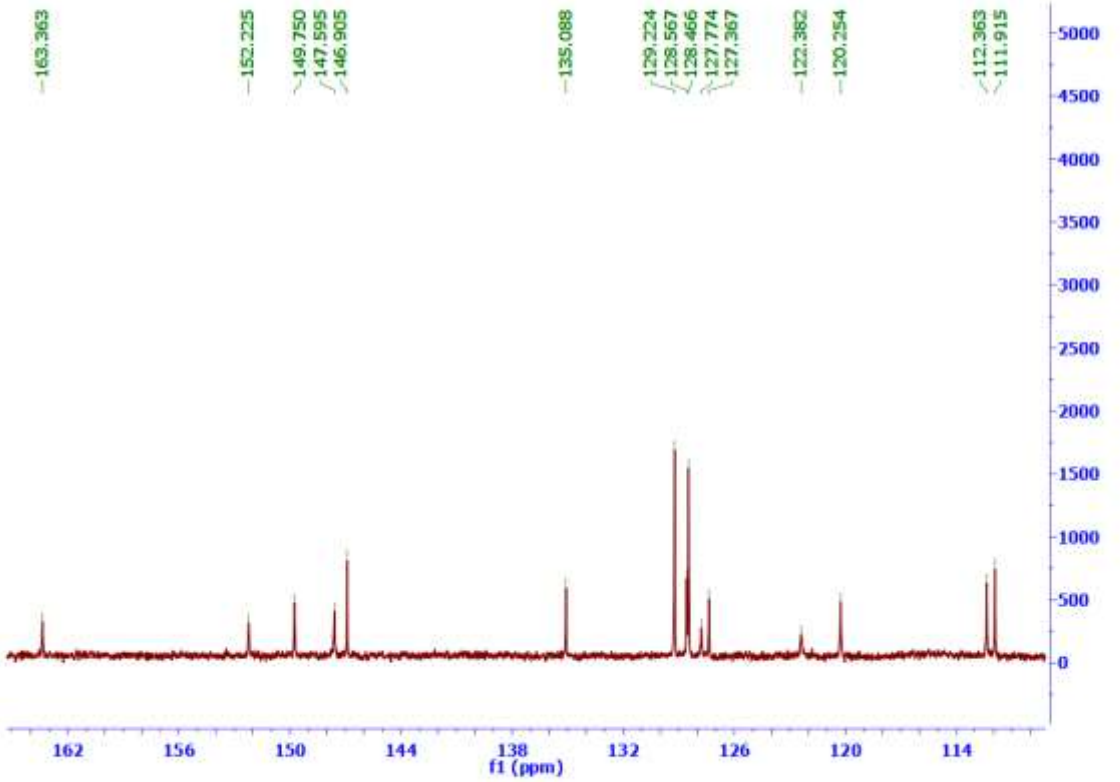

Figure S38. ^{13}C (100 MHz) NMR spectra of **8s** in DMSO-d_6

2-(4-Chlorophenyl)-*N'*-(2,5-dimethoxybenzylidene)-1*H*-benzo[*d*]imidazole-5-carbohydrazide (**8t**)

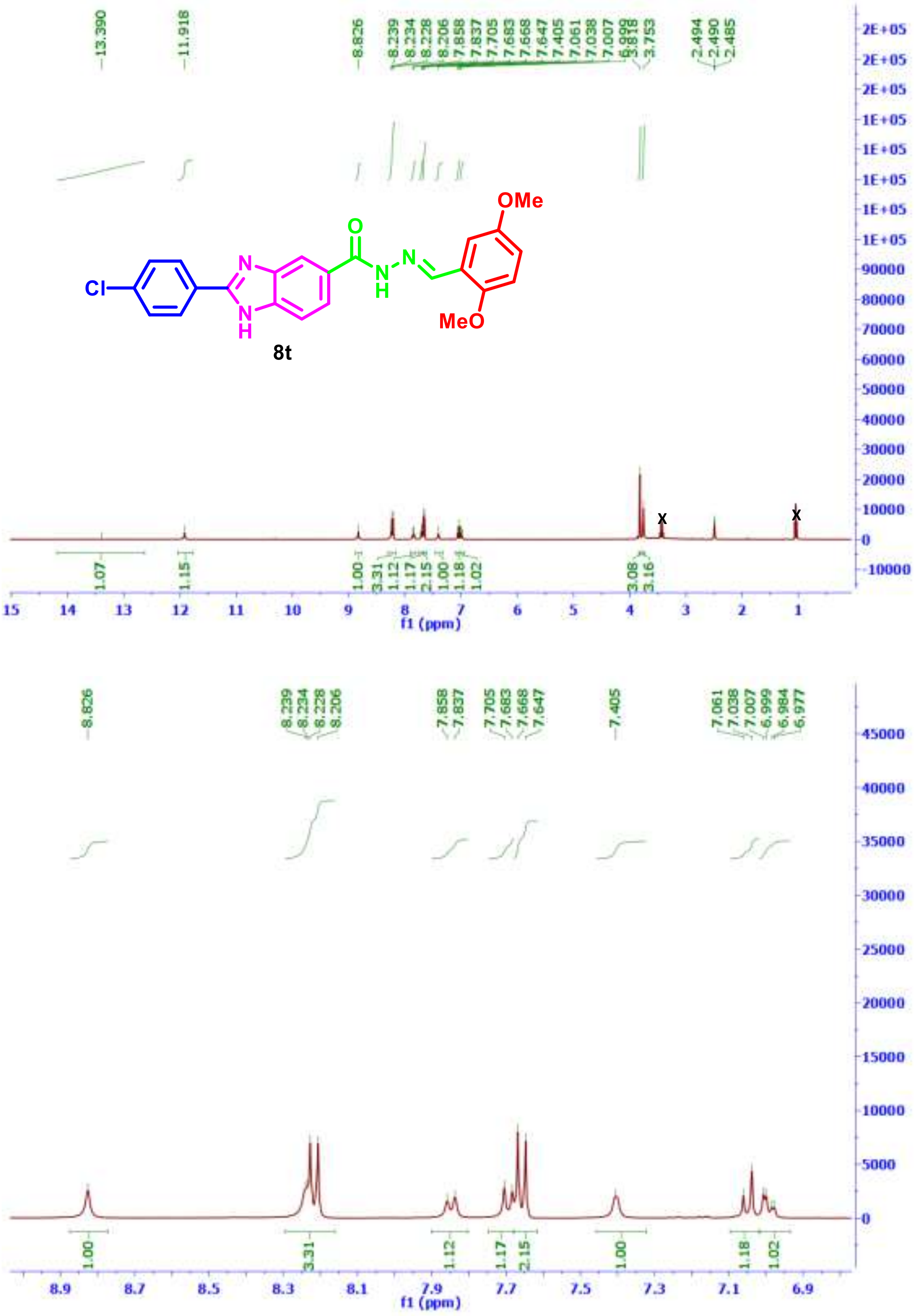

Figure S39. ^{1}H (400 MHz) NMR spectrum of **8t** in DMSO-d_6

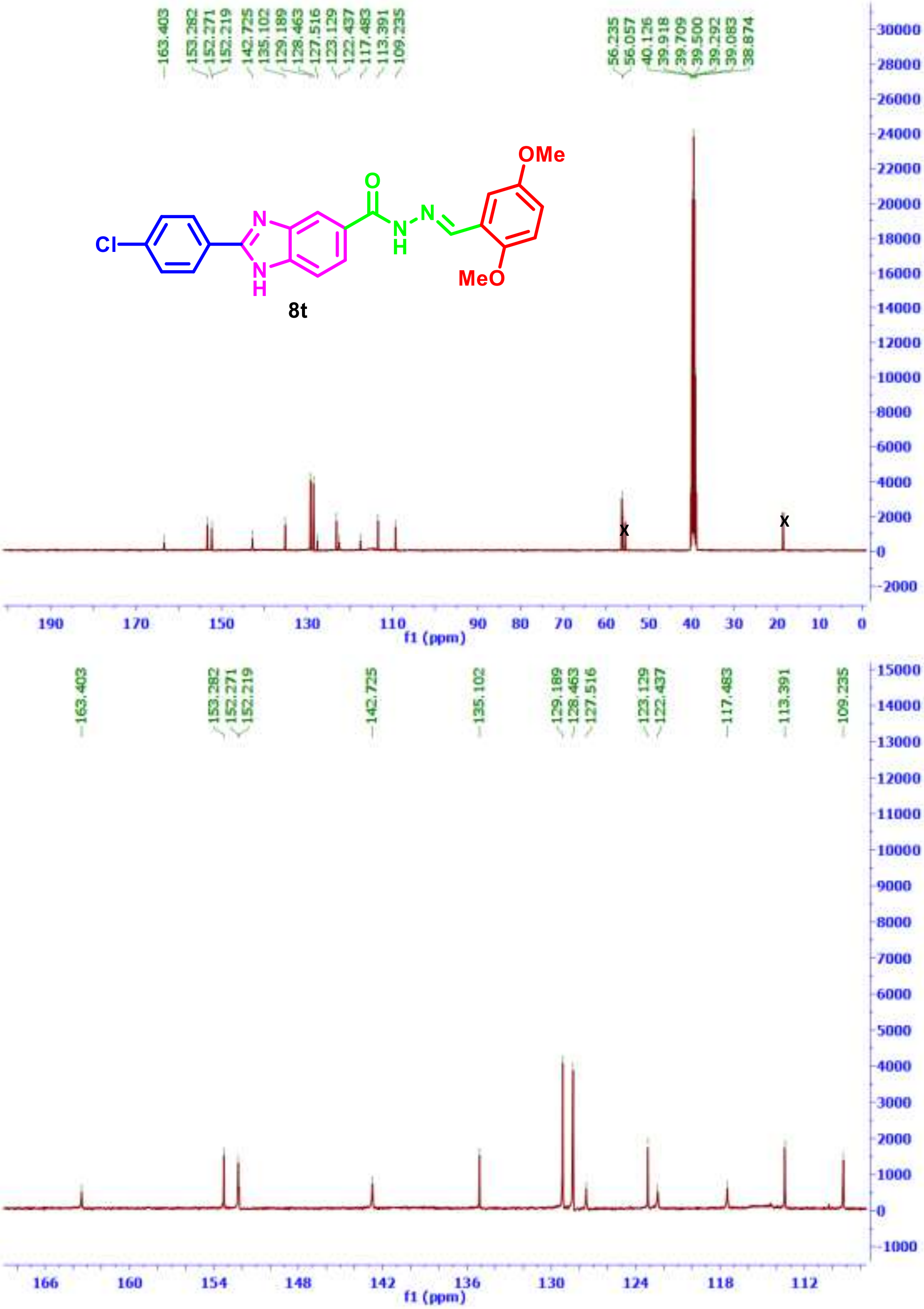

Figure S40. ^{13}C (100 MHz) NMR spectrum of **8t** in DMSO-d_6

2-(4-Chlorophenyl)-*N*'-(3,4,5-trimethoxybenzylidene)-1*H*-benzo[*d*]imidazole-5-carbohydrazide (**8u**)

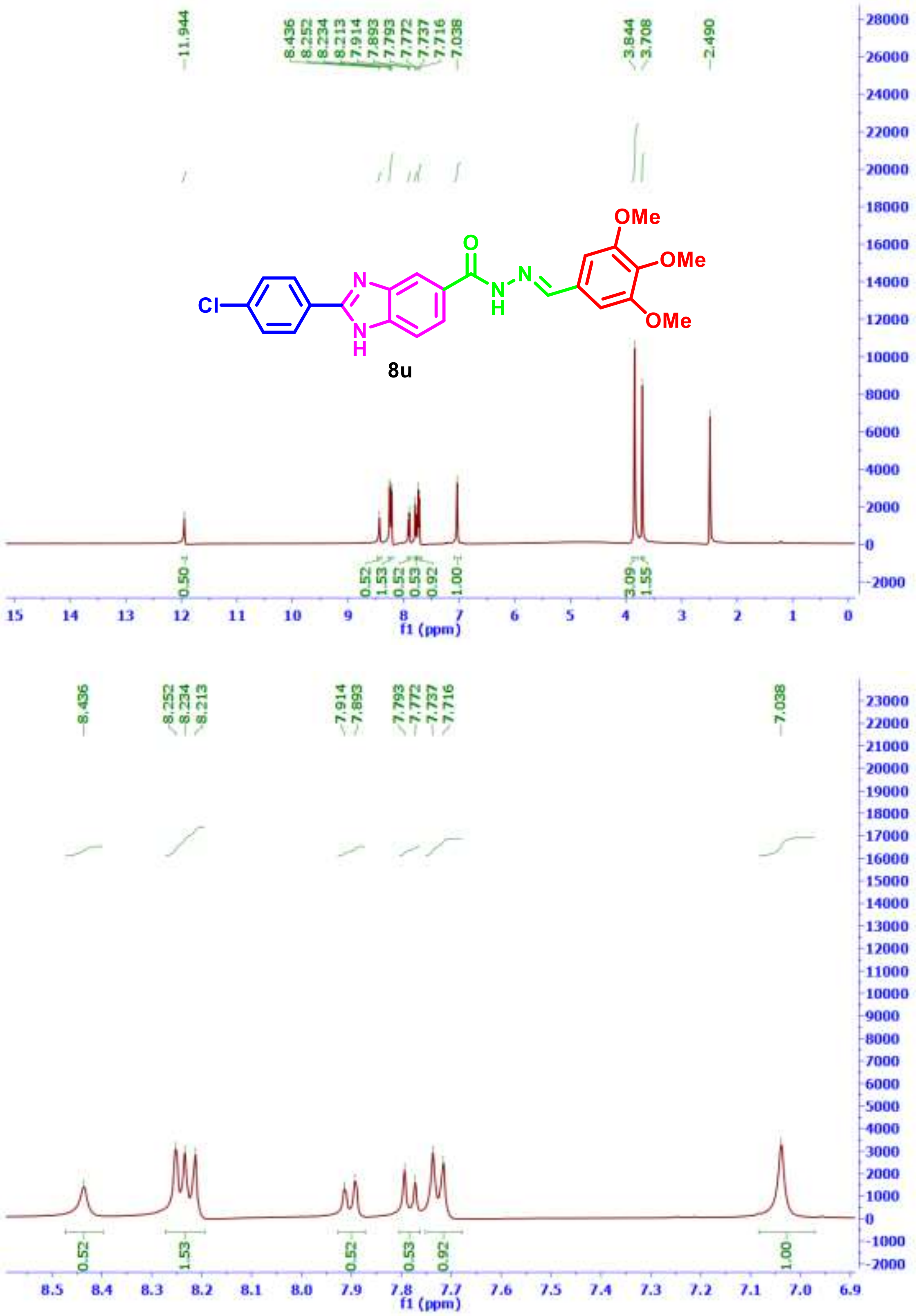

Figure S41. ^{1}H (400 MHz) NMR spectrum of **8u** in DMSO-d_6.

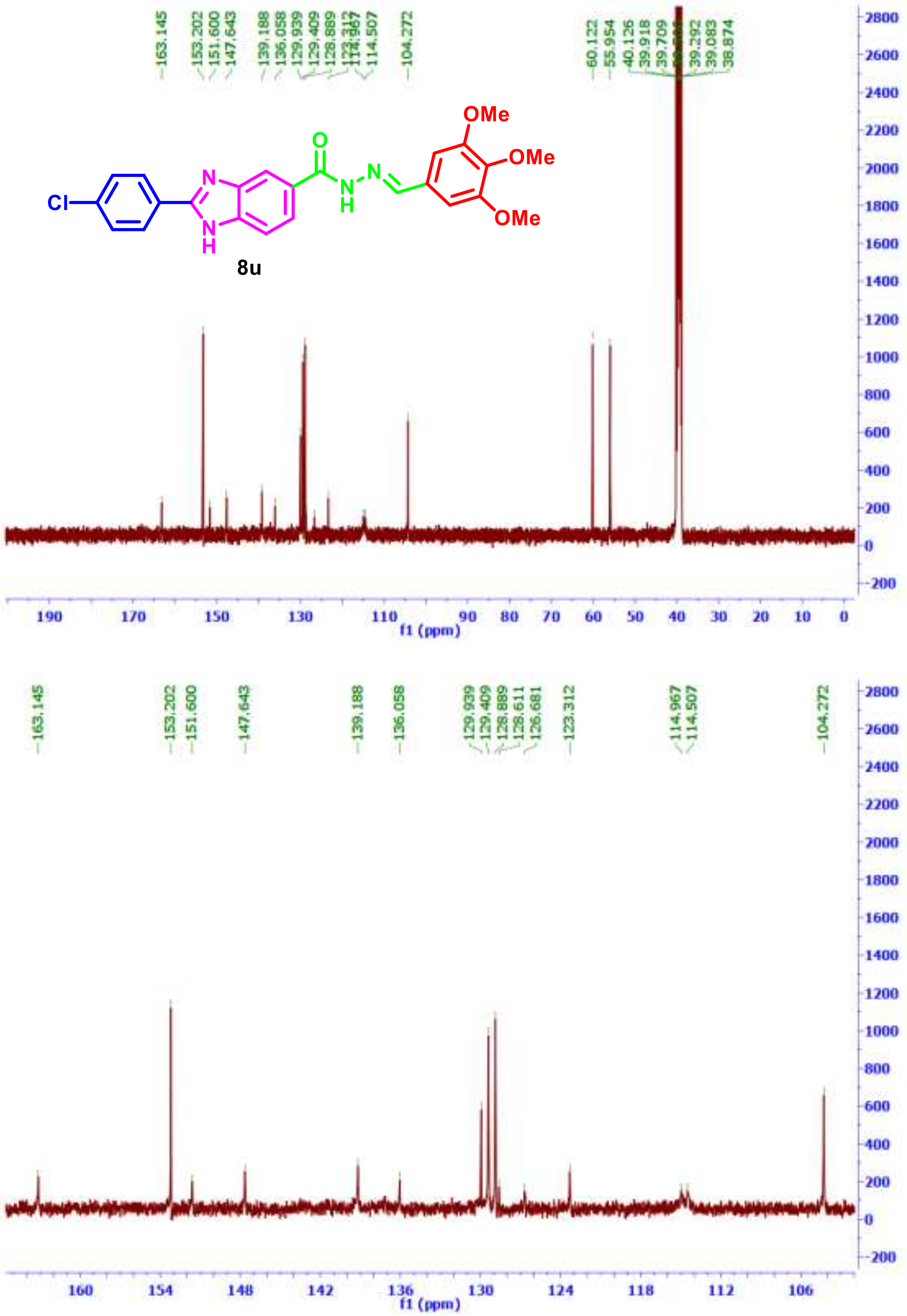

Figure S42. ^{13}C (100 MHz) NMR spectrum of **8u** in DMSO-d_6

2. Biochemical kinase assay procedure

The inhibitory activity against different kinases was determined using kinase kits (VEGFR-2 cat ID: 40325), (FGFR-1 cat ID: 40210), (BRAF (WT) cat. ID: 78316); purchased from BPS Biosciences and kinase-Glo Max luminescence kinase assay kit (Promega). Sorafenib was used as a reference multi-kinases inhibitor.

The assay was carried out according to the protocol provided by the manufacturer. A stock solution of the synthesized derivatives in 100% DMSO was prepared. Subsequently, the compounds were diluted to 10% DMSO.

A master mixture was prepared according to the number of wells. For VEGFR-2 and FGFR-1 biochemical assays each well include 6 µl of 5x Kinase Buffer 1 + 1 µl of 500 µM ATP + 1 µl of PTK Substrate (Poly-Glu,Tyr 4:1) (10 mg/ml) + 17 µl of distilled water. For BRAF assay each well include 6 µl of 5x Kinase Buffer 1 + 1 µl of ATP (500 µM) + 10 µl of 5X Raf substrate + 8 µl of distilled water. 1x Kinase buffer 1 was prepared by mixing 600 µl of 5x Kinase Buffer 1 with 2400 µl water to give 3 mL. Kinases were thawed on ice and were diluted with 1x Kinase Buffer 1.

To start the biochemical reaction, 25 µL of the master mixture was added to each well in 96 well plate. 5 µL of 10% DMSO was added to positive control wells and blank wells. 5 µL of diluted inhibitor was added to each well labelled with the test inhibitor so that the final concentration of DMSO is 1% in all reactions. Then 20 µL of (1 ng / µl) in case of VEGFR-2 and FGFR-1 or (2.5 ng/µl) in case of BRAF in 1x kinase buffer was added to positive control wells and wells labelled with the inhibitor, while 20 µL 1x kinase buffer 1 was added to the blank wells.

The plate was incubated at 30 ^{o}C for 45 min. Subsequently, 50 µl of Kinase-Glo Max luminescence reagent was added to each well and the plate was covered with aluminum foil and incubated at room temperature 15 min. Finally, the luminescence was recorded using multimode microplate reader. Kinase activity assays were performed in duplicate at each concentration.

The luminescence data were analyzed as follows. The difference between luminescence intensities in the absence of kinase (Lu_t) and in the presence of kinase (Lu_c) was defined as 100 % activity ($Lu_t – Lu_c$). Using luminescence signal (Lu) in the presence of the compound, % activity was calculated as: % activity = $\{(Lu_t - Lu)/(Lu_t - Lu_c)\}\times100\%$. The

concentration of the test compounds required to reduce the kinase activity by 50% was determined from dose-response curves and recorded as their IC_{50}.

3. Dose response curves of 8u on VEGFR-2, FGFR-1 and BRAF

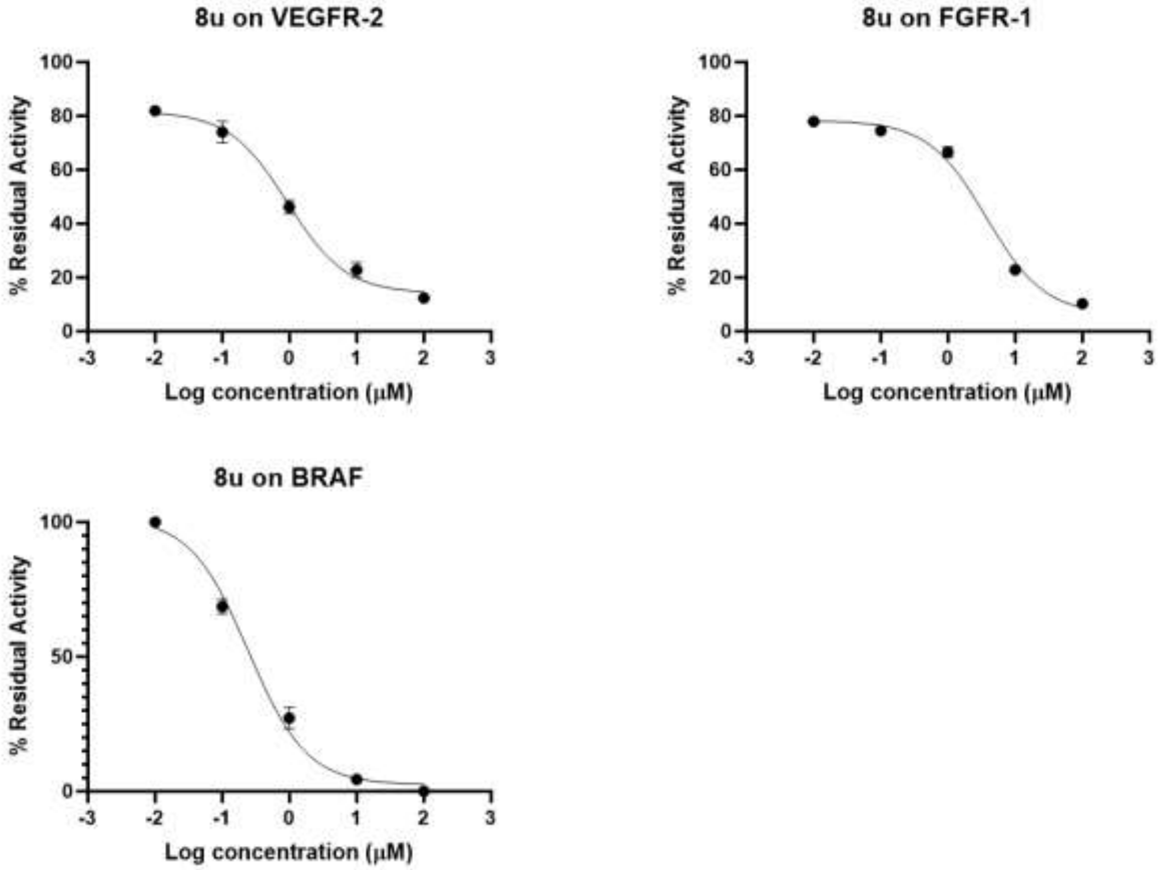

Figure S43. Dose response curves of **8u** on diverse kinases

4. Screening of cytotoxic activity against a panel of sixty human tumor cell lines

The selected compounds by NCI were evaluated for their anticancer activity in a two-stage process. First, these compounds were screened against the full NCI 60 cell lines panel at a single high dose of 10 µM. Then, the output from the single dose screen is reported as a mean graph. Second, compounds exhibiting significant growth inhibition were evaluated against the 60 cell panel at five different minimal concentrations.

Assay protocol. The human tumor cell lines of the cancer screening panel are grown in RPMI 1640 medium containing 5% fetal bovine serum and 2 mM L-glutamine. For a typical screening experiment, cells are inoculated into 96 well microtiter plates in 100 µL at plating densities ranging from 5000 to 40,000 cells/well depending on the doubling time of individual cell lines. After cell inoculation, the microtiter plates are incubated at 37 ^{o}C, 5% CO_2, 95% air and 100% relative humidity for 24 h prior to addition of the experimental drugs.

After 24 h, two plates of each cell line are fixed in situ with TCA, to represent a measurement of the cell population for each cell line at the time of drug addition (Tz). The experimental drugs are solubilized in dimethyl sulfoxide at 400-fold the desired final maximum test concentration and stored frozen prior to use. At the time of drug addition, an aliquot of frozen concentrate is thawed and diluted to twice the desired final maximum test concentration with complete medium containing 50 µg/mL gentamicin. Additional four, 10-fold or ½ log serial dilutions are made to provide a total of five drug concentrations plus control.

Aliquots of 100 mL of these different drug dilutions are added to the appropriate microtiter wells already containing 100 mL of medium, resulting in the required final drug concentrations. Following drug addition, the plates are incubated for an additional 48 h at 37 ^{o}C, 5% CO_2, 95% air, and 100% relative humidity. For adherent cells, the assay is terminated by the addition of cold TCA. Cells are fixed in situ by the gentle addition of 50 µL of cold 50% (w/v) TCA (final concentration, 10% TCA) and incubated for 60 min at 4 ^{o}C. The supernatant is discarded, and the plates are washed five times with tap water and air dried. Sulforhodamine B (SRB) solution (100 mL) at 0.4% (w/v) in 1% acetic acid is added to each well, and plates are incubated for 10 min at room temperature. After staining, unbound dye is removed by washing five times with 1% acetic acid and the plates are air dried. Bound stain is subsequently solubilized with 10 mM trizma base, and the absorbance is read on an

automated plate reader at a wavelength of 515 nm. For suspension cells, the methodology is the same except that the assay is terminated by fixing settled cells at the bottom of the wells by gently adding 50 µl of 80% TCA (final concentration, 16% TCA). Using the seven absorbance measurements [time zero, (Tz), control growth, (C), and test growth in the presence of drug at the five concentration levels (Ti)], the percentage growth is calculated at each of the drug concentrations levels.

Percentage growth inhibition is calculated as: [(Ti Tz)/(C–Tz)] x 100 for concentrations for which Ti>/=Tz and [(Ti-Tz)/Tz] x 100 for concentrations for which Ti < Tz.

Three dose response parameters are calculated for each experimental agent. Growth inhibition of 50% (GI_{50}) is calculated from [(Ti -Tz)/(C–Tz)] x 100 = 50, which is the drug concentration resulting in a 50% reduction in the net protein increase (as measured by SRB staining) in control cells during the drug incubation. The drug concentration resulting in total growth inhibition (TGI) is calculated from Ti = Tz. The LC_{50} (concentration of drug resulting in a 50% reduction in the measured protein at the end of the drug treatment as compared to that at the beginning) indicating a net loss of cells following treatment is calculated from [(Ti–Tz)/Tz] x 100 = $^{-}$50. Values are calculated for each of these three parameters if the level of activity is reached; however, if the effect is not reached or is exceeded, the value for that parameter is expressed as greater or less than the maximum or minimum concentration tested. Results for each compound were reported as a mean graph of the percent growth of the treated cells when compared to the untreated control cells. There after obtaining the results for one dose assay, analysis of historical Development Therapeutics Programme (DTP) was performed and compounds which satisfies predetermined threshold inhibition criteria is selected for NCI full panel 5 dose assay.

5. Dose response curves of the 2,5-diaryl benzimidazole conjugates on NCI cancer cell lines

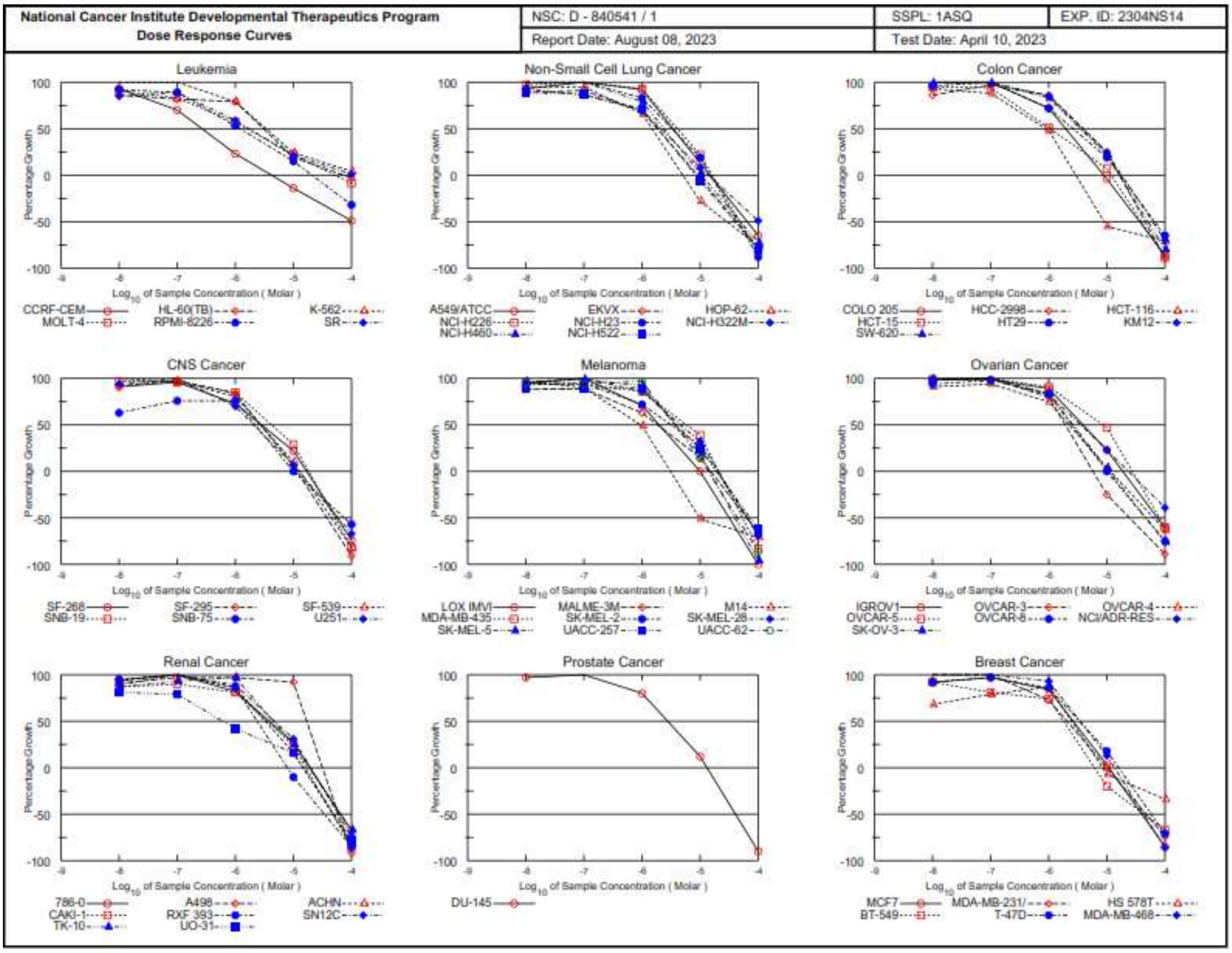

Figure S44. Dose response curves of **8a** on NCI cancer cell lines

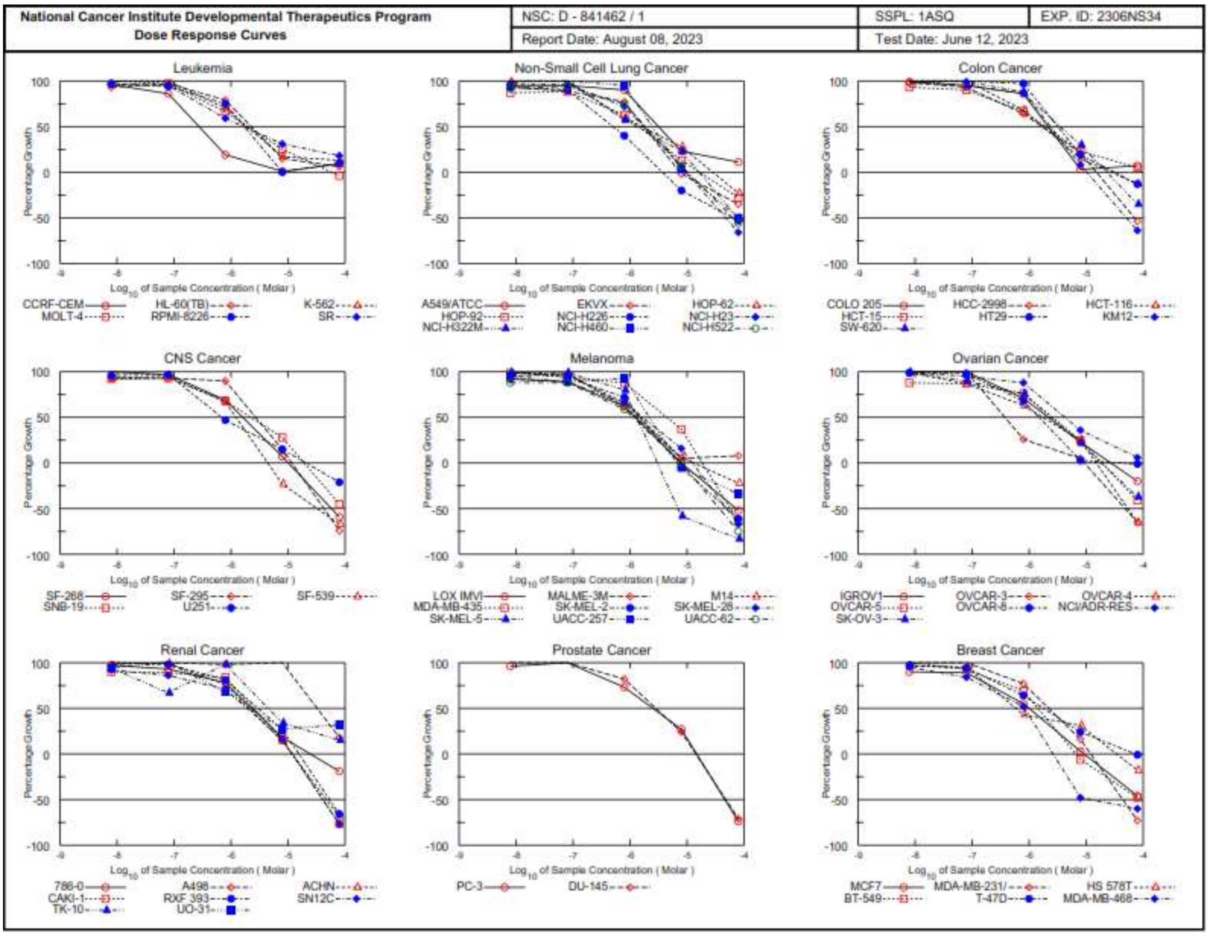

Figure S45. Dose response curves of **8d** on NCI cancer cell lines

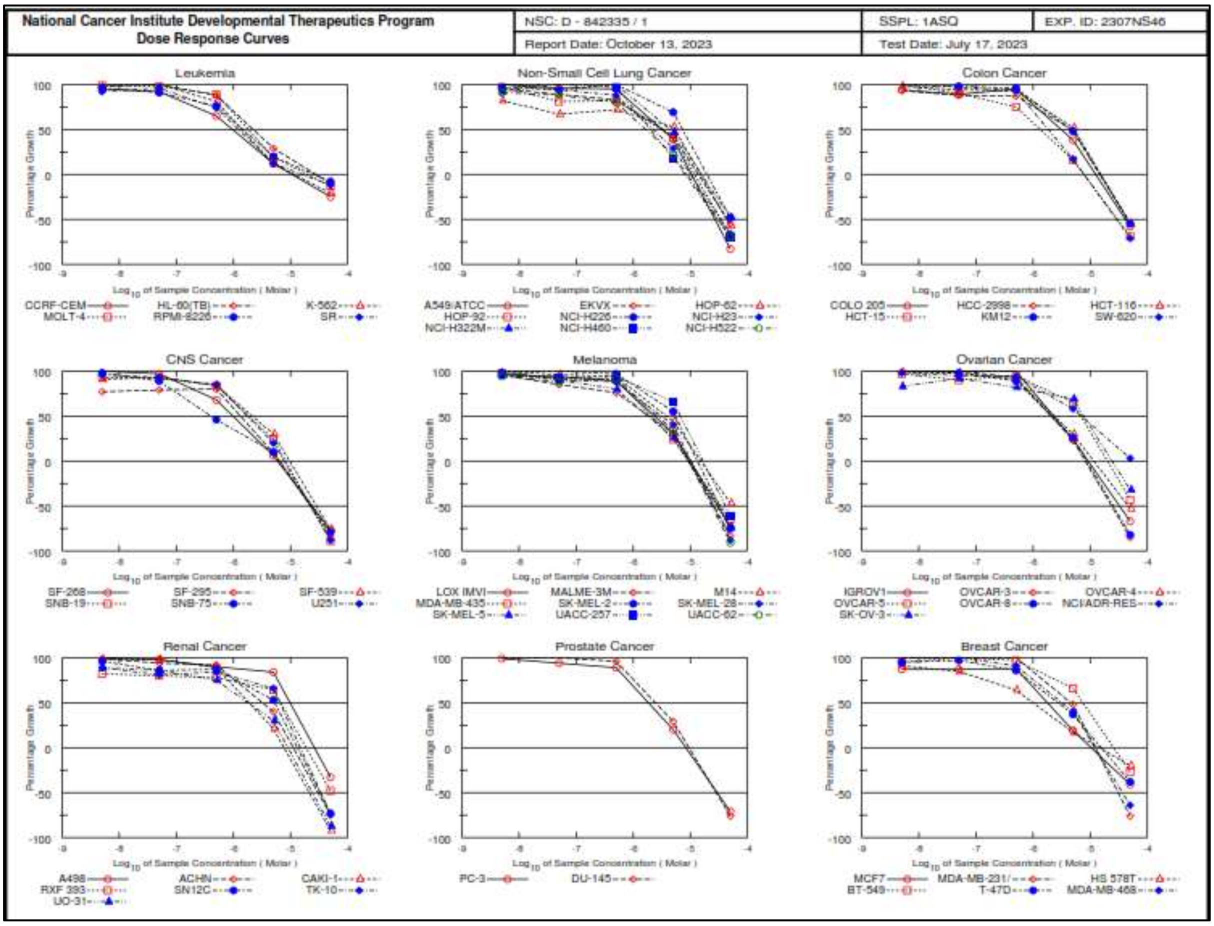

Figure S46. Dose response curves of **8h** on NCI cancer cell lines

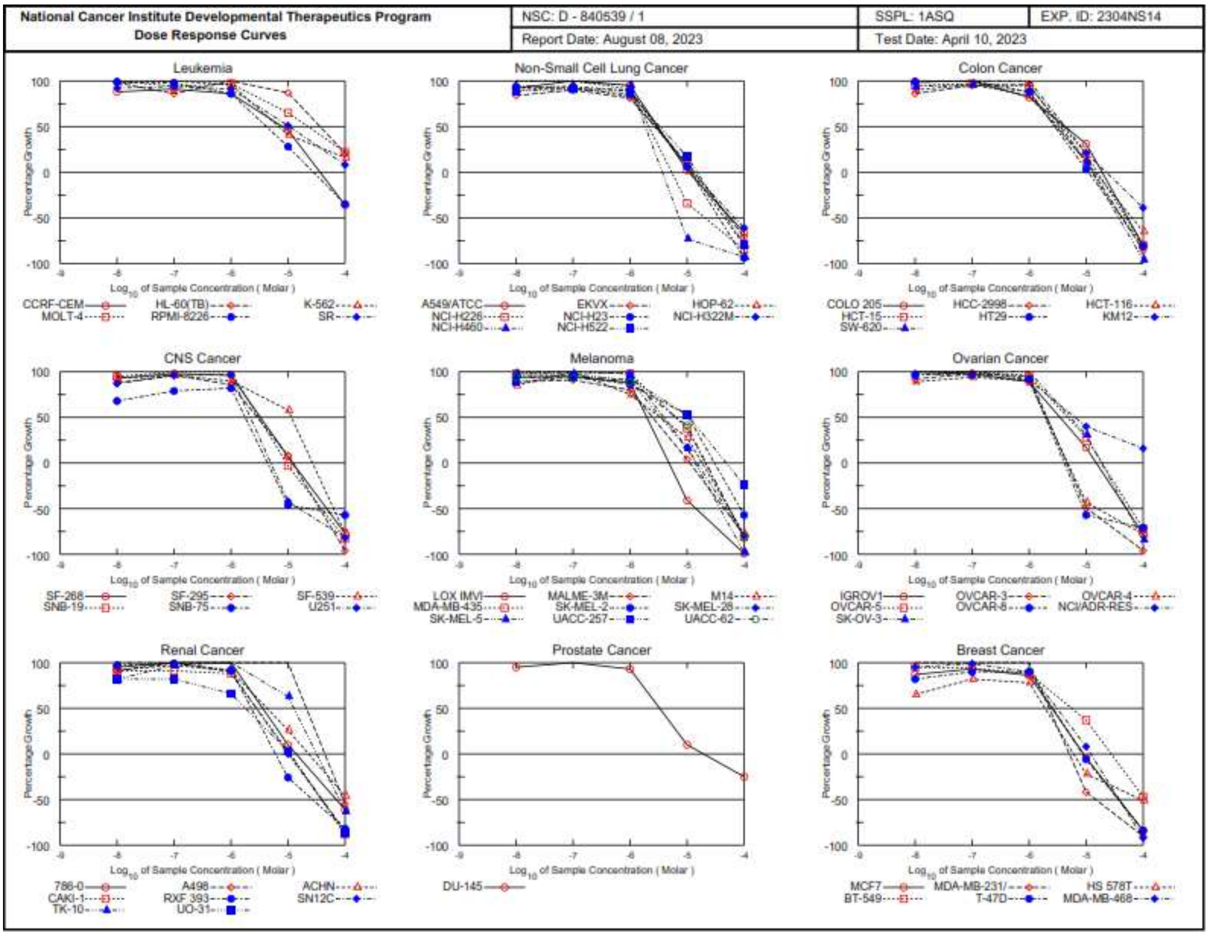

Figure S47. Dose response curves of **8j** on NCI cancer cell lines

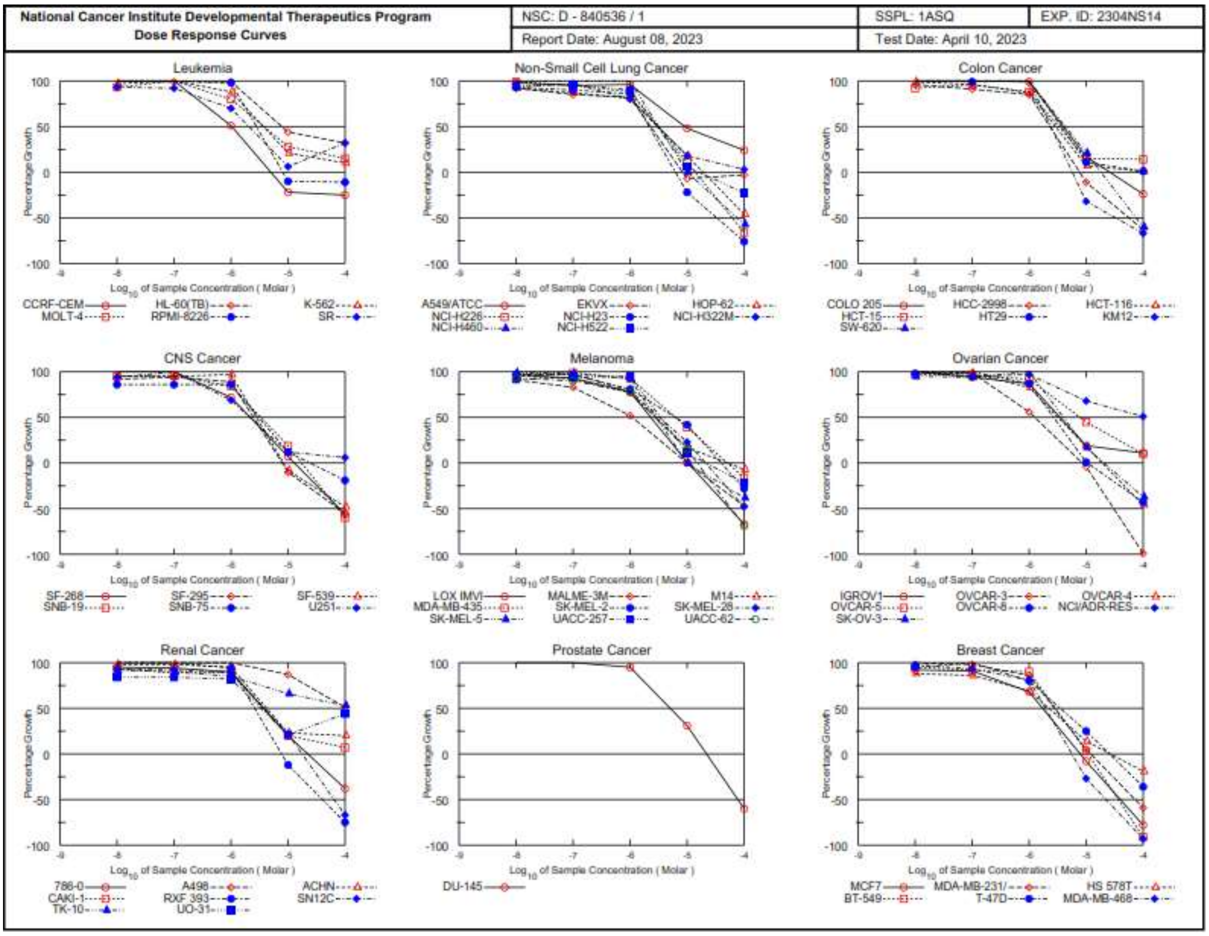

Figure S48. Dose response curves of **8k** on NCI cancer cell lines

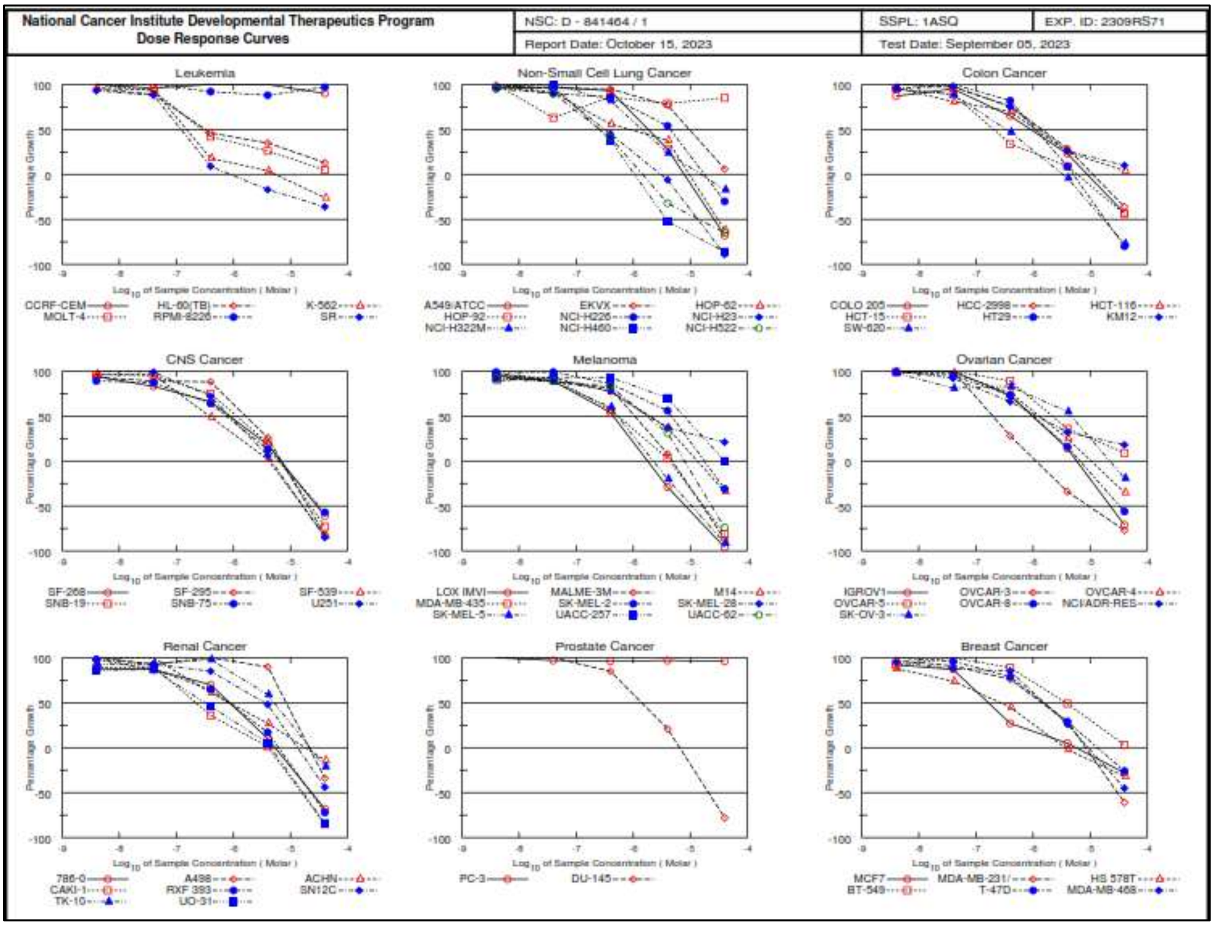

Figure S49. Dose response curves of **8o** on NCI cancer cell lines

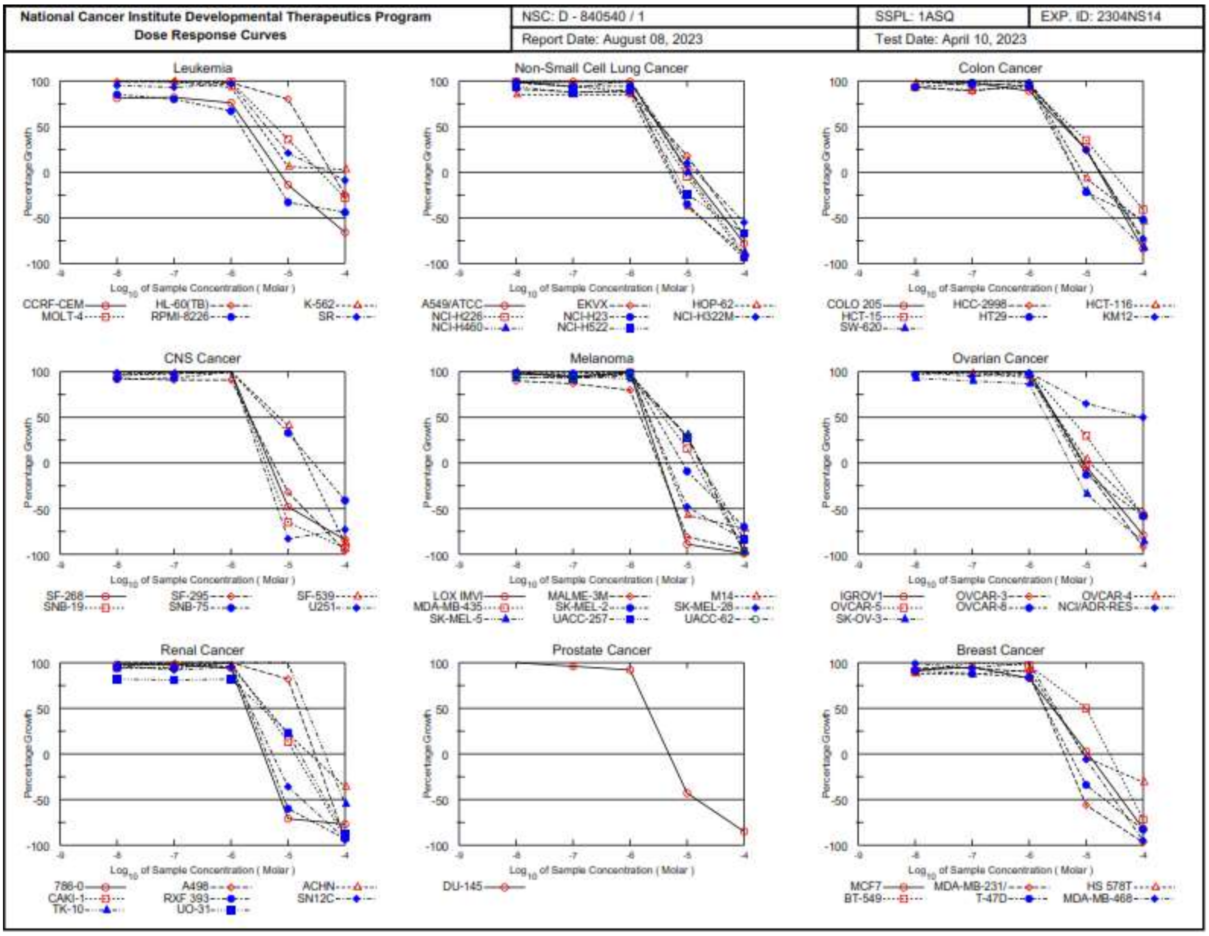

Figure S50. Dose response curves of **8q** on NCI cancer cell lines

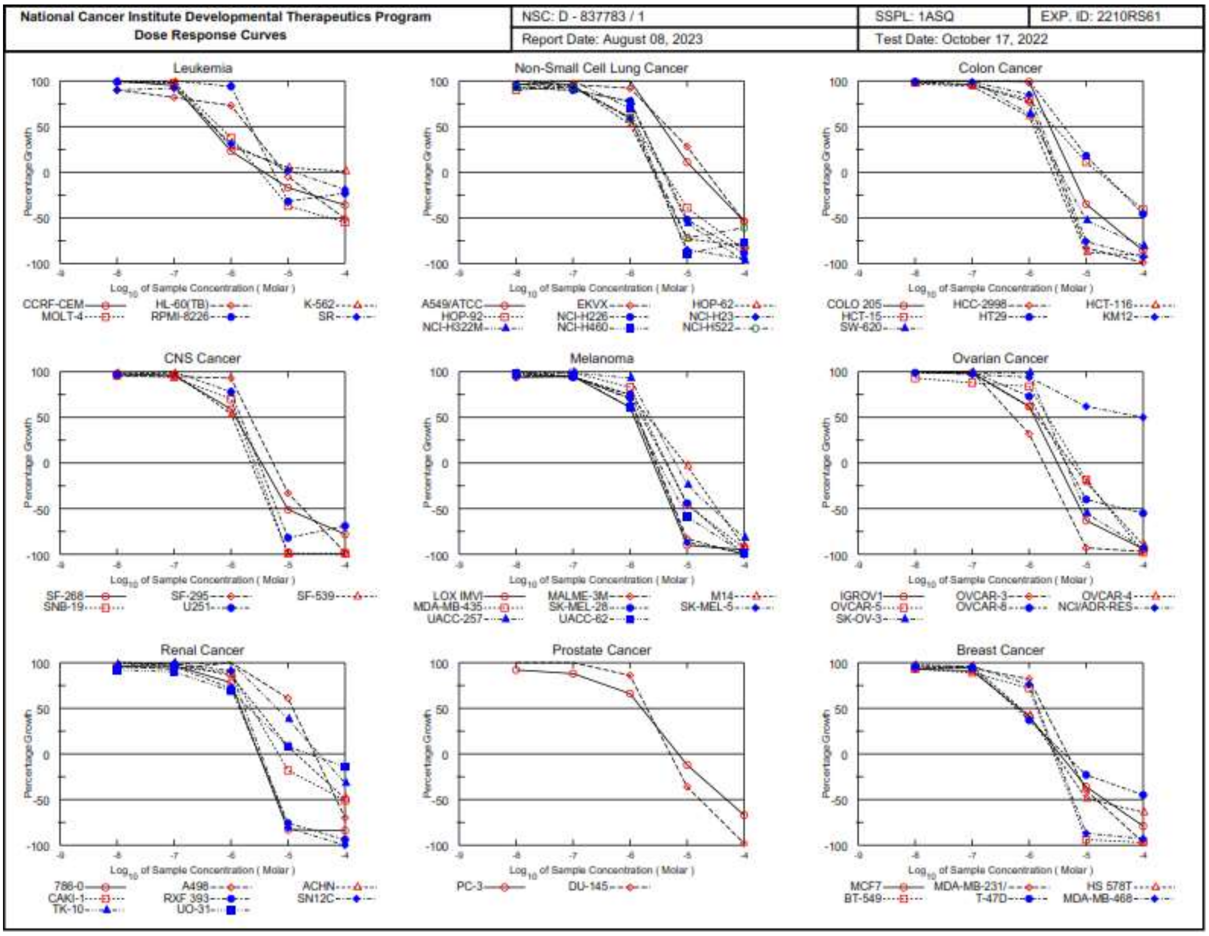

Figure S51. Dose response curves of **8r** on NCI cancer cell lines

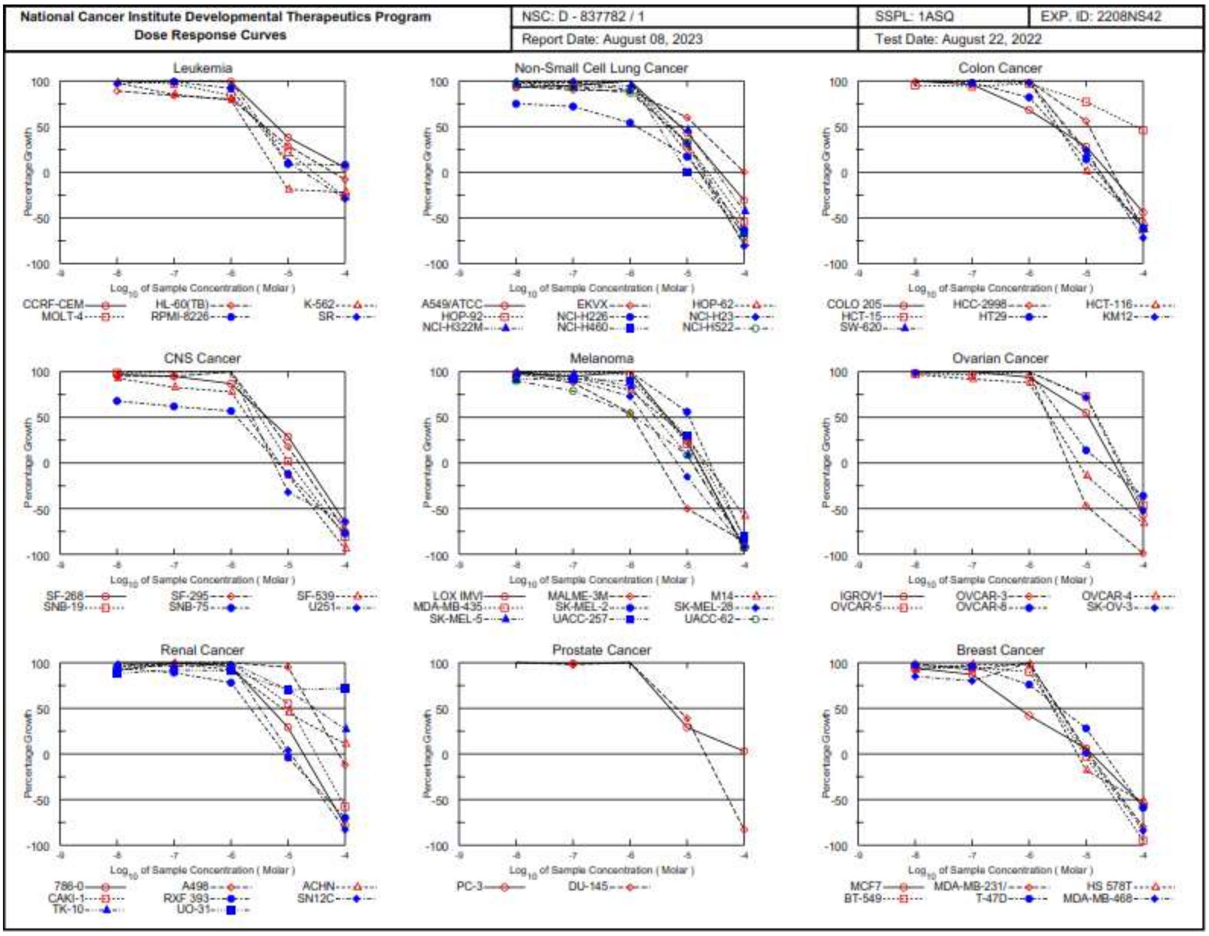

Figure S52. Dose response curves of **8u** on NCI cancer cell lines

6. Analysis of Cell Cycle Distribution

MCF-7: Breast Adenocarcinoma was obtained from Nawah Scientific Inc., (Mokatam, Cairo, Egypt). Cells were maintained in DMEM media supplemented with 100 mg/mL of streptomycin, 100 units/mL of penicillin and 10% of heat-inactivated fetal bovine serum in humidified, 5% (v/v) CO_2 atmosphere at 37 °C. After treatment with **8u** for 48h, cells (105 cells) are collected by trypsinization and washed twice with ice-cold PBS (pH 7.4). Cells are re-suspended in two milliliters of 60% ice-cold ethanol and incubated at 4ºC for 1 h for fixation. Fixed cells are washed twice again with PBS (pH 7.4) and re-suspended in 1 mL of PBS containing 50 µg/mL RNAase A and 10 µg/mL propidium iodide (PI). After 20 min of incubation in dark at 37 C, cells are analyzed for DNA contents using flow cytometry analysis using FL2 (λex/em 535/617 nm) signal detector (ACEA Novocyte™ flowcytometer, ACEA Biosciences Inc., San Diego, CA, USA). For each sample, 12,000 events are acquired. Cell cycle distribution is calculated using ACEA NovoExpress™ software (ACEA Biosciences Inc., San Diego, CA, USA).

*G0/G1-phase: This phase denotes the non-proliferating cells. Compounds with antiproliferative effect (regardless of their cytotoxicity) are expected to increase this cell population significantly.

*S-phase: This phase denotes proliferating cells and particularly the cells who are undergoing DNA synthesis step of replication. Some compounds might induce S-phase arrest and increase this cell population significantly.

*G2/M-phase: This phase indicates the final phase of cell replication (mitosis). Compounds interfering with micro-tubular spindles (stabilize or destabilize) are expected to increase this cell population significantly.

7. Apoptosis assay

Apoptosis and necrosis cell populations are determined using Annexin V-FITC apoptosis detection kit (Abcam Inc., Cambridge Science Park, Cambridge, UK) coupled with 2 fluorescent channels flowcytometry. After treatment with test compounds for 48h, cells (105 cells) are collected by trypsinization and washed twice with ice-cold PBS (pH 7.4). Then, cells are incubated in dark with 0.5 ml of Annexin V-FITC/PI solution for 30 min in dark at room temperature according to manufacturer protocol. After staining, cells are injected via ACEA

Novocyte™ flowcytometer (ACEA Biosciences Inc., San Diego, CA, USA) and analyzed for FITC and PI fluorescent signals using FL1 and FL2 signal detector, respectively (λex/em 488/530 nm for FITC and λex/em 535/617 nm for PI). For each sample, 12,000 events are acquired and positive FITC and/or PI cells are quantified by quadrant analysis and calculated using ACEA NovoExpress™ software (ACEA Biosciences Inc., San Diego, CA, USA).
